# Theoretical Exploration of the Physical-Chemical Properties of Divalent (*np* ^2^) Cation Mixing in Double Cs_2_AgBiBr_6_ Perovskite

**DOI:** 10.1021/acsomega.5c12243

**Published:** 2026-03-11

**Authors:** Iván Ornelas-Cruz, Ramiro M. dos Santos, Matheus P. Lima, Juarez L. F. Da Silva

**Affiliations:** † São Carlos Institute of Chemistry, University of São Paulo, Av. Trabalhador São-Carlense 400, 13560-970 São Carlos, São Paulo, Brazil; ‡ Department of Physics, 67828 Federal University of São Carlos, 13565-905 São Carlos, São Paulo, Brazil

## Abstract

Lead-free halide double perovskites have emerged as promising alternatives to conventional lead-based materials for photovoltaic applications, as they combine environmental compatibility with structural stability. However, their indirect band gaps limit optoelectronic performance, motivating compositional and structural optimization to achieve higher efficiency. In this work, we used density functional theory calculations to investigate complex mixed-halide double perovskites with general composition Cs_2_Ag_*x*_Bi_*x*_ *B*_*y*_ *B*'_*z*_Br_6_, where *B*,*B′* = Ge, Sn, or Pb. By coupling electronic-structure calculations with high-throughput stress-tensor optimizations across thousands of configurations, we identified the energetic and structural principles governing their stability and electronic properties. The results revealed a narrow energy distribution, indicating high structural flexibility and entropy-driven stabilization. Substitutional trends are dictated by ionic size, with larger cations reducing octahedral distortions and promoting lattice symmetry. Although all ternary mixtures exhibited positive excess energies, Sn- and Pb-rich compositions were energetically favored. The decomposition of the bond energy consistently linked the strength of the metal-halide interaction to the stability of the lattice, with increasing Ag content weakening the overall bonding. In particular, partial substitution of Ge, Sn, or Pb at the *B*-site of pristine Cs_2_AgBiBr_6_ enhanced electronic transitions and induced nonlinear bowing effects, demonstrating heterovalent substitution as an effective strategy for tuning stability and optoelectronic performance in lead-free perovskite absorbers.

## Graphical abstract

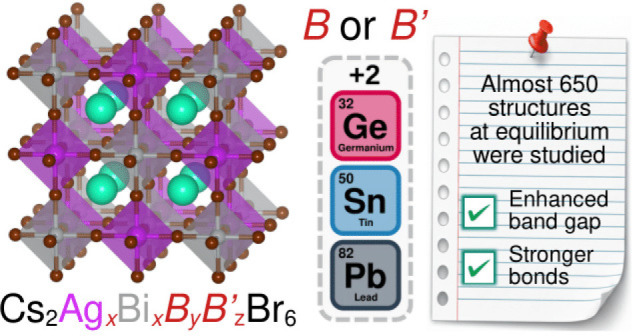

## 1 Introduction

The shift to low-carbon renewable energy places photovoltaic (PV) technology at the forefront of sustainable electricity generation, due to its carbon-free energy source (sunlight), rapid deployability, modular scalability, and falling production costs, enabling applications from small rooftop systems to multigigawatt solar farms.^1^ Current technologies include crystalline silicon (c-Si) cells, cadmium telluride (CdTe) thin films, and copper indium gallium selenide (CIGS) cells.^1,2^ Emerging PV technologies, particularly perovskite solar cells and tandem perovskite-Si modules, offer the potential for unprecedented efficiencies with economically viable manufacturing.^2,3^

Metal-halide perovskite solar cells (PSC) have achieved record power conversion efficiencies (PCEs), e.g., above 26.9%,^2^ but their large-scale deployment is limited by material instability under operating conditions and lead toxicity.^4^ These issues are most critical for the absorber layer, the metal-halide perovskite itself, which governs the performance and lifetime of the PSC. By contrast, CdTe and c-Si technologies do not exhibit comparable stability or toxicity concerns under normal or even extreme operating conditions, assuming appropriate manufacturing and recycling practices.^5–7^

The highest PCE values for PSCs (i.e., >26%) have been achieved with an absorber layer based on lead-halide perovskites (*A*Pb*X*_3_),^8^ where lead plays an essential role in achieving superior optoelectronic properties.^8^ However, lead usage also raises significant environmental and health concerns due to its toxicity.^6^ Furthermore, the challenges in PSCs are exacerbated by the thermodynamically favored decomposition pathway: *A*Pb*X*_3_(s) = *AX*(s) + Pb*X*_2_(s), which can be thermodynamically favored under extreme conditions.^9^ In principle, it is possible to address the two problems at once, i.e., replacing Pb by alternative species that yield similar electronic properties and improved thermodynamic stability. Thus, there is a unique research opportunity here, which is connected with the search for alternative Pb-free materials with higher stability under environmental conditions.

For example, through the homovalent substitution of Pb by Sn, which is less hazardous to human health,^5^ it is still possible to obtain the photoactive phase of perovskite; nevertheless, thermodynamics favors the oxidation of these metals from Sn­(II) to Sn­(IV),^10^ which is also a limitation. For these compounds, only PCE values up to 16% have been possible.^8^ Thus, there is increasing interest in more environmentally friendly metal-halide perovskite absorbers. For example, based on experience with double-perovskite oxides,^11^ in 2016 the Cs_2_AgBi*X*_6_ compound with *X* = Br or Cl was first synthesized,^12–14^ and was rapidly implemented in a PSC, obtaining a PCE < 2%.^15^ However, since the influential article by Ken and Kanatzidis,^16^ progress of this technology has remained limited (PCE < 4%),^8^ and there is still potential for further improvements. For single-junction photovoltaics (with an upper limit of 33.2%), absorber materials with a band gap from 0.80 to 1.88 eV would have, in the radiative limit, a maximum achievable PCE of 25.0 up to 33.2%.^17^ Moreover, it is desirable for an absorber material to possess a direct band gap to avoid the need for momentum phonons or to reduce the amount of material in the solar cell.^18^ However, Cs_2_AgBiBr_6_ has an indirect band gap of 1.95 eV.^12^

Therefore, to improve the photoelectronic properties of this class of compounds and based on the general chemical formula *A*_2_ *B*(I)*B′*(III)*X*_6_ (with *A* = monovalent cation, and *X* = halogen), metal substitution can be done at either *B*(I) or *B′*(III) through: (i) isoelectronic species, e.g., mixing species from groups IB and VA at *B*(I) and *B′*(II), respectively; or (ii) nonisoelectronic species, e.g., mixing species from groups IA and IB at *B*(I), and species from groups IIIA and VA at *B′*(II). Changes in the magnitude and character of the material’s band gap are observed in the latter case, owing to the distinct orbital hybridization between the species involved in the metal-halide bonds.^19^ In this context, heterovalent substitutions have been carried out in the past using divalent metals, namely, Cu­(II),^20^ Zn­(II),^21^ Mn­(II),^22,23^ Ge­(II),^21^ Sn­(II),^21,24^ and Pb­(II),^25^ to mimic the optoelectronic properties of simple lead-halide compounds (*A*Pb*X*_3_), and exploiting the versatility of perovskite materials.

In this work, we systematically investigated complex perovskite mixtures Cs_2_Ag_*x*_Bi_*x*_ *B*_*y*_ *B'*_*z*_Br_6_ (*B*, *B′* = Ge, Sn, Pb) using high-throughput stress-tensor and electronic-structure calculations based on density functional theory (DFT) to map configurational space and assess structural flexibility, energetic stability, and optoelectronic properties. The substitution of divalent metals produced a narrow energy distribution of configurations, indicating configurational degeneracy and entropy-driven stabilization. The ionic size of the substituents strongly governed the lattice response, with larger cations reducing octahedral distortions. The bonding-energy analysis showed a strong link between bonding strength and lattice stability, and a higher Ag content systematically weakened the metal-halide interactions. Substitutional Ge, Sn, or Pb narrowed the wide indirect band gap of pristine Cs_2_AgBiBr_6_, inducing bowing and enhancing electronic transitions. In general, chemical heterovalent substitution emerges as an effective route to tune the stability and optical response in halide double perovskites, enabling the design of stable, efficient, and lead-free photovoltaic absorbers.

## 2 Theoretical Approach and Computational Details

### 2.1 Total Energy Calculations

All calculations were based on the DFT framework, as implemented in the Vienna Ab initio Simulation Package (VASP),^26^ version 5.4.4. For the exchange-correlation energy functional, we employed the semilocal Generalized Gradient Approximation (GGA) as proposed by Perdew–Burke–Ernzerhof (PBE).^27^ It has been well established that plain DFT-PBE commonly yields equilibrium lattice parameters slightly larger than experimental values.^28^ Thus, to improve the description of the equilibrium lattice parameters, which can be achieved by considering the attractive dispersive interactions between atoms, primarily arising from halogen species within the double-perovskite Cs_2_Ag_*x*_Bi_*x*_ *B*_*y*_ *B'*_*z*_Br_6_ solid,^29^ we used the Grimme semiempirical D3 correction for van der Waals (vdW) interactions.^30^ The D3 correction adds an attractive term to the total energy, contributing to the reduction of equilibrium lattice parameters and bringing them closer to experimental values.^30,31^ The description of core-valence electron interactions was performed using the Projector Augmented-Wave (PAW) method,^32,33^ while the Kohn–Sham (KS) states are represented by a plane wave expansion.

Investigating all possible structural configurations within the bulk of double-perovskite Cs_2_Ag_*x*_Bi_*x*_ *B*_*y*_ *B'*_*z*_Br_6_ presents a significant challenge due to the large number of configurations, even when employing small unit cells. For example, using a 2 × 2 × 2 unit-cell, which comprises 40 atoms, results in 11578 structures across 13 compositions, which represents a substantial computational cost. Consequently, we implemented the following approaches: (i) single-point total energy calculations to select the most important structural configurations through the analysis of the relative energy distribution profile and (ii) equilibrium structure optimizations through minimization of the stress tensor for selected configurations based on specific energy criteria.

To evaluate the total energy of all possible configurations of each mixture using single-point total energy calculations (i.e., without structural optimizations), we considered the following computational parameters: (i) a cutoff energy of 422.363 eV, which is 12.5% higher than the maximum recommended cutoff energy among the selected PAW projectors for all chemical species (Ag, Bi, Ge, Sn, Pb, Br, and Cs), i.e., 1.125 × ENMAX_max_; (ii) an energy convergence criterion of 1 × 10^–4^eV to converge the KS self-consistent field (SCF) iterations; (iii) only the Γ point for the integration of the Brillouin-zone; and (iv) shared equilibrium lattice constants of the compounds Cs_2_AgBiBr_6_ ^13^ and CsGeBr_3_.^34^

For the selected configurations, identified from the analysis of the relative total energy profile, we optimized the equilibrium volumes by iterative optimization of the stress tensor and atomic forces. All structural optimizations were performed under the constraint of cubic symmetry. In this stage, we used a plane-wave cutoff energy of 563.151 eV (corresponding to 1.5 × ENMAX_max_), which is required for the slow convergence of the stress-tensor as a function of the number of plane-waves. After obtaining the equilibrium structures, we performed a final refinement of the atomic positions for all compounds using a reduced cutoff energy of 422.363 eV. In these calculations, all equilibrium configurations satisfied the convergence criterion that the residual force on each atom was smaller than 0.025 eV/Å, while the KS SCF cycle at each ionic step was terminated once the total energy change was below 1 × 10^–5^eV. Brillouin-zone integrations were performed using a Monkhorst–Pack **k**-point mesh of 2 × 2 × 2.^35^

Using optimized equilibrium geometries, single-point calculations were performed to determine electronic and optical properties, specifically band structures, density of states, absorption coefficient, and the crystal orbital Hamilton population (COHP), employing a plane-wave cutoff energy of 422.363 eV. Band-structure calculations, including those incorporating spin-orbit coupling, were performed using a mesh of 4 × 4 × 4 **k**-points. Subsequently, to obtain the remaining properties, a denser 6 × 6 × 6 **k**-point mesh was used to achieve an accurate description of the electronic states in the vicinity of the valence-band maximum. For these calculations, an SCF energy convergence threshold of 1 × 10^–6^ eV was used to improve the numerical accuracy and reliability of the resulting data.

### 2.2 Discussion of the Composition of the Atomistic Models

The metal-halide perovskite structure, conventionally described by the chemical formula *ABX*_3_, is governed by the requirements of overall charge neutrality and stereochemical compatibility among the constituent ions, where *A* denotes a monovalent cation, *B* a divalent metal cation, and *X* a halide anion. These criteria are typically assessed on the basis of the oxidation state for monatomic species or the effective charge for polyatomic species, within the framework of a hard-sphere model approximation employing octahedral,^36^ Goldschmidt,^37^ or τ tolerance factors.^38^ According to these considerations, the allowed species are *A* = Cs^+^, CH_3_NH_3_^+^, or HC­(NH_2_)_2_^+^; *B* = Ge^2+^, Sn^2+^, or Pb^2+^; and *X* = Cl^–^, Br^–^, or I^–^. In the present work, all investigated perovskite-like compounds were restricted to *A* = Cs^+^ and *X* = Br^–^.

The simple cubic unit cell of the perovskite compound Cs*B*Br_3_ contains a single formula unit and has a *Pm*3̅*m* symmetry, as shown in Figure 1. Therefore, a 2 × 2 × 2 supercell built from a simple cubic cell contains eight formula units (Cs_8_ *B*_8_Br_24_), or equivalently, four units of Cs_2_ *B*_2_Br_6_ (Figure 1). In this supercell model, mixtures involving more than one divalent *B* cation, namely *B* and *B′*, can occupy the centers of the octahedra in the corner-sharing cuboctahedral array. In that case, the supercubic cell represents the compound Cs_2_ *B*_*x*_ *B'*_*y*_Br_6_, with *x* + *y* = 2.

**Figure 1:**
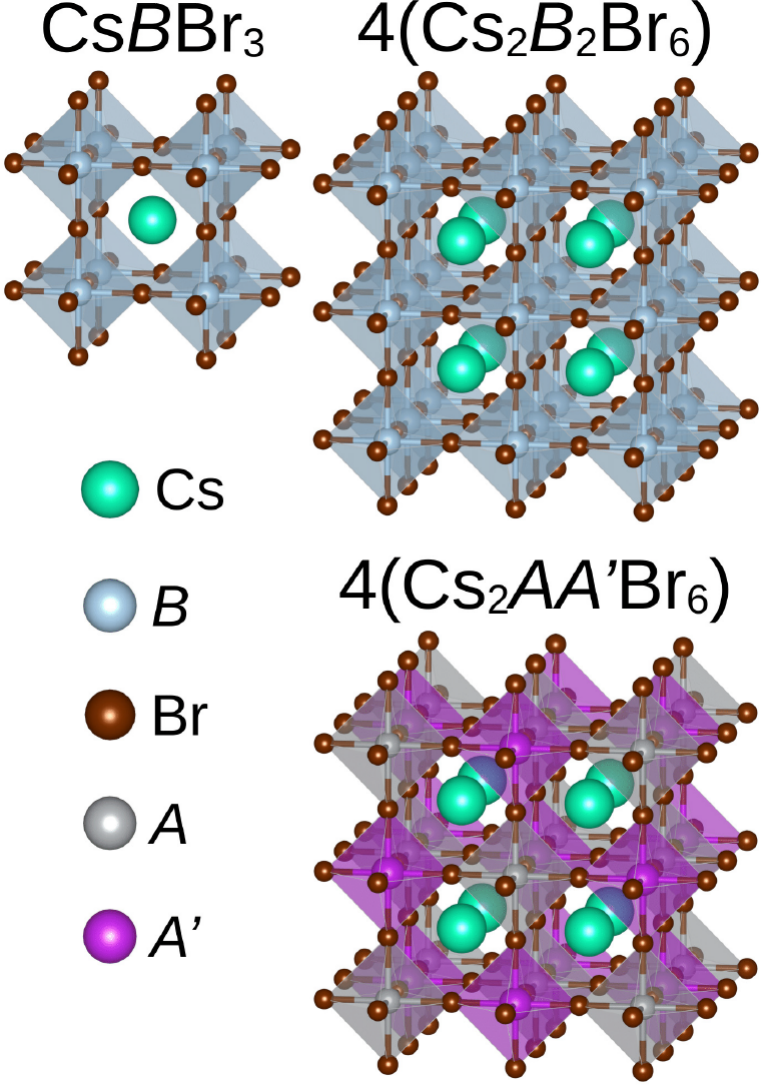
Molecular representation of the unit cells used to model the compounds discussed in the text: simple cubic and supercubic structures for Cs*B*Br_3_, and a cubic double-perovskite structure for Cs_2_ *AA′*Br_6_. The number of chemical units within each cell is indicated above the corresponding structure (note that 4­(Cs_2_ *B*_2_Br_6_) = Cs_8_ *B*_8_Br_24_).

In halide double perovskites, instead of divalent cations, metals with different oxidation states occupy the centers of the octahedra. Considering that metals *A* and *A′* exhibit oxidation states of +1 and +3, respectively, a charge-balanced perovskite-like compound has the chemical formula Cs_2_ *AA′*Br_6_, where *A* = Rb^+^, Cs^+^, or Ag^+^, and *A′* = In^3+^, Sb^3+^, or Bi^3+^.^39^ Assuming a cubic unit cell with *Fm*3̅*m* symmetry, the Cs_2_ *AA′*Br_6_ structure contains four formula units per unit cell (Figure 1).

Based on this framework, a 40-atom cubic unit cell can model a mixture of the form Cs_2_ *A*_*w*_ *A'*_*x*_ *B*_*y*_ *B'*_*z*_Br_6_ (*w* + *x* + *y* + *z* = 2). Alternatively, because the supercell contains four formula units, the composition can be written as Cs_8_ *A*_*m*_ *A'*_*n*_ *B*_*i*_ *B'*_*j*_Br_24_, with *m* = 4*w*, *n* = 4*x*, *i* = 4*y*, and *j* = 4*z* (*m* + *n* + *i* + *j* = 8). Hence, for each mixture studied in this work, eqs 1 and 2 define the composition, while eqs 3 and 4 define the number of atoms of each species within the unit cell. Equation 5 then establishes the relationships between them, i.e.,

$${\mathrm{Cs}}_{2}A_{w}A_{x}^{\prime }B_{y}B_{z}^{\prime }{\mathrm{Br}}_{6},$$ 1

w+x+y+z=2; 2

$${\mathrm{Cs}}_{8}A_{m}A_{n}^{\prime }B_{i}B_{j}^{\prime }{\mathrm{Br}}_{24},$$ 3

m+n+i+j=8; 4

m=4w,n=4x,i=4y,j=4z. 5

For all compositions investigated in this study (Table 1), the species *A* and *A′* are fixed to Ag and Bi, respectively. In addition, the following constraints were imposed on each atomistic model:

1. Due to the oxidation states of *A* (+1) and *A′* (+3), *w* = *x* or equivalently *m* = *n* must hold in all mixtures, to ensure equal amounts of *A* and *A′*.
2. For mixtures with one divalent metal, *B* = *B′* = Ge, Sn, or Pb.
3. For mixtures with two divalent metals, *B* ≠ *B'*, *B* = Ge or Sn, and *B′* = Sn or Pb, therefore, *B′* > *B* for all cases.

**Table 1:** Main Information about the Structural Models of the Mixtures Studied in This Work^a^

	Cs_2_Ag_*w*_Bi_*x*_ *B*_*y*_ *B'*_*z*_Br_6_				Cs_8_Ag_*m*_Bi_*n*_ *B*_*i*_ *B'*_*j*_Br_24_					
*B & B*′ conditions	*w*	*x*	*y*	*z*	*m*	*n*	*i*	*j*	*N_P_*	*N_G_*
*B* = *B′* = 0	1.00	1.00	0.00	0.00	4	4	0	0	70	10
*B* = *B′* ≠ 0	0.75	0.75	0.25	0.25	3	3	1	1	560	21
	0.50	0.50	0.50	0.50	2	2	2	2	420	15
	0.25	0.25	0.75	0.75	1	1	3	3	56	3
*B* ≠ *B′*, both ≠0	0.75	0.75	0.25	0.25	3	3	1	1	1120	29
	0.50	0.50	0.25	0.75	2	2	1	3	1680	24
	0.50	0.50	0.50	0.50	2	2	2	2	2520	30
	0.50	0.50	0.75	0.25	2	2	3	1	1680	26
	0.25	0.25	0.25	1.25	1	1	1	5	336	9
	0.25	0.25	0.50	1.00	1	1	2	4	840	11
	0.25	0.25	0.75	0.75	1	1	3	3	1120	13
	0.25	0.25	1.00	0.50	1	1	4	2	840	16
	0.25	0.25	1.25	0.25	1	1	5	1	336	12
total *N*_P_ and *N*_G_									11,578	219

a *N*_P_ was obtained from permutations of the metal species at the different *B*-site positions of the perovskite structure. *N*_G_ was obtained after calculating the total energies of the structures generated by metal permutations and grouping them using the criterion Δ*E*_tot_ < 0.10 eV (2.5 meV/atom).

### 2.3 Generation and Selectivity Criteria of the Atomic Structure Configurations

To generate all possible model configurations of the mixtures, we performed all permutations of the metal species across the eight available *B*-sites within the supercubic unit cells. In eqs 6 and 7, each *N*_*j*_ denotes the number of atoms of metal *j* (with *j* = Ag, Bi, *B*, or *B′*) within the perovskite structure.

$$N_{P}(N_{1},N_{2},\cdot \cdot \cdot ,N_{j})=\frac{N!}{N_{1}!N_{2}!\cdot \cdot \cdot N_{j}!},$$ 6

$$\sum_{j}N_{j}=N=8.$$ 7

However, based on eqs 1–5 and Table 1, we define three main cases for *B* and *B′*:

1. When *B* = *B′* = 0, we have only two different metals, Ag and Bi, where *N*_Ag_ = *N*_Bi_ = *m* = *n*.
2. When *B* = *B′*, we have three different metals, Ag, Bi, and *B*, where *N*_Ag_ = *N*_Bi_ = *m* = *n*, and *N*_*B*_ = *i* + *j*.
3. When *B* ≠ *B′*, we have four different metals, Ag, Bi, *B*, and *B′*, where *N*_Ag_ = *N*_Bi_ = *m* = *n*, *N*_*B*_ = *i*, and *N*_*B′*_ = *j*.

In Table 1, we report the number of distinct structures obtained by permuting the metal species within the perovskite framework. To optimize the use of computational resources, the complete set of 11578 configurations generated using eq 6 was constructed using divalent Ge and Sn cations: Ge was used for compositions containing a single divalent metal, while both Ge and Sn were included for compositions containing two distinct divalent metals. All structures were constrained to share the same lattice parameter as Cs_2_AgBiBr_6_ and the supercubic unit cell of CsGeBr_3_ (i.e., the 2 × 2 × 2 supercell derived from the primitive simple-cubic cell), namely *a*_0_ = 11.27 Å.^13,34^ Although a very large number of atomistic models are, in principle, compatible with a given nominal composition, crystalline solids typically adopt the periodic arrangement of the lowest-energy configuration. Consequently, we defined an energetic selection criterion to identify a representative subset of atomistic models for each composition.

As a reference for the achievable accuracy of the present DFT-based calculations (about 1.0 meV/atom compared to all-electron codes),^40^ we grouped the models using an energy threshold of Δ*E* < 0.10 eV (2.5 meV/atom). Consequently, all structures belonging to a particular group {*n*_*k*_} met the energy criterion Δ*E*_tot_^*ij*^ = *E*_tot_^*i*^ – *E*_tot_^*j*^ < 2.5 meV/atom, with *i* ≠ *j* and *i*, *j* ∈ {*n*_*k*_}. For consistency, if |{*n*_*k*_}| = *N*_*k*_, where *k* = 1, 2, ···, *N*_G_, then *N*_1_ + *N*_2_ + ··· + *N*_*N*_G__ = *N*_P_.

However, it is important to note the following: Sn is, of course, larger in atomic size than Ge; for example, their ionic radii are *r*_Sn_ = 1.15 Å and *r*_Ge_ = 0.73 Å, respectively.^41^ Also, *a*_0_ = 11.61 Å for the supercubic structure of CsSnBr_3_,^42^ which is 3.02% larger than that of CsGeBr_3_ and Cs_2_AgBiBr_3_. However, it has been shown that considering volume relaxation in an energy curve generated by the total energies of different structures mainly results in a vertical shift of the curve, while its overall shape is preserved.^43^ Furthermore, our energy criterion was applied to select representative models from groups of structures that are equivalent to each other at the level of theory employed in this work. Therefore, different approximate volumes in each compound (e.g., through Vegard’s law) might change the selection of representatives, but the number of groups, *N*_G_, will remain the same.

Finally, asymmetric distortions were introduced into each representative structure by randomly displacing Br atoms by up to 0.50Å prior to structural relaxation. This approach can yield a more accurate description of perovskite systems,^44^ since energy gains can result from octahedral symmetry breaking, particularly in mixtures containing Ge.^45^ An important limitation of our models is their size, which, in all cases, results in fully miscible systems (e.g., phase segregation or defect formation are outside the scope of the present work). In addition, we avoided the term “alloy” and instead used “mixtures” to refer to these systems, since we did not apply standard alloy-theory approaches (e.g., the SQS method) to generate the structures.^46^ Figure 2 summarizes Sections 2.2 and 2.3.

**Figure 2:**
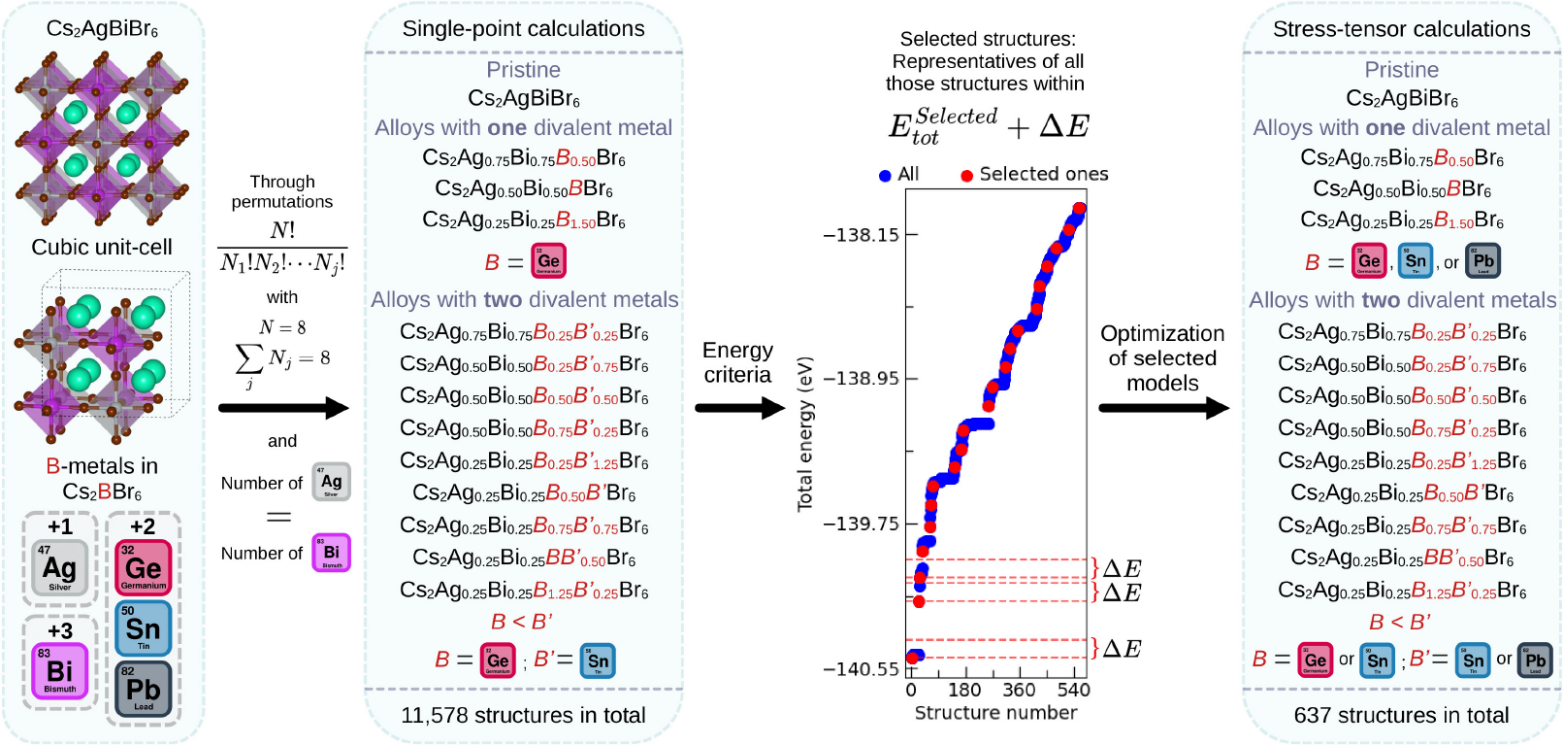
Diagram summarizing the procedure implemented in this work for the screening and selection of the representative structures for each studied mixture.

## 3 Results and Discussion

### 3.1 Characterization of the Single-Point Relative Total Energy Profiles

As shown in Table 1, analyzing the relative total energies (with Δ*E*_tot_ < 0.10 eV) of frozen-structure calculations reduced the number of metal-permuted structural configurations from 11578 (*N*_P_) to 219 (*N*_G_), i.e., 1.89% of the full configuration space. Thus, a single representative structure can capture several hundred distinct atomic arrangements that are degenerate in total energy within the chosen precision, as supported by the energy histograms in Figure 3. Only these representative configurations are then fully relaxed to determine equilibrium volumes by minimizing the stress tensor and atomic forces, making the overall hierarchical strategy computationally tractable.

**Figure 3:**
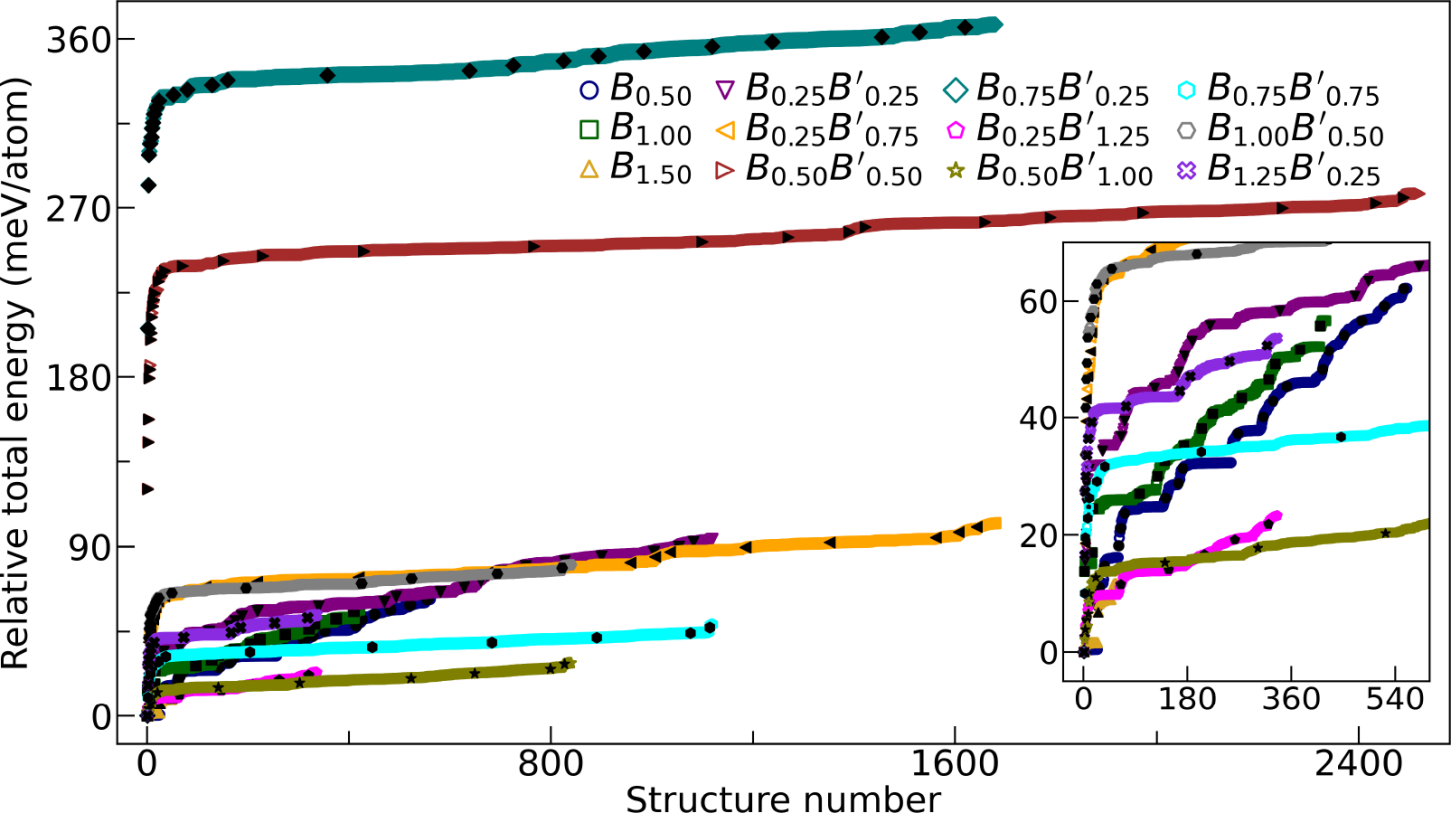
Relative total energies with respect to the lowest-energy structure, Δ*E*_tot_^*i*^ = *E*_tot_^*i*^ – *E*_tot_^lowest^, for all structures generated through metal permutations and evaluated using single-point calculations. Both mixtures containing one divalent metal (Cs_2_Ag_*x*_Bi_*x*_ *B*_*y*_Br_6_) and those containing two divalent metals (Cs_2_Ag_*x*_Bi_*x*_ *B*_*y*_ *B'*_*z*_Br_6_) are identified solely by their divalent-metal concentration(s). The black filled symbols denote the relative energies of the selected representative structures.

We found that compounds with the same *N*_P_ can have different *N*_G_, showing that even with an equal total number of generated structures, changes in the chemical nature of *B* and *B′*, such as a larger principal quantum number *n* for *B′* than for *B*, alter the topology and organization of the energy landscapes. In addition, the differing ratios *N*_P_/*N*_G_ indicate that there is no simple rule to predict how many representative configurations are needed to accurately model a given alloy of the form Cs_2_Ag_*x*_Bi_*x*_ *B*_*y*_ *B'*_*z*_Br_6_ (within the assumptions and energetic thresholds of our model).

Based on the Spearman correlation coefficient (*r*_*s*_), mixtures with two divalent metals show a nonlinear monotonic increase in |{*n*_*k*_}| with increasing total relative energy (*r*_*s*_ > 0.55). Identifying the mixture only by its metal composition, this correlation is weak for Ag_0.75_Bi_0.75_ *B*_0.50_ (*r*_*s*_ = 0.32) and Ag_0.50_Bi_0.50_ *B* (*r*_*s*_ = 0.29). A high correlation indicates that more structures are associated with representative structures at higher relative energies, as observed for Ag_0.50_Bi_0.50_ *B*_0.50_ *B'*_0.50_ (*r*_*s*_ = 0.83), Ag_0.50_Bi_0.50_ *B*_0.75_ *B'*_0.25_ (*r*_*s*_ = 0.85), and Ag_0.25_Bi_0.25_ *B*_1.25_ *B'*_0.25_ (*r*_*s*_ = 0.84). Compared with relative energy distributions, mixtures with two divalent metals have |{*n*_*k*_}| ≤ 5 for *k* = 1, 2, 3, whereas mixtures with one divalent metal have |{*n*_*k*_}| ≤ 24 for the same *k*. Thus, mixtures with one divalent metal have many structures near the lowest-energy configuration, while mixtures with two divalent metals have only a few.

### 3.2 Trends in Optimized Structures via Stress Tensor Calculations

To map the energy landscape of the selected double perovskites, we calculated the relative total energies, Δ*E*_tot_^*i*^ = *E*_tot_^*i*^ – *E*_tot_^lowest^, for all structural configurations from the stress-tensor calculations. As shown in Figure 4, the relative energies lie within a very narrow range: Δ*E*_tot_^*i*^ never exceeded 44 meV/atom. This is notably smaller than the typical metastability range in multicomponent alloys, which can reach 70 meV/atom while still permitting experimental synthesis in multiple phases.^47^

**Figure 4:**
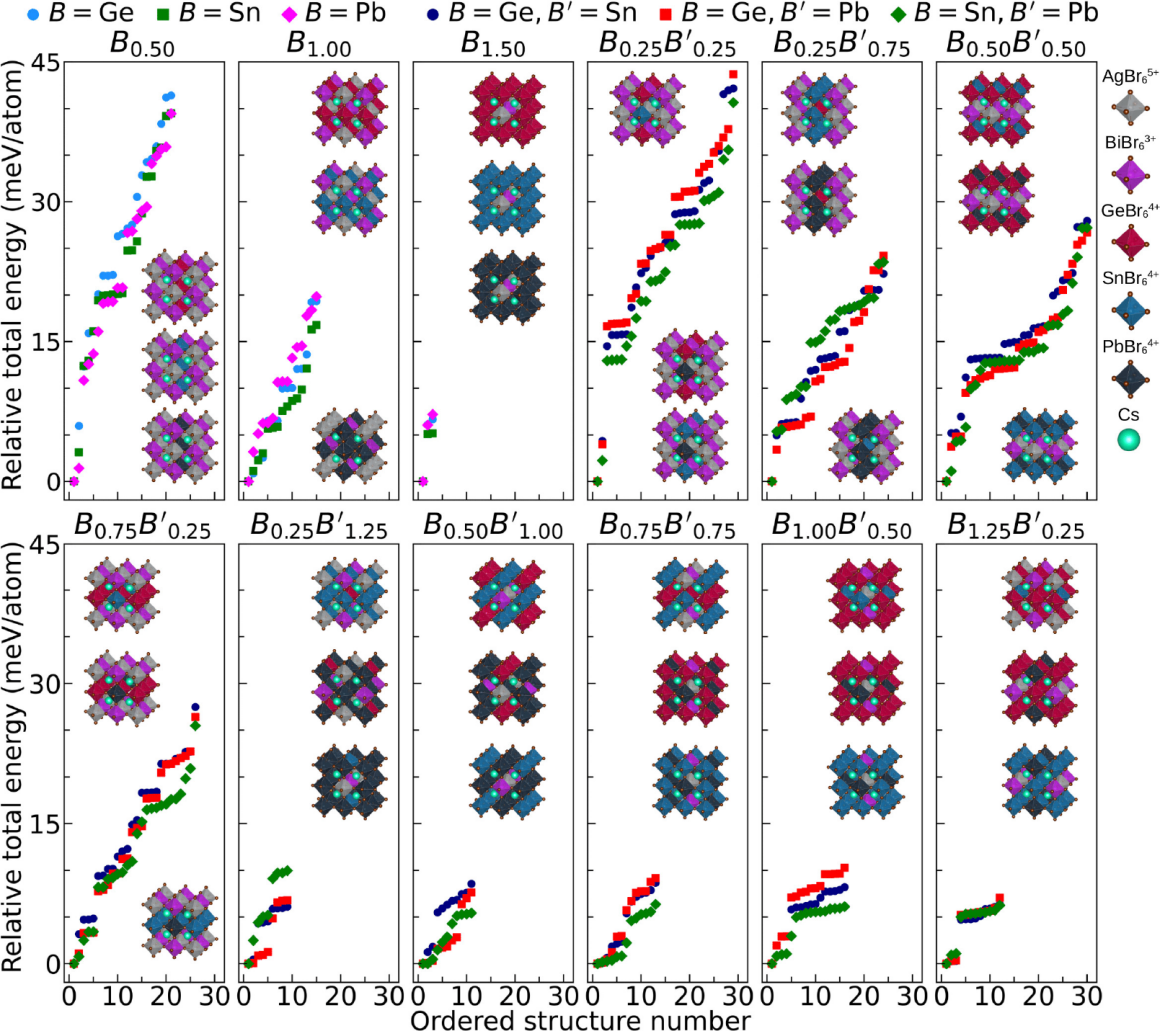
Relative total energies with respect to the lowest-energy structure, Δ*E*_tot_^*i*^ = *E*_tot_^*i*^ – *E*_tot_^lowest^, for all representative structures obtained from stress-tensor calculations. Mixtures containing either one divalent metal (Cs_2_Ag_*x*_Bi_*x*_ *B*_*y*_Br_6_) or two divalent metals (Cs_2_Ag_*x*_Bi_*x*_ *B*_*y*_ *B'*_*z*_Br_6_) are identified solely by their divalent-metal concentration(s). The lowest-energy structural configurations are shown as insets within each graph, and the different metal-halide octahedra are identified on the right side of the figure.

In our optimizations, all structures were constrained to cubic symmetry, so energy variations arose only from different periodic atomic arrangements, not phase transitions. The narrow energy spread implies that many configurations are energetically accessible within one phase, indicating strong configurational flexibility. Thermodynamically, this near degeneracy may allow entropy-driven stabilization of disordered or partially ordered states at finite temperatures. In both systems containing one divalent metal (*B*_*y*_) or two (*B*_*y*_ *B'*_*z*_), three distinct groups emerged, defined by the fraction of perovskite octahedral sites occupied by Ag and Bi:

1. 75% occupancy: corresponding to *B*_0.50_ or *B*_0.25_ *B'*_0.25_.
2. 50% occupancy: corresponding to *B*_1.00_ or *B*_0.25_ *B'*_0.75_, *B*_0.50_ *B'*_0.50_, and *B*_0.75_ *B'*_0.50_.
3. 25% occupancy: corresponding to *B*_1.50_ or *B*_0.25_ *B'*_1.25_, *B*_0.50_ *B'*_1.00_, *B*_0.75_ *B'*_0.75_, *B*_1.00_ *B'*_0.50_, and *B*_1.25_ *B'*_0.25_.

As Figure 4 shows, reducing the content of Ag and Bi narrows the energy range of structural configurations. Each configuration represents a group of energetically equivalent structures; therefore, when they lie within a narrower interval closer to the lowest-energy configuration, the probability of forming a polycrystalline solid increases at lower concentrations of Ag and Bi. Considering halide complexes such as AgBr_6_^5–^, BiBr_6_^3–^, GeBr_6_^4–^, SnBr_6_^4–^, and PbBr_6_^4–^ in the perovskite array alters the energetic landscape, yielding different degrees of structural stability and complexity. The stability of these mixed systems may be governed by the strain of size mismatch and by electronic interactions that modulate the bonding preferences.

For mixtures with one divalent metal, the relative energy distributions in all Ag/Bi compositions showed an approximately linear trend (*R* ^2^ ≥ 0.8, see Supporting Information), with the smallest slope for *B* = Sn, indicating higher intrinsic stability between the structures. This behavior could be attributed to the reduced lattice strain arising from the size match between Ag^+^ and Sn^2+^ (*r*_Ag_ = *r*_Sn_ = 1.15Å).^48,49^

In mixtures with two divalent metals, we obtain three different average (or effective) ionic radii: *r*_av_^Ge/Sn^ = 0.94 Å, *r*_av_^Ge/Pb^ = 0.96 Å, and *r*_av_^Sn/Pb^ = 1.17 Å.^48,49^ This increases structural complexity by enhancing strain effects due to ionic size disparities. However, the difference in ionic radii is small (*r*_av_^Sn/Pb^ – *r*_Ag_ = 0.02 Å). Consequently, analysis of relative energy dispersions showed that in more than half of the studied mixtures the smallest slope occurs for *B* = Sn and *B′* = Pb, indicating a higher relative stability likely arising from minimal structural strain.

In cases where such relative stability or linear behavior is not evident, other factors become dominant, including cases where more than 50% of the metal species share the same ionic radius or where complex orbital interactions arise from electronic repulsion. Additionally, certain cation combinations may favor specific ordering motifs (e.g., rock-salt or layered structures) that minimize the total energy.^50^

We find distinct equilibrium volumes (*V*_0_) for each structure and mixture. The lowest-energy configuration does not always have the smallest *V*_0_, which shows that expansion of the volume of the unit cell does not systematically stabilize the system. This decoupling of volume and energy minima implies that stress-induced unit-cell distortions can create spatial variations in local density with little energetic cost. Because all of the solids studied lie in a narrow energy range, laboratory synthesis of such mixtures will likely generate additional macroscopic internal stress from coexisting regions with different equilibrium volumes and the resulting intrinsic density inhomogeneity.

### 3.3 Equilibrium Lattice Parameters

First, all relative errors obtained for the lattice constants (*a*_0_) of the unit cells of the pristine materials were below 1.5%. In addition, they all agreed very well with the tendency of the ionic radii of the metal cations: *r*_Pb_ = 1.19Å (*a*_0_ = 11.86 Å for CsPbBr_3_) > *r*_Sn_ = 1.15Å (*a*_0_ = 11.69 Å for CsSnBr_3_) > *r*_av_^Ag/Bi^ = 1.09 Å (*a*_0_ = 11.37 Å for Cs_2_AgBiBr_6_) > *r*_Ge_ = 0.73 Å (*a*_0_ = 11.35 Å for CsGeBr_3_). As observed in Figure 5, the average equilibrium lattice constants (⟨*a*_0_⟩) of the representative structures were indeed regulated by the metal composition based on this tendency, having the smallest values in compounds with a large amount of Ge and gradually increasing as the amount of Ag and Bi, Sn or Pb increases.

**Figure 5:**
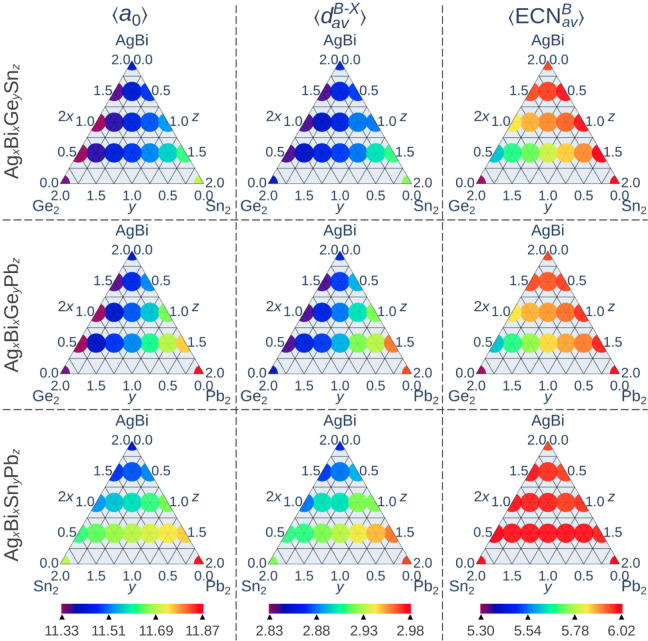
Average structural properties (columns) for each mixture type, identified solely by their metal composition (rows). Based on the chemical formula Cs_2_Ag_*x*_Bi_*x*_ *B*_*y*_ *B'*_*z*_Br_6_ and the discussion in Section 2.2, the quantity 2*x* is used in each ternary plot, as the AgBi concentration is the variable component within the mixtures. From left to right: the average equilibrium lattice constant, ⟨*a*_0_⟩ = ∑_*j*_^*N*_*G*_^ *a*_0_^*j*^/*N*_*G*_; the average metal-halide bond, ⟨*d*_av_^*B*–*X*^⟩ = ∑_*j*_^*N*_*G*_^(*d*_av_^*B*–*X*^)_*j*_/*N*_*G*_; and the average effective coordination number for metals at the *B* perovskite site, ⟨ECN_av_^*B*^⟩ = ∑_*j*_^*N*_*G*_^(ECN_av_^*B*^)_*j*_/*N*_*G*_. In all cases, *j* denotes the *j*th representative structure, and both *d*_av_^*B*–*X*^ and ECN_av_^*B*^ correspond to the average values calculated for that structure.

Furthermore, the mean lengths of the metal-halide bonds (⟨*d*_av_^*B*–*X*^⟩) were consistent with the variation of ⟨*a*_0_⟩, indicating that ⟨*d*_av_^*B*–*X*^⟩ indeed governed the dimensions of cubic unit cells. This result is entirely expected, as the different unit-cell’s volumes depend directly on the volume of each octahedron, which, in turn, is determined by the metal-halide bond lengths. Moreover, since averaged values were considered, steric effects emerging from the different spatial arrangements of atoms within the structures for mixtures with the same compositions were effectively smoothed out, leading to the elucidation of the clear correlation between bond lengths and lattice parameters.

### 3.4 Average *B*–*X* Distances and Effective Coordination Numbers

It has been well-known that the GeBr_6_^4–^ octahedra within the perovskite structure tend to exhibit significant distortion, characterized by three *short* Ge–Br bond lengths (*d*_*s*_^Ge–Br^) and three *long* ones (*d*_*l*_^Ge–Br^).^45,51^ To provide an idea of this difference, for the pristine CsGeBr_3_ in the present work, we obtained *d*_*l*_^Ge–Br^ – *d*_*s*_^Ge–Br^ ≈ 0.5 Å, which is consistent with the literature.^51^ To gain insights into this octahedral distortion, a modified version of the weighted effective coordination number (ECN_*w*_) approach of Da Silva^43,52^ was employed, defined by

$${\mathrm{ECN}}_{w}^{i}=\sum_{j}\exp[1-(d^{ij}/2r_{w}^{i}{)}^{6}];$$ 8

$$r_{w}^{i}=\frac{\sum_{j}d^{ij}\exp[1-(d^{ij}/r_{w}^{ij}{)}^{6}]}{2\sum_{j}\exp[1-(d^{ij}/r_{w}^{ij}{)}^{6}]}.$$ 9

In eqs 8 and 9, *d* ^*ij*^ is the distance between atoms *i* and *j*, *r*_*w*_^*i*^ is the weighted atomic radius of atom *i*, and *r*_*w*_^*ij*^ is the sum of the weighted atomic radii of atoms *i* and *j*. *r*_*w*_^*i*^ is obtained self-consistently, using as a first guess half of the nearest-neighbor distance.

In an ideal undistorted corner-sharing octahedron, ECN_*w*_^*B*^ = 6.00 NNN (where NNN denotes the number of nearest neighbors), indicating that orbital interactions of the *B*-site cation are governed solely by *B*–*X* bonds. In pristine CsGeBr_3_, the counterbalance between *d*_*l*_^Ge–Br^ and *d*_*s*_^Ge–Br^ leads to a reduced value of ECN_av_^Ge^ = 5.30 NNN, reflecting a pronounced octahedral distortion. By contrast, the minimal dispersion of the length of the bonds in *d* ^Sn–Br^ and *d* ^Pb–Br^ yielded nearly ideal values of ECN_av_^Sn^ = ECN_av_^Pb^ = 6.01 NNN for CsSnBr_3_ and CsPbB_3_, respectively (Figure 5). For Cs_2_AgBiBr_6_, the lowest-energy configuration, consistent with the experimentally reported structure,^13^ showed ECN_av_^*B*^ = 6.01 NNN and an overall average of ⟨ECN_av_^*B*^⟩ = 5.97 NNN, indicating that despite the volume differences between the AgBr_6_^5–^ and BiBr_6_^3–^ octahedra, they remained largely undistorted.

These results support the applicability of the empirical octahedral parameter *μ* = *r*_*B*_/*r*_*X*_ (0.44 ≤ *μ* ≤ 0.90),^53^ that can correlate smaller values *μ* with stronger octahedral distortions. In particular, Ge is the only cation outside of this distortion-tolerant range (*μ* = 0.37), while all other metals fall within the perovskite criterion. This is clearly reflected in the alloys Ag_*x*_Bi_*x*_Sn_*y*_Pb_*z*_, where, on average, the Jahn–Teller effects were not significant across the octahedral networks.^45^

Consequently, deviation from ideal halide octahedra increases with higher Ge content, reaching maximum distortion in CsGeBr_3_. According to eq 8, octahedral distortions are inferred from *B*–Br bond lengths. When ⟨ECN_*w*_^*B*^⟩ < 6 NNN, the local coordination environment departs from an ideal Platonic octahedron, leading to possible off-centering of the *B*-site cation and lone-pair-driven distortions, well-known in germanium halides and referred to as Jahn–Teller distortions.^45,54^ These distortions are mitigated by larger cations such as Ag, Bi, Sn, or Pb. Considering the average ionic radii at the *B*-site, these mixtures satisfy the Bartel tolerance factor criterion τ < 4.18 for perovskite stability.^38^ All studied compositions lie within this limit (τ ≤ 4.15), except CsGeBr_3_ (τ = 4.41), consistent with its strong structural distortion.

### 3.5 Energetic Stability Characterization via Mixing and Cohesive Energies

The diagrams provided in Figure 6 map three key properties throughout the compositional space of the mixtures: the excess energy (*E*_exc_^min^), calculated by eq 10, the cohesive energy (*E*_coh_^min^) and the energy of the cumulative Integrated Crystal Orbital Hamilton Population,^55,56^ CICOHP (*E*_CICOHP_^min^), all obtained exclusively for the structure with the lowest total energy among the set of representative configurations in each composition.

$$E_{\mathrm{exc}}=\frac{1}{2}\left(E_{\mathrm{tot}}^{{\mathrm{Cs}}_{2}{\mathrm{Ag}}_{x}{\mathrm{Bi}}_{x}B_{y}B_{z}^{\prime }{\mathrm{Br}}_{6}}-xE_{\mathrm{tot}}^{{\mathrm{Cs}}_{2}\mathrm{AgBiB}r_{6}}-\frac{y}{2}E_{\mathrm{tot}}^{{\mathrm{Cs}}_{2}B_{2}{\mathrm{Br}}_{6}}-\frac{z}{2}E_{\mathrm{tot}}^{{\mathrm{Cs}}_{2}B_{2}^{\prime }{\mathrm{Br}}_{6}}\right)$$ 10

In eq 10, the factor $\frac{1}{2}$ ensures that the resulting energies are directly comparable to those of simple perovskites. Using Cs as the reference cation in the double-perovskite Cs_2_AgBiBr_6_, all calculated *E*_exc_^min^ values are divided by two, consistent with the stoichiometry of simple perovskites such as Cs*B*Br_3_.

**Figure 6:**
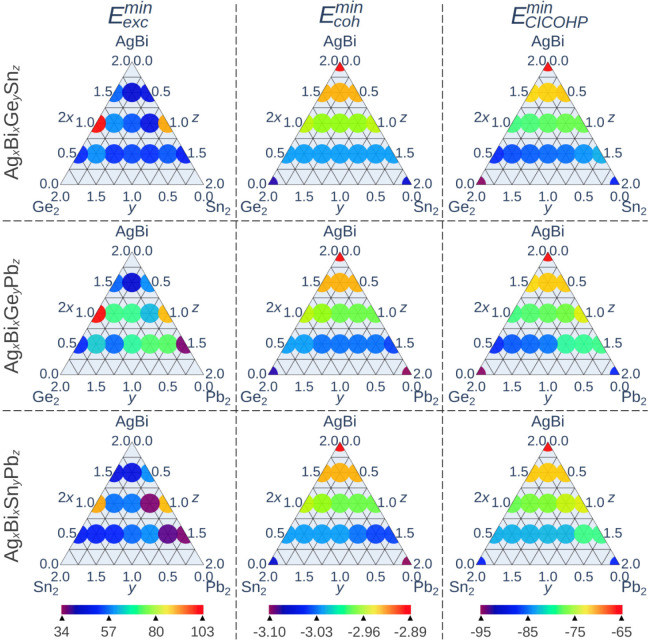
Energy descriptors (columns) for each mixture type, identified solely by their metal composition (rows). Based on the chemical formula Cs_2_Ag_*x*_Bi_*x*_ *B*_*y*_ *B'*_*z*_Br_6_ and the discussion in Section 2.2, the quantity 2*x* is used in each ternary plot, as the AgBi concentration is the variable component within the mixtures. From left to right: minimum excess energy, *E*_exc_^min^; minimum cohesive energy, *E*_coh_^min^; and minimum cumulative ICOHP energy, *E*_CICOHP_^min^. Because only the lowest-energy configuration was considered for each composition, the reported values correspond to the minimum attainable energies, which justifies the use of the superscript ‘min’. The units for *E*_exc_^min^, *E*_coh_^min^, and *E*_CICOHP_^min^ values are meV, eV/atom, and eV, respectively.

The stability of a mixture or alloy is central in materials science and can serve as a descriptor for predicting new materials. It is quantified by the formation enthalpy (*H*_f_) of the solid-state reaction forming the *A*_1–*x*_ *B*_*x*_ alloy, *H*_f_(*x*) = *H* ^*A*_1–*x*_ *B*_*x*_^ – (1 – *x*)*H* ^*A*^ – *xH* ^*B*^,^57^ where the energies are the total energies of the solids from first-principles calculations. This excess energy is also referred to as formation energy^58^ or mixing energy.^59^ A negative *E*_exc_ means exothermic, energetically favorable alloying, with stronger average bonds than in the pure constituents. However, all ternary mixtures show positive excess energies (Figure 6), indicating that, for all studied compositions, mixture formation is endothermic and less favorable than the pure components.

The stability criterion based on excess energy can be expressed using the cohesive energies of the constituent solids, since the total electronic energy of a solid is the sum of atomic and cohesive contributions.^60^ However, as shown in Figure 6, the excess energies do not mirror the cohesive energies. This is because the relative concentrations of the constituents weight their energetic contributions, eq 10, preventing a direct link between cohesive energy and the stability of the mixture. Since no negative values *E*_exc_^min^ were found, the cohesive energy of the most stable configuration in each mixture is never lower than the concentration-weighted cohesive energies of the pristine constituents.

Simple perovskite alloys can form mixed-halide and mixed-metal compounds that are more stable than their pristine counterparts.^58,61^ For the present systems, the most stable compositions (with the lowest positive *E*_exc_^min^) are Ag_0.25_Bi_0.25_Pb_1.50_, Ag_0.50_Bi_0.50_Sn_0.25_Pb_0.75_, and Ag_0.25_Bi_0.25_Sn_0.25_Pb_1.25_. As PSC absorbers, these compounds may preserve their photoactive phase and could help maintain device performance over long periods,^62^ mitigating long-term stability losses. The cohesive energy (*E*_coh_), which measures the energy needed to dissociate a crystal into neutral atoms, quantifies the microscopic bonding interactions governing macroscopic stability; more negative values indicate stronger bonding. As shown in Figure 6, even small amounts of substitutional divalent metal in Cs_2_AgBiBr_6_ strengthen the metal-halide bonds.

Incorporating divalent cations increases the valence electrons available for bonding, strengthening orbital overlap and bond covalency. This led to higher lattice cohesion than in pristine Cs_2_AgBiBr_6_ and improved solid stability. However, none of the mixtures showed a lower *E*_coh_^min^ than the simple perovskites CsGeBr_3_, CsSnBr_3_, or CsPbBr_3_. Thus, the instability inferred from the excess energy is mainly governed by the weighted cohesive energy of the simple perovskites, not the double perovskite. The intrinsic thermodynamic stability of the simple perovskites dictated the energetic balance of the mixtures and, consequently, determined the observed positive *E*_exc_^min^ energetic values.

Based on *E*_coh_^min^, the strongest bonding occurs in CsPbI_3_ (−3.10 eV/atom), consistent with the greater thermodynamic tendency of Sn and Ge to oxidize to Sn­(IV) and Ge­(IV), a behavior absent in lead-based perovskites.^63,64^ On the contrary, the single valence electron of Ag favors more ionic AgBr_6_^5–^ octahedra. Thus, Cs_2_AgBiBr_6_, which has the highest Ag content among the compounds studied, showed the smallest (least negative) *E*_coh_^min^ (−2.89 eV/atom). Accordingly, in all mixtures the cohesive energy becomes less negative toward AgBi-rich compositions.

All computed values of *E*_coh_^min^ lied in a narrow range, from −3.10 up to −2.89 eV/atom, which is comparable to elemental α-Sn (−3.14 eV/atom) and fcc-Ag (−2.95 eV/atom).^65,66^ This indicates that, despite the chemical complexity of multiple oxidation states and differences in electronegativity, electronic compensation between cations stabilizes the lattice. The almost constant cohesive energies across horizontal substitutions (from *B*_2_ to *B'*_2_, Figure 6) support this view, while the decrease toward compositions rich in AgBi reflects the distinct bonding environment introduced by Ag.

### 3.6 Energetic Characterization via Cumulative Integrated Crystal Orbital Hamilton Population

As mentioned above, the *E*_CICOHP_^min^ values were also obtained. The term “cumulative” indicates that the ICOHP values of all individual metal-halide bonds (i.e., Ag–Br, Bi–Br, Ge–Br, Sn–Br, and Pb–Br), considering all valence electrons, were calculated and subsequently summed to produce the total covalent energy associated with the 48 metal-halide bonds per unit cell. ICOHP is a quantum-chemical metric that partitions the energy of the band-structure into contributions from atoms to atoms, thus providing a measure of the bonding strength (covalent interaction) between specific atomic pairs; more negative values of ICOHP correspond to stronger bonding interactions.^55^

Because only off-diagonal COHP contributions were considered, and the analysis is based on Hamiltonian matrix elements derived from the KS wavefunctions obtained in the DFT calculations,^55^ the energy trends of *E*_CICOHP_^min^ closely mirrored those of *E*_coh_^min^. For all species, the covalent contribution in the mixtures was scaled proportionally with their concentration in the crystal lattice. Incorporation of Ag consistently reduced the orbital overlap of the metal-halide bonds, explaining the decrease in cohesive energy observed as the composition approached the purity Cs_2_AgBiBr_6_, which exhibited the lowest value of *E*_CICOHP_^min^.

Consequently, the introduction of divalent metals (Ge, Sn, or Pb) improved covalent interactions, consistent with the cohesive energy analysis. Moreover, on average, the covalent character of the metal-halide bonds in the mixtures was stronger than that of Cs_2_AgBiBr_6_ but weaker than that of CsGeBr_3_, CsSnBr_3_, or CsPbBr_3_, making the mixtures more stable than the double perovskite but less stable than the simple perovskite parent compounds. Consequently, no mixture was more stable than its pristine constituents, as confirmed by the absence of negative *E*_exc_^min^.

The polarization of the bond, evaluated from the differences in electronegativity between the constituent elements (Δχ_*i*–*j*_ = χ_*i*_ – χ_*j*_), yields the following values: χ_Br–Ag_ = 1.01, χ_Br–Sn_ = 0.93, χ_Br–Pb_ = 0.88, χ_Br–Ge_ = 0.79 and χ_Br–Bi_ = 0.71.^67^ On this basis, stronger covalent interactions would be expected for Bi–Br bonds. However, the energy contribution from the overlap of the Bi-6*s* and Bi-6*p* orbitals with the Br-4*p* orbitals was comparable to that of the divalent metals. Moreover, ICOHP analysis showed that for a given metal species at fixed concentration, orbital interactions depended on the chemical environment. These variations were attributed to differences in orbital hybridization, also reflected in the distinct valence-band density-of-state shapes of the Ag-*d*, Br-*p*, Bi-*s*, *B*-*s*, and *B′*-*s* orbitals, which modulated both bonding and antibonding contributions.

Finally, the strongest covalent interactions were found in CsGeI_3_, where the coexistence of long and short Ge–Br bonds arises from the stabilization of the bond. The shorter bonds exhibited a stronger covalent character than the longer ones, highlighting the structural and electronic heterogeneity of the compound. Notably, the cumulative ICOHP did not fully correlate with the lowest excess energies, indicating that crystal stability cannot be explained solely by stronger orbital interactions but also requires significant electrostatic contributions to lattice stabilization.

### 3.7 Fundamental and Optical Band Gaps

Three types of electronic structure calculations were performed for the lowest-energy configurations of all mixtures. Single-point calculations in equilibrium geometries were used to obtain band structures along the path proposed by Setyawan and Curtarolo^68^ for the cubic Brillouin zone, using two approaches: DFT-PBE+D3 and DFT-PBE+D3+SOC. The latter was used due to the presence of heavy atoms in the mixtures (Bi, Sn, and Pb), whose *p* states contribute significantly to the conduction band. In addition, electronic energies were calculated at the Γ point using the hybrid HSE06 functional to achieve an improved description of short-range exchange effects.

Due to the large number of calculations required to provide a complete description of the mixtures, single-point calculations at the DFT-HSE06+SOC level become impractical. However, to account for both spin-orbit coupling (SOC) and Hartree–Fock (HF) short-range exchange (SRE) effects, the following scissor operator was computed:

$$\chi^{\mathrm{HSE06}}=E_{g}^{\Gamma \mathrm{‐HSE06}}-E_{g}^{\Gamma ‐\mathrm{PBE}+D3}$$ 11

In eq 11, *χ* ^HSE06^ is the scissor operator that quantifies the energy correction attributed to the HF-SRE effects. *E*_g_^Γ‑HSE06^ and *E*_g_^Γ‑PBE+D3^ are the band gaps at the point Γ obtained at the DFT-HSE06 and DFT-PBE+D3 levels, respectively. Therefore, using *χ* ^HSE06^, we have defined the fundamental electronic band gap as follows:

$$E_{g}^{\mathrm{PBE}+D3+\mathrm{SOC}+\chi^{\mathrm{HSE06}}}=E_{g}^{\mathrm{PBE}+D3+\mathrm{SOC}}+\chi^{\mathrm{HSE06}}$$ 12

It is worth emphasizing that the scissor-operator approach has inherent limitations, as it applies a rigid, **k**-independent shift to the conduction band and cannot capture band-edge reshaping. However, previous studies on perovskites have shown that the HSE06 band structure closely matches the PBE+D3 dispersion, differing mainly by an overall energy shift,^69^ unlike the case when SOC effects are considered.^70^ SOC effects were explicitly included in our electronic-structure calculations. Moreover, the scissor-operator correction is evaluated separately for each composition and configuration, as it depends on the electronic structure of each system. Accordingly, absolute band gap values should be interpreted with caution, with emphasis placed on comparative trends rather than quantitative accuracy.

In addition to the fundamental gap, we also considered the optical band gap (*E*_g_^optical^), which is the energy that a photon would need to generate the lowest vertical transition (a direct transition), i.e., preserving the electron’s momentum.^71,72^ In this work, we define the optical band gap, *E*_g_^optical^, as the energy at which the absorption coefficient exceeds 1 cm^–1^. Within the Tauc-plot formalism, the absorption spectrum can be divided into three distinct regions: (i) the band-to-band absorption regime, characterized by α­(ω) > 10 × 10^4^ cm^–1^; (ii) the Urbach tail, where α­(ω) ≈ 1 – 10 × 10^4^ cm^–1^; and (iii) the weak absorption tail (WAT), defined by α­(ω) < 1 cm^–1^.^73^

The WAT is commonly associated with defect- or impurity-related states within the crystal, which are not expected to play a role in the present study. By contrast, the Urbach tail also takes place in crystalline semiconductors, displacing the absorption onset to lower energies.^73^ Considering that optically allowed transitions exhibit transition probabilities significantly larger than those of forbidden transitions, the criterion adopted here for *E*_g_^optical^ ensures that the extracted optical band gap corresponds to allowed electronic transitions of the solid.

On this basis, α­(ω) was calculated using the standard definition of solid state optics,^74^ via the frequency-dependent dielectric matrix calculated based on the longitudinal formalism developed by Gajdoš et al.^71^ Finally, due to the cubic symmetry in each unit cell, α­(ω) in each mixture was obtained through: α­(ω) = 1/3 · (α_*xx*_(ω) + α_*yy*_(ω) + α_*zz*_(ω)).

In addition, to quantify the energy contributions of both the effects of SOC and HF-SRE on *E*_g_^optical^, we define the scissor operator *χ* as follows:

$$\chi =E_{g}^{\mathrm{PBE}+D3+\mathrm{SOC}+\chi^{\mathrm{HSE06}}}-E_{g}^{\mathrm{PBE}+D3}$$ 13

and used it to obtain the corrected optical band gap, *E*_g_^optical + *χ*^, by

$$E_{g}^{\mathrm{optical}+\chi }=E_{g}^{\mathrm{optical}}+\chi$$ 14

All results for the fundamental and optical band gaps are shown in Figure 7. Within the level of theory used in this work, the compound Cs_2_AgBiBr_6_ exhibited the most reliable description of the band gap. Our calculations yielded a direct band gap equal to 2.11 eV, compared with 2.45 eV reported by Savory et al.^75^ using DFT-HSE06+SOC, and the experimental interval 2.28–2.62 eV.^8^ In contrast, consistent with previous DFT-PBE results for metal-bromine perovskites,^76–78^ our calculations underestimated the experimental band gap by approximately 1 eV.^8,79^ Although partial compensation arises from HF-SRE effects when SOC ones are included, the well-known band gap underestimation inherent to DFT persisted also in this study.^70^ Consequently, we focused on analyzing band gap variations on the different mixtures, the nature of the electronic transitions, and the comparison between the fundamental and optical band gaps.

**Figure 7:**
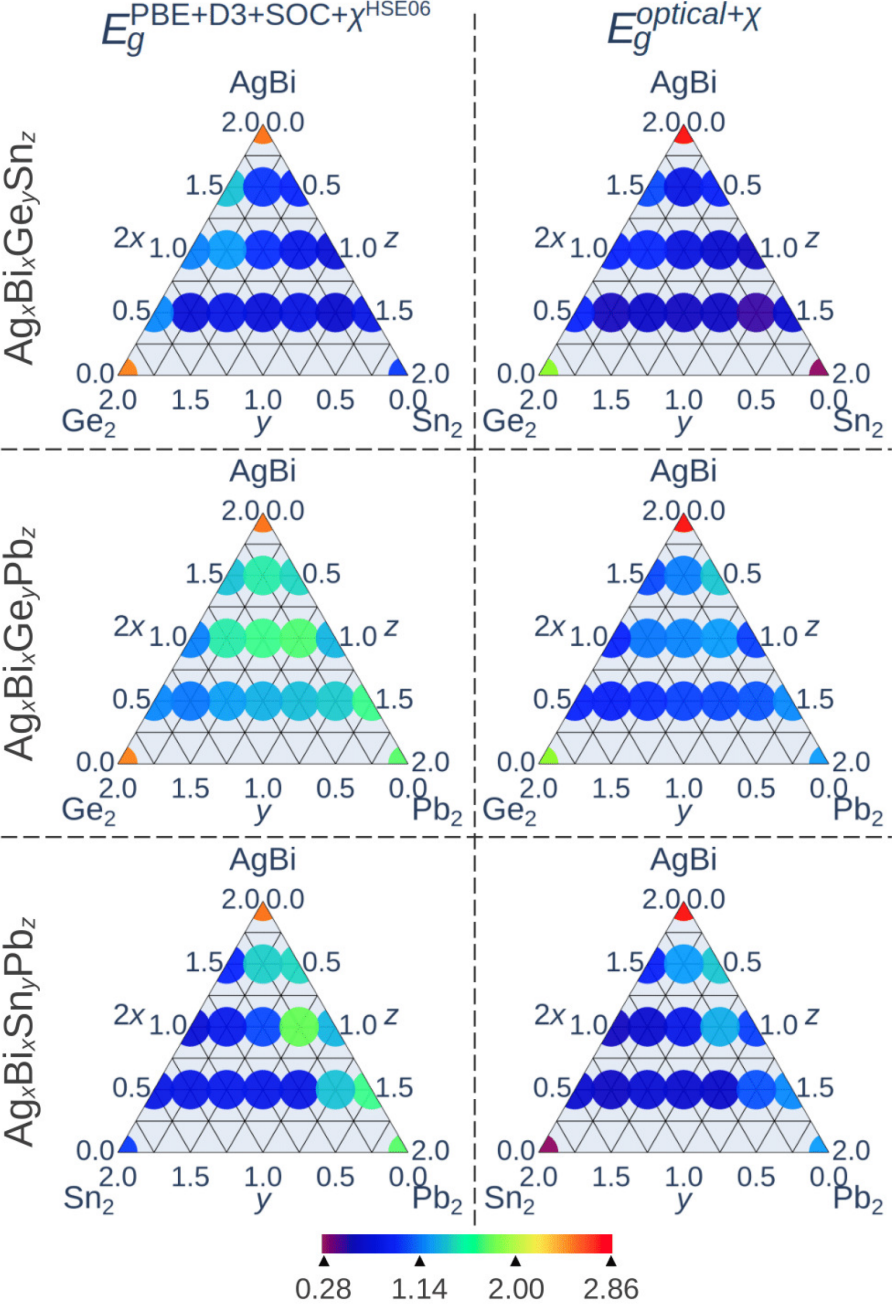
Left: Fundamental electronic band gaps calculated at the PBE+D3+SOC+*χ* ^HSE06^ level. Right: Optical band gap obtained through PBE+D3 including the energy correction from spin-orbit coupling and correlation effects through *χ*. Both band gaps are given in eV for each mixture type, identified solely by their metal composition (rows). Based on the chemical formula Cs_2_Ag_*x*_Bi_*x*_ *B*_*y*_ *B'*_*z*_Br_6_ and the discussion in Section 2.2, the quantity 2*x* is used in each ternary plot, as the AgBi concentration is the variable component within the mixtures.

#### 3.7.1 Band Gap Variation and Electronic States Analysis

As seen in Figure 7, even the smallest concentration of a divalent metal (25%) within the double-perovskite crystal reduced both the fundamental and optical band gap by approximately 1.0 eV, compared to the pristine Cs_2_AgBiBr_6_. This result agreed well with experimental literature, where a reduction of 0.2–0.4 eV in the band gap is observed with the inclusion of 15–20% of Sn,^21,24^ a phenomenon that also occurs with the inclusion of Ge, but to a lesser extent.^21^

An intrinsic limitation of Cs_2_AgBiBr_6_ is its wide band gap, which limits the maximum PCE attainable of a single-junction solar cell to approximately 16.8%.^17^ In contrast, the incorporation of both Ge and Pb could narrow the band gap to an optimal range for photovoltaic applications, making Ag_*x*_Bi_*x*_Ge_*y*_Pb_*z*_ mixtures among the most promising candidates. In addition, compounds that exhibited the smallest *E*_exc_^min^ may also offer good photovoltaic performance.

Besides, when two semiconductors are alloyed, the band gap of the resulting material does not typically vary linearly with composition, as would be expected from Vegard’s law. Instead, it often exhibits a “bowing” behavior, which can be described by the quadratic expression: *E*_g_^*A*_1–*x*_ *B*_*x*_^(*x*) = (1 – *x*)*E*_g_^*A*^ + *xE*_g_^*B*^ – *bx*(1 – *x*), where *b* is the bowing parameter and *A* and *B* are the pristine semiconductors that form the alloy *A*_1–*x*_ *B*_*x*_. As we can see in Figure 7, none of the ternary plots followed a trend comparable to ⟨*a*_0_⟩, ⟨*d*_av_^*B*–*X*^⟩, or even to the ⟨*E*_coh_^min^⟩ energies, which means that neither the fundamental band gap nor the optical one followed a linear behavior in any case. Moreover, since along horizontal lines of the ternary plots we have constant concentrations of Ag_*x*_Bi_*x*_, we calculated the *b* for every Ag_*x*_Bi_*x*_ *B*_*y*_ *B*_*z*_ *′* at fixed *x* and found that all *b*≠0 (see Supporting Information file). Therefore, our systems cannot be regarded as fictitious alloy models.

Interestingly, in central regions of the ternary plots, such as in the Ag_0.50_Bi_0.50_ *B*_*y*_ *B'*_*z*_ mixtures, higher band gap values were obtained than those from Vegard-like behavior, which produced a downward parabola, suggesting stronger repulsive interactions between metal and halogen orbitals, which result in a widening of the band gap in these central compositions.^80^

As noted above, even low concentrations of divalent metal substitution significantly reduced the band gap. This reduction arose from the contribution of *s* and *p* states from both *B* and *B′* cations to the valence (VB) and conduction bands (CB). Consequently, while *B*-*p* orbitals, or both *B*-*p* and *B′*-*p* orbitals in systems with two divalent metals, dominated the CB edge, the contribution of Ag-*s* states at the conduction band minimum (CBM) is reduced and shifted to higher energies.

For VB, in mixtures with one divalent metal, the top is mainly composed of states Ag-*d* and *B*-*s*; even at higher concentrations of Bi, *B*-*s* appeared at higher energies. In mixtures with two divalent metals, Ag-*d* states always contributed to VB, regardless of the amount of Ag. The relative contributions of the Bi-*s*, *B*-*s*, and *B′*-*s* states depended on their concentrations.

#### 3.7.2 Band Gap Character and Comparison between Fundamental and Optical Band Gaps

Another intrinsic limitation of Cs_2_AgBiBr_6_ is its indirect band gap, which arises from momentum-mismatched electronic transitions caused by the hybridization of Ag and Bi orbitals at both the valence band maximum (VBM), primarily Ag-4*d* and Bi-6*s* states, and the CBM, dominated by Ag-5*s* and Bi-6*p* states.^81,82^ Although this limitation can be mitigated, as demonstrated by c-Si technology,^83,84^ absorbers with a direct band gap are generally preferred, as they enable research efforts to focus primarily on device engineering, an area that already demands substantial optimization.^18,85^

Defining the fundamental band gap as *E*_g_^f^ ≡ *E*_g_^PBE+SOC+D3+*χ* ^HSE06^^, only four mixtures exhibited an indirect *E*_g_^f^: Ag_0.50_Bi_0.50_Sn, Ag_0.25_Bi_0.25_Sn_1.50_, Ag_0.25_Bi_0.25_Sn_0.50_Pb, and Ag_0.25_Bi_0.25_Sn_0.75_Pb_0.75_. However, in all these cases, the energy difference between *E*_g_^f^ and the lowest band-to-band transition did not exceed 30 meV (see Supporting Information), a value comparable to the direct-indirect energy splitting at the CBM induced by Rashba-Dresselhaus effects.^86^ By contrast, this difference reached approximately 200 meV in Cs_2_AgBiBr_6_. Therefore, the incorporation of substitutional divalent metals into the heterovalent metal sublattice of double perovskites can effectively suppress the indirect nature of their electronic transitions.

In addition, it can be observed from Figure 7 that slight differences are found between *E*_g_^f^ and *E*_g_^opt.^ (defining *E*_g_^opt.^ ≡ *E*_g_^optical + *χ*^) between all the mixtures studied. Inequality *E*_g_^opt.^ > *E*_g_^f^ is indicative of an indirect band gap semiconductor. However, for almost all systems (35 of 40), the inequality *E*_g_^opt.^ < *E*_g_^f^ was obtained, with a difference not greater than 50 meV between *E*_g_^f^ and *E*_g_^opt.^. It should be noted that *E*_g_^f^ was located at the first peak of the absorption coefficient spectrum. This small shift may be influenced by the presence of an Urbach tail in the absorption coefficient, which may lead to a displacement of the first absorption peak toward higher energies.^73^ In fact, *E*_g_^opt.^ has previously been defined as the energy associated with the first peak of the absorption coefficient spectrum.^87^

However, for CsSnBr_3_, which is well-known to have a direct band gap,^79^ we obtained *E*_g_^f^ – *E*_g_^opt.^ = 0.5 eV, with *E*_g_^f^ still located at the first peak of the absorption coefficient. This energy difference may suggest the need for a higher level of theory for an accurate description of excited states, such as time-dependent DFT (TD-DFT),^88^ which is beyond the scope of the present work.

In addition, in four of the mixtures studied, inequality *E*_g_^opt.^ > *E*_g_^f^ was obtained. The metal compositions of these mixtures were AgBi, Ag_0.75_Bi_0.75_Sn_0.50_, Ag_0.75_Bi_0.75_Pb_0.50_, and Ag_0.75_Bi_0.75_Sn_0.25_Pb_0.25_; their band structures and absorption coefficients are shown in Figure 8. As can be observed, the band at the bottom of the CB at the R point, which belongs to Bi-*p* states,^75,89^ was affected by both SOC and HF-SRE effects, thereby contributing to the shift of the band gap character from indirect (Γ → R) to direct (Γ → Γ).

**Figure 8:**
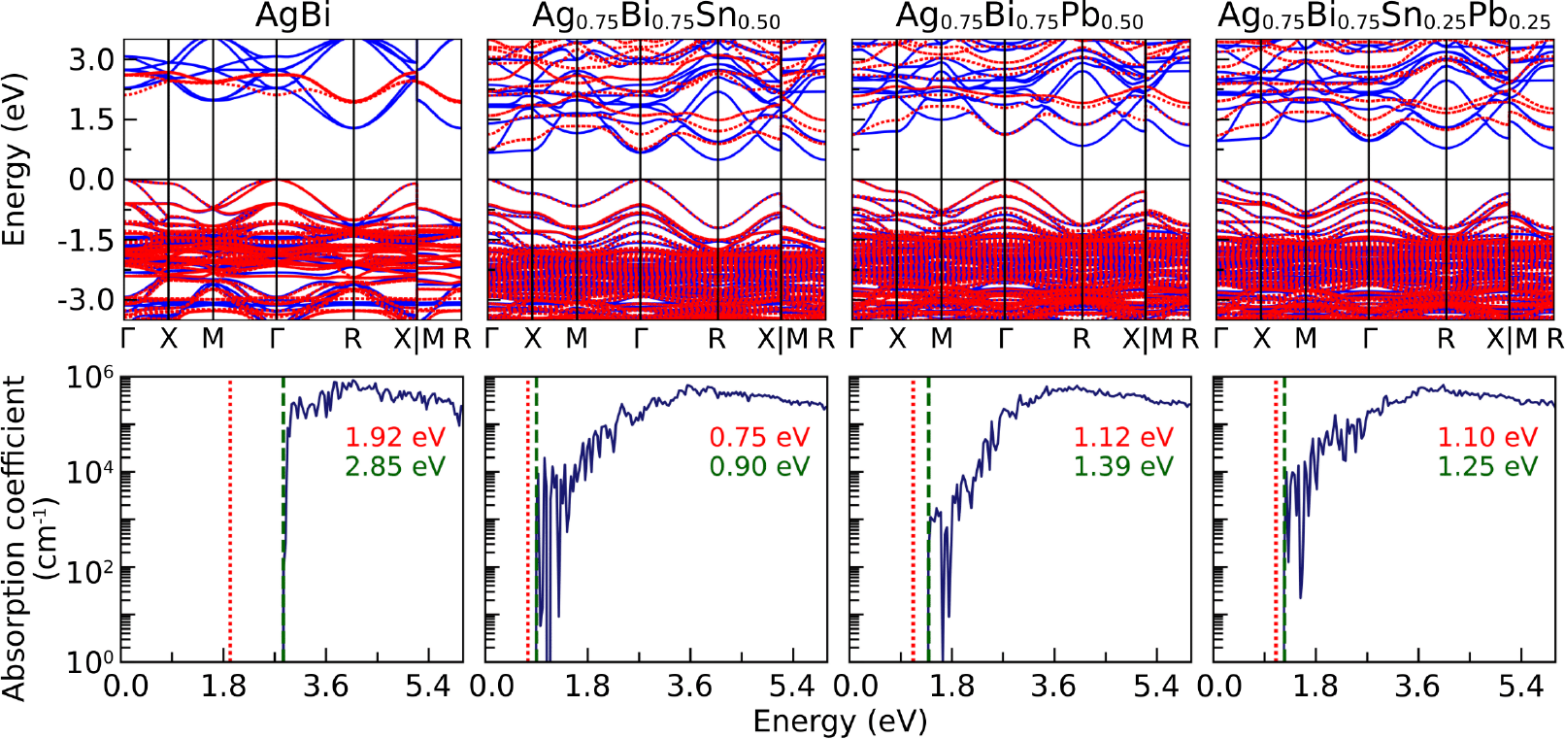
Band structures (upper panels) and absorption coefficients (lower panels) of the mixtures, identified solely by their metal composition, for which the condition *E*_g_^opt.^ > *E*_g_^f^ was satisfied. Electronic dispersions in solid blue and dotted red were obtained at the PBE + D3 and PBE + D3 + SOC + *χ* ^HSE06^ levels, respectively. All absorption coefficient curves were shifted by *χ*. The values of *E*_g_^f^ and *E*_g_^opt.^ are shown as insets in red and green, respectively, and are indicated by vertical lines in the corresponding colors.

Using Cs_2_AgBiBr_6_ as reference, where *E*_g_^f^ = 1.92 eV (Γ → R), the condition *E*_g_^opt.^ > *E*_g_^f^ was attributed to its indirect band gap. This behavior arose because, despite the shift of Bi-*p* states in R induced by both SOC and HF-SRE effects, the Ag-*s* states remained at higher energies in Γ. In contrast, for the remaining compounds, the presence of the Sn-*p* and Pb-*p* states at the bottom of the CB shifted the electronic states at Γ toward lower energies, an effect mainly driven by SOC, resulting in a direct band gap.

Based on this analysis and considering two additional aspects: (i) in metal-halide perovskites the first allowed transition corresponds to that of its direct electronic band gap,^90^ and (ii) the optical absorption calculated here becomes nonzero at the first allowed vertical transition; the inequality *E*_g_^opt.^ > *E*_g_^f^ emerged as a consequence of the absorption spectrum being rigidly shifted by *χ*, without the explicit inclusion of both SOC and HF-SRE effects.

In general, the photoelectronic properties are enhanced even by the smallest amount of substitutional divalent metal in Cs_2_AgBiBr_6_, due to the presence of *B*-*p* (or *B′*-*p*) states, which significantly reduce the energy difference between *E*_g_^f^ and *E*_g_^opt.^.

## 4 Insights into the Role of Divalent Metal Cation Substitution in Cs_2_AgBiBr_6_

### 4.1 Equilibrium Structures

Stress-tensor calculations for representative structures 637, selected from 11578 based on an energy-accuracy criterion for single-point calculations, showed that double-perovskite mixtures have a narrow energy spread across configurations, indicating a high configurational flexibility. This near-degeneracy allows multiple ordered and disordered arrangements to coexist within a single phase, enabling entropy-driven stabilization at finite temperatures. Stability trends were related to the matching of the ionic size at the perovskite *B*-site. The absence of a clear correlation between the equilibrium volume and energy minima further indicates that internal stress can create local density variations at a low energetic cost. The equilibrium lattice constants of the mixtures scaled with the ionic radii of the cations of the *B*-site, as the lengths of the metal-halide bonds set the dimensions of the unit cell. The compounds based on Sn and Pb showed nearly ideal octahedral coordination, while the systems based on Ge, with their smaller ionic radius, exhibited strong distortions and a reduced effective coordination number, in line with the perovskite octahedral criterion. Introducing larger cations such as Ag, Bi, Sn, or Pb reduced these distortions.

### 4.2 Relative Energetic Stability

All complex mixtures showed positive excess energies, indicating endothermic formation and lower energetic favorability than the pure compounds. Thus, none of the compounds stabilized beyond the level implied by the weighted cohesive energies of the pristine materials. However, perovskites with metal compositions Ag_0.25_Bi_0.25_Pb_1.50_, Ag_0.50_Bi_0.50_Sn_0.25_Pb_0.75_, and Ag_0.25_Bi_0.25_Sn_0.25_Pb_1.25_ were the most intrinsically stable in the set. Substitutional divalent metals strengthened metal-halide bonds relative to Cs_2_AgBiBr_6_, which had the highest cohesive energy (i.e., least negative value) due to weaker bonding associated to its high Ag content. In all mixtures, higher AgBi content correlated with higher cohesive energy, but the small variation (≃0.2 eV/atom) indicates that electronic compensation helped stabilize the mixtures despite differences in oxidation states and electronegativities.

### 4.3 Integrated Crystal Orbital Hamilton Populations

This analysis of metal-halide bonds clarified their covalent interactions. The total covalent contributions increased with the concentration of divalent species, while a higher Ag content reduced the orbital overlap and cohesive energy. ICOHP trends closely followed the cohesive energies, underscoring the dominant role of covalent bonding in lattice stability. The strongest covalent interactions occurred in CsGeI_3_, where shorter Ge–Br bonds showed a particularly strong covalent character.

### 4.4 Fundamental and Optical Band Gap

A key result is the systematic reduction of the wide band gap of Cs_2_AgBiBr_6_ through substitution with divalent metals, directly addressing its main limitation as a photovoltaic absorber. Incorporating Ge, Sn, or Pb not only narrows the gap, but also weakens its indirect character and shifts the band edges even at low concentrations, underscoring the high sensitivity of the Ag/Bi framework to the substitution of divalent-metals and progressively aligning the electronic structure with simple metal-halide perovskites. The bowing parameters indicate a strongly nonlinear composition-gap relationship, with the largest deviations at intermediate stoichiometries due to enhanced orbital repulsion and the complex coupling between chemical substitution and electronic structure. The difference between fundamental and optical band gaps remains small; where present, it mainly stems from spin-orbit coupling and hybrid-functional corrections rather than intrinsic electronic rearrangements. Thus, optical transitions stay close to the fundamental gap for most compositions, so band gap engineering directly improves optical absorption.

## 5 Conclusions

This work performed high-throughput DFT calculations to systematically study the structural, energetic, and electronic effects of the divalent-metal mixing in double perovskites based on Cs_2_AgBiBr_6_. The broad configurational space revealed strong structural flexibility, enabling entropy-driven stabilization at finite temperatures despite positive excess energies.

Structural trends followed the ionic size of the *B* perovskite site: larger divalent metals reduce octahedral distortions and satisfy perovskite stability criteria, while smaller cations cause local distortions. All mixed compositions were thermodynamically less stable than their pristine end members, but Sn- and Pb-rich mixtures are the most favorable in the explored set. The cohesive-energy and ICOHP analyses showed that the strength of the bonding reflects a balance of covalent and electrostatic interactions, with higher Ag content systematically weakening the metal-halide bonds.

Substitutional divalent metals consistently narrowed the wide band gap of Cs_2_AgBiBr_6_ and reduced its indirect character, bringing the electronic structure closer to that of simple metal-halide perovskites. The nonlinear dependence of the band gap on composition highlighted the strong sensitivity of the electronic structure to chemistry, while the small fundamental-optical gap difference ensured that band gap tuning directly improved optical absorption.

## Supplementary Material



## Notes

Data employed for the Figures, as well as complementary analyses and additional technical details (PDF)

## Notes

As mentioned, all DFT calculations were done using the Vienna Ab initio Simulation Package (VASP), which can be used under a nonfree academic license. Additional details can be obtained from the link, https://www.vasp.at/. Furthermore, additional details are provided within the electronic Supporting Information, while additional crude data can be obtained directly from the authors upon request. ESI is available free, in the online version at http://www.rsc.org/.

## Abbreviations

PV: photovoltaics
DFT: density functional theory
PBE: Perdew–Burke–Ernzerhof
VASP: Vienna ab initio simulation package
PSC: perovskite solar cell
PCE: power conversion efficiency
KS: Kohn−Sham
SCF: self-consistent field
PAW: Projector Augmented-Wave
SOC: spin-orbit coupling
HF: Hartree−Fock
SRE: short-range exchange
COHP: crystal orbital Hamilton population
