# Supplementary material for: Theoretical Exploration of the Physical-Chemical Properties of Divalent (np 2) Cation Mixing in Double Cs2AgBiBr6 Perovskite
Source: ACS Omega. 2026 Mar 11;11(11):17779–95. doi: 10.1021/acsomega.5c12243 (PMC13019227; doi:10.1021/acsomega.5c12243)
Supplement: Supplementary file 1 [file ao5c12243_si_001.pdf]

Electronic Supporting Information File: Theoretical Exploration of the Physical-Chemical Properties of Divalent (np^2) Cation Mixing in Double $Cs_2AgBiBr_6$ Perovskite

Iván Ornelas-Cruz,[*,†] Ramiro M. dos Santos,[*,†] Matheus P. Lima,[*,‡] and Juarez L. F. Da Silva[*,†]

†*São Carlos Institute of Chemistry, University of São Paulo, Av. Trabalhador São-Carlense* 400, 13560-970, *São Carlos, São Paulo, Brazil*

‡*Department of Physics, Federal University of São Carlos,* 13565-905, *São Carlos, São Paulo, Brazil*

E-mail: iornelas.ipn@gmail.com; ramiromarcelo2@gmail.com; mplima@df.ufscar.br; juarez_dasilva@iqsc.usp.br

Contents

S-1 Introduction **S-3**

S-2 Additional Details on the Theoretical Approach **S-3**

S-2.1 Selected PAW Projectors S-3

S-3 Generation of the Atomic Structure Configurations **S-6**

S-4 Screening of Structure Configurations via Single-Point Total Energy Calculations **S-7**

S-5 Investigation of the Role of Blurring Halogen Atomic Positions for Geometric Optimizations S-21

S-6 Optimization of the Perovskite Mixture Structures via Stress Tensor Optimizations S-22

S-6.1 Comment on **k**-points Sampling of the Brillouin Zone S-22

S-6.2 Computational Convergence of Stress Tensor Optimizations S-24

S-6.3 Strategies to Speed up Stress Tensor Calculations S-24

S-6.4 Total Energy, Lattice Parameter, and Volume of all Optimized Structures at Equilibrium S-30

S-6.5 Pristine Double-Perovskite S-67

S-6.6 Mixtures with One Divalent Metal S-67

S-6.7 Mixtures with Two Divalent Metals S-72

S-7 Convergence of the k-mesh for Density of States and COHP calcluations S-85

S-8 Additional Data on the Physical-Chemical Properties S-93

S-8.1 Energy of Atomic Species S-93

S-8.2 Energy Analysis of the Lowest Structures S-93

S-8.2.1 Further Energy Analysis of the Lowest Structures: Cumulative ICOHP S-94

S-8.3 Electronic and Optical Bandgaps S-98

S-8.4 Bowing Parameter of Fundamental and Optical Band Gap S-102

S-8.5 Band structures at PBE+D3, PBE+D3+SOC, and PBE+D3+SOC+χ^{HSE06} levels S-104

S-8.5.1 Pristine Compounds S-104

S-8.5.2 Mixtures with One Divalent Metal S-105

S-8.5.3 Mixtures with Two Divalent Metals S-108

S-8.6 Density of States at PBE+D3 level S-118

S-8.6.1 Pristine Compounds S-118

S-8.6.2 Resume for Mixtures with One and Two Divalent Metals S-122

S-8.6.3 Mixtures with One Divalent Metal S-123

S-8.6.4 Mixtures with Two Divalent Metals S-132
S-8.7 Absorption Coefficient and Optical Band Gap S-159
S-8.7.1 Pristine Compounds S-159
S-8.7.2 Mixtures with One Divalent Metal S-162
S-8.7.3 Mixtures with Two Divalent Metals S-171
References **S-187**

S-1 INTRODUCTION

This project examined how cation alloying modifies the physico-chemical properties of perovskites (ABX_3) using density functional theory (DFT).[1,2] We chose the double perovskite $Cs_2AgBiBr_6$ as the reference system[3,4] and considered substitutional cations such as Ge^{2+}, Sn^{2+}, and Pb^{2+} to tune its properties. The exploratory nature of this work required numerous DFT calculations to support our conclusions, but space limitations prevented us from including all results in the manuscript. This document compiles additional theoretical details, figure data, and complementary analyses. Upon publication, all raw calculations will be deposited in public repositories.

S-2 ADDITIONAL DETAILS ON THE THEORETICAL APPROACH

S-2.1 Selected PAW Projectors

Table S-1 lists the projectors used for all compounds in this work. Projectors with the highest precision, yielding results comparable to all-electron calculations,[5] were used, except for Ag.

Table S-1. Technical details of the PAW projectors used in this work. From left to right we have: (*i*) chemical species (CS); (*ii*) projector name or label; (*iii*) recommended cutoff energy, identified as `ENMAX`; (*iv*) number of valence electrons considered by each projector (Z_{val}); and, valence electron configuration (VEC) of each projector.

CS	PAW projector	`ENMAX` (eV)	Z_{val}	VEC
Ag	PAW_PBE Ag_GW 06Mar2008	249.844	11	$4d^{10}5s^1$
Bi	PAW_PBE Bi_d_GW 14Apr2014	261.876	17	$5s^25d^{10}6s^26p^3$
Br	PAW_PBE Br_GW 20Mar2012	216.285	7	$5s^25p^5$
Cs	PAW_PBE Cs_sv_GW 23Mar2010	198.101	9	$5s^25p^65d^1$
Ge	PAW_PBE Ge_d_GW 19Mar2013	375.434	14	$3d^{10}4s^24p^2$
Sn	PAW_PBE Sn_d_GW 15Mar2013	260.066	14	$4d^{10}5s^25p^2$
Pb	PAW_PBE Pb_d_GW 14Apr2014	237.809	16	$5s^25d^{10}6s^26p^2$

Although the Ag_GW projector guarantees 1 meV precision, we compared it with the Ag_sv_GW projector (with `ENMAX` = 354.430 eV and VEC = $4s^24p^64d^{11}$) using the primitive unit cell of $Cs_2AgBiBr_6$.[4] The main results are reported in Tables S-2 and S-3.

Table S-2. Total energy and bad gap at the gamma point (E_{tot} and E_g^{Γ}, respectively), of the primitive unit-cell of $Cs_2AgBiBr_6$ for different number of reciprocal integration points within the first Brillouin zone at equilibrium volume and for both projectors, Ag_GW and Ag_sv_GW. $N_{\mathbf{k}}$ is the number of **k**-points constituting the grid, and $N_{\mathbf{k}}^{\text{IBZ}}$ is the number of irreducible **k**-points. The relative energies, $\Delta E_{tot}^{proj.} = E_{tot}^{\text{Ag\_GW}} - E_{tot}^{\text{Ag\_sv\_GW}}$ and $\Delta E_{tot}^{\Gamma\text{-}proj.} = E_{tot}^{\Gamma\text{-Ag\_GW}} - E_{tot}^{\Gamma\text{-Ag\_sv\_GW}}$, are also shown. All values are given in eV.

$N_{\mathbf{k}}$	$N_{\mathbf{k}}^{\text{IBZ}}$	Ag_GW		Ag_sv_GW		$\Delta E_{tot}^{proj.}$	$\Delta E_g^{\Gamma\text{-}proj.}$
		E_{tot}	E_g^{Γ}	E_{tot}	E_g^{Γ}		
8	3	−35.487 083	2.85	−33.412 446	2.89	−2.075	−0.04
64	8	−35.678 988	2.86	−33.598 889	2.86	−2.080	0.00
216	16	−35.680 224	2.91	−33.600 275	2.91	−2.080	0.00
512	29	−35.680 213	2.86	−33.600 227	2.92	−2.080	−0.06

Table S-3. Lattice parameter of the primitive and face-centered cubic cells ($a_0^{\text{pc-fcc}}$ and a_0^{fcc}, respectively) of $Cs_2AgBiBr_6$ for different number of reciprocal integration points within the first Brillouin zone at equilibrium volume and for both projectors, Ag_GW and Ag_sv_GW. $N_{\mathbf{k}}$ is the number of **k**-points constituting the grid, and $N_{\mathbf{k}}^{\text{IBZ}}$ is the number of irreducible **k**-points. The relative structural parameters, $\Delta a_0^x = a_0^x - a_0^{\text{Exp.}}$ with x = pc-fcc or fcc, are also shown. All values are given in Å.

$N_{\mathbf{k}}$	$N_{\mathbf{k}}^{\text{IBZ}}$	Ag_GW				Ag_sv_GW			
		$a_0^{\text{pc-fcc}}$	$\Delta a_0^{\text{pc-fcc}}$	a_0^{fcc}	Δa_0^{fcc}	$a_0^{\text{pc-fcc}}$	$\Delta a_0^{\text{pc-fcc}}$	a_0^{fcc}	Δa_0^{fcc}
8	3	8.06	0.09	11.40	0.13	8.05	0.08	11.39	0.12
64	8	8.04	0.07	11.37	0.10	8.05	0.08	11.38	0.11
216	16	8.04	0.07	11.37	0.10	8.04	0.07	11.37	0.10
512	29	8.05	0.08	11.38	0.11	8.04	0.07	11.37	0.10
Exp.[4]		7.97		11.27		7.97		11.27	

S-3 GENERATION OF THE ATOMIC STRUCTURE CONFIGURATIONS

```
Ag2Bi2GeSn3_597
   1.00000000000000
    11.2699999999999996    0.0000000000000000    0.0000000000000000
     0.0000000000000000   11.2699999999999996    0.0000000000000000
     0.0000000000000000    0.0000000000000000   11.2699999999999996
   Sn    Bi    Bi    Sn    Ag    Sn    Ge    Ag    Br    Cs
     1     1     1     1     1     1     1     1    24     8
Direct
  0.0000000000000000  0.0000000000000000  0.0000000000000000
  0.5000000000000000  0.0000000000000000  0.0000000000000000
  0.0000000000000000  0.5000000000000000  0.0000000000000000
  0.0000000000000000  0.0000000000000000  0.5000000000000000
  0.5000000000000000  0.5000000000000000  0.0000000000000000
  0.5000000000000000  0.0000000000000000  0.5000000000000000
  0.0000000000000000  0.5000000000000000  0.5000000000000000
  0.5000000000000000  0.5000000000000000  0.5000000000000000
  0.2500000000000000  0.0000000000000000  0.0000000000000000
  0.7500000000000000  0.0000000000000000  0.0000000000000000
  0.0000000000000000  0.2500000000000000  0.0000000000000000
  0.0000000000000000  0.7500000000000000  0.0000000000000000
  0.0000000000000000  0.0000000000000000  0.2500000000000000
  0.0000000000000000  0.0000000000000000  0.7500000000000000
  0.2500000000000000  0.5000000000000000  0.0000000000000000
  0.7500000000000000  0.5000000000000000  0.0000000000000000
  0.2500000000000000  0.0000000000000000  0.5000000000000000
  0.7500000000000000  0.0000000000000000  0.5000000000000000
  0.5000000000000000  0.2500000000000000  0.0000000000000000
  0.5000000000000000  0.7500000000000000  0.0000000000000000
  0.0000000000000000  0.2500000000000000  0.5000000000000000
  0.0000000000000000  0.7500000000000000  0.5000000000000000
  0.5000000000000000  0.0000000000000000  0.2500000000000000
  0.5000000000000000  0.0000000000000000  0.7500000000000000
  0.0000000000000000  0.5000000000000000  0.2500000000000000
  0.0000000000000000  0.5000000000000000  0.7500000000000000
  0.2500000000000000  0.5000000000000000  0.5000000000000000
  0.7500000000000000  0.5000000000000000  0.5000000000000000
  0.5000000000000000  0.2500000000000000  0.5000000000000000
  0.5000000000000000  0.7500000000000000  0.5000000000000000
  0.5000000000000000  0.5000000000000000  0.2500000000000000
  0.5000000000000000  0.5000000000000000  0.7500000000000000
  0.2500000000000000  0.2500000000000000  0.2500000000000000
  0.7500000000000000  0.2500000000000000  0.2500000000000000
  0.2500000000000000  0.7500000000000000  0.2500000000000000
  0.7500000000000000  0.7500000000000000  0.2500000000000000
  0.2500000000000000  0.2500000000000000  0.7500000000000000
  0.7500000000000000  0.2500000000000000  0.7500000000000000
  0.2500000000000000  0.7500000000000000  0.7500000000000000
  0.7500000000000000  0.7500000000000000  0.7500000000000000
```

Figure S-1. Example of a POSCAR file, i.e., the input file needed in VASP that contains the lattice geometry and the atomic positions. Each generated model structure, through the permutations of the order of the metal species (line 6), was identified simply by a number, in this particular case it is the structure 597 representing the compound $Cs_2Ag_{0.50}Bi_{0.50}Ge_{0.25}Sn_{0.75}Br_6$.

```
1c1
< Ag2Bi2GeSn3_597
---
> Ag2Bi2GeSn3_832
6c6
<    Sn   Bi   Bi   Sn   Ag   Sn   Ge   Ag   Br   Cs
---
>    Sn   Ge   Bi   Bi   Sn   Ag   Sn   Ag   Br   Cs
```

Figure S-2. Result of applying the line: `diff ./Ag2Bi2GeSn3_597/POSCAR_597 ./Ag2Bi2GeSn3_832/POSCAR_832` in the Bash terminal, i.e., the result of the difference between the input files POSCAR_597 and POSCAR_832 representing the compound $Cs_2Ag_{0.50}Bi_{0.50}Ge_{0.25}Sn_{0.75}Br_6$. As it could be seen the only thing that changes from one POSCAR file to the other is the order of the metal species and the name which contains the structure number.

```
name@user:/path/$ grep TITEL ./Ag2Bi2GeSn3_597/POTCAR_597
   TITEL  = PAW_PBE Sn_d_GW 15Mar2013
   TITEL  = PAW_PBE Bi_d_GW 14Apr2014
   TITEL  = PAW_PBE Bi_d_GW 14Apr2014
   TITEL  = PAW_PBE Sn_d_GW 15Mar2013
   TITEL  = PAW_PBE Ag_GW 06Mar2008
   TITEL  = PAW_PBE Sn_d_GW 15Mar2013
   TITEL  = PAW_PBE Ge_d_GW 19Mar2013
   TITEL  = PAW_PBE Ag_GW 06Mar2008
   TITEL  = PAW_PBE Br_GW 20Mar2012
   TITEL  = PAW_PBE Cs_sv_GW 23Mar2010

name@user:/path/$ grep TITEL ./Ag2Bi2GeSn3_832/POTCAR_832
   TITEL  = PAW_PBE Sn_d_GW 15Mar2013
   TITEL  = PAW_PBE Ge_d_GW 19Mar2013
   TITEL  = PAW_PBE Bi_d_GW 14Apr2014
   TITEL  = PAW_PBE Bi_d_GW 14Apr2014
   TITEL  = PAW_PBE Sn_d_GW 15Mar2013
   TITEL  = PAW_PBE Ag_GW 06Mar2008
   TITEL  = PAW_PBE Sn_d_GW 15Mar2013
   TITEL  = PAW_PBE Ag_GW 06Mar2008
   TITEL  = PAW_PBE Br_GW 20Mar2012
   TITEL  = PAW_PBE Cs_sv_GW 23Mar2010
```

Figure S-3. Result of the search for the occurrence of the word "TITEL" within the files ./Ag2Bi2GeSn3_597/POTCAR_597 and ./Ag2Bi2GeSn3_832/POTCAR_832, to show how the atomic POTCAR files were concatenated based on the order of the species in the POSCAR files of the structures 597 and 832 representing the compound $Cs_2Ag_{0.50}Bi_{0.50}Ge_{0.25}Sn_{0.75}Br_6$.

S-4 SCREENING OF STRUCTURE CONFIGURATIONS VIA SINGLE-POINT TOTAL ENERGY CALCULATIONS

We performed single-point Gamma-point energy calculations on all structures generated by permuting Ag, Bi, Ge, and Sn on the perovskite *B*-site of the compounds in

Table S-4. The Kohn–Sham self-consistent cycles used an energy convergence criterion of 1.0×10^{-4} eV. The plane-wave cut-off was 1.125×$\texttt{ENMAX}_{max}$ with $\texttt{ENMAX}_{max}$ = 375.434 eV (Table S-1), giving a cut-off of 422.363 eV. Table S-4 (column "Analogous compounds") also lists compounds that can be studied using the representative structures obtained for the above alloys (Ag, Bi, Ge, Sn). Representative structures were chosen considering the accuracy of this level of theory; in the best case, our energies are estimated to be 1–3 meV / atom from the exact value.[5]

Table S-4. Number of structures to model the alloys in this work. N_P was obtained through permutations of the metal species in the different B-site positions of the perovskite structure. N_G was obtained after having calculated the total energy of the structures generated through the metal's permutations and having grouped them by the energy criteria of $\Delta E < 0.10$ eV (2.5 meV/atom). All structures belonging to a particular group $\{n_k\}$ fulfilled the energy criteria by means of $\Delta E = E^i_{tot} - E^j_{tot}$, with $i{\neq}j$ and $i, j{\in}\{n_k\}$. For consistency, if $|\{n_k\}| = N_k$, where $k = 1, 2, \ldots, N_G$; thus $N_1 + N_2 + \cdots + N_{N_G} = N_P$. The tolerance factor τ is also shown: those who fulfilled $\tau < 4.18$ are empirically predicted with a perovskite structure.

Compound	τ	N_P	N_G	Analogous compounds	τ
$Cs_8Ag_4Bi_4Br_{24}$	3.96	70	10		
$Cs_8Ag_3Bi_3Ge_2Br_{24}$	3.94	560	21	$Cs_8Ag_3Bi_3Sn_2Br_{24}$	3.97
				$Cs_8Ag_3Bi_3Pb_2Br_{24}$	3.98
$Cs_8Ag_2Bi_2Ge_4Br_{24}$	4.00	420	15	$Cs_8Ag_2Bi_2Sn_4Br_{24}$	3.99
				$Cs_8Ag_2Bi_2Pb_4Br_{24}$	4.02
$Cs_8AgBiGe_6Br_{24}$	4.15	56	3	$Cs_8AgBiSn_6Br_{24}$	4.00
				$Cs_8AgBiPb_6Br_{24}$	4.06
$Cs_8Ag_3Bi_3GeSnBr_{24}$	3.94	1120	29	$Cs_8Ag_3Bi_3GePbBr_{24}$	3.94
				$Cs_8Ag_3Bi_3SnPbBr_{24}$	3.98
$Cs_8Ag_2Bi_2GeSn_3Br_{24}$	3.95	1680	24	$Cs_8Ag_2Bi_2GePb_3Br_{24}$	3.96
				$Cs_8Ag_2Bi_2SnPb_3Br_{24}$	4.02
$Cs_8Ag_2Bi_2Ge_2Sn_2Br_{24}$	3.94	2520	30	$Cs_8Ag_2Bi_2Ge_2Pb_2Br_{24}$	3.94
				$Cs_8Ag_2Bi_2Sn_2Pb_2Br_{24}$	4.00
$Cs_8Ag_2Bi_2Ge_3SnBr_{24}$	3.96	1680	26	$Cs_8Ag_2Bi_2Ge_3PbBr_{24}$	3.95
				$Cs_8Ag_2Bi_2Sn_3PbBr_{24}$	3.99
$Cs_8AgBiGeSn_5Br_{24}$	3.96	336	9	$Cs_8AgBiGePb_5Br_{24}$	3.98
				$Cs_8AgBiSnPb_5Br_{24}$	4.05

Continued on next page

Table S-4 – Continued from previous page

Compound	τ	N_P	N_G	Analogous compounds	τ
$Cs_8AgBiGe_2Sn_4Br_{24}$	3.94	840	11	$Cs_8AgBiGe_2Pb_4Br_{24}$	3.94
				$Cs_8AgBiSn_2Pb_4Br_{24}$	4.03
$Cs_8AgBiGe_3Sn_3Br_{24}$	3.94	1120	13	$Cs_8AgBiGe_3Pb_3Br_{24}$	3.94
				$Cs_8AgBiSn_3Pb_3Br_{24}$	4.03
$Cs_8AgBiGe_4Sn_2Br_{24}$	3.99	840	16	$Cs_8AgBiGe_4Pb_2Br_{24}$	3.98
				$Cs_8AgBiSn_4Pb_2Br_{24}$	4.03
$Cs_8AgBiGe_5SnBr_{24}$	4.06	336	12	$Cs_8AgBiGe_5PbBr_{24}$	4.04
				$Cs_8AgBiSn_5PbBr_{24}$	4.02
Total N_P and N_G		11 578	219		

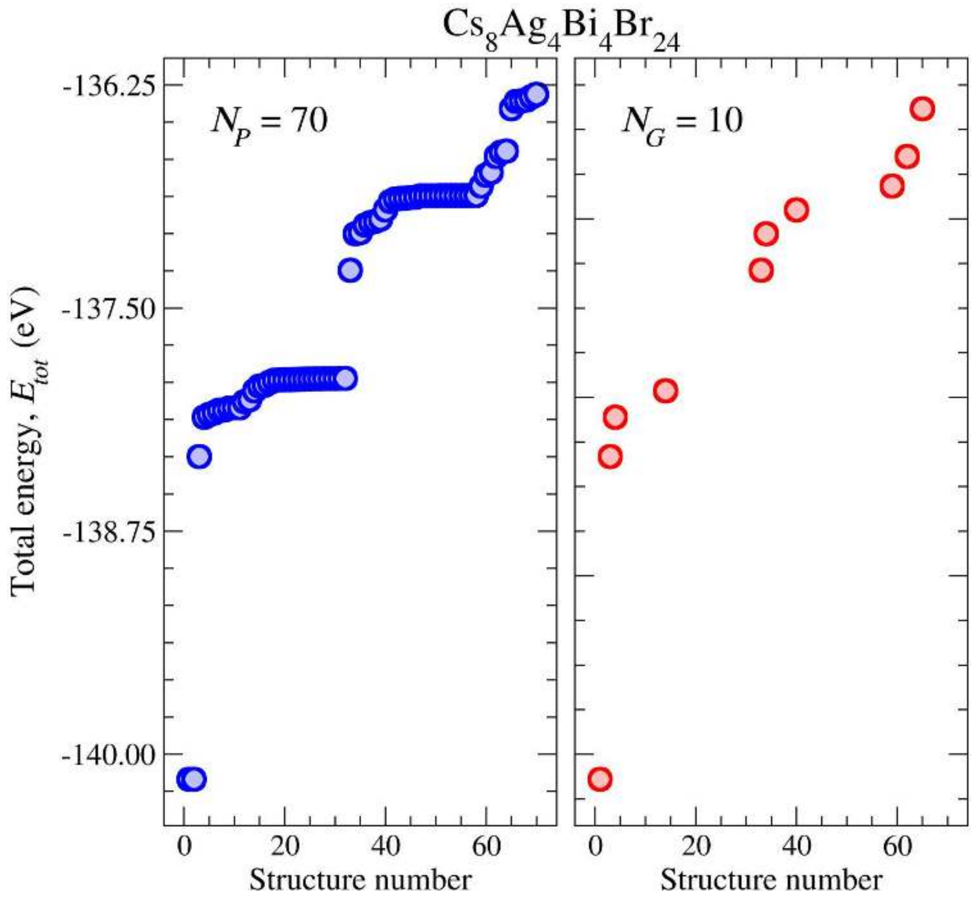

Figure S-4. Total energy as a function of the structure number for $Cs_8Ag_4Bi_4Br_{24}$. Left (blue): energies of all N_P structures generated by permutations. Right (red): energies of the structures with the lowest energy from each group $\{n_k\}$ (the number of groups is given by N_G). The major spacing in y-axis (Δ_y) is $\Delta_y = 1.25$ eV.

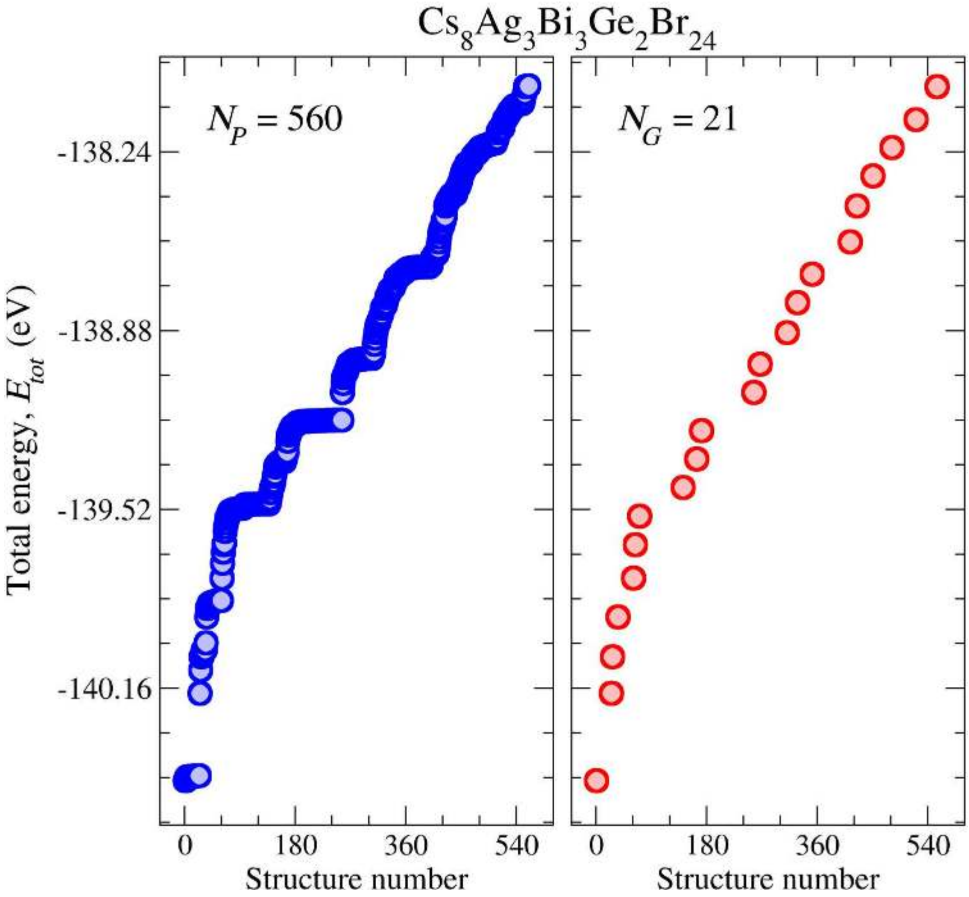

Figure S-5. Total energy as a function of the structure number for $Cs_8Ag_3Bi_3Ge_2Br_{24}$. Left (blue): energies of all N_P structures generated by permutations. Right (red): energies of the structures with the lowest energy from each group $\{n_k\}$ (the number of groups is given by N_G). The major spacing in y-axis (Δ_y) is $\Delta_y = 0.64$ eV.

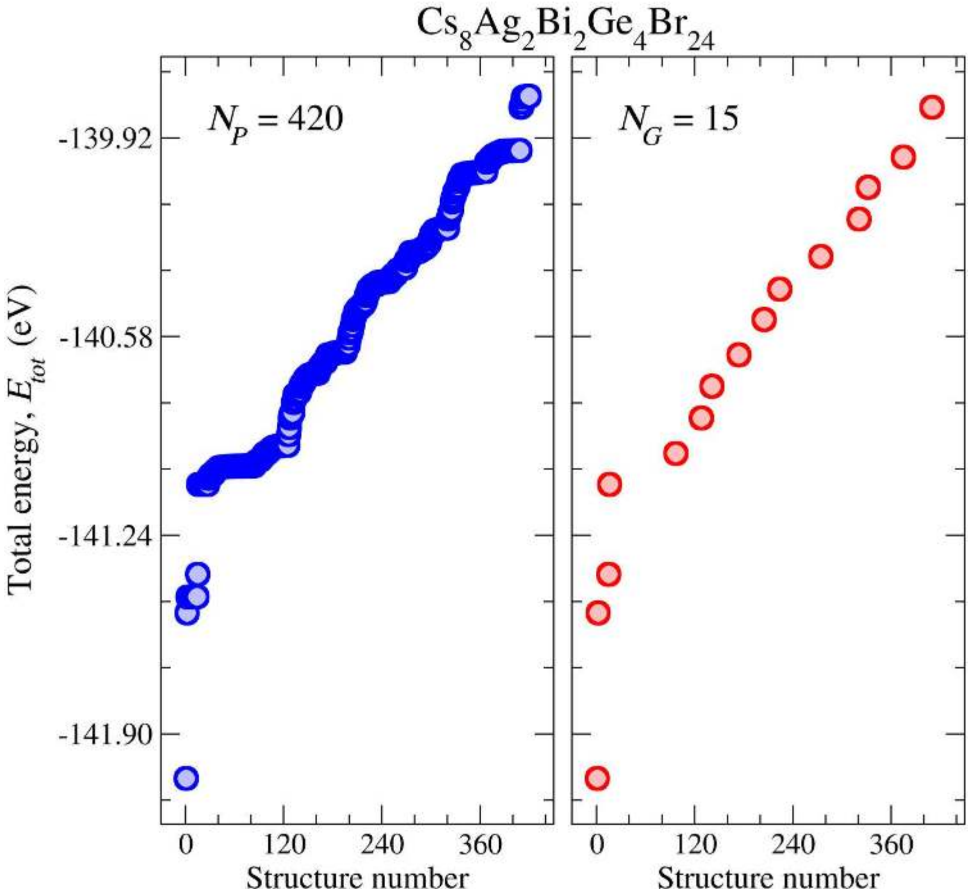

Figure S-6. Total energy as a function of the structure number for $Cs_8Ag_2Bi_2Ge_4Br_{24}$. Left (blue): energies of all N_P structures generated by permutations. Right (red): energies of the structures with the lowest energy from each group $\{n_k\}$ (the number of groups is given by N_G). The major spacing in y-axis (Δ_y) is $\Delta_y = 0.66$ eV.

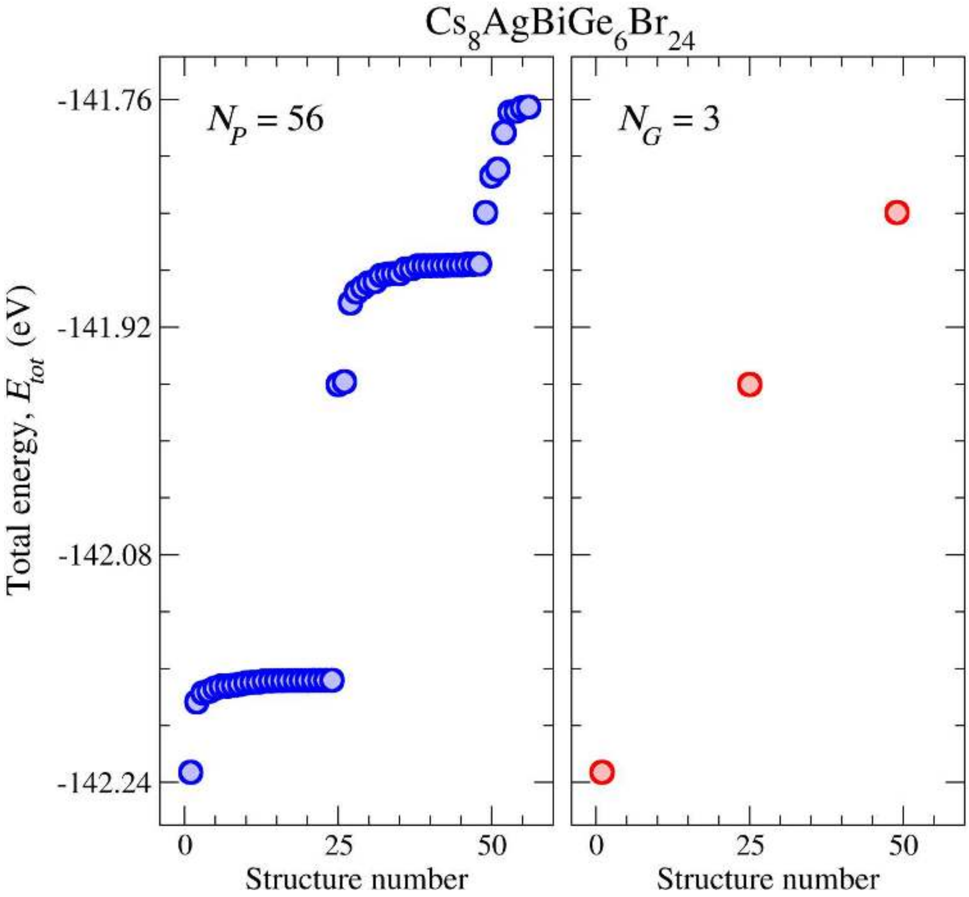

Figure S-7. Total energy as a function of the structure number for $Cs_8AgBiGe_6Br_{24}$. Left (blue): energies of all N_P structures generated by permutations. Right (red): energies of the structures with the lowest energy from each group $\{n_k\}$ (the number of groups is given by N_G). The major spacing in y-axis (Δ_y) is $\Delta_y = 0.16$ eV.

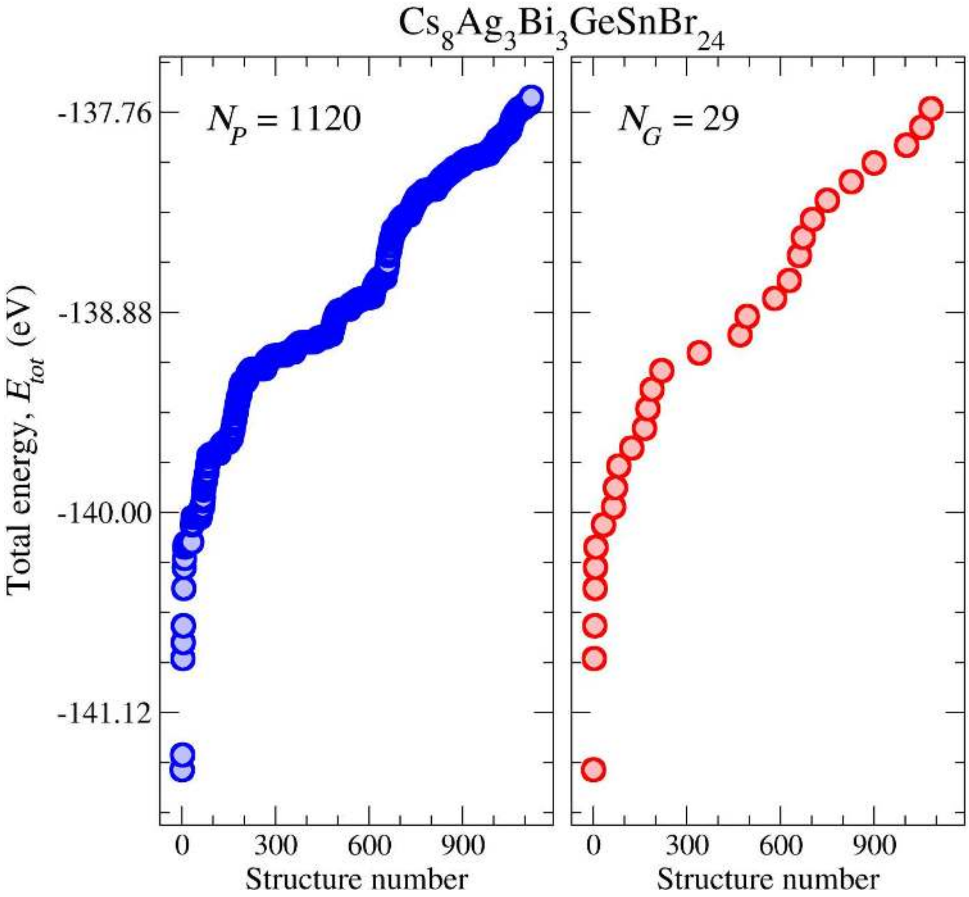

Figure S-8. Total energy as a function of the structure number for $Cs_8Ag_3Bi_3GeSnBr_{24}$. Left (blue): energies of all N_P structures generated by permutations. Right (red): energies of the structures with the lowest energy from each group $\{n_k\}$ (the number of groups is given by N_G). The major spacing in y-axis (Δ_y) is $\Delta_y = 1.12$ eV.

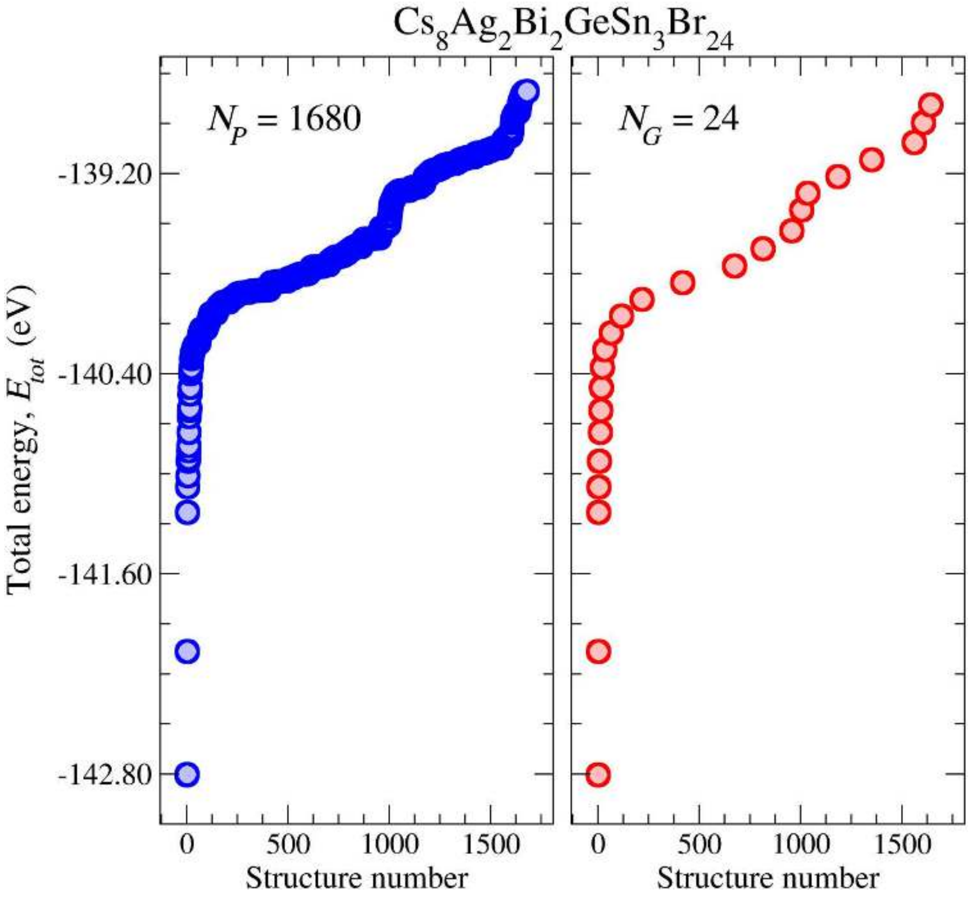

Figure S-9. Total energy as a function of the structure number for $Cs_8Ag_2Bi_2GeSn_3Br_{24}$. Left (blue): energies of all N_P structures generated by permutations. Right (red): energies of the structures with the lowest energy from each group $\{n_k\}$ (the number of groups is given by N_G). The major spacing in y-axis (Δ_y) is Δ_y = 1.20 eV.

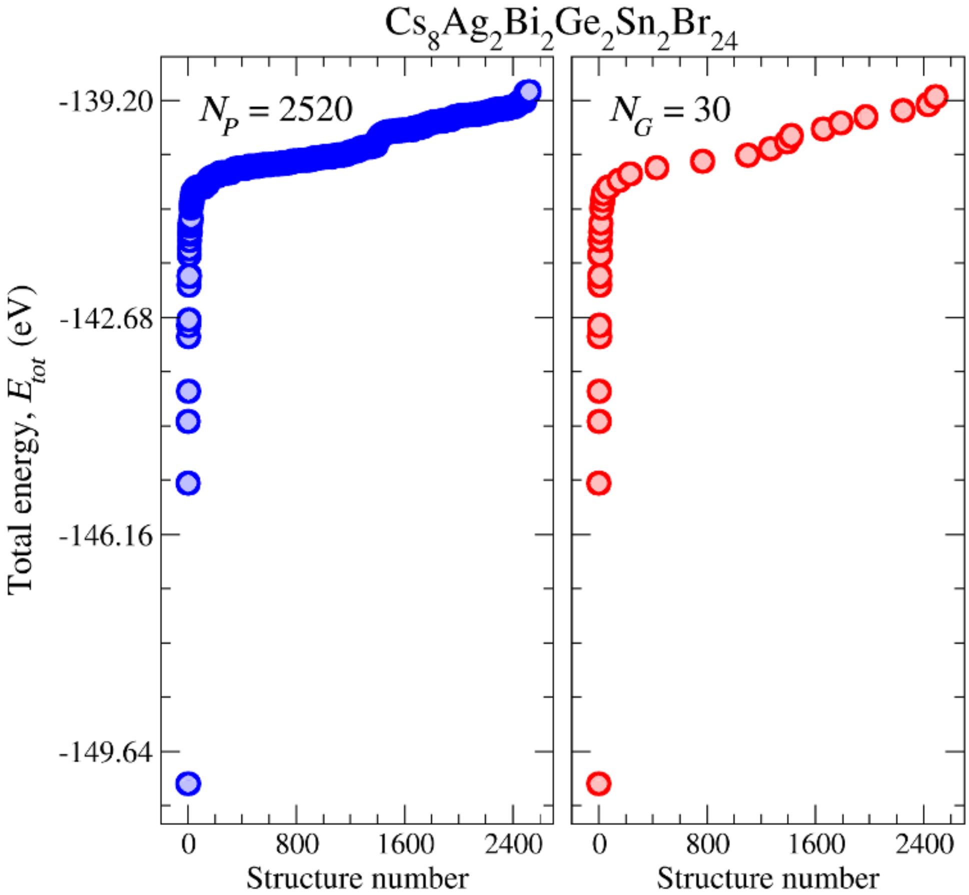

Figure S-10. Total energy as a function of the structure number for $Cs_8Ag_2Bi_2Ge_2Sn_2Br_{24}$. Left (blue): energies of all N_P structures generated by permutations. Right (red): energies of the structures with the lowest energy from each group $\{n_k\}$ (the number of groups is given by N_G). The major spacing in y-axis (Δ_y) is $\Delta_y = 3.48$ eV.

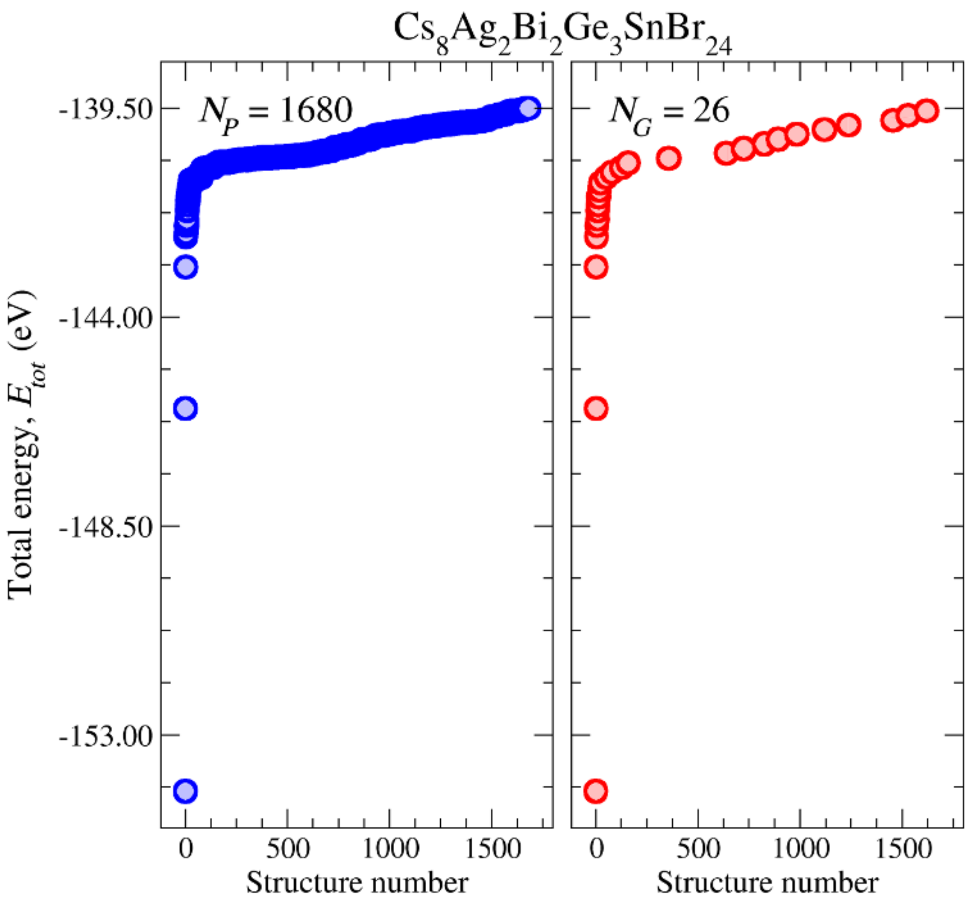

Figure S-11. Total energy as a function of the structure number for $Cs_8Ag_2Bi_2Ge_3SnBr_{24}$. Left (blue): energies of all N_P structures generated by permutations. Right (red): energies of the structures with the lowest energy from each group $\{n_k\}$ (the number of groups is given by N_G). The major spacing in y-axis (Δ_y) is $\Delta_y = 4.50$ eV.

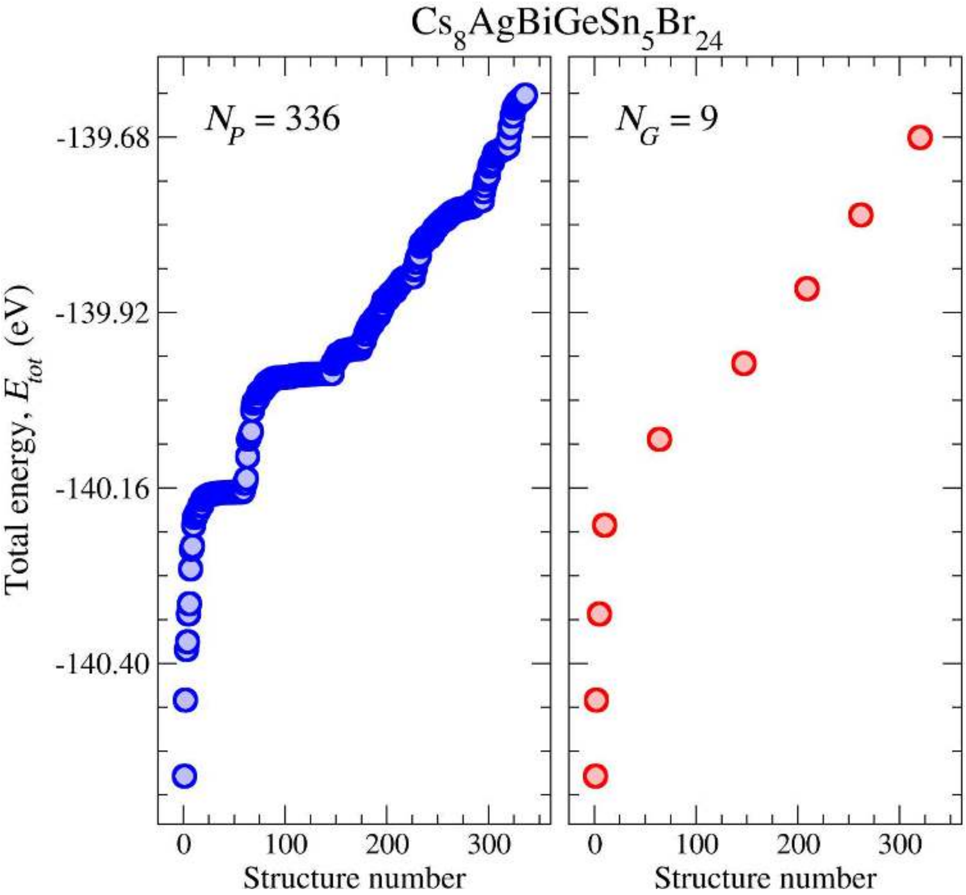

Figure S-12. Total energy as a function of the structure number for $Cs_8AgBiGeSn_5Br_{24}$. Left (blue): energies of all N_P structures generated by permutations. Right (red): energies of the structures with the lowest energy from each group $\{n_k\}$ (the number of groups is given by N_G). The major spacing in y-axis (Δ_y) is $\Delta_y = 0.24$ eV.

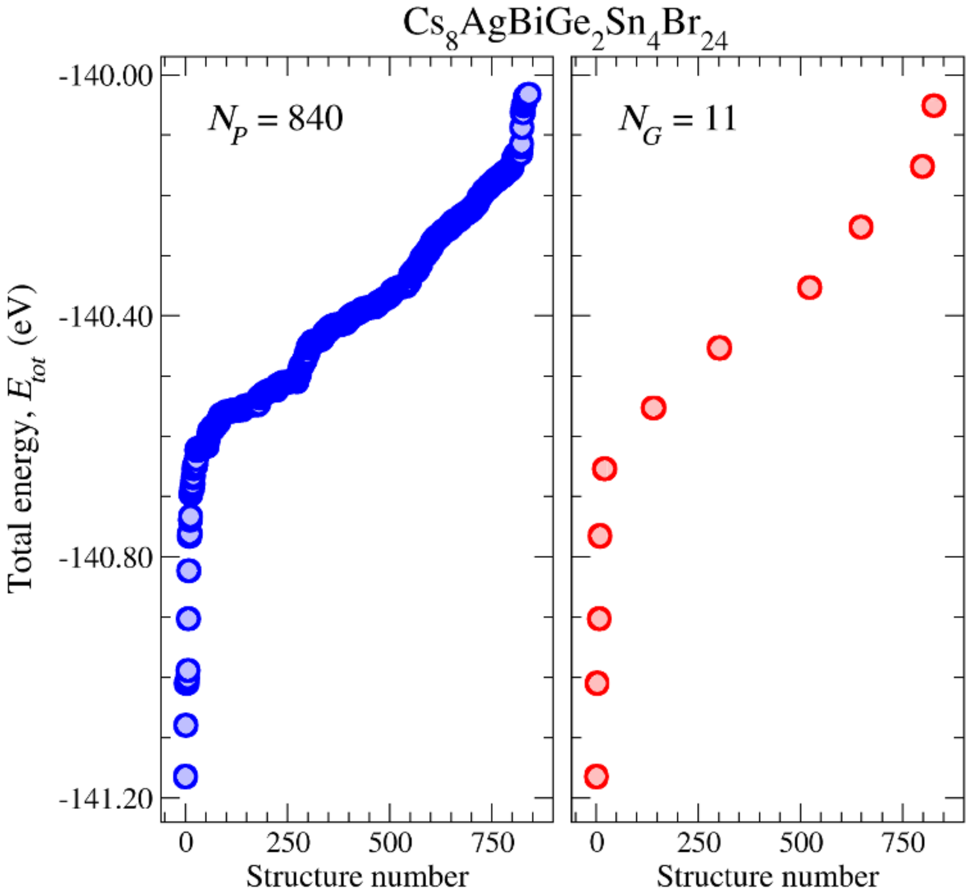

Figure S-13. Total energy as a function of the structure number for $Cs_8AgBiGe_2Sn_4Br_{24}$. Left (blue): energies of all N_P structures generated by permutations. Right (red): energies of the structures with the lowest energy from each group $\{n_k\}$ (the number of groups is given by N_G). The major spacing in y-axis (Δ_y) is $\Delta_y = 0.40$ eV.

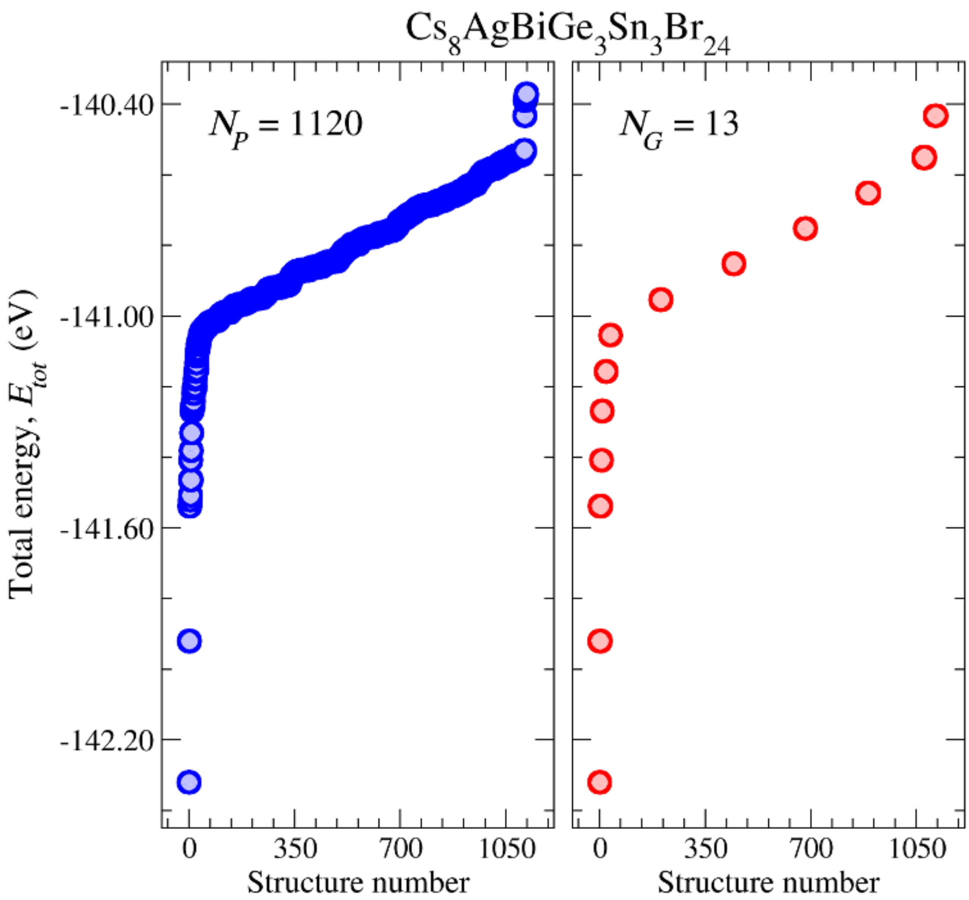

Figure S-14. Total energy as a function of the structure number for $Cs_8AgBiGe_3Sn_3Br_{24}$. Left (blue): energies of all N_P structures generated by permutations. Right (red): energies of the structures with the lowest energy from each group $\{n_k\}$ (the number of groups is given by N_G). The major spacing in y-axis (Δ_y) is $\Delta_y = 0.60$ eV.

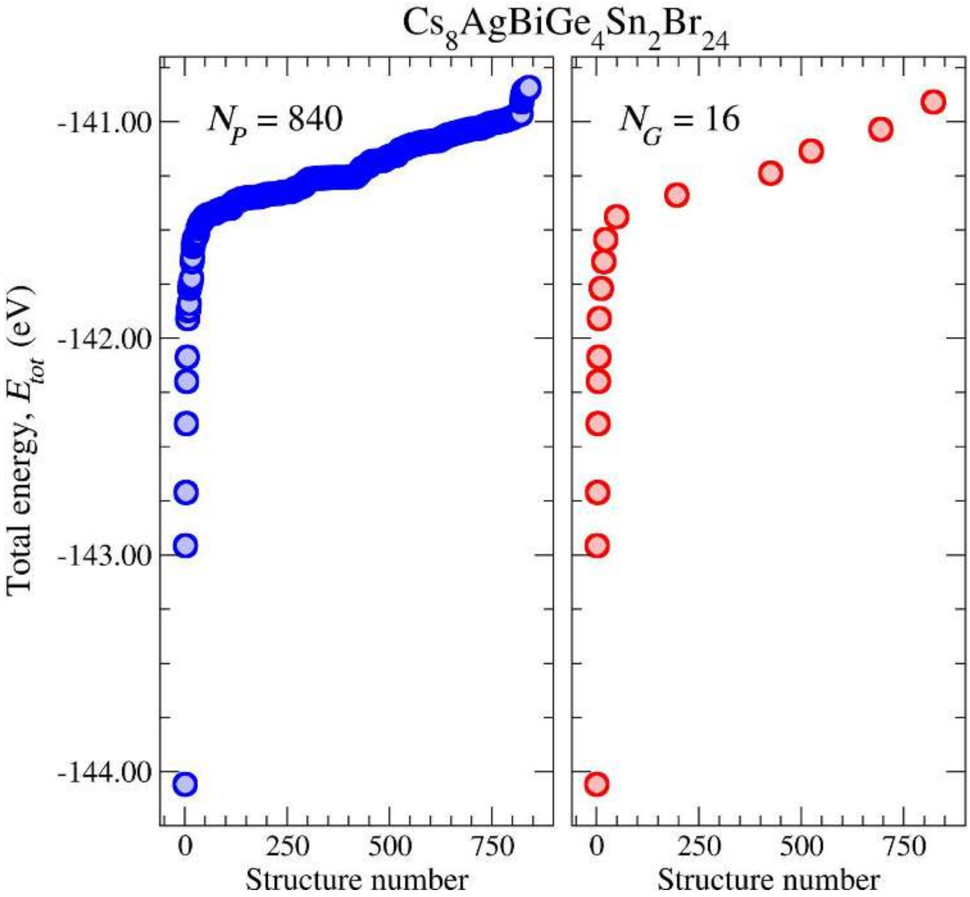

Figure S-15. Total energy as a function of the structure number for $Cs_8AgBiGe_4Sn_2Br_{24}$. Left (blue): energies of all N_P structures generated by permutations. Right (red): energies of the structures with the lowest energy from each group $\{n_k\}$ (the number of groups is given by N_G). The major spacing in y-axis (Δ_y) is $\Delta_y = 1.00$ eV.

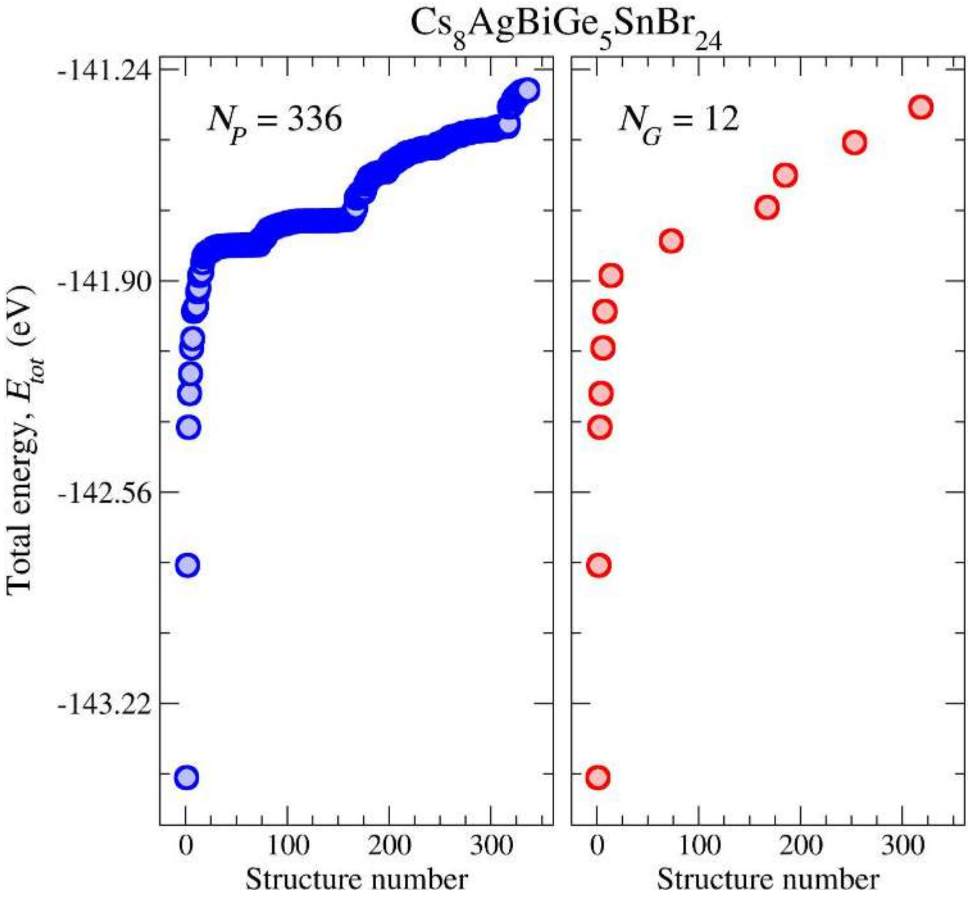

Figure S-16. Total energy as a function of the structure number for $Cs_8AgBiGe_5SnBr_{24}$. Left (blue): energies of all N_P structures generated by permutations. Right (red): energies of the structures with the lowest energy from each group $\{n_k\}$ (the number of groups is given by N_G). The major spacing in y-axis (Δ_y) is $\Delta_y = 0.66$ eV.

S-5 INVESTIGATION OF THE ROLE OF BLURRING HALOGEN ATOMIC POSITIONS FOR GEOMETRIC OPTIMIZATIONS

Jahn–Teller distortions lower the energy of perovskite structures, which is essential for describing the base structure of metal halide octahedra containing Sn and Ge.[6,7] Accordingly, three DFT-based approaches are compared:

- **Single-point not blurred:** Total energy from a self-consistent DFT calculation without structural optimization and with fixed Br positions.
- **Relaxed blurred:** Total energy after optimizing the stress tensor to satisfy equilibrium volume and force criteria, starting from a structure with blurred Br positions.

- **Relaxed not blurred:** Total energy after optimizing the stress tensor to satisfy equilibrium volume and force criteria, starting from a structure with fixed Br positions.

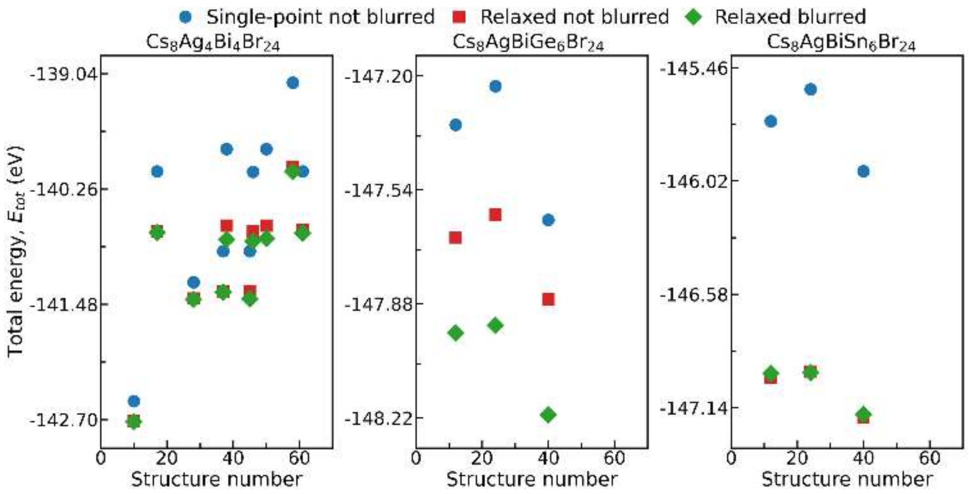

Figure S-17. Total energy comparison among different DFT calculations for $Cs_8Ag_4Bi_4Br_{24}$ (N_G = 10), $Cs_8AgBiGe_6Br_{24}$ (N_G = 3), and $Cs_8AgBiSn_6Br_{24}$ (N_G = 3).

Figure S-17 shows an energy gain when the initial blurred bromine atoms are considered in $Cs_8Ag_4Bi_4Br_{24}$ and $Cs_8AgBiGe_6Br_{24}$. For $Cs_8AgBiSn_6Br_{24}$, the largest energy difference between the "Relaxed not blurred" and "Relaxed blurred" structures is 0.019 eV, or 0.472 meV/atom. Thus, symmetry breaking in perovskites induced by halide blurring within the unit cell can lower the energy after structural optimization or produce an equivalent structural model of the solid (at the DFT-PBE+D3 level).

S-6 OPTIMIZATION OF THE PEROVSKITE MIXTURE STRUCTURES VIA STRESS TENSOR OPTIMIZATIONS

S-6.1 Comment on k-points Sampling of the Brillouin Zone

All **k**-meshes used here were generated using the Monkhorst-Pack scheme, ensuring that all sampled points form an equidistant **k**-point grid. Therefore, based on the fact that the electronic density can be calculated as

$$\rho(\mathbf{r}) = \frac{\Omega}{(2\pi)^3} \sum_n \int_{BZ} d\mathbf{k} f_{n\mathbf{k}} |\psi_n(\mathbf{r},\mathbf{k})|^2 \simeq \sum_n \sum_{\mathbf{k}} f_{n\mathbf{k}} |\psi_n(\mathbf{r},\mathbf{k})|^2 \,, \quad (1)$$

where Ω is the volume of the unit cell, n is the number of bands, BZ refers to the Brillouin zone, and $f_{n\mathbf{k}}$ is the orbital occupation; the electronic density is then "... *the sum over the Brillouin zone of the square modulus of the Bloch wavefunctions...*", as Dal Corso very well addressed[8]. In theory, it would be necessary to calculate the modulus in an infinite number of **k** points. However, in practice, this task becomes unfeasible.

$$\tilde{\rho} = \sum_{n} \sum_{\mathbf{k} \in IBZ} w_{\mathbf{k}} f_{n\mathbf{k}} |\psi_n(\mathbf{r},\mathbf{k})|^2 . \tag{2}$$

Thus, the electronic density can be computed in a reduced region of the Brillouin zone, the irreducible Brillouin zone (IBZ), where an unsymmetrized charge density (Equation 2) is evaluated using only a subset of special **k**-points. Through symmetry operations, these **k**-points reconstruct the full Brillouin zone mesh, allowing the symmetrized electronic density $\rho(\mathbf{r})$ to be obtained from the unsymmetrized one $\tilde{\rho}(\mathbf{r})$.

Table S-5. **k**-grids defined in the first Brillouin zone, number of **k**-points that constitute the **k**-mesh, and number of irreducible **k**-points in the Brillouin zone ($N_{\mathbf{k}}^{\mathrm{IBZ}}$), employed for the convergence tests perforomed on the pristine compounds of this study.

**k**-mesh	$N_{\mathbf{k}}$	$N_{\mathbf{k}}^{\mathrm{IBZ}}$
1×1×1	1	1
2×2×2	8	8
3×3×3	27	14
4×4×4	64	36

S-6.2 Computational Convergence of Stress Tensor Optimizations

Table S-6. Total energy (E_{tot}) convergence with respect to the number of reciprocal integration points within the first Brillouin zone and for the pristine compounds: $Cs_8Ag_4Bi_4Br_{24}$, $Cs_8Ge_8Br_{24}$, $Cs_8Sn_8Br_{24}$, and $Cs_8Pb_8Br_{24}$, at equilibrium volume. $N_{\mathbf{k}}$ is the number of **k**-points constituting the grid, and $N_{\mathbf{k}}^{\mathrm{IBZ}}$ is the number of irreducible **k**-points. Below each E_{tot} value (up to the forth decimal) we have the relative total energy (ΔE_{tot}) with respect to the system with the greatest $N_{\mathbf{k}}$, i.e., $\Delta E_{tot} = E_{tot}^{i} - E_{tot}^{N_{\mathbf{k}}=64}$. All values are given in eV.

$N_{\mathbf{k}}$	$N_{\mathbf{k}}^{\mathrm{IBZ}}$	Energy	$Cs_8Ag_4Bi_4Br_{24}$	$Cs_8Ge_8Br_{24}$	$Cs_8Sn_8Br_{24}$	$Cs_8Pb_8Br_{24}$
1	1	E_{tot}	−141.737 243	−147.999 317	−146.617 022	−147.362 410
		ΔE_{tot}	0.9699	2.8438	3.0114	2.6888
8	8	E_{tot}	−142.718 570	−150.596 386	−149.189 459	−149.830 275
		ΔE_{tot}	−0.0114	0.2468	0.4390	0.2209
27	14	E_{tot}	−142.706 927	−150.818 408	−149.572 575	−150.026 613
		ΔE_{tot}	0.0002	0.0247	0.0558	0.0246
64	36	E_{tot}	−142.707 159	−150.843 153	−149.628 417	−150.051 202

Table S-7. Equilibrium lattice constant (a_0) convergence with respect to the number of reciprocal integration points within the first Brillouin zone and for the pristine compounds: $Cs_8Ag_4Bi_4Br_{24}$, $Cs_8Ge_8Br_{24}$, $Cs_8Sn_8Br_{24}$, and $Cs_8Pb_8Br_{24}$, at equilibrium volume. $N_{\mathbf{k}}$ is the number of **k**-points constituting the grid, and $N_{\mathbf{k}}^{\mathrm{IBZ}}$ is the number of irreducible **k**-points. The relative lattice constant (Δa_0) is also shown, and it was obtained with respect to the experimental value, i.e., $\Delta a_0 = a_0^{i} - a_0^{\mathrm{Exp.}}$. All values are given in Å.

$N_{\mathbf{k}}$	$N_{\mathbf{k}}^{\mathrm{IBZ}}$	$Cs_8Ag_4Bi_4Br_{24}$		$Cs_8Ge_8Br_{24}$		$Cs_8Sn_8Br_{24}$		$Cs_8Pb_8Br_{24}$	
		a_0	Δa_0	a_0	Δa_0	a_0	Δa_0	a_0	Δa_0
1	1	11.504	0.233	11.815	0.545	12.044	0.435	11.937	0.248
8	8	11.368	0.097	11.350	0.080	11.694	0.085	11.863	0.174
27	14	11.366	0.095	11.290	0.020	11.668	0.059	11.850	0.161
64	36	11.366	0.095	11.290	0.020	11.679	0.070	11.849	0.160
Exp.		11.271[4]		11.270[9]		11.609[10]		11.689[11]	

S-6.3 Strategies to Speed up Stress Tensor Calculations

Equilibrium volumes were obtained by minimizing the stress tensor while constraining all unit cells to be cubic. Since the four pristine compounds, $Cs_2AgBiBr_6$[4], $CsGeBr_3$[9], $CsSnBr_3$[10], and $CsPbBr_3$[11], crystallize in the cubic perovskite phase, assuming cubic unit cells for all alloys is reasonable. This constraint improves (*i*) computational

efficiency, (*ii*) the description of structural and electronic properties, and (*iii*) data analysis consistency.

In addition, below we show an example of INCAR file, the principal VASP input file where the calculations to be performed and their initial conditions are set up.

```
SYSTEM = Cs2AgBiBr6 !  INCAR file starts here

ENCUT = 563.151

ALGO = Normal

NELMIN = 6

NELM = 120

NELMDL = -12

EDIFF = 1.0E-5

AMIX = 0.1000

BMIX = 0.0001

PREC = Normal

ISPIN = 1

ADDGRID = .TRUE.

LASPH = .TRUE.

LREAL = Auto

IVDW = 11

NSW = 10

EDIFFG = -0.025

IBRION = 2

ISIF = 3

POTIM = 0.50

ISMEAR = 0

SIGMA = 0.01

LORBIT = 10

NEDOS = 61

NWRITE = 1
```

```
LWAVE = .FALSE.
LCHARG = .FALSE.
NCORE = 5
LPLANE = .TRUE.
EOF ! INCAR file finishes here
```

To optimize computational resources, we focus on four tags: (*i*) `ENCUT` (plane-wave cutoff energy in eV), (*ii*) `NSW` (number of ionic steps), (*iii*) `IBRION` (method of structural optimization), and (*iv*) `ISIF` (controls stress-tensor calculation and constrained structural degrees of freedom).

The optimization procedures used in this work are summarized in Table S-8. The number of optimization runs differed from the number of structures. No calculations were submitted with many ionic steps in anticipation of mid-run force convergence. Instead, each structure was first brought to its equilibrium volume (V_0), then forces between atom pairs were optimized at V_0 until the force criteria were met. Because the total energy is highly sensitive to volume and to avoid Pulay stress from unconverged calculations, a higher cutoff energy was used for stress tensor calculations than for force relaxation (Table S-8). As examples, Figures S-18–S-20 show pressures, relative volumes, and relative energies at each ionic step and for each optimization run for three representative compounds.

Table S-8. Values used for each of the above-mentioned tags, i.e., `ENCUT`, `NSW`, `IBRION`, and `ISIF`, in each of the optimization processes, here named as RUN. (*i*) About `ENCUT`: 563.151 eV = 1.5×$\mathtt{ENMAX}_{max}$ and 422.363 eV = 1.125×$\mathtt{ENMAX}_{max}$, where $\mathtt{ENMAX}_{max}$ = 375.434 eV, according to Table S-1. (*ii*) About `IBRION`: `IBRION` = 2 refers to the residual minimization method with direct inversion in the iterative subspace (RMM-DIIS)[12], and `IBRION` = 1 refers to the conjugate gradient (CG) method[13]. About `ISIF`: `IBRION` = 3 establishes that the forces and stress tensor are calculated in each ionic (`NSW`) step, and the atomic position, cell shape, and cell volume are allowed to change; `IBRION` = 0 allows the forces to be calculated in each `NSW` step and allows the atomic position to change.

RUN number	INCAR tag	Simple and double perovskites	Double perovskite with one divalent metal	Double perovskite with two divalent metals
1	`ENCUT`	563.151	563.151	563.151
	`NSW`	10	10	10
	`IBRION`	2	2	2
	`ISIF`	3	3	3
2	`ENCUT`	563.151	563.151	563.151
	`NSW`	20	20	10
	`IBRION`	2	2	2
	`ISIF`	3	3	3
3	`ENCUT`	563.151	563.151	563.151
	`NSW`	40	40	20
	`IBRION`	2	2	2
	`ISIF`	3	3	3
4	`ENCUT`	563.151	563.151	563.151
	`NSW`	40	40	20
	`IBRION`	1	1	1
	`ISIF`	3	3	3
5	`ENCUT`	422.363	422.363	563.151
	`NSW`	80	40	20
	`IBRION`	2	2	1
	`ISIF`	0	0	3
6	`ENCUT`	422.363	422.363	422.363
	`NSW`	80	120	40
	`IBRION`	1	1	2
	`ISIF`	0	0	0
7	`ENCUT`			422.363
	`NSW`			120
	`IBRION`			1
	`ISIF`			0

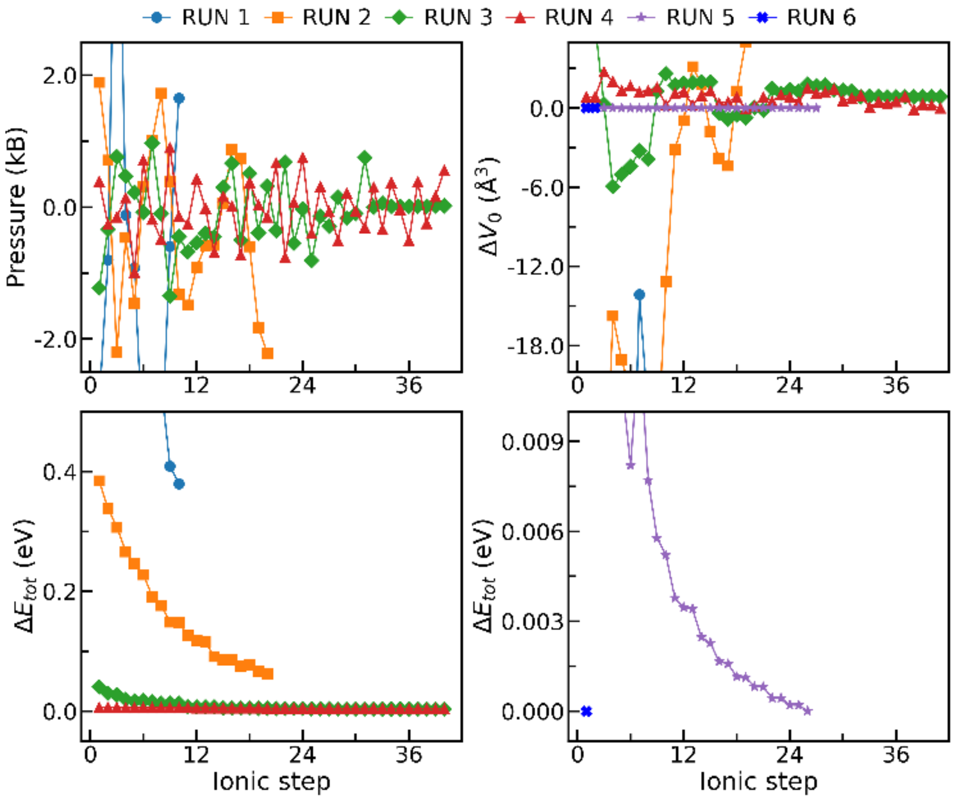

Figure S-18. Structural optimization process for the $CsGeBr_3$ perovskite model. The relative equilibrium volume (ΔV_0) is defined as $\Delta V = V^i - V_0$, where V^i and V_0 are the volume at a particular ionic step and the equilibrium volume, respectively. The relative total energy (ΔE_0) is defined as $\Delta E_{tot} = E^i_{tot} - E^0_{tot}$, where E^i_{tot} and E^0_{tot} are the total energy at a particular ionic step and the total energy at equilibrium, respectively. All calculations were performed at DFT-PBE+D3 level.

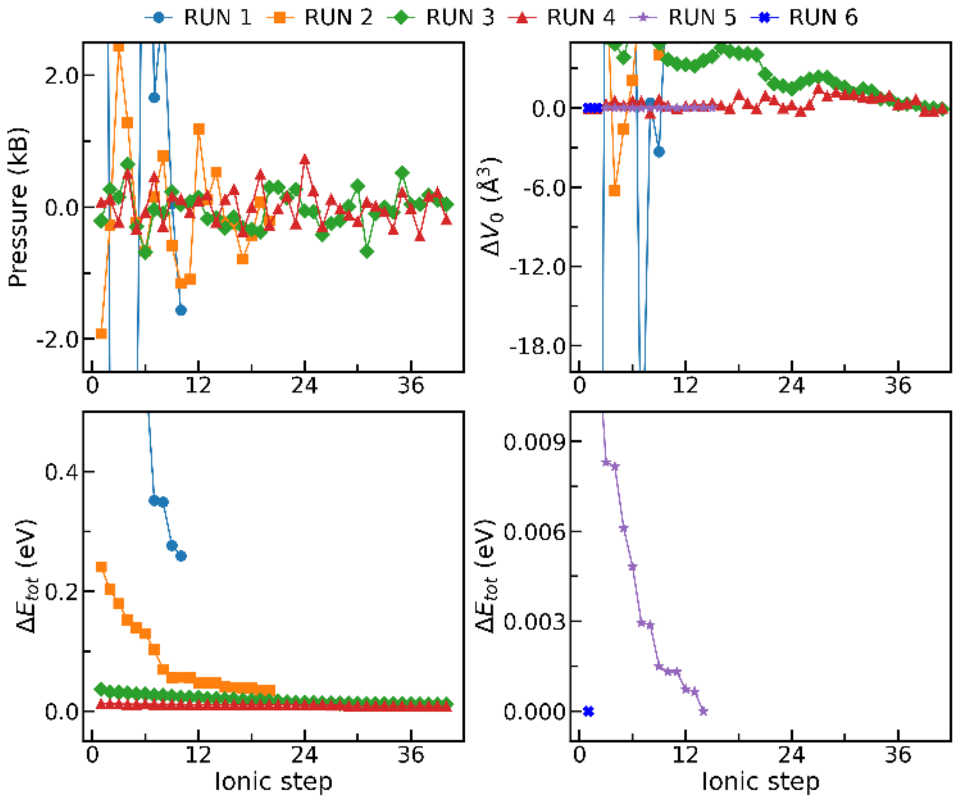

Figure S-19. Structural optimization process for the $Cs_8Ag_2Bi_2Sn_4Br_{24}$ (or $Cs_2Ag_{0.50}Bi_{0.50}SnBr_6$) compound model number 50. The relative equilibrium volume (ΔV_0) is defined as $\Delta V = V^i - V_0$, where V^i and V_0 are the volume at a particular ionic step and the equilibrium volume, respectively. The relative total energy (ΔE_0) is defined as $\Delta E_{tot} = E^i_{tot} - E^0_{tot}$, where E^i_{tot} and E^0_{tot} are the total energy at a particular ionic step and the total energy at equilibrium, respectively. All calculations were performed at DFT-PBE+D3 level.

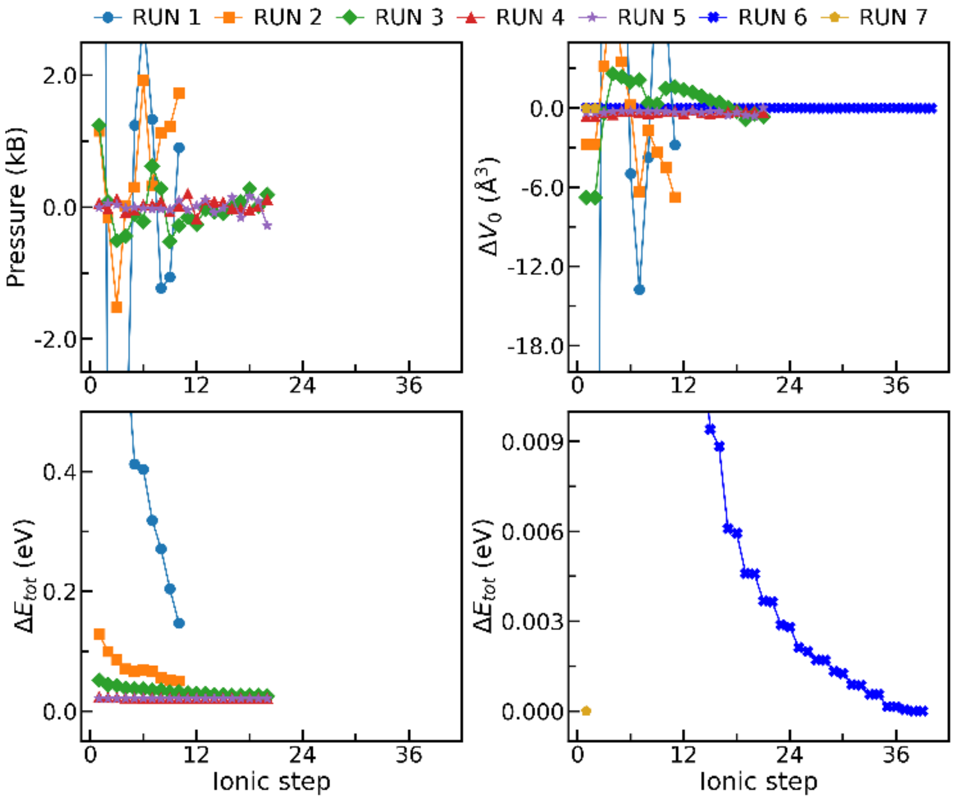

Figure S-20. Structural optimization process for the $Cs_8Ag_2Bi_2Ge_2Pb_2Br_{24}$ (or $Cs_2Ag_{0.50}Bi_{0.50}Ge_{0.50}Pb_{0.50}SnBr_6$) compound model number 1185. The relative equilibrium volume (ΔV_0) is defined as $\Delta V = V^i - V_0$, where V^i and V_0 are the volume at a particular ionic step and the equilibrium volume, respectively. The relative total energy (ΔE_0) is defined as $\Delta E_{tot} = E^i_{tot} - E^0_{tot}$, where E^i_{tot} and E^0_{tot} are the total energy at a particular ionic step and the total energy at equilibrium, respectively. All calculations were performed at DFT-PBE+D3 level.

S-6.4 Total Energy, Lattice Parameter, and Volume of all Optimized Structures at Equilibrium

Table S-9. Total energy (E_{tot}), equilibrium lattice parameter (a_0), and equilibrium volume (V_0) of all optimized structures given in eV, Å, and Å^3, respectively. $N_{Str.}$ refers to "structure number", which follows the enumeration given in the permutation process. The subscripts in the metal composition column indicate the number of atoms (of that species) within the unit-cell.

Metal composition	$N_{Str.}$	E_{tot}	a_0	V_0
Ag_4Bi_4	10	−142.721 303	11.365	1468.10
Ag_4Bi_4	28	−141.427 605	11.399	1481.15

Continued on next page

Table S-9 – Continued from previous page

Metal composition	$N_{\text{Str.}}$	E_{tot}	a_0	V_0
Ag_4Bi_4	45	−141.422 362	11.398	1480.63
Ag_4Bi_4	37	−141.350 744	11.399	1480.99
Ag_4Bi_4	46	−140.811 682	11.427	1491.96
Ag_4Bi_4	38	−140.794 068	11.430	1493.23
Ag_4Bi_4	50	−140.783 326	11.425	1491.23
Ag_4Bi_4	61	−140.725 715	11.425	1491.38
Ag_4Bi_4	17	−140.719 723	11.430	1493.38
Ag_4Bi_4	58	−140.076 001	11.449	1500.83
$Ag_3Bi_3Ge_2$	338	−144.243 780	11.352	1462.93
$Ag_3Bi_3Ge_2$	500	−144.005 504	11.352	1462.97
$Ag_3Bi_3Ge_2$	491	−143.750 586	11.342	1459.16
$Ag_3Bi_3Ge_2$	411	−143.607 440	11.339	1457.94
$Ag_3Bi_3Ge_2$	475	−143.598 512	11.370	1469.73
$Ag_3Bi_3Ge_2$	280	−143.440 275	11.376	1472.19
$Ag_3Bi_3Ge_2$	555	−143.360 981	11.359	1465.67
$Ag_3Bi_3Ge_2$	192	−143.360 728	11.358	1465.28
$Ag_3Bi_3Ge_2$	161	−143.358 029	11.357	1464.88
$Ag_3Bi_3Ge_2$	466	−143.192 041	11.341	1458.51
$Ag_3Bi_3Ge_2$	155	−143.180 944	11.339	1457.88
$Ag_3Bi_3Ge_2$	363	−143.158 386	11.364	1467.66
$Ag_3Bi_3Ge_2$	14	−143.141 474	11.365	1468.12
$Ag_3Bi_3Ge_2$	55	−143.022 698	11.370	1469.91
$Ag_3Bi_3Ge_2$	243	−142.930 077	11.363	1466.99
$Ag_3Bi_3Ge_2$	261	−142.873 289	11.386	1476.18
$Ag_3Bi_3Ge_2$	539	−142.860 199	11.382	1474.53
$Ag_3Bi_3Ge_2$	283	−142.805 590	11.384	1475.15
$Ag_3Bi_3Ge_2$	360	−142.708 712	11.363	1467.16
$Ag_3Bi_3Ge_2$	490	−142.595 827	11.370	1469.70
$Ag_3Bi_3Ge_2$	149	−142.587 339	11.372	1470.59
$Ag_3Bi_3Sn_2$	338	−143.965 636	11.458	1504.15
$Ag_3Bi_3Sn_2$	500	−143.840 280	11.459	1504.55
$Ag_3Bi_3Sn_2$	411	−143.470 130	11.457	1503.70
$Ag_3Bi_3Sn_2$	491	−143.446 431	11.471	1509.51
$Ag_3Bi_3Sn_2$	475	−143.321 093	11.464	1506.70
$Ag_3Bi_3Sn_2$	280	−143.188 283	11.487	1515.64
$Ag_3Bi_3Sn_2$	466	−143.168 833	11.446	1499.68

Continued on next page

Table S-9 – Continued from previous page

Metal composition	$N_{\mathrm{Str.}}$	E_{tot}	a_0	V_0
$Ag_3Bi_3Sn_2$	155	−143.166 508	11.445	1499.25
$Ag_3Bi_3Sn_2$	192	−143.163 222	11.472	1509.99
$Ag_3Bi_3Sn_2$	161	−143.161 221	11.469	1508.78
$Ag_3Bi_3Sn_2$	555	−143.157 519	11.470	1509.20
$Ag_3Bi_3Sn_2$	14	−142.975 080	11.456	1503.68
$Ag_3Bi_3Sn_2$	363	−142.973 325	11.455	1503.15
$Ag_3Bi_3Sn_2$	55	−142.935 694	11.475	1511.08
$Ag_3Bi_3Sn_2$	243	−142.815 973	11.465	1507.02
$Ag_3Bi_3Sn_2$	539	−142.658 324	11.468	1508.21
$Ag_3Bi_3Sn_2$	261	−142.657 471	11.470	1509.20
$Ag_3Bi_3Sn_2$	283	−142.548 276	11.462	1506.00
$Ag_3Bi_3Sn_2$	360	−142.532 625	11.468	1508.27
$Ag_3Bi_3Sn_2$	490	−142.398 362	11.484	1514.62
$Ag_3Bi_3Sn_2$	149	−142.386 107	11.469	1508.54
$Ag_3Bi_3Pb_2$	338	−144.026 719	11.506	1523.20
$Ag_3Bi_3Pb_2$	500	−143.969 760	11.508	1523.95
$Ag_3Bi_3Pb_2$	411	−143.593 219	11.505	1522.87
$Ag_3Bi_3Pb_2$	280	−143.522 892	11.534	1534.23
$Ag_3Bi_3Pb_2$	491	−143.478 833	11.515	1526.68
$Ag_3Bi_3Pb_2$	475	−143.383 387	11.517	1527.64
$Ag_3Bi_3Pb_2$	555	−143.263 050	11.516	1527.23
$Ag_3Bi_3Pb_2$	192	−143.256 280	11.521	1529.19
$Ag_3Bi_3Pb_2$	161	−143.254 170	11.513	1525.86
$Ag_3Bi_3Pb_2$	155	−143.196 254	11.499	1520.63
$Ag_3Bi_3Pb_2$	466	−143.195 205	11.503	1521.98
$Ag_3Bi_3Pb_2$	363	−142.959 247	11.511	1525.08
$Ag_3Bi_3Pb_2$	14	−142.952 452	11.512	1525.78
$Ag_3Bi_3Pb_2$	261	−142.900 035	11.516	1527.18
$Ag_3Bi_3Pb_2$	243	−142.865 400	11.516	1527.13
$Ag_3Bi_3Pb_2$	55	−142.847 908	11.518	1528.03
$Ag_3Bi_3Pb_2$	149	−142.663 047	11.522	1529.61
$Ag_3Bi_3Pb_2$	539	−142.630 589	11.530	1532.95
$Ag_3Bi_3Pb_2$	360	−142.601 265	11.513	1525.96
$Ag_3Bi_3Pb_2$	283	−142.590 773	11.523	1530.04
$Ag_3Bi_3Pb_2$	490	−142.448 107	11.523	1529.97
$Ag_2Bi_2Ge_4$	50	−145.840 512	11.317	1449.45

Continued on next page

Table S-9 – Continued from previous page

Metal composition	$N_{\text{Str.}}$	E_{tot}	a_0	V_0
$Ag_2Bi_2Ge_4$	83	−145.805 951	11.313	1447.93
$Ag_2Bi_2Ge_4$	339	−145.746 309	11.354	1463.81
$Ag_2Bi_2Ge_4$	112	−145.738 889	11.353	1463.25
$Ag_2Bi_2Ge_4$	391	−145.580 451	11.332	1455.01
$Ag_2Bi_2Ge_4$	406	−145.580 195	11.336	1456.79
$Ag_2Bi_2Ge_4$	143	−145.579 482	11.337	1457.26
$Ag_2Bi_2Ge_4$	49	−145.442 044	11.335	1456.43
$Ag_2Bi_2Ge_4$	128	−145.440 693	11.337	1456.99
$Ag_2Bi_2Ge_4$	237	−145.438 957	11.335	1456.24
$Ag_2Bi_2Ge_4$	270	−145.357 441	11.325	1452.60
$Ag_2Bi_2Ge_4$	97	−145.356 338	11.322	1451.44
$Ag_2Bi_2Ge_4$	172	−145.295 072	11.339	1457.98
$Ag_2Bi_2Ge_4$	311	−145.070 751	11.334	1455.99
$Ag_2Bi_2Ge_4$	225	−145.066 863	11.332	1455.33
$Ag_2Bi_2Sn_4$	50	−145.231 428	11.522	1529.56
$Ag_2Bi_2Sn_4$	83	−145.186 723	11.536	1535.18
$Ag_2Bi_2Sn_4$	112	−145.140 124	11.547	1539.68
$Ag_2Bi_2Sn_4$	339	−145.110 299	11.560	1544.99
$Ag_2Bi_2Sn_4$	143	−145.005 734	11.519	1528.51
$Ag_2Bi_2Sn_4$	406	−145.001 569	11.516	1527.38
$Ag_2Bi_2Sn_4$	391	−144.996 784	11.521	1529.32
$Ag_2Bi_2Sn_4$	237	−144.928 766	11.533	1534.12
$Ag_2Bi_2Sn_4$	270	−144.909 298	11.515	1526.68
$Ag_2Bi_2Sn_4$	49	−144.885 757	11.542	1537.50
$Ag_2Bi_2Sn_4$	97	−144.875 369	11.513	1526.06
$Ag_2Bi_2Sn_4$	128	−144.837 458	11.533	1534.20
$Ag_2Bi_2Sn_4$	172	−144.745 988	11.519	1528.27
$Ag_2Bi_2Sn_4$	225	−144.579 145	11.515	1526.98
$Ag_2Bi_2Sn_4$	311	−144.559 997	11.518	1527.99
$Ag_2Bi_2Pb_4$	339	−145.565 596	11.666	1587.72
$Ag_2Bi_2Pb_4$	50	−145.438 215	11.636	1575.50
$Ag_2Bi_2Pb_4$	83	−145.359 966	11.639	1576.56
$Ag_2Bi_2Pb_4$	112	−145.315 372	11.673	1590.50
$Ag_2Bi_2Pb_4$	128	−145.313 839	11.656	1583.60
$Ag_2Bi_2Pb_4$	49	−145.294 076	11.656	1583.44
$Ag_2Bi_2Pb_4$	391	−145.139 863	11.646	1579.38

Continued on next page

Table S-9 – Continued from previous page

Metal composition	$N_{\mathrm{Str.}}$	E_{tot}	a_0	V_0
$Ag_2Bi_2Pb_4$	406	−145.137 141	11.646	1579.58
$Ag_2Bi_2Pb_4$	143	−145.137 061	11.641	1577.30
$Ag_2Bi_2Pb_4$	237	−145.035 932	11.654	1582.89
$Ag_2Bi_2Pb_4$	97	−144.989 425	11.631	1573.61
$Ag_2Bi_2Pb_4$	270	−144.983 777	11.631	1573.59
$Ag_2Bi_2Pb_4$	225	−144.854 909	11.636	1575.59
$Ag_2Bi_2Pb_4$	172	−144.829 903	11.651	1581.73
$Ag_2Bi_2Pb_4$	311	−144.771 963	11.644	1578.74
$AgBiGe_6$	40	−148.211 053	11.337	1457.19
$AgBiGe_6$	12	−147.966 657	11.324	1451.95
$AgBiGe_6$	24	−147.944 471	11.331	1454.66
$AgBiSn_6$	40	−147.173 587	11.614	1566.63
$AgBiSn_6$	12	−146.970 887	11.619	1568.38
$AgBiSn_6$	24	−146.967 010	11.613	1566.15
$AgBiPb_6$	40	−147.768 541	11.740	1617.90
$AgBiPb_6$	12	−147.526 514	11.753	1623.36
$AgBiPb_6$	24	−147.480 704	11.767	1629.17
Ag_3Bi_3GeSn	474	−144.156 503	11.390	1477.57
Ag_3Bi_3GeSn	1075	−143.982 466	11.411	1485.84
Ag_3Bi_3GeSn	1068	−143.575 669	11.410	1485.41
Ag_3Bi_3GeSn	499	−143.528 060	11.397	1480.46
Ag_3Bi_3GeSn	1112	−143.527 977	11.403	1482.83
Ag_3Bi_3GeSn	426	−143.525 326	11.397	1480.40
Ag_3Bi_3GeSn	466	−143.524 914	11.399	1481.29
Ag_3Bi_3GeSn	379	−143.409 921	11.420	1489.39
Ag_3Bi_3GeSn	1096	−143.323 758	11.445	1499.07
Ag_3Bi_3GeSn	626	−143.263 146	11.411	1485.79
Ag_3Bi_3GeSn	1045	−143.239 625	11.423	1490.60
Ag_3Bi_3GeSn	922	−143.187 856	11.410	1485.53
Ag_3Bi_3GeSn	658	−143.158 479	11.440	1497.22
Ag_3Bi_3GeSn	284	−143.154 286	11.439	1496.74
Ag_3Bi_3GeSn	528	−143.132 315	11.398	1480.60
Ag_3Bi_3GeSn	330	−143.115 645	11.438	1496.28
Ag_3Bi_3GeSn	20	−143.010 419	11.409	1485.13
Ag_3Bi_3GeSn	734	−143.005 398	11.402	1482.43
Ag_3Bi_3GeSn	761	−143.002 170	11.405	1483.57

Continued on next page

Table S-9 – Continued from previous page

Metal composition	$N_{\text{Str.}}$	E_{tot}	a_0	V_0
Ag_3Bi_3GeSn	889	−143.001 462	11.405	1483.66
Ag_3Bi_3GeSn	276	−142.996 741	11.404	1483.30
Ag_3Bi_3GeSn	972	−142.904 768	11.432	1493.94
Ag_3Bi_3GeSn	1057	−142.876 294	11.418	1488.43
Ag_3Bi_3GeSn	533	−142.864 433	11.418	1488.62
Ag_3Bi_3GeSn	509	−142.741 680	11.427	1492.27
Ag_3Bi_3GeSn	692	−142.738 150	11.434	1494.72
Ag_3Bi_3GeSn	335	−142.494 542	11.438	1496.36
Ag_3Bi_3GeSn	1024	−142.477 975	11.431	1493.74
Ag_3Bi_3GeSn	171	−142.469 894	11.428	1492.41
Ag_3Bi_3GePb	474	−144.243 466	11.422	1490.25
Ag_3Bi_3GePb	1075	−144.083 494	11.436	1495.80
Ag_3Bi_3GePb	1068	−143.577 715	11.440	1497.15
Ag_3Bi_3GePb	1112	−143.567 271	11.434	1494.66
Ag_3Bi_3GePb	426	−143.565 641	11.435	1495.32
Ag_3Bi_3GePb	466	−143.564 980	11.433	1494.31
Ag_3Bi_3GePb	499	−143.560 275	11.432	1494.08
Ag_3Bi_3GePb	1096	−143.457 176	11.481	1513.30
Ag_3Bi_3GePb	379	−143.438 392	11.444	1498.77
Ag_3Bi_3GePb	626	−143.309 212	11.443	1498.42
Ag_3Bi_3GePb	658	−143.306 908	11.459	1504.77
Ag_3Bi_3GePb	1045	−143.253 909	11.448	1500.36
Ag_3Bi_3GePb	330	−143.245 611	11.455	1503.20
Ag_3Bi_3GePb	528	−143.237 393	11.430	1493.16
Ag_3Bi_3GePb	922	−143.185 489	11.444	1498.68
Ag_3Bi_3GePb	284	−143.185 038	11.461	1505.46
Ag_3Bi_3GePb	20	−143.022 787	11.440	1497.37
Ag_3Bi_3GePb	276	−143.021 603	11.440	1497.38
Ag_3Bi_3GePb	889	−143.000 651	11.439	1496.67
Ag_3Bi_3GePb	734	−142.999 493	11.439	1496.67
Ag_3Bi_3GePb	761	−142.997 027	11.441	1497.40
Ag_3Bi_3GePb	972	−142.918 968	11.462	1505.80
Ag_3Bi_3GePb	533	−142.893 653	11.450	1501.15
Ag_3Bi_3GePb	1057	−142.880 563	11.452	1501.78
Ag_3Bi_3GePb	509	−142.832 980	11.453	1502.23
Ag_3Bi_3GePb	335	−142.804 378	11.461	1505.31

Continued on next page

Table S-9 – Continued from previous page

Metal composition	$N_{\text{Str.}}$	E_{tot}	a_0	V_0
Ag_3Bi_3GePb	1024	−142.767 598	11.459	1504.58
Ag_3Bi_3GePb	692	−142.731 526	11.457	1504.01
Ag_3Bi_3GePb	171	−142.496 454	11.451	1501.51
Ag_3Bi_3SnPb	474	−144.045 965	11.476	1511.34
Ag_3Bi_3SnPb	1075	−143.956 058	11.481	1513.37
Ag_3Bi_3SnPb	499	−143.527 197	11.479	1512.75
Ag_3Bi_3SnPb	1112	−143.524 637	11.484	1514.50
Ag_3Bi_3SnPb	426	−143.523 648	11.480	1513.10
Ag_3Bi_3SnPb	466	−143.522 129	11.478	1512.35
Ag_3Bi_3SnPb	1068	−143.465 326	11.497	1519.53
Ag_3Bi_3SnPb	1096	−143.422 699	11.509	1524.59
Ag_3Bi_3SnPb	379	−143.345 446	11.502	1521.71
Ag_3Bi_3SnPb	658	−143.272 222	11.512	1525.65
Ag_3Bi_3SnPb	284	−143.272 053	11.512	1525.80
Ag_3Bi_3SnPb	1045	−143.187 938	11.504	1522.65
Ag_3Bi_3SnPb	626	−143.185 484	11.494	1518.31
Ag_3Bi_3SnPb	528	−143.176 199	11.466	1507.33
Ag_3Bi_3SnPb	922	−143.147 069	11.493	1518.17
Ag_3Bi_3SnPb	972	−143.036 151	11.507	1523.71
Ag_3Bi_3SnPb	330	−143.030 979	11.510	1524.67
Ag_3Bi_3SnPb	889	−142.944 167	11.480	1512.89
Ag_3Bi_3SnPb	20	−142.943 016	11.484	1514.53
Ag_3Bi_3SnPb	761	−142.942 793	11.480	1513.02
Ag_3Bi_3SnPb	276	−142.940 682	11.483	1514.13
Ag_3Bi_3SnPb	734	−142.938 352	11.479	1512.51
Ag_3Bi_3SnPb	1057	−142.841 022	11.504	1522.54
Ag_3Bi_3SnPb	533	−142.834 930	11.504	1522.28
Ag_3Bi_3SnPb	692	−142.820 464	11.507	1523.50
Ag_3Bi_3SnPb	509	−142.805 670	11.508	1523.99
Ag_3Bi_3SnPb	335	−142.664 590	11.515	1526.66
Ag_3Bi_3SnPb	1024	−142.622 517	11.508	1524.19
Ag_3Bi_3SnPb	171	−142.419 732	11.509	1524.52
$Ag_2Bi_2GeSn_3$	649	−145.755 297	11.485	1514.90
$Ag_2Bi_2GeSn_3$	84	−145.557 202	11.476	1511.22
$Ag_2Bi_2GeSn_3$	224	−145.508 112	11.489	1516.58
$Ag_2Bi_2GeSn_3$	1248	−145.503 823	11.494	1518.65

Continued on next page

Table S-9 – Continued from previous page

Metal composition	$N_{\text{Str.}}$	E_{tot}	a_0	V_0
$Ag_2Bi_2GeSn_3$	1664	−145.502 595	11.490	1516.92
$Ag_2Bi_2GeSn_3$	532	−145.500 284	11.491	1517.13
$Ag_2Bi_2GeSn_3$	390	−145.399 700	11.487	1515.55
$Ag_2Bi_2GeSn_3$	377	−145.328 465	11.484	1514.57
$Ag_2Bi_2GeSn_3$	934	−145.280 838	11.485	1514.90
$Ag_2Bi_2GeSn_3$	1586	−145.275 755	11.511	1525.32
$Ag_2Bi_2GeSn_3$	764	−145.230 932	11.477	1511.82
$Ag_2Bi_2GeSn_3$	894	−145.226 588	11.481	1513.21
$Ag_2Bi_2GeSn_3$	1625	−145.221 322	11.502	1521.64
$Ag_2Bi_2GeSn_3$	385	−145.216 895	11.501	1521.35
$Ag_2Bi_2GeSn_3$	179	−145.112 991	11.472	1509.90
$Ag_2Bi_2GeSn_3$	1217	−145.109 915	11.469	1508.51
$Ag_2Bi_2GeSn_3$	218	−145.020 025	11.507	1523.63
$Ag_2Bi_2GeSn_3$	966	−145.007 082	11.482	1513.76
$Ag_2Bi_2GeSn_3$	1258	−144.995 158	11.508	1524.10
$Ag_2Bi_2GeSn_3$	693	−144.937 132	11.469	1508.52
$Ag_2Bi_2GeSn_3$	1138	−144.936 000	11.495	1518.92
$Ag_2Bi_2GeSn_3$	1647	−144.934 022	11.470	1508.97
$Ag_2Bi_2GeSn_3$	664	−144.932 801	11.495	1518.89
$Ag_2Bi_2GeSn_3$	901	−144.864 408	11.492	1517.67
$Ag_2Bi_2GePb_3$	649	−145.873 286	11.539	1536.48
$Ag_2Bi_2GePb_3$	84	−145.736 947	11.543	1538.07
$Ag_2Bi_2GePb_3$	224	−145.642 454	11.563	1545.90
$Ag_2Bi_2GePb_3$	1248	−145.639 424	11.561	1545.20
$Ag_2Bi_2GePb_3$	1664	−145.636 010	11.564	1546.35
$Ag_2Bi_2GePb_3$	390	−145.631 869	11.561	1545.32
$Ag_2Bi_2GePb_3$	532	−145.628 408	11.564	1546.36
$Ag_2Bi_2GePb_3$	1625	−145.600 109	11.574	1550.41
$Ag_2Bi_2GePb_3$	385	−145.595 023	11.582	1553.60
$Ag_2Bi_2GePb_3$	377	−145.444 840	11.550	1540.69
$Ag_2Bi_2GePb_3$	1586	−145.433 836	11.569	1548.29
$Ag_2Bi_2GePb_3$	1258	−145.383 233	11.570	1548.92
$Ag_2Bi_2GePb_3$	934	−145.381 308	11.571	1549.06
$Ag_2Bi_2GePb_3$	218	−145.374 412	11.574	1550.35
$Ag_2Bi_2GePb_3$	894	−145.361 687	11.525	1530.86
$Ag_2Bi_2GePb_3$	764	−145.359 368	11.522	1529.51

Continued on next page

Table S-9 – Continued from previous page

Metal composition	$N_{\text{Str.}}$	E_{tot}	a_0	V_0
$Ag_2Bi_2GePb_3$	966	−145.300 239	11.580	1552.74
$Ag_2Bi_2GePb_3$	179	−145.188 849	11.559	1544.30
$Ag_2Bi_2GePb_3$	1217	−145.184 215	11.568	1547.98
$Ag_2Bi_2GePb_3$	664	−145.147 247	11.588	1556.16
$Ag_2Bi_2GePb_3$	1138	−145.049 101	11.587	1555.50
$Ag_2Bi_2GePb_3$	1647	−144.966 704	11.560	1544.95
$Ag_2Bi_2GePb_3$	693	−144.966 520	11.557	1543.53
$Ag_2Bi_2GePb_3$	901	−144.904 550	11.560	1544.72
$Ag_2Bi_2SnPb_3$	649	−145.912 067	11.614	1566.65
$Ag_2Bi_2SnPb_3$	532	−145.697 441	11.615	1566.83
$Ag_2Bi_2SnPb_3$	1664	−145.688 939	11.612	1565.86
$Ag_2Bi_2SnPb_3$	84	−145.560 536	11.613	1566.03
$Ag_2Bi_2SnPb_3$	1248	−145.547 552	11.612	1565.91
$Ag_2Bi_2SnPb_3$	224	−145.537 894	11.613	1565.95
$Ag_2Bi_2SnPb_3$	385	−145.505 102	11.644	1578.63
$Ag_2Bi_2SnPb_3$	1625	−145.504 565	11.641	1577.54
$Ag_2Bi_2SnPb_3$	934	−145.315 856	11.613	1566.12
$Ag_2Bi_2SnPb_3$	377	−145.315 087	11.616	1567.34
$Ag_2Bi_2SnPb_3$	390	−145.300 906	11.614	1566.76
$Ag_2Bi_2SnPb_3$	1586	−145.266 951	11.637	1575.98
$Ag_2Bi_2SnPb_3$	218	−145.222 476	11.633	1574.19
$Ag_2Bi_2SnPb_3$	966	−145.215 594	11.629	1572.77
$Ag_2Bi_2SnPb_3$	1138	−145.181 280	11.626	1571.59
$Ag_2Bi_2SnPb_3$	664	−145.175 299	11.627	1572.00
$Ag_2Bi_2SnPb_3$	764	−145.166 937	11.609	1564.44
$Ag_2Bi_2SnPb_3$	894	−145.162 060	11.606	1563.19
$Ag_2Bi_2SnPb_3$	1647	−145.155 660	11.608	1564.32
$Ag_2Bi_2SnPb_3$	693	−145.141 255	11.610	1564.74
$Ag_2Bi_2SnPb_3$	1217	−145.124 926	11.608	1564.09
$Ag_2Bi_2SnPb_3$	179	−145.124 567	11.613	1566.19
$Ag_2Bi_2SnPb_3$	901	−144.978 866	11.619	1568.65
$Ag_2Bi_2SnPb_3$	1258	−144.970 719	11.635	1574.99
$Ag_2Bi_2Ge_2Sn_2$	1185	−145.867 275	11.439	1496.91
$Ag_2Bi_2Ge_2Sn_2$	404	−145.659 204	11.446	1499.71
$Ag_2Bi_2Ge_2Sn_2$	1827	−145.658 229	11.443	1498.25
$Ag_2Bi_2Ge_2Sn_2$	378	−145.588 982	11.432	1493.97

Continued on next page

Table S-9 – Continued from previous page

Metal composition	$N_{\text{Str.}}$	E_{tot}	a_0	V_0
$Ag_2Bi_2Ge_2Sn_2$	2425	−145.420 652	11.441	1497.58
$Ag_2Bi_2Ge_2Sn_2$	538	−145.344 110	11.452	1502.08
$Ag_2Bi_2Ge_2Sn_2$	291	−145.340 743	11.437	1496.17
$Ag_2Bi_2Ge_2Sn_2$	1325	−145.339 376	11.438	1496.40
$Ag_2Bi_2Ge_2Sn_2$	1479	−145.337 386	11.439	1496.75
$Ag_2Bi_2Ge_2Sn_2$	457	−145.336 962	11.439	1496.67
$Ag_2Bi_2Ge_2Sn_2$	1194	−145.336 928	11.437	1495.84
$Ag_2Bi_2Ge_2Sn_2$	992	−145.336 534	11.438	1496.44
$Ag_2Bi_2Ge_2Sn_2$	880	−145.277 441	11.446	1499.71
$Ag_2Bi_2Ge_2Sn_2$	181	−145.273 093	11.455	1503.27
$Ag_2Bi_2Ge_2Sn_2$	516	−145.269 143	11.434	1494.92
$Ag_2Bi_2Ge_2Sn_2$	411	−145.268 473	11.432	1494.22
$Ag_2Bi_2Ge_2Sn_2$	136	−145.241 004	11.422	1490.04
$Ag_2Bi_2Ge_2Sn_2$	1694	−145.237 926	11.419	1488.84
$Ag_2Bi_2Ge_2Sn_2$	2253	−145.210 329	11.437	1496.01
$Ag_2Bi_2Ge_2Sn_2$	473	−145.205 875	11.436	1495.43
$Ag_2Bi_2Ge_2Sn_2$	1888	−145.201 092	11.418	1488.71
$Ag_2Bi_2Ge_2Sn_2$	2407	−145.201 067	11.441	1497.74
$Ag_2Bi_2Ge_2Sn_2$	1792	−145.069 049	11.429	1492.71
$Ag_2Bi_2Ge_2Sn_2$	531	−145.051 030	11.422	1490.18
$Ag_2Bi_2Ge_2Sn_2$	1914	−145.004 384	11.423	1490.66
$Ag_2Bi_2Ge_2Sn_2$	1950	−144.999 431	11.426	1491.70
$Ag_2Bi_2Ge_2Sn_2$	372	−144.973 387	11.441	1497.64
$Ag_2Bi_2Ge_2Sn_2$	1906	−144.775 372	11.430	1493.25
$Ag_2Bi_2Ge_2Sn_2$	2340	−144.772 051	11.434	1494.73
$Ag_2Bi_2Ge_2Sn_2$	675	−144.749 400	11.429	1493.04
$Ag_2Bi_2Ge_2Pb_2$	1185	−145.915 708	11.480	1512.96
$Ag_2Bi_2Ge_2Pb_2$	378	−145.766 420	11.475	1510.96
$Ag_2Bi_2Ge_2Pb_2$	404	−145.723 523	11.504	1522.32
$Ag_2Bi_2Ge_2Pb_2$	1827	−145.723 047	11.501	1521.24
$Ag_2Bi_2Ge_2Pb_2$	538	−145.535 564	11.495	1518.98
$Ag_2Bi_2Ge_2Pb_2$	2425	−145.501 841	11.476	1511.19
$Ag_2Bi_2Ge_2Pb_2$	181	−145.481 171	11.503	1521.98
$Ag_2Bi_2Ge_2Pb_2$	880	−145.474 764	11.510	1524.98
$Ag_2Bi_2Ge_2Pb_2$	992	−145.464 783	11.482	1513.88
$Ag_2Bi_2Ge_2Pb_2$	2407	−145.460 618	11.503	1521.96

Continued on next page

Table S-9 – Continued from previous page

Metal composition	$N_{\mathrm{Str.}}$	E_{tot}	a_0	V_0
$Ag_2Bi_2Ge_2Pb_2$	291	−145.432 818	11.485	1514.77
$Ag_2Bi_2Ge_2Pb_2$	1479	−145.432 374	11.496	1519.15
$Ag_2Bi_2Ge_2Pb_2$	1325	−145.429 635	11.494	1518.41
$Ag_2Bi_2Ge_2Pb_2$	457	−145.428 146	11.486	1515.37
$Ag_2Bi_2Ge_2Pb_2$	1194	−145.427 343	11.486	1515.33
$Ag_2Bi_2Ge_2Pb_2$	473	−145.340 900	11.467	1507.71
$Ag_2Bi_2Ge_2Pb_2$	1950	−145.327 737	11.478	1512.10
$Ag_2Bi_2Ge_2Pb_2$	411	−145.323 055	11.496	1519.17
$Ag_2Bi_2Ge_2Pb_2$	516	−145.319 098	11.501	1521.27
$Ag_2Bi_2Ge_2Pb_2$	1694	−145.274 549	11.474	1510.51
$Ag_2Bi_2Ge_2Pb_2$	136	−145.265 433	11.466	1507.60
$Ag_2Bi_2Ge_2Pb_2$	2253	−145.249 786	11.466	1507.46
$Ag_2Bi_2Ge_2Pb_2$	1888	−145.221 987	11.476	1511.29
$Ag_2Bi_2Ge_2Pb_2$	531	−145.211 516	11.501	1521.42
$Ag_2Bi_2Ge_2Pb_2$	1792	−145.094 046	11.468	1508.18
$Ag_2Bi_2Ge_2Pb_2$	372	−145.029 847	11.482	1513.91
$Ag_2Bi_2Ge_2Pb_2$	1914	−144.981 612	11.478	1512.10
$Ag_2Bi_2Ge_2Pb_2$	2340	−144.900 403	11.480	1512.93
$Ag_2Bi_2Ge_2Pb_2$	1906	−144.882 157	11.488	1516.27
$Ag_2Bi_2Ge_2Pb_2$	675	−144.847 325	11.475	1511.08
$Ag_2Bi_2Sn_2Pb_2$	1185	−145.655 254	11.586	1555.43
$Ag_2Bi_2Sn_2Pb_2$	378	−145.610 699	11.590	1556.73
$Ag_2Bi_2Sn_2Pb_2$	404	−145.485 088	11.588	1556.05
$Ag_2Bi_2Sn_2Pb_2$	1827	−145.478 729	11.588	1556.21
$Ag_2Bi_2Sn_2Pb_2$	2425	−145.422 300	11.590	1557.01
$Ag_2Bi_2Sn_2Pb_2$	1888	−145.258 263	11.580	1552.76
$Ag_2Bi_2Sn_2Pb_2$	538	−145.240 988	11.609	1564.47
$Ag_2Bi_2Sn_2Pb_2$	1479	−145.178 044	11.572	1549.74
$Ag_2Bi_2Sn_2Pb_2$	880	−145.146 610	11.601	1561.25
$Ag_2Bi_2Sn_2Pb_2$	181	−145.144 910	11.605	1562.86
$Ag_2Bi_2Sn_2Pb_2$	457	−145.142 605	11.574	1550.24
$Ag_2Bi_2Sn_2Pb_2$	1950	−145.140 140	11.573	1549.95
$Ag_2Bi_2Sn_2Pb_2$	291	−145.138 113	11.576	1551.04
$Ag_2Bi_2Sn_2Pb_2$	992	−145.136 603	11.571	1549.36
$Ag_2Bi_2Sn_2Pb_2$	1194	−145.135 897	11.575	1550.84
$Ag_2Bi_2Sn_2Pb_2$	1325	−145.134 043	11.573	1549.85

Continued on next page

Table S-9 – Continued from previous page

Metal composition	$N_{\text{Str.}}$	E_{tot}	a_0	V_0
$Ag_2Bi_2Sn_2Pb_2$	531	−145.100 335	11.600	1561.05
$Ag_2Bi_2Sn_2Pb_2$	136	−145.098 633	11.586	1555.22
$Ag_2Bi_2Sn_2Pb_2$	1694	−145.098 460	11.583	1553.90
$Ag_2Bi_2Sn_2Pb_2$	516	−145.084 308	11.583	1553.92
$Ag_2Bi_2Sn_2Pb_2$	411	−145.082 356	11.581	1553.15
$Ag_2Bi_2Sn_2Pb_2$	2407	−144.987 867	11.585	1554.65
$Ag_2Bi_2Sn_2Pb_2$	473	−144.983 391	11.584	1554.35
$Ag_2Bi_2Sn_2Pb_2$	2253	−144.982 457	11.591	1557.10
$Ag_2Bi_2Sn_2Pb_2$	1792	−144.938 145	11.574	1550.49
$Ag_2Bi_2Sn_2Pb_2$	1914	−144.922 051	11.578	1551.84
$Ag_2Bi_2Sn_2Pb_2$	372	−144.803 985	11.598	1560.26
$Ag_2Bi_2Sn_2Pb_2$	675	−144.692 278	11.575	1550.83
$Ag_2Bi_2Sn_2Pb_2$	1906	−144.568 361	11.583	1554.15
$Ag_2Bi_2Sn_2Pb_2$	2340	−144.566 769	11.587	1555.67
$Ag_2Bi_2Ge_3Sn$	1394	−146.011 729	11.386	1476.05
$Ag_2Bi_2Ge_3Sn$	684	−145.884 390	11.386	1476.16
$Ag_2Bi_2Ge_3Sn$	556	−145.822 764	11.374	1471.58
$Ag_2Bi_2Ge_3Sn$	1304	−145.821 971	11.380	1473.69
$Ag_2Bi_2Ge_3Sn$	473	−145.817 270	11.378	1472.80
$Ag_2Bi_2Ge_3Sn$	325	−145.635 973	11.356	1464.40
$Ag_2Bi_2Ge_3Sn$	690	−145.634 165	11.355	1464.21
$Ag_2Bi_2Ge_3Sn$	1676	−145.606 105	11.379	1473.19
$Ag_2Bi_2Ge_3Sn$	911	−145.605 591	11.379	1473.26
$Ag_2Bi_2Ge_3Sn$	233	−145.552 203	11.396	1480.16
$Ag_2Bi_2Ge_3Sn$	1647	−145.530 202	11.383	1474.81
$Ag_2Bi_2Ge_3Sn$	34	−145.519 715	11.390	1477.48
$Ag_2Bi_2Ge_3Sn$	689	−145.416 366	11.393	1478.97
$Ag_2Bi_2Ge_3Sn$	186	−145.396 213	11.392	1478.40
$Ag_2Bi_2Ge_3Sn$	243	−145.279 710	11.370	1469.95
$Ag_2Bi_2Ge_3Sn$	1584	−145.279 444	11.371	1470.46
$Ag_2Bi_2Ge_3Sn$	682	−145.278 328	11.380	1473.91
$Ag_2Bi_2Ge_3Sn$	265	−145.274 863	11.413	1486.56
$Ag_2Bi_2Ge_3Sn$	346	−145.155 943	11.398	1480.59
$Ag_2Bi_2Ge_3Sn$	1640	−145.155 831	11.370	1469.69
$Ag_2Bi_2Ge_3Sn$	328	−145.153 494	11.398	1480.61
$Ag_2Bi_2Ge_3Sn$	86	−145.133 968	11.367	1468.90

Continued on next page

Table S-9 – Continued from previous page

Metal composition	$N_{\text{Str.}}$	E_{tot}	a_0	V_0
$Ag_2Bi_2Ge_3Sn$	977	−145.128 864	11.406	1483.69
$Ag_2Bi_2Ge_3Sn$	1380	−145.104 292	11.386	1476.13
$Ag_2Bi_2Ge_3Sn$	354	−145.103 955	11.384	1475.34
$Ag_2Bi_2Ge_3Sn$	87	−144.912 325	11.384	1475.20
$Ag_2Bi_2Ge_3Pb$	1394	−146.007 773	11.405	1483.53
$Ag_2Bi_2Ge_3Pb$	684	−145.964 693	11.411	1485.86
$Ag_2Bi_2Ge_3Pb$	473	−145.875 798	11.391	1477.97
$Ag_2Bi_2Ge_3Pb$	556	−145.875 182	11.386	1476.23
$Ag_2Bi_2Ge_3Pb$	1304	−145.874 107	11.394	1479.04
$Ag_2Bi_2Ge_3Pb$	325	−145.697 374	11.381	1474.07
$Ag_2Bi_2Ge_3Pb$	690	−145.691 056	11.377	1472.57
$Ag_2Bi_2Ge_3Pb$	1647	−145.671 612	11.408	1484.60
$Ag_2Bi_2Ge_3Pb$	911	−145.621 645	11.401	1481.88
$Ag_2Bi_2Ge_3Pb$	1676	−145.621 023	11.398	1480.72
$Ag_2Bi_2Ge_3Pb$	233	−145.558 855	11.403	1482.68
$Ag_2Bi_2Ge_3Pb$	34	−145.556 884	11.392	1478.55
$Ag_2Bi_2Ge_3Pb$	689	−145.444 768	11.424	1491.11
$Ag_2Bi_2Ge_3Pb$	265	−145.425 850	11.445	1499.15
$Ag_2Bi_2Ge_3Pb$	186	−145.418 797	11.430	1493.17
$Ag_2Bi_2Ge_3Pb$	243	−145.300 105	11.393	1478.98
$Ag_2Bi_2Ge_3Pb$	682	−145.298 548	11.400	1481.39
$Ag_2Bi_2Ge_3Pb$	1584	−145.297 815	11.398	1480.61
$Ag_2Bi_2Ge_3Pb$	1640	−145.189 842	11.401	1481.81
$Ag_2Bi_2Ge_3Pb$	328	−145.154 714	11.431	1493.83
$Ag_2Bi_2Ge_3Pb$	346	−145.153 238	11.435	1495.13
$Ag_2Bi_2Ge_3Pb$	977	−145.140 170	11.439	1496.62
$Ag_2Bi_2Ge_3Pb$	86	−145.127 144	11.385	1475.59
$Ag_2Bi_2Ge_3Pb$	1380	−145.118 537	11.408	1484.72
$Ag_2Bi_2Ge_3Pb$	354	−145.098 675	11.396	1479.80
$Ag_2Bi_2Ge_3Pb$	87	−144.951 316	11.422	1490.16
$Ag_2Bi_2Sn_3Pb$	1394	−145.578 870	11.560	1544.90
$Ag_2Bi_2Sn_3Pb$	473	−145.547 985	11.558	1543.94
$Ag_2Bi_2Sn_3Pb$	684	−145.478 158	11.557	1543.79
$Ag_2Bi_2Sn_3Pb$	1304	−145.442 334	11.560	1544.70
$Ag_2Bi_2Sn_3Pb$	556	−145.441 755	11.557	1543.48
$Ag_2Bi_2Sn_3Pb$	690	−145.250 973	11.566	1547.36

Continued on next page

Table S-9 – Continued from previous page

Metal composition	$N_{\mathrm{Str.}}$	E_{tot}	a_0	V_0
$Ag_2Bi_2Sn_3Pb$	325	−145.250 888	11.563	1545.92
$Ag_2Bi_2Sn_3Pb$	911	−145.216 597	11.568	1548.07
$Ag_2Bi_2Sn_3Pb$	1676	−145.210 584	11.561	1545.13
$Ag_2Bi_2Sn_3Pb$	1647	−145.194 692	11.566	1547.13
$Ag_2Bi_2Sn_3Pb$	689	−145.187 247	11.548	1539.83
$Ag_2Bi_2Sn_3Pb$	34	−145.157 316	11.592	1557.61
$Ag_2Bi_2Sn_3Pb$	233	−145.140 515	11.572	1549.62
$Ag_2Bi_2Sn_3Pb$	86	−145.021 759	11.549	1540.46
$Ag_2Bi_2Sn_3Pb$	186	−144.971 249	11.556	1543.38
$Ag_2Bi_2Sn_3Pb$	682	−144.919 456	11.544	1538.24
$Ag_2Bi_2Sn_3Pb$	1584	−144.913 620	11.547	1539.76
$Ag_2Bi_2Sn_3Pb$	243	−144.911 355	11.551	1541.35
$Ag_2Bi_2Sn_3Pb$	354	−144.900 563	11.564	1546.41
$Ag_2Bi_2Sn_3Pb$	1380	−144.895 752	11.563	1545.95
$Ag_2Bi_2Sn_3Pb$	1640	−144.875 157	11.567	1547.71
$Ag_2Bi_2Sn_3Pb$	265	−144.873 262	11.575	1550.97
$Ag_2Bi_2Sn_3Pb$	346	−144.851 946	11.564	1546.31
$Ag_2Bi_2Sn_3Pb$	977	−144.785 913	11.565	1546.62
$Ag_2Bi_2Sn_3Pb$	328	−144.742 459	11.560	1544.72
$Ag_2Bi_2Sn_3Pb$	87	−144.559 594	11.548	1539.96
$AgBiGeSn_5$	8	−147.308 722	11.580	1553.00
$AgBiGeSn_5$	53	−147.290 238	11.575	1551.02
$AgBiGeSn_5$	285	−147.136 218	11.573	1550.18
$AgBiGeSn_5$	186	−147.131 934	11.579	1552.34
$AgBiGeSn_5$	140	−147.127 088	11.573	1549.91
$AgBiGeSn_5$	196	−147.073 656	11.566	1547.11
$AgBiGeSn_5$	191	−147.071 821	11.584	1554.47
$AgBiGeSn_5$	146	−147.070 232	11.578	1552.08
$AgBiGeSn_5$	327	−147.064 875	11.566	1547.23
$AgBiGePb_5$	285	−147.575 013	11.679	1593.13
$AgBiGePb_5$	186	−147.572 449	11.692	1598.31
$AgBiGePb_5$	53	−147.540 639	11.684	1594.99
$AgBiGePb_5$	8	−147.538 387	11.690	1597.45
$AgBiGePb_5$	196	−147.525 239	11.699	1601.29
$AgBiGePb_5$	140	−147.380 181	11.683	1594.57
$AgBiGePb_5$	327	−147.311 833	11.697	1600.35

Continued on next page

Table S-9 – Continued from previous page

Metal composition	$N_{\text{Str.}}$	E_{tot}	a_0	V_0
$AgBiGePb_5$	146	−147.305 079	11.700	1601.45
$AgBiGePb_5$	191	−147.303 410	11.694	1599.35
$AgBiSnPb_5$	53	−147.673 676	11.726	1612.11
$AgBiSnPb_5$	8	−147.573 830	11.728	1613.03
$AgBiSnPb_5$	327	−147.496 750	11.732	1614.91
$AgBiSnPb_5$	186	−147.472 623	11.728	1613.22
$AgBiSnPb_5$	196	−147.465 574	11.735	1616.12
$AgBiSnPb_5$	191	−147.309 265	11.731	1614.39
$AgBiSnPb_5$	140	−147.284 753	11.733	1615.37
$AgBiSnPb_5$	285	−147.284 183	11.727	1612.72
$AgBiSnPb_5$	146	−147.275 152	11.740	1618.09
$AgBiGe_2Sn_4$	411	−147.505 338	11.523	1530.15
$AgBiGe_2Sn_4$	57	−147.455 170	11.527	1531.55
$AgBiGe_2Sn_4$	559	−147.433 158	11.513	1526.14
$AgBiGe_2Sn_4$	747	−147.285 594	11.521	1529.25
$AgBiGe_2Sn_4$	788	−147.268 948	11.516	1527.16
$AgBiGe_2Sn_4$	112	−147.252 632	11.521	1529.08
$AgBiGe_2Sn_4$	706	−147.236 236	11.515	1526.96
$AgBiGe_2Sn_4$	171	−147.231 831	11.511	1525.38
$AgBiGe_2Sn_4$	335	−147.206 163	11.506	1523.25
$AgBiGe_2Sn_4$	86	−147.201 466	11.509	1524.46
$AgBiGe_2Sn_4$	398	−147.163 390	11.509	1524.60
$AgBiGe_2Pb_4$	747	−147.678 043	11.613	1565.98
$AgBiGe_2Pb_4$	411	−147.675 704	11.611	1565.30
$AgBiGe_2Pb_4$	788	−147.665 694	11.615	1567.11
$AgBiGe_2Pb_4$	335	−147.613 676	11.602	1561.71
$AgBiGe_2Pb_4$	171	−147.609 165	11.594	1558.39
$AgBiGe_2Pb_4$	57	−147.604 537	11.619	1568.58
$AgBiGe_2Pb_4$	559	−147.577 250	11.590	1557.01
$AgBiGe_2Pb_4$	398	−147.565 302	11.613	1565.96
$AgBiGe_2Pb_4$	112	−147.423 329	11.623	1570.38
$AgBiGe_2Pb_4$	706	−147.398 085	11.599	1560.48
$AgBiGe_2Pb_4$	86	−147.372 655	11.601	1561.50
$AgBiSn_2Pb_4$	411	−147.420 846	11.711	1606.02
$AgBiSn_2Pb_4$	559	−147.419 899	11.705	1603.58
$AgBiSn_2Pb_4$	57	−147.401 705	11.706	1604.02

Continued on next page

Table S-9 – Continued from previous page

Metal composition	$N_{\text{Str.}}$	E_{tot}	a_0	V_0
$AgBiSn_2Pb_4$	171	−147.361 497	11.713	1606.84
$AgBiSn_2Pb_4$	86	−147.328 578	11.707	1604.48
$AgBiSn_2Pb_4$	335	−147.306 230	11.713	1606.82
$AgBiSn_2Pb_4$	706	−147.248 963	11.712	1606.49
$AgBiSn_2Pb_4$	788	−147.216 248	11.707	1604.29
$AgBiSn_2Pb_4$	398	−147.209 557	11.710	1605.87
$AgBiSn_2Pb_4$	112	−147.207 946	11.708	1604.93
$AgBiSn_2Pb_4$	747	−147.203 913	11.713	1606.97
$AgBiGe_3Sn_3$	114	−147.666 178	11.487	1515.58
$AgBiGe_3Sn_3$	279	−147.662 926	11.479	1512.64
$AgBiGe_3Sn_3$	138	−147.639 661	11.485	1514.91
$AgBiGe_3Sn_3$	821	−147.592 656	11.480	1513.08
$AgBiGe_3Sn_3$	540	−147.579 728	11.469	1508.47
$AgBiGe_3Sn_3$	20	−147.570 988	11.467	1507.87
$AgBiGe_3Sn_3$	1083	−147.449 880	11.468	1508.24
$AgBiGe_3Sn_3$	473	−147.400 386	11.472	1509.60
$AgBiGe_3Sn_3$	383	−147.379 900	11.468	1508.21
$AgBiGe_3Sn_3$	14	−147.367 274	11.465	1507.11
$AgBiGe_3Sn_3$	956	−147.362 058	11.469	1508.57
$AgBiGe_3Sn_3$	88	−147.351 086	11.461	1505.53
$AgBiGe_3Sn_3$	754	−147.319 474	11.463	1506.26
$AgBiGe_3Pb_3$	279	−147.798 303	11.535	1534.74
$AgBiGe_3Pb_3$	114	−147.792 347	11.532	1533.70
$AgBiGe_3Pb_3$	138	−147.780 272	11.524	1530.58
$AgBiGe_3Pb_3$	540	−147.749 250	11.517	1527.76
$AgBiGe_3Pb_3$	821	−147.682 621	11.557	1543.49
$AgBiGe_3Pb_3$	20	−147.679 876	11.524	1530.61
$AgBiGe_3Pb_3$	1083	−147.568 382	11.513	1525.87
$AgBiGe_3Pb_3$	14	−147.531 396	11.529	1532.44
$AgBiGe_3Pb_3$	473	−147.495 143	11.518	1528.02
$AgBiGe_3Pb_3$	754	−147.488 007	11.512	1525.72
$AgBiGe_3Pb_3$	383	−147.487 335	11.518	1528.19
$AgBiGe_3Pb_3$	956	−147.447 771	11.523	1530.13
$AgBiGe_3Pb_3$	88	−147.431 717	11.506	1523.28
$AgBiSn_3Pb_3$	279	−147.361 932	11.684	1595.07
$AgBiSn_3Pb_3$	114	−147.354 283	11.692	1598.30

Continued on next page

Table S-9 – Continued from previous page

Metal composition	$N_{\text{Str.}}$	E_{tot}	a_0	V_0
$AgBiSn_3Pb_3$	540	−147.350 530	11.690	1597.55
$AgBiSn_3Pb_3$	20	−147.347 158	11.694	1599.06
$AgBiSn_3Pb_3$	821	−147.331 698	11.684	1594.99
$AgBiSn_3Pb_3$	138	−147.328 681	11.684	1594.98
$AgBiSn_3Pb_3$	14	−147.272 797	11.696	1600.07
$AgBiSn_3Pb_3$	754	−147.177 498	11.687	1596.22
$AgBiSn_3Pb_3$	1083	−147.166 109	11.694	1599.14
$AgBiSn_3Pb_3$	956	−147.150 390	11.692	1598.22
$AgBiSn_3Pb_3$	383	−147.146 868	11.694	1599.08
$AgBiSn_3Pb_3$	473	−147.139 013	11.694	1599.31
$AgBiSn_3Pb_3$	88	−147.107 321	11.697	1600.42
$AgBiGe_4Sn_2$	19	−147.854 411	11.432	1494.25
$AgBiGe_4Sn_2$	701	−147.820 494	11.409	1484.92
$AgBiGe_4Sn_2$	272	−147.814 351	11.451	1501.49
$AgBiGe_4Sn_2$	515	−147.812 206	11.449	1500.74
$AgBiGe_4Sn_2$	171	−147.622 488	11.454	1502.61
$AgBiGe_4Sn_2$	304	−147.613 766	11.449	1500.68
$AgBiGe_4Sn_2$	75	−147.613 057	11.439	1496.99
$AgBiGe_4Sn_2$	582	−147.606 953	11.439	1496.95
$AgBiGe_4Sn_2$	33	−147.600 575	11.439	1496.74
$AgBiGe_4Sn_2$	202	−147.598 687	11.425	1491.38
$AgBiGe_4Sn_2$	236	−147.571 447	11.416	1487.81
$AgBiGe_4Sn_2$	668	−147.545 680	11.425	1491.40
$AgBiGe_4Sn_2$	361	−147.545 311	11.429	1492.88
$AgBiGe_4Sn_2$	199	−147.543 159	11.424	1491.03
$AgBiGe_4Sn_2$	103	−147.538 129	11.422	1490.29
$AgBiGe_4Sn_2$	623	−147.526 119	11.424	1490.94
$AgBiGe_4Pb_2$	19	−147.982 340	11.462	1505.74
$AgBiGe_4Pb_2$	701	−147.905 105	11.466	1507.35
$AgBiGe_4Pb_2$	515	−147.866 507	11.494	1518.59
$AgBiGe_4Pb_2$	272	−147.865 750	11.501	1521.47
$AgBiGe_4Pb_2$	75	−147.698 155	11.472	1509.71
$AgBiGe_4Pb_2$	304	−147.694 063	11.500	1520.87
$AgBiGe_4Pb_2$	171	−147.677 917	11.501	1521.27
$AgBiGe_4Pb_2$	202	−147.674 892	11.455	1503.22
$AgBiGe_4Pb_2$	582	−147.659 921	11.468	1508.04

Continued on next page

Table S-9 – Continued from previous page

Metal composition	$N_{\mathrm{Str.}}$	E_{tot}	a_0	V_0
$AgBiGe_4Pb_2$	33	−147.658 321	11.469	1508.69
$AgBiGe_4Pb_2$	236	−147.649 752	11.458	1504.20
$AgBiGe_4Pb_2$	668	−147.599 196	11.464	1506.69
$AgBiGe_4Pb_2$	361	−147.599 173	11.463	1506.43
$AgBiGe_4Pb_2$	199	−147.597 108	11.461	1505.62
$AgBiGe_4Pb_2$	103	−147.595 907	11.459	1504.66
$AgBiGe_4Pb_2$	623	−147.571 532	11.461	1505.62
$AgBiSn_4Pb_2$	19	−147.320 549	11.661	1585.58
$AgBiSn_4Pb_2$	701	−147.287 858	11.668	1588.57
$AgBiSn_4Pb_2$	515	−147.281 613	11.665	1587.46
$AgBiSn_4Pb_2$	272	−147.278 075	11.660	1585.37
$AgBiSn_4Pb_2$	33	−147.201 646	11.666	1587.66
$AgBiSn_4Pb_2$	75	−147.121 141	11.669	1588.87
$AgBiSn_4Pb_2$	582	−147.113 459	11.662	1586.12
$AgBiSn_4Pb_2$	361	−147.103 054	11.664	1586.99
$AgBiSn_4Pb_2$	171	−147.100 686	11.665	1587.22
$AgBiSn_4Pb_2$	236	−147.098 625	11.667	1588.29
$AgBiSn_4Pb_2$	202	−147.097 239	11.676	1591.91
$AgBiSn_4Pb_2$	304	−147.095 089	11.674	1591.09
$AgBiSn_4Pb_2$	623	−147.085 629	11.673	1590.38
$AgBiSn_4Pb_2$	668	−147.084 258	11.661	1585.45
$AgBiSn_4Pb_2$	199	−147.082 944	11.664	1586.87
$AgBiSn_4Pb_2$	103	−147.076 083	11.662	1586.07
$AgBiGe_5Sn$	45	−147.980 873	11.384	1475.32
$AgBiGe_5Sn$	2	−147.971 030	11.351	1462.65
$AgBiGe_5Sn$	173	−147.968 948	11.369	1469.45
$AgBiGe_5Sn$	247	−147.791 935	11.393	1478.64
$AgBiGe_5Sn$	329	−147.789 627	11.393	1478.75
$AgBiGe_5Sn$	5	−147.789 551	11.394	1479.28
$AgBiGe_5Sn$	294	−147.786 102	11.393	1478.83
$AgBiGe_5Sn$	60	−147.776 918	11.393	1478.63
$AgBiGe_5Sn$	306	−147.747 725	11.362	1466.76
$AgBiGe_5Sn$	312	−147.745 226	11.376	1472.07
$AgBiGe_5Sn$	61	−147.737 747	11.374	1471.28
$AgBiGe_5Sn$	13	−147.726 606	11.381	1474.10
$AgBiGe_5Pb$	2	−148.018 624	11.392	1478.30

Continued on next page

Table S-9 – Continued from previous page

Metal composition	$N_{\text{Str.}}$	E_{tot}	a_0	V_0
$AgBiGe_5Pb$	173	−148.007 603	11.398	1480.79
$AgBiGe_5Pb$	45	−148.004 791	11.397	1480.22
$AgBiGe_5Pb$	247	−147.809 378	11.420	1489.33
$AgBiGe_5Pb$	60	−147.808 746	11.423	1490.49
$AgBiGe_5Pb$	5	−147.804 069	11.425	1491.15
$AgBiGe_5Pb$	294	−147.801 468	11.416	1487.71
$AgBiGe_5Pb$	329	−147.797 058	11.420	1489.26
$AgBiGe_5Pb$	61	−147.795 933	11.395	1479.49
$AgBiGe_5Pb$	312	−147.792 235	11.395	1479.56
$AgBiGe_5Pb$	306	−147.780 105	11.380	1473.76
$AgBiGe_5Pb$	13	−147.735 925	11.401	1481.93
$AgBiSn_5Pb$	2	−147.249 324	11.634	1574.55
$AgBiSn_5Pb$	173	−147.211 925	11.639	1576.68
$AgBiSn_5Pb$	45	−147.205 588	11.637	1575.84
$AgBiSn_5Pb$	247	−147.048 900	11.637	1575.81
$AgBiSn_5Pb$	312	−147.036 752	11.649	1580.60
$AgBiSn_5Pb$	294	−147.035 316	11.639	1576.68
$AgBiSn_5Pb$	60	−147.033 732	11.640	1577.15
$AgBiSn_5Pb$	329	−147.030 230	11.640	1577.08
$AgBiSn_5Pb$	5	−147.029 359	11.643	1578.49
$AgBiSn_5Pb$	306	−147.024 032	11.639	1576.52
$AgBiSn_5Pb$	61	−147.019 960	11.649	1580.63
$AgBiSn_5Pb$	13	−146.998 949	11.654	1582.64

Table S-10. Relative total energy (ΔE_{tot}), relative equilibrium lattice parameter (Δa_0), and relative equilibrium volume (ΔV_0) of all optimized structures given in eV, Å, and Å^3, respectively. Having as reference the lowest-energy structure configuration, each relative property of structure i was calculated through: $\Delta E^i_{tot} = E^i_{tot} - E^{lowest}_{tot}$, $\Delta a^i_0 = a^i_0 - a^{lowest}_0$, and $\Delta V^i_0 = V^i_0 - V^{lowest}_0$. $N_{\text{Str.}}$ refers to "structure number", which follows the enumeration given in the permutation process. The subscripts in the metal composition column indicate the number of atoms (of that species) within the unit-cell.

Metal composition	$N_{\text{Str.}}$	ΔE^i_{tot}	Δa^i_0	ΔV^i_0
Ag_4Bi_4	10	0.00	0.00	0.00
Ag_4Bi_4	28	32.34	0.03	0.33
Ag_4Bi_4	45	32.47	0.03	0.31
Ag_4Bi_4	37	34.26	0.03	0.32
Ag_4Bi_4	46	47.74	0.06	0.60
Ag_4Bi_4	38	48.18	0.06	0.63
Ag_4Bi_4	50	48.45	0.06	0.58
Ag_4Bi_4	61	49.89	0.06	0.58
Ag_4Bi_4	17	50.04	0.06	0.63
Ag_4Bi_4	58	66.13	0.08	0.82
$Ag_3Bi_3Ge_2$	338	0.00	0.00	0.00
$Ag_3Bi_3Ge_2$	500	5.96	0.00	0.00
$Ag_3Bi_3Ge_2$	491	12.33	−0.01	−0.09
$Ag_3Bi_3Ge_2$	411	15.91	−0.01	−0.12
$Ag_3Bi_3Ge_2$	475	16.13	0.02	0.17
$Ag_3Bi_3Ge_2$	280	20.09	0.02	0.23
$Ag_3Bi_3Ge_2$	555	22.07	0.01	0.07
$Ag_3Bi_3Ge_2$	192	22.08	0.01	0.06
$Ag_3Bi_3Ge_2$	161	22.14	0.01	0.05
$Ag_3Bi_3Ge_2$	466	26.29	−0.01	−0.11
$Ag_3Bi_3Ge_2$	155	26.57	−0.01	−0.13
$Ag_3Bi_3Ge_2$	363	27.13	0.01	0.12
$Ag_3Bi_3Ge_2$	14	27.56	0.01	0.13
$Ag_3Bi_3Ge_2$	55	30.53	0.02	0.17
$Ag_3Bi_3Ge_2$	243	32.84	0.01	0.10
$Ag_3Bi_3Ge_2$	261	34.26	0.03	0.33
$Ag_3Bi_3Ge_2$	539	34.59	0.03	0.29
$Ag_3Bi_3Ge_2$	283	35.95	0.03	0.31
$Ag_3Bi_3Ge_2$	360	38.38	0.01	0.11
$Ag_3Bi_3Ge_2$	490	41.20	0.02	0.17

Continued on next page

Table S-10 – Continued from previous page

Metal composition	$N_{\text{Str.}}$	ΔE^i_{tot}	Δa^i_0	ΔV^i_0
$Ag_3Bi_3Ge_2$	149	41.41	0.02	0.19
$Ag_3Bi_3Sn_2$	338	0.00	0.00	0.00
$Ag_3Bi_3Sn_2$	500	3.13	0.00	0.01
$Ag_3Bi_3Sn_2$	411	12.39	0.00	−0.01
$Ag_3Bi_3Sn_2$	491	12.98	0.01	0.13
$Ag_3Bi_3Sn_2$	475	16.11	0.01	0.06
$Ag_3Bi_3Sn_2$	280	19.43	0.03	0.29
$Ag_3Bi_3Sn_2$	466	19.92	−0.01	−0.11
$Ag_3Bi_3Sn_2$	155	19.98	−0.01	−0.12
$Ag_3Bi_3Sn_2$	192	20.06	0.01	0.15
$Ag_3Bi_3Sn_2$	161	20.11	0.01	0.12
$Ag_3Bi_3Sn_2$	555	20.20	0.01	0.13
$Ag_3Bi_3Sn_2$	14	24.76	0.00	−0.01
$Ag_3Bi_3Sn_2$	363	24.81	0.00	−0.03
$Ag_3Bi_3Sn_2$	55	25.75	0.02	0.17
$Ag_3Bi_3Sn_2$	243	28.74	0.01	0.07
$Ag_3Bi_3Sn_2$	539	32.68	0.01	0.10
$Ag_3Bi_3Sn_2$	261	32.70	0.01	0.13
$Ag_3Bi_3Sn_2$	283	35.43	0.00	0.05
$Ag_3Bi_3Sn_2$	360	35.83	0.01	0.10
$Ag_3Bi_3Sn_2$	490	39.18	0.03	0.26
$Ag_3Bi_3Sn_2$	149	39.49	0.01	0.11
$Ag_3Bi_3Pb_2$	338	0.00	0.00	0.00
$Ag_3Bi_3Pb_2$	500	1.42	0.00	0.02
$Ag_3Bi_3Pb_2$	411	10.84	0.00	−0.01
$Ag_3Bi_3Pb_2$	280	12.60	0.03	0.28
$Ag_3Bi_3Pb_2$	491	13.70	0.01	0.09
$Ag_3Bi_3Pb_2$	475	16.08	0.01	0.11
$Ag_3Bi_3Pb_2$	555	19.09	0.01	0.10
$Ag_3Bi_3Pb_2$	192	19.26	0.02	0.15
$Ag_3Bi_3Pb_2$	161	19.31	0.01	0.07
$Ag_3Bi_3Pb_2$	155	20.76	−0.01	−0.06
$Ag_3Bi_3Pb_2$	466	20.79	0.00	−0.03
$Ag_3Bi_3Pb_2$	363	26.69	0.00	0.05
$Ag_3Bi_3Pb_2$	14	26.86	0.01	0.06
$Ag_3Bi_3Pb_2$	261	28.17	0.01	0.10

Continued on next page

Table S-10 – Continued from previous page

Metal composition	$N_{Str.}$	ΔE^i_{tot}	Δa^i_0	ΔV^i_0
$Ag_3Bi_3Pb_2$	243	29.03	0.01	0.10
$Ag_3Bi_3Pb_2$	55	29.47	0.01	0.12
$Ag_3Bi_3Pb_2$	149	34.09	0.02	0.16
$Ag_3Bi_3Pb_2$	539	34.90	0.02	0.24
$Ag_3Bi_3Pb_2$	360	35.64	0.01	0.07
$Ag_3Bi_3Pb_2$	283	35.90	0.02	0.17
$Ag_3Bi_3Pb_2$	490	39.47	0.02	0.17
$Ag_2Bi_2Ge_4$	50	0.00	0.00	0.00
$Ag_2Bi_2Ge_4$	83	0.86	0.00	−0.04
$Ag_2Bi_2Ge_4$	339	2.36	0.04	0.36
$Ag_2Bi_2Ge_4$	112	2.54	0.04	0.34
$Ag_2Bi_2Ge_4$	391	6.50	0.01	0.14
$Ag_2Bi_2Ge_4$	406	6.51	0.02	0.18
$Ag_2Bi_2Ge_4$	143	6.53	0.02	0.20
$Ag_2Bi_2Ge_4$	49	9.96	0.02	0.17
$Ag_2Bi_2Ge_4$	128	10.00	0.02	0.19
$Ag_2Bi_2Ge_4$	237	10.04	0.02	0.17
$Ag_2Bi_2Ge_4$	270	12.08	0.01	0.08
$Ag_2Bi_2Ge_4$	97	12.10	0.01	0.05
$Ag_2Bi_2Ge_4$	172	13.64	0.02	0.21
$Ag_2Bi_2Ge_4$	311	19.24	0.02	0.16
$Ag_2Bi_2Ge_4$	225	19.34	0.02	0.15
$Ag_2Bi_2Sn_4$	50	0.00	0.00	0.00
$Ag_2Bi_2Sn_4$	83	1.12	0.01	0.14
$Ag_2Bi_2Sn_4$	112	2.28	0.03	0.25
$Ag_2Bi_2Sn_4$	339	3.03	0.04	0.39
$Ag_2Bi_2Sn_4$	143	5.64	0.00	−0.03
$Ag_2Bi_2Sn_4$	406	5.75	−0.01	−0.05
$Ag_2Bi_2Sn_4$	391	5.87	0.00	−0.01
$Ag_2Bi_2Sn_4$	237	7.57	0.01	0.11
$Ag_2Bi_2Sn_4$	270	8.05	−0.01	−0.07
$Ag_2Bi_2Sn_4$	49	8.64	0.02	0.20
$Ag_2Bi_2Sn_4$	97	8.90	−0.01	−0.09
$Ag_2Bi_2Sn_4$	128	9.85	0.01	0.12
$Ag_2Bi_2Sn_4$	172	12.14	0.00	−0.03
$Ag_2Bi_2Sn_4$	225	16.31	−0.01	−0.06

Continued on next page

Table S-10 – Continued from previous page

Metal composition	$N_{\text{Str.}}$	ΔE^i_{tot}	Δa^i_0	ΔV^i_0
$Ag_2Bi_2Sn_4$	311	16.79	0.00	−0.04
$Ag_2Bi_2Pb_4$	339	0.00	0.00	0.00
$Ag_2Bi_2Pb_4$	50	3.18	−0.03	−0.31
$Ag_2Bi_2Pb_4$	83	5.14	−0.03	−0.28
$Ag_2Bi_2Pb_4$	112	6.26	0.01	0.07
$Ag_2Bi_2Pb_4$	128	6.29	−0.01	−0.10
$Ag_2Bi_2Pb_4$	49	6.79	−0.01	−0.11
$Ag_2Bi_2Pb_4$	391	10.64	−0.02	−0.21
$Ag_2Bi_2Pb_4$	406	10.71	−0.02	−0.20
$Ag_2Bi_2Pb_4$	143	10.71	−0.03	−0.26
$Ag_2Bi_2Pb_4$	237	13.24	−0.01	−0.12
$Ag_2Bi_2Pb_4$	97	14.40	−0.03	−0.35
$Ag_2Bi_2Pb_4$	270	14.55	−0.03	−0.35
$Ag_2Bi_2Pb_4$	225	17.77	−0.03	−0.30
$Ag_2Bi_2Pb_4$	172	18.39	−0.01	−0.15
$Ag_2Bi_2Pb_4$	311	19.84	−0.02	−0.22
$AgBiGe_6$	40	0.00	0.00	0.00
$AgBiGe_6$	12	6.11	−0.01	−0.13
$AgBiGe_6$	24	6.66	−0.01	−0.06
$AgBiSn_6$	40	0.00	0.00	0.00
$AgBiSn_6$	12	5.07	0.00	0.04
$AgBiSn_6$	24	5.16	0.00	−0.01
$AgBiPb_6$	40	0.00	0.00	0.00
$AgBiPb_6$	12	6.05	0.01	0.14
$AgBiPb_6$	24	7.20	0.03	0.28
Ag_3Bi_3GeSn	474	0.00	0.00	0.00
Ag_3Bi_3GeSn	1075	4.35	0.02	0.21
Ag_3Bi_3GeSn	1068	14.52	0.02	0.20
Ag_3Bi_3GeSn	499	15.71	0.01	0.07
Ag_3Bi_3GeSn	1112	15.71	0.01	0.13
Ag_3Bi_3GeSn	426	15.78	0.01	0.07
Ag_3Bi_3GeSn	466	15.79	0.01	0.09
Ag_3Bi_3GeSn	379	18.66	0.03	0.30
Ag_3Bi_3GeSn	1096	20.82	0.05	0.54
Ag_3Bi_3GeSn	626	22.33	0.02	0.21
Ag_3Bi_3GeSn	1045	22.92	0.03	0.33

Continued on next page

Table S-10 – Continued from previous page

Metal composition	$N_{\text{Str.}}$	ΔE^i_{tot}	Δa^i_0	ΔV^i_0
Ag_3Bi_3GeSn	922	24.22	0.02	0.20
Ag_3Bi_3GeSn	658	24.95	0.05	0.49
Ag_3Bi_3GeSn	284	25.06	0.05	0.48
Ag_3Bi_3GeSn	528	25.60	0.01	0.08
Ag_3Bi_3GeSn	330	26.02	0.05	0.47
Ag_3Bi_3GeSn	20	28.65	0.02	0.19
Ag_3Bi_3GeSn	734	28.78	0.01	0.12
Ag_3Bi_3GeSn	761	28.86	0.02	0.15
Ag_3Bi_3GeSn	889	28.88	0.02	0.15
Ag_3Bi_3GeSn	276	28.99	0.01	0.14
Ag_3Bi_3GeSn	972	31.29	0.04	0.41
Ag_3Bi_3GeSn	1057	32.01	0.03	0.27
Ag_3Bi_3GeSn	533	32.30	0.03	0.28
Ag_3Bi_3GeSn	509	35.37	0.04	0.37
Ag_3Bi_3GeSn	692	35.46	0.04	0.43
Ag_3Bi_3GeSn	335	41.55	0.05	0.47
Ag_3Bi_3GeSn	1024	41.96	0.04	0.40
Ag_3Bi_3GeSn	171	42.17	0.04	0.37
Ag_3Bi_3GePb	474	0.00	0.00	0.00
Ag_3Bi_3GePb	1075	4.00	0.01	0.14
Ag_3Bi_3GePb	1068	16.64	0.02	0.17
Ag_3Bi_3GePb	1112	16.90	0.01	0.11
Ag_3Bi_3GePb	426	16.95	0.01	0.13
Ag_3Bi_3GePb	466	16.96	0.01	0.10
Ag_3Bi_3GePb	499	17.08	0.01	0.10
Ag_3Bi_3GePb	1096	19.66	0.06	0.58
Ag_3Bi_3GePb	379	20.13	0.02	0.21
Ag_3Bi_3GePb	626	23.36	0.02	0.20
Ag_3Bi_3GePb	658	23.41	0.04	0.36
Ag_3Bi_3GePb	1045	24.74	0.03	0.25
Ag_3Bi_3GePb	330	24.95	0.03	0.32
Ag_3Bi_3GePb	528	25.15	0.01	0.07
Ag_3Bi_3GePb	922	26.45	0.02	0.21
Ag_3Bi_3GePb	284	26.46	0.04	0.38
Ag_3Bi_3GePb	20	30.52	0.02	0.18
Ag_3Bi_3GePb	276	30.55	0.02	0.18

Continued on next page

Table S-10 – Continued from previous page

Metal composition	$N_{\text{Str.}}$	ΔE^i_{tot}	Δa^i_0	ΔV^i_0
Ag_3Bi_3GePb	889	31.07	0.02	0.16
Ag_3Bi_3GePb	734	31.10	0.02	0.16
Ag_3Bi_3GePb	761	31.16	0.02	0.18
Ag_3Bi_3GePb	972	33.11	0.04	0.39
Ag_3Bi_3GePb	533	33.75	0.03	0.27
Ag_3Bi_3GePb	1057	34.07	0.03	0.29
Ag_3Bi_3GePb	509	35.26	0.03	0.30
Ag_3Bi_3GePb	335	35.98	0.04	0.38
Ag_3Bi_3GePb	1024	36.90	0.04	0.36
Ag_3Bi_3GePb	692	37.80	0.04	0.34
Ag_3Bi_3GePb	171	43.68	0.03	0.28
Ag_3Bi_3SnPb	474	0.00	0.00	0.00
Ag_3Bi_3SnPb	1075	2.25	0.01	0.05
Ag_3Bi_3SnPb	499	12.97	0.00	0.04
Ag_3Bi_3SnPb	1112	13.03	0.01	0.08
Ag_3Bi_3SnPb	426	13.06	0.00	0.04
Ag_3Bi_3SnPb	466	13.10	0.00	0.03
Ag_3Bi_3SnPb	1068	14.52	0.02	0.20
Ag_3Bi_3SnPb	1096	15.58	0.03	0.33
Ag_3Bi_3SnPb	379	17.51	0.03	0.26
Ag_3Bi_3SnPb	658	19.34	0.04	0.36
Ag_3Bi_3SnPb	284	19.35	0.04	0.36
Ag_3Bi_3SnPb	1045	21.45	0.03	0.28
Ag_3Bi_3SnPb	626	21.51	0.02	0.17
Ag_3Bi_3SnPb	528	21.74	−0.01	−0.10
Ag_3Bi_3SnPb	922	22.47	0.02	0.17
Ag_3Bi_3SnPb	972	25.25	0.03	0.31
Ag_3Bi_3SnPb	330	25.37	0.03	0.33
Ag_3Bi_3SnPb	889	27.54	0.00	0.04
Ag_3Bi_3SnPb	20	27.57	0.01	0.08
Ag_3Bi_3SnPb	761	27.58	0.00	0.04
Ag_3Bi_3SnPb	276	27.63	0.01	0.07
Ag_3Bi_3SnPb	734	27.69	0.00	0.03
Ag_3Bi_3SnPb	1057	30.12	0.03	0.28
Ag_3Bi_3SnPb	533	30.28	0.03	0.27
Ag_3Bi_3SnPb	692	30.64	0.03	0.30

Continued on next page

Table S-10 – Continued from previous page

Metal composition	$N_{\mathrm{Str.}}$	ΔE^{i}_{tot}	Δa^{i}_{0}	ΔV^{i}_{0}
Ag_3Bi_3SnPb	509	31.01	0.03	0.32
Ag_3Bi_3SnPb	335	34.53	0.04	0.38
Ag_3Bi_3SnPb	1024	35.59	0.03	0.32
Ag_3Bi_3SnPb	171	40.66	0.03	0.33
$Ag_2Bi_2GeSn_3$	649	0.00	0.00	0.00
$Ag_2Bi_2GeSn_3$	84	4.95	−0.01	−0.09
$Ag_2Bi_2GeSn_3$	224	6.18	0.00	0.04
$Ag_2Bi_2GeSn_3$	1248	6.29	0.01	0.09
$Ag_2Bi_2GeSn_3$	1664	6.32	0.01	0.05
$Ag_2Bi_2GeSn_3$	532	6.38	0.01	0.06
$Ag_2Bi_2GeSn_3$	390	8.89	0.00	0.02
$Ag_2Bi_2GeSn_3$	377	10.67	0.00	−0.01
$Ag_2Bi_2GeSn_3$	934	11.86	0.00	0.00
$Ag_2Bi_2GeSn_3$	1586	11.99	0.03	0.26
$Ag_2Bi_2GeSn_3$	764	13.11	−0.01	−0.08
$Ag_2Bi_2GeSn_3$	894	13.22	0.00	−0.04
$Ag_2Bi_2GeSn_3$	1625	13.35	0.02	0.17
$Ag_2Bi_2GeSn_3$	385	13.46	0.02	0.16
$Ag_2Bi_2GeSn_3$	179	16.06	−0.01	−0.12
$Ag_2Bi_2GeSn_3$	1217	16.13	−0.02	−0.16
$Ag_2Bi_2GeSn_3$	218	18.38	0.02	0.22
$Ag_2Bi_2GeSn_3$	966	18.71	0.00	−0.03
$Ag_2Bi_2GeSn_3$	1258	19.00	0.02	0.23
$Ag_2Bi_2GeSn_3$	693	20.45	−0.02	−0.16
$Ag_2Bi_2GeSn_3$	1138	20.48	0.01	0.10
$Ag_2Bi_2GeSn_3$	1647	20.53	−0.02	−0.15
$Ag_2Bi_2GeSn_3$	664	20.56	0.01	0.10
$Ag_2Bi_2GeSn_3$	901	22.27	0.01	0.07
$Ag_2Bi_2GePb_3$	649	0.00	0.00	0.00
$Ag_2Bi_2GePb_3$	84	3.41	0.00	0.04
$Ag_2Bi_2GePb_3$	224	5.77	0.02	0.24
$Ag_2Bi_2GePb_3$	1248	5.85	0.02	0.22
$Ag_2Bi_2GePb_3$	1664	5.93	0.02	0.25
$Ag_2Bi_2GePb_3$	390	6.04	0.02	0.22
$Ag_2Bi_2GePb_3$	532	6.12	0.02	0.25
$Ag_2Bi_2GePb_3$	1625	6.83	0.03	0.35

Continued on next page

Table S-10 – Continued from previous page

Metal composition	$N_{\mathrm{Str.}}$	ΔE^i_{tot}	Δa^i_0	ΔV^i_0
$Ag_2Bi_2GePb_3$	385	6.96	0.04	0.43
$Ag_2Bi_2GePb_3$	377	10.71	0.01	0.11
$Ag_2Bi_2GePb_3$	1586	10.99	0.03	0.30
$Ag_2Bi_2GePb_3$	1258	12.25	0.03	0.31
$Ag_2Bi_2GePb_3$	934	12.30	0.03	0.31
$Ag_2Bi_2GePb_3$	218	12.47	0.03	0.35
$Ag_2Bi_2GePb_3$	894	12.79	−0.01	−0.14
$Ag_2Bi_2GePb_3$	764	12.85	−0.02	−0.17
$Ag_2Bi_2GePb_3$	966	14.33	0.04	0.41
$Ag_2Bi_2GePb_3$	179	17.11	0.02	0.20
$Ag_2Bi_2GePb_3$	1217	17.23	0.03	0.29
$Ag_2Bi_2GePb_3$	664	18.15	0.05	0.49
$Ag_2Bi_2GePb_3$	1138	20.60	0.05	0.48
$Ag_2Bi_2GePb_3$	1647	22.66	0.02	0.21
$Ag_2Bi_2GePb_3$	693	22.67	0.02	0.18
$Ag_2Bi_2GePb_3$	901	24.22	0.02	0.21
$Ag_2Bi_2SnPb_3$	649	0.00	0.00	0.00
$Ag_2Bi_2SnPb_3$	532	5.37	0.00	0.00
$Ag_2Bi_2SnPb_3$	1664	5.58	0.00	−0.02
$Ag_2Bi_2SnPb_3$	84	8.79	0.00	−0.02
$Ag_2Bi_2SnPb_3$	1248	9.11	0.00	−0.02
$Ag_2Bi_2SnPb_3$	224	9.35	0.00	−0.02
$Ag_2Bi_2SnPb_3$	385	10.17	0.03	0.30
$Ag_2Bi_2SnPb_3$	1625	10.19	0.03	0.27
$Ag_2Bi_2SnPb_3$	934	14.91	0.00	−0.01
$Ag_2Bi_2SnPb_3$	377	14.92	0.00	0.02
$Ag_2Bi_2SnPb_3$	390	15.28	0.00	0.00
$Ag_2Bi_2SnPb_3$	1586	16.13	0.02	0.23
$Ag_2Bi_2SnPb_3$	218	17.24	0.02	0.19
$Ag_2Bi_2SnPb_3$	966	17.41	0.02	0.15
$Ag_2Bi_2SnPb_3$	1138	18.27	0.01	0.12
$Ag_2Bi_2SnPb_3$	664	18.42	0.01	0.13
$Ag_2Bi_2SnPb_3$	764	18.63	−0.01	−0.06
$Ag_2Bi_2SnPb_3$	894	18.75	−0.01	−0.09
$Ag_2Bi_2SnPb_3$	1647	18.91	−0.01	−0.06
$Ag_2Bi_2SnPb_3$	693	19.27	0.00	−0.05

Continued on next page

Table S-10 – Continued from previous page

Metal composition	$N_{\text{Str.}}$	ΔE^{i}_{tot}	Δa^{i}_{0}	ΔV^{i}_{0}
$Ag_2Bi_2SnPb_3$	1217	19.68	−0.01	−0.06
$Ag_2Bi_2SnPb_3$	179	19.69	0.00	−0.01
$Ag_2Bi_2SnPb_3$	901	23.33	0.00	0.05
$Ag_2Bi_2SnPb_3$	1258	23.53	0.02	0.21
$Ag_2Bi_2Ge_2Sn_2$	1185	0.00	0.00	0.00
$Ag_2Bi_2Ge_2Sn_2$	404	5.20	0.01	0.07
$Ag_2Bi_2Ge_2Sn_2$	1827	5.23	0.00	0.03
$Ag_2Bi_2Ge_2Sn_2$	378	6.96	−0.01	−0.07
$Ag_2Bi_2Ge_2Sn_2$	2425	11.17	0.00	0.02
$Ag_2Bi_2Ge_2Sn_2$	538	13.08	0.01	0.13
$Ag_2Bi_2Ge_2Sn_2$	291	13.16	0.00	−0.02
$Ag_2Bi_2Ge_2Sn_2$	1325	13.20	0.00	−0.01
$Ag_2Bi_2Ge_2Sn_2$	1479	13.25	0.00	0.00
$Ag_2Bi_2Ge_2Sn_2$	457	13.26	0.00	−0.01
$Ag_2Bi_2Ge_2Sn_2$	1194	13.26	0.00	−0.03
$Ag_2Bi_2Ge_2Sn_2$	992	13.27	0.00	−0.01
$Ag_2Bi_2Ge_2Sn_2$	880	14.75	0.01	0.07
$Ag_2Bi_2Ge_2Sn_2$	181	14.85	0.02	0.16
$Ag_2Bi_2Ge_2Sn_2$	516	14.95	−0.01	−0.05
$Ag_2Bi_2Ge_2Sn_2$	411	14.97	−0.01	−0.07
$Ag_2Bi_2Ge_2Sn_2$	136	15.66	−0.02	−0.17
$Ag_2Bi_2Ge_2Sn_2$	1694	15.73	−0.02	−0.20
$Ag_2Bi_2Ge_2Sn_2$	2253	16.42	0.00	−0.02
$Ag_2Bi_2Ge_2Sn_2$	473	16.54	0.00	−0.04
$Ag_2Bi_2Ge_2Sn_2$	1888	16.65	−0.02	−0.20
$Ag_2Bi_2Ge_2Sn_2$	2407	16.66	0.00	0.02
$Ag_2Bi_2Ge_2Sn_2$	1792	19.96	−0.01	−0.10
$Ag_2Bi_2Ge_2Sn_2$	531	20.41	−0.02	−0.17
$Ag_2Bi_2Ge_2Sn_2$	1914	21.57	−0.02	−0.16
$Ag_2Bi_2Ge_2Sn_2$	1950	21.70	−0.01	−0.13
$Ag_2Bi_2Ge_2Sn_2$	372	22.35	0.00	0.02
$Ag_2Bi_2Ge_2Sn_2$	1906	27.30	−0.01	−0.09
$Ag_2Bi_2Ge_2Sn_2$	2340	27.38	−0.01	−0.05
$Ag_2Bi_2Ge_2Sn_2$	675	27.95	−0.01	−0.10
$Ag_2Bi_2Ge_2Pb_2$	1185	0.00	0.00	0.00
$Ag_2Bi_2Ge_2Pb_2$	378	3.73	−0.01	−0.05

Continued on next page

Table S-10 – Continued from previous page

Metal composition	$N_{\text{Str.}}$	ΔE^i_{tot}	Δa^i_0	ΔV^i_0
$Ag_2Bi_2Ge_2Pb_2$	404	4.80	0.02	0.23
$Ag_2Bi_2Ge_2Pb_2$	1827	4.82	0.02	0.21
$Ag_2Bi_2Ge_2Pb_2$	538	9.50	0.02	0.15
$Ag_2Bi_2Ge_2Pb_2$	2425	10.35	0.00	−0.04
$Ag_2Bi_2Ge_2Pb_2$	181	10.86	0.02	0.23
$Ag_2Bi_2Ge_2Pb_2$	880	11.02	0.03	0.30
$Ag_2Bi_2Ge_2Pb_2$	992	11.27	0.00	0.02
$Ag_2Bi_2Ge_2Pb_2$	2407	11.38	0.02	0.22
$Ag_2Bi_2Ge_2Pb_2$	291	12.07	0.00	0.05
$Ag_2Bi_2Ge_2Pb_2$	1479	12.08	0.02	0.15
$Ag_2Bi_2Ge_2Pb_2$	1325	12.15	0.01	0.14
$Ag_2Bi_2Ge_2Pb_2$	457	12.19	0.01	0.06
$Ag_2Bi_2Ge_2Pb_2$	1194	12.21	0.01	0.06
$Ag_2Bi_2Ge_2Pb_2$	473	14.37	−0.01	−0.13
$Ag_2Bi_2Ge_2Pb_2$	1950	14.70	0.00	−0.02
$Ag_2Bi_2Ge_2Pb_2$	411	14.82	0.02	0.16
$Ag_2Bi_2Ge_2Pb_2$	516	14.92	0.02	0.21
$Ag_2Bi_2Ge_2Pb_2$	1694	16.03	−0.01	−0.06
$Ag_2Bi_2Ge_2Pb_2$	136	16.26	−0.01	−0.13
$Ag_2Bi_2Ge_2Pb_2$	2253	16.65	−0.01	−0.14
$Ag_2Bi_2Ge_2Pb_2$	1888	17.34	0.00	−0.04
$Ag_2Bi_2Ge_2Pb_2$	531	17.60	0.02	0.21
$Ag_2Bi_2Ge_2Pb_2$	1792	20.54	−0.01	−0.12
$Ag_2Bi_2Ge_2Pb_2$	372	22.15	0.00	0.02
$Ag_2Bi_2Ge_2Pb_2$	1914	23.35	0.00	−0.02
$Ag_2Bi_2Ge_2Pb_2$	2340	25.38	0.00	0.00
$Ag_2Bi_2Ge_2Pb_2$	1906	25.84	0.01	0.08
$Ag_2Bi_2Ge_2Pb_2$	675	26.71	0.00	−0.05
$Ag_2Bi_2Sn_2Pb_2$	1185	0.00	0.00	0.00
$Ag_2Bi_2Sn_2Pb_2$	378	1.11	0.00	0.03
$Ag_2Bi_2Sn_2Pb_2$	404	4.25	0.00	0.02
$Ag_2Bi_2Sn_2Pb_2$	1827	4.41	0.00	0.02
$Ag_2Bi_2Sn_2Pb_2$	2425	5.82	0.00	0.04
$Ag_2Bi_2Sn_2Pb_2$	1888	9.92	−0.01	−0.07
$Ag_2Bi_2Sn_2Pb_2$	538	10.36	0.02	0.23
$Ag_2Bi_2Sn_2Pb_2$	1479	11.93	−0.01	−0.14

Continued on next page

Table S-10 – Continued from previous page

Metal composition	$N_{\text{Str.}}$	ΔE^i_{tot}	Δa^i_0	ΔV^i_0
$Ag_2Bi_2Sn_2Pb_2$	880	12.72	0.01	0.15
$Ag_2Bi_2Sn_2Pb_2$	181	12.76	0.02	0.19
$Ag_2Bi_2Sn_2Pb_2$	457	12.82	−0.01	−0.13
$Ag_2Bi_2Sn_2Pb_2$	1950	12.88	−0.01	−0.14
$Ag_2Bi_2Sn_2Pb_2$	291	12.93	−0.01	−0.11
$Ag_2Bi_2Sn_2Pb_2$	992	12.97	−0.02	−0.15
$Ag_2Bi_2Sn_2Pb_2$	1194	12.98	−0.01	−0.11
$Ag_2Bi_2Sn_2Pb_2$	1325	13.03	−0.01	−0.14
$Ag_2Bi_2Sn_2Pb_2$	531	13.87	0.01	0.14
$Ag_2Bi_2Sn_2Pb_2$	136	13.92	0.00	−0.01
$Ag_2Bi_2Sn_2Pb_2$	1694	13.92	0.00	−0.04
$Ag_2Bi_2Sn_2Pb_2$	516	14.27	0.00	−0.04
$Ag_2Bi_2Sn_2Pb_2$	411	14.32	−0.01	−0.06
$Ag_2Bi_2Sn_2Pb_2$	2407	16.68	0.00	−0.02
$Ag_2Bi_2Sn_2Pb_2$	473	16.80	0.00	−0.03
$Ag_2Bi_2Sn_2Pb_2$	2253	16.82	0.00	0.04
$Ag_2Bi_2Sn_2Pb_2$	1792	17.93	−0.01	−0.12
$Ag_2Bi_2Sn_2Pb_2$	1914	18.33	−0.01	−0.09
$Ag_2Bi_2Sn_2Pb_2$	372	21.28	0.01	0.12
$Ag_2Bi_2Sn_2Pb_2$	675	24.07	−0.01	−0.12
$Ag_2Bi_2Sn_2Pb_2$	1906	27.17	0.00	−0.03
$Ag_2Bi_2Sn_2Pb_2$	2340	27.21	0.00	0.01
$Ag_2Bi_2Ge_3Sn$	1394	0.00	0.00	0.00
$Ag_2Bi_2Ge_3Sn$	684	3.18	0.00	0.00
$Ag_2Bi_2Ge_3Sn$	556	4.72	−0.01	−0.11
$Ag_2Bi_2Ge_3Sn$	1304	4.74	−0.01	−0.06
$Ag_2Bi_2Ge_3Sn$	473	4.86	−0.01	−0.08
$Ag_2Bi_2Ge_3Sn$	325	9.39	−0.03	−0.29
$Ag_2Bi_2Ge_3Sn$	690	9.44	−0.03	−0.30
$Ag_2Bi_2Ge_3Sn$	1676	10.14	−0.01	−0.07
$Ag_2Bi_2Ge_3Sn$	911	10.15	−0.01	−0.07
$Ag_2Bi_2Ge_3Sn$	233	11.49	0.01	0.10
$Ag_2Bi_2Ge_3Sn$	1647	12.04	0.00	−0.03
$Ag_2Bi_2Ge_3Sn$	34	12.30	0.00	0.04
$Ag_2Bi_2Ge_3Sn$	689	14.88	0.01	0.07
$Ag_2Bi_2Ge_3Sn$	186	15.39	0.01	0.06

Continued on next page

Table S-10 – Continued from previous page

Metal composition	$N_{\text{Str.}}$	ΔE^i_{tot}	Δa^i_0	ΔV^i_0
$Ag_2Bi_2Ge_3Sn$	243	18.30	−0.02	−0.15
$Ag_2Bi_2Ge_3Sn$	1584	18.31	−0.01	−0.14
$Ag_2Bi_2Ge_3Sn$	682	18.34	−0.01	−0.05
$Ag_2Bi_2Ge_3Sn$	265	18.42	0.03	0.26
$Ag_2Bi_2Ge_3Sn$	346	21.39	0.01	0.11
$Ag_2Bi_2Ge_3Sn$	1640	21.40	−0.02	−0.16
$Ag_2Bi_2Ge_3Sn$	328	21.46	0.01	0.11
$Ag_2Bi_2Ge_3Sn$	86	21.94	−0.02	−0.18
$Ag_2Bi_2Ge_3Sn$	977	22.07	0.02	0.19
$Ag_2Bi_2Ge_3Sn$	1380	22.69	0.00	0.00
$Ag_2Bi_2Ge_3Sn$	354	22.69	0.00	−0.02
$Ag_2Bi_2Ge_3Sn$	87	27.49	0.00	−0.02
$Ag_2Bi_2Ge_3Pb$	1394	0.00	0.00	0.00
$Ag_2Bi_2Ge_3Pb$	684	1.08	0.01	0.06
$Ag_2Bi_2Ge_3Pb$	473	3.30	−0.01	−0.14
$Ag_2Bi_2Ge_3Pb$	556	3.31	−0.02	−0.18
$Ag_2Bi_2Ge_3Pb$	1304	3.34	−0.01	−0.11
$Ag_2Bi_2Ge_3Pb$	325	7.76	−0.02	−0.24
$Ag_2Bi_2Ge_3Pb$	690	7.92	−0.03	−0.27
$Ag_2Bi_2Ge_3Pb$	1647	8.40	0.00	0.03
$Ag_2Bi_2Ge_3Pb$	911	9.65	0.00	−0.04
$Ag_2Bi_2Ge_3Pb$	1676	9.67	−0.01	−0.07
$Ag_2Bi_2Ge_3Pb$	233	11.22	0.00	−0.02
$Ag_2Bi_2Ge_3Pb$	34	11.27	−0.01	−0.12
$Ag_2Bi_2Ge_3Pb$	689	14.08	0.02	0.19
$Ag_2Bi_2Ge_3Pb$	265	14.55	0.04	0.39
$Ag_2Bi_2Ge_3Pb$	186	14.72	0.02	0.24
$Ag_2Bi_2Ge_3Pb$	243	17.69	−0.01	−0.11
$Ag_2Bi_2Ge_3Pb$	682	17.73	−0.01	−0.05
$Ag_2Bi_2Ge_3Pb$	1584	17.75	−0.01	−0.07
$Ag_2Bi_2Ge_3Pb$	1640	20.45	0.00	−0.04
$Ag_2Bi_2Ge_3Pb$	328	21.33	0.03	0.26
$Ag_2Bi_2Ge_3Pb$	346	21.36	0.03	0.29
$Ag_2Bi_2Ge_3Pb$	977	21.69	0.03	0.33
$Ag_2Bi_2Ge_3Pb$	86	22.02	−0.02	−0.20
$Ag_2Bi_2Ge_3Pb$	1380	22.23	0.00	0.03

Continued on next page

Table S-10 – Continued from previous page

Metal composition	$N_{\text{Str.}}$	ΔE^i_{tot}	Δa^i_0	ΔV^i_0
$Ag_2Bi_2Ge_3Pb$	354	22.73	−0.01	−0.09
$Ag_2Bi_2Ge_3Pb$	87	26.41	0.02	0.17
$Ag_2Bi_2Sn_3Pb$	1394	0.00	0.00	0.00
$Ag_2Bi_2Sn_3Pb$	473	0.77	0.00	−0.02
$Ag_2Bi_2Sn_3Pb$	684	2.52	0.00	−0.03
$Ag_2Bi_2Sn_3Pb$	1304	3.41	0.00	0.00
$Ag_2Bi_2Sn_3Pb$	556	3.43	0.00	−0.04
$Ag_2Bi_2Sn_3Pb$	690	8.20	0.01	0.06
$Ag_2Bi_2Sn_3Pb$	325	8.20	0.00	0.03
$Ag_2Bi_2Sn_3Pb$	911	9.06	0.01	0.08
$Ag_2Bi_2Sn_3Pb$	1676	9.21	0.00	0.01
$Ag_2Bi_2Sn_3Pb$	1647	9.60	0.01	0.06
$Ag_2Bi_2Sn_3Pb$	689	9.79	−0.01	−0.13
$Ag_2Bi_2Sn_3Pb$	34	10.54	0.03	0.32
$Ag_2Bi_2Sn_3Pb$	233	10.96	0.01	0.12
$Ag_2Bi_2Sn_3Pb$	86	13.93	−0.01	−0.11
$Ag_2Bi_2Sn_3Pb$	186	15.19	0.00	−0.04
$Ag_2Bi_2Sn_3Pb$	682	16.49	−0.02	−0.17
$Ag_2Bi_2Sn_3Pb$	1584	16.63	−0.01	−0.13
$Ag_2Bi_2Sn_3Pb$	243	16.69	−0.01	−0.09
$Ag_2Bi_2Sn_3Pb$	354	16.96	0.00	0.04
$Ag_2Bi_2Sn_3Pb$	1380	17.08	0.00	0.03
$Ag_2Bi_2Sn_3Pb$	1640	17.59	0.01	0.07
$Ag_2Bi_2Sn_3Pb$	265	17.64	0.02	0.15
$Ag_2Bi_2Sn_3Pb$	346	18.17	0.00	0.04
$Ag_2Bi_2Sn_3Pb$	977	19.82	0.00	0.04
$Ag_2Bi_2Sn_3Pb$	328	20.91	0.00	0.00
$Ag_2Bi_2Sn_3Pb$	87	25.48	−0.01	−0.12
$AgBiGeSn_5$	8	0.00	0.00	0.00
$AgBiGeSn_5$	53	0.46	0.00	−0.05
$AgBiGeSn_5$	285	4.31	−0.01	−0.07
$AgBiGeSn_5$	186	4.42	0.00	−0.02
$AgBiGeSn_5$	140	4.54	−0.01	−0.08
$AgBiGeSn_5$	196	5.88	−0.01	−0.15
$AgBiGeSn_5$	191	5.92	0.00	0.04
$AgBiGeSn_5$	146	5.96	0.00	−0.02

Continued on next page

Table S-10 – Continued from previous page

Metal composition	$N_{\mathrm{Str.}}$	ΔE^i_{tot}	Δa^i_0	ΔV^i_0
$AgBiGeSn_5$	327	6.10	−0.01	−0.14
$AgBiGePb_5$	285	0.00	0.00	0.00
$AgBiGePb_5$	186	0.06	0.01	0.13
$AgBiGePb_5$	53	0.86	0.00	0.05
$AgBiGePb_5$	8	0.92	0.01	0.11
$AgBiGePb_5$	196	1.24	0.02	0.20
$AgBiGePb_5$	140	4.87	0.00	0.04
$AgBiGePb_5$	327	6.58	0.02	0.18
$AgBiGePb_5$	146	6.75	0.02	0.21
$AgBiGePb_5$	191	6.79	0.02	0.16
$AgBiSnPb_5$	53	0.00	0.00	0.00
$AgBiSnPb_5$	8	2.50	0.00	0.02
$AgBiSnPb_5$	327	4.42	0.01	0.07
$AgBiSnPb_5$	186	5.03	0.00	0.03
$AgBiSnPb_5$	196	5.20	0.01	0.10
$AgBiSnPb_5$	191	9.11	0.01	0.06
$AgBiSnPb_5$	140	9.72	0.01	0.08
$AgBiSnPb_5$	285	9.74	0.00	0.02
$AgBiSnPb_5$	146	9.96	0.01	0.15
$AgBiGe_2Sn_4$	411	0.00	0.00	0.00
$AgBiGe_2Sn_4$	57	1.25	0.00	0.04
$AgBiGe_2Sn_4$	559	1.80	−0.01	−0.10
$AgBiGe_2Sn_4$	747	5.49	0.00	−0.02
$AgBiGe_2Sn_4$	788	5.91	−0.01	−0.07
$AgBiGe_2Sn_4$	112	6.32	0.00	−0.03
$AgBiGe_2Sn_4$	706	6.73	−0.01	−0.08
$AgBiGe_2Sn_4$	171	6.84	−0.01	−0.12
$AgBiGe_2Sn_4$	335	7.48	−0.02	−0.17
$AgBiGe_2Sn_4$	86	7.60	−0.01	−0.14
$AgBiGe_2Sn_4$	398	8.55	−0.01	−0.14
$AgBiGe_2Pb_4$	747	0.00	0.00	0.00
$AgBiGe_2Pb_4$	411	0.06	0.00	−0.02
$AgBiGe_2Pb_4$	788	0.31	0.00	0.03
$AgBiGe_2Pb_4$	335	1.61	−0.01	−0.11
$AgBiGe_2Pb_4$	171	1.72	−0.02	−0.19
$AgBiGe_2Pb_4$	57	1.84	0.01	0.06

Continued on next page

Table S-10 – Continued from previous page

Metal composition	$N_{\mathrm{Str.}}$	ΔE_{tot}^{i}	Δa_0^{i}	ΔV_0^{i}
$AgBiGe_2Pb_4$	559	2.52	−0.02	−0.22
$AgBiGe_2Pb_4$	398	2.82	0.00	0.00
$AgBiGe_2Pb_4$	112	6.37	0.01	0.11
$AgBiGe_2Pb_4$	706	7.00	−0.01	−0.14
$AgBiGe_2Pb_4$	86	7.63	−0.01	−0.11
$AgBiSn_2Pb_4$	411	0.00	0.00	0.00
$AgBiSn_2Pb_4$	559	0.02	−0.01	−0.06
$AgBiSn_2Pb_4$	57	0.48	0.00	−0.05
$AgBiSn_2Pb_4$	171	1.48	0.00	0.02
$AgBiSn_2Pb_4$	86	2.31	0.00	−0.04
$AgBiSn_2Pb_4$	335	2.87	0.00	0.02
$AgBiSn_2Pb_4$	706	4.30	0.00	0.01
$AgBiSn_2Pb_4$	788	5.11	0.00	−0.04
$AgBiSn_2Pb_4$	398	5.28	0.00	0.00
$AgBiSn_2Pb_4$	112	5.32	0.00	−0.03
$AgBiSn_2Pb_4$	747	5.42	0.00	0.02
$AgBiGe_3Sn_3$	114	0.00	0.00	0.00
$AgBiGe_3Sn_3$	279	0.08	−0.01	−0.07
$AgBiGe_3Sn_3$	138	0.66	0.00	−0.02
$AgBiGe_3Sn_3$	821	1.84	−0.01	−0.06
$AgBiGe_3Sn_3$	540	2.16	−0.02	−0.18
$AgBiGe_3Sn_3$	20	2.38	−0.02	−0.19
$AgBiGe_3Sn_3$	1083	5.41	−0.02	−0.18
$AgBiGe_3Sn_3$	473	6.64	−0.02	−0.15
$AgBiGe_3Sn_3$	383	7.16	−0.02	−0.18
$AgBiGe_3Sn_3$	14	7.47	−0.02	−0.21
$AgBiGe_3Sn_3$	956	7.60	−0.02	−0.18
$AgBiGe_3Sn_3$	88	7.88	−0.03	−0.25
$AgBiGe_3Sn_3$	754	8.67	−0.02	−0.23
$AgBiGe_3Pb_3$	279	0.00	0.00	0.00
$AgBiGe_3Pb_3$	114	0.15	0.00	−0.03
$AgBiGe_3Pb_3$	138	0.45	−0.01	−0.10
$AgBiGe_3Pb_3$	540	1.23	−0.02	−0.17
$AgBiGe_3Pb_3$	821	2.89	0.02	0.22
$AgBiGe_3Pb_3$	20	2.96	−0.01	−0.10
$AgBiGe_3Pb_3$	1083	5.75	−0.02	−0.22

Continued on next page

Table S-10 – Continued from previous page

Metal composition	$N_{\text{Str.}}$	ΔE^i_{tot}	Δa^i_0	ΔV^i_0
$AgBiGe_3Pb_3$	14	6.67	−0.01	−0.06
$AgBiGe_3Pb_3$	473	7.58	−0.02	−0.17
$AgBiGe_3Pb_3$	754	7.76	−0.02	−0.23
$AgBiGe_3Pb_3$	383	7.77	−0.02	−0.16
$AgBiGe_3Pb_3$	956	8.76	−0.01	−0.12
$AgBiGe_3Pb_3$	88	9.16	−0.03	−0.29
$AgBiSn_3Pb_3$	279	0.00	0.00	0.00
$AgBiSn_3Pb_3$	114	0.19	0.01	0.08
$AgBiSn_3Pb_3$	540	0.29	0.01	0.06
$AgBiSn_3Pb_3$	20	0.37	0.01	0.10
$AgBiSn_3Pb_3$	821	0.76	0.00	0.00
$AgBiSn_3Pb_3$	138	0.83	0.00	0.00
$AgBiSn_3Pb_3$	14	2.23	0.01	0.12
$AgBiSn_3Pb_3$	754	4.61	0.00	0.03
$AgBiSn_3Pb_3$	1083	4.90	0.01	0.10
$AgBiSn_3Pb_3$	956	5.29	0.01	0.08
$AgBiSn_3Pb_3$	383	5.38	0.01	0.10
$AgBiSn_3Pb_3$	473	5.57	0.01	0.11
$AgBiSn_3Pb_3$	88	6.37	0.01	0.13
$AgBiGe_4Sn_2$	19	0.00	0.00	0.00
$AgBiGe_4Sn_2$	701	0.85	−0.02	−0.23
$AgBiGe_4Sn_2$	272	1.00	0.02	0.18
$AgBiGe_4Sn_2$	515	1.06	0.02	0.16
$AgBiGe_4Sn_2$	171	5.80	0.02	0.21
$AgBiGe_4Sn_2$	304	6.02	0.02	0.16
$AgBiGe_4Sn_2$	75	6.03	0.01	0.07
$AgBiGe_4Sn_2$	582	6.19	0.01	0.07
$AgBiGe_4Sn_2$	33	6.35	0.01	0.06
$AgBiGe_4Sn_2$	202	6.39	−0.01	−0.07
$AgBiGe_4Sn_2$	236	7.07	−0.02	−0.16
$AgBiGe_4Sn_2$	668	7.72	−0.01	−0.07
$AgBiGe_4Sn_2$	361	7.73	0.00	−0.03
$AgBiGe_4Sn_2$	199	7.78	−0.01	−0.08
$AgBiGe_4Sn_2$	103	7.91	−0.01	−0.10
$AgBiGe_4Sn_2$	623	8.21	−0.01	−0.08
$AgBiGe_4Pb_2$	19	0.00	0.00	0.00

Continued on next page

Table S-10 – Continued from previous page

Metal composition	$N_{\text{Str.}}$	ΔE^i_{tot}	Δa^i_0	ΔV^i_0
$AgBiGe_4Pb_2$	701	1.93	0.00	0.04
$AgBiGe_4Pb_2$	515	2.90	0.03	0.32
$AgBiGe_4Pb_2$	272	2.91	0.04	0.39
$AgBiGe_4Pb_2$	75	7.10	0.01	0.10
$AgBiGe_4Pb_2$	304	7.21	0.04	0.38
$AgBiGe_4Pb_2$	171	7.61	0.04	0.39
$AgBiGe_4Pb_2$	202	7.69	−0.01	−0.06
$AgBiGe_4Pb_2$	582	8.06	0.01	0.06
$AgBiGe_4Pb_2$	33	8.10	0.01	0.07
$AgBiGe_4Pb_2$	236	8.31	0.00	−0.04
$AgBiGe_4Pb_2$	668	9.58	0.00	0.02
$AgBiGe_4Pb_2$	361	9.58	0.00	0.02
$AgBiGe_4Pb_2$	199	9.63	0.00	0.00
$AgBiGe_4Pb_2$	103	9.66	0.00	−0.03
$AgBiGe_4Pb_2$	623	10.27	0.00	0.00
$AgBiSn_4Pb_2$	19	0.00	0.00	0.00
$AgBiSn_4Pb_2$	701	0.82	0.01	0.07
$AgBiSn_4Pb_2$	515	0.97	0.00	0.05
$AgBiSn_4Pb_2$	272	1.06	0.00	−0.01
$AgBiSn_4Pb_2$	33	2.97	0.01	0.05
$AgBiSn_4Pb_2$	75	4.99	0.01	0.08
$AgBiSn_4Pb_2$	582	5.18	0.00	0.01
$AgBiSn_4Pb_2$	361	5.44	0.00	0.04
$AgBiSn_4Pb_2$	171	5.50	0.00	0.04
$AgBiSn_4Pb_2$	236	5.55	0.01	0.07
$AgBiSn_4Pb_2$	202	5.58	0.02	0.16
$AgBiSn_4Pb_2$	304	5.64	0.01	0.14
$AgBiSn_4Pb_2$	623	5.87	0.01	0.12
$AgBiSn_4Pb_2$	668	5.91	0.00	0.00
$AgBiSn_4Pb_2$	199	5.94	0.00	0.03
$AgBiSn_4Pb_2$	103	6.11	0.00	0.01
$AgBiGe_5Sn$	45	0.00	0.00	0.00
$AgBiGe_5Sn$	2	0.25	−0.03	−0.32
$AgBiGe_5Sn$	173	0.30	−0.02	−0.15
$AgBiGe_5Sn$	247	4.72	0.01	0.08
$AgBiGe_5Sn$	329	4.78	0.01	0.09

Continued on next page

Table S-10 – Continued from previous page

Metal composition	$N_{\mathrm{Str.}}$	ΔE^{i}_{tot}	Δa^{i}_{0}	ΔV^{i}_{0}
$AgBiGe_5Sn$	5	4.78	0.01	0.10
$AgBiGe_5Sn$	294	4.87	0.01	0.09
$AgBiGe_5Sn$	60	5.10	0.01	0.08
$AgBiGe_5Sn$	306	5.83	−0.02	−0.21
$AgBiGe_5Sn$	312	5.89	−0.01	−0.08
$AgBiGe_5Sn$	61	6.08	−0.01	−0.10
$AgBiGe_5Sn$	13	6.36	0.00	−0.03
$AgBiGe_5Pb$	2	0.00	0.00	0.00
$AgBiGe_5Pb$	173	0.28	0.01	0.06
$AgBiGe_5Pb$	45	0.35	0.00	0.05
$AgBiGe_5Pb$	247	5.23	0.03	0.28
$AgBiGe_5Pb$	60	5.25	0.03	0.30
$AgBiGe_5Pb$	5	5.36	0.03	0.32
$AgBiGe_5Pb$	294	5.43	0.02	0.24
$AgBiGe_5Pb$	329	5.54	0.03	0.27
$AgBiGe_5Pb$	61	5.57	0.00	0.03
$AgBiGe_5Pb$	312	5.66	0.00	0.03
$AgBiGe_5Pb$	306	5.96	−0.01	−0.11
$AgBiGe_5Pb$	13	7.07	0.01	0.09
$AgBiSn_5Pb$	2	0.00	0.00	0.00
$AgBiSn_5Pb$	173	0.93	0.01	0.05
$AgBiSn_5Pb$	45	1.09	0.00	0.03
$AgBiSn_5Pb$	247	5.01	0.00	0.03
$AgBiSn_5Pb$	312	5.31	0.01	0.15
$AgBiSn_5Pb$	294	5.35	0.01	0.05
$AgBiSn_5Pb$	60	5.39	0.01	0.06
$AgBiSn_5Pb$	329	5.48	0.01	0.06
$AgBiSn_5Pb$	5	5.50	0.01	0.10
$AgBiSn_5Pb$	306	5.63	0.00	0.05
$AgBiSn_5Pb$	61	5.73	0.01	0.15
$AgBiSn_5Pb$	13	6.26	0.02	0.20

S-6.5 Pristine Double-Perovskite

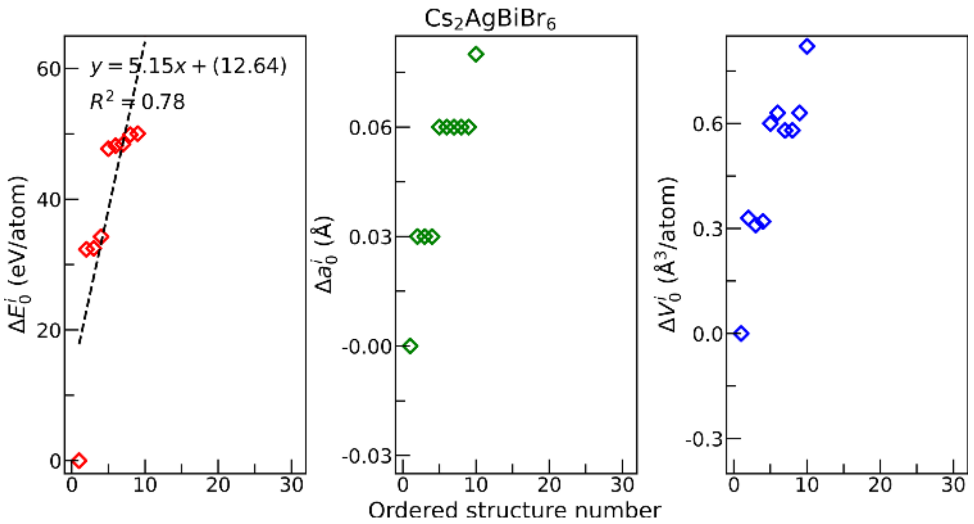

Figure S-21. Relative total energy (ΔE^i_{tot}), relative lattice constant at equilibrium (Δa^i_0), and relative equilibrium volume (ΔV^i_0), of the optimized structures of $Cs_2AgBiBr_6$ (or $Cs_8Ag_4Bi_4Br_{24}$) with respect to the structure with the lowest energy; $N_G = 10$.

S-6.6 Mixtures with One Divalent Metal

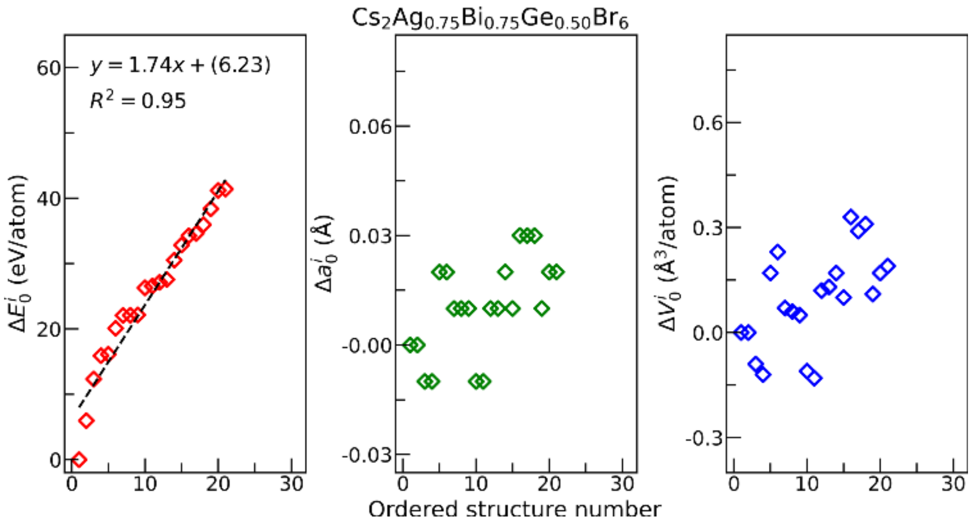

Figure S-22. Relative total energy (ΔE^i_{tot}), relative lattice constant at equilibrium (Δa^i_0), and relative equilibrium volume (ΔV^i_0), of the optimized structures of $Cs_2Ag_{0.75}Bi_{0.75}Ge_{0.50}Br_6$ (or $Cs_8Ag_3Bi_3Ge_2Br_{24}$) with respect to the structure with the lowest energy; $N_G = 21$.

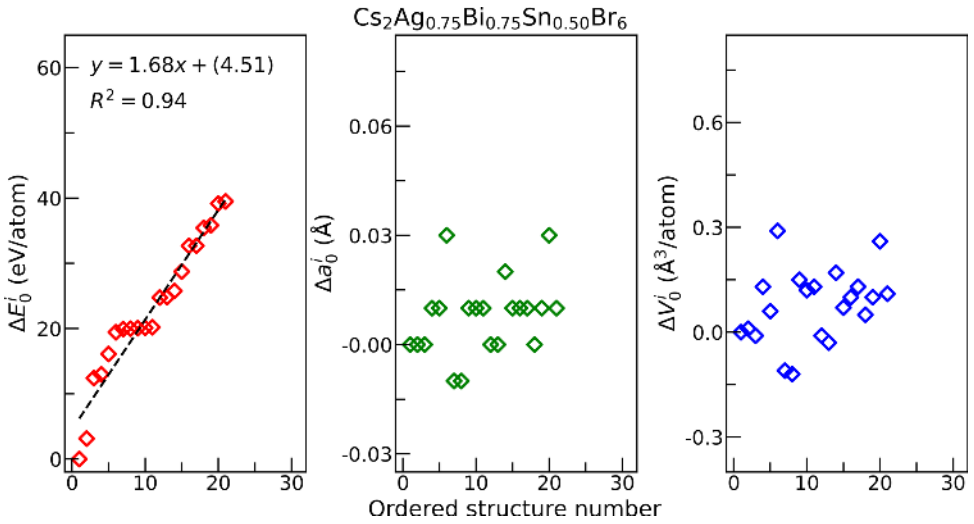

Figure S-23. Relative total energy (ΔE^i_{tot}), relative lattice constant at equilibrium (Δa^i_0), and relative equilibrium volume (ΔV^i_0), of the optimized structures of $Cs_2Ag_{0.75}Bi_{0.75}Sn_{0.50}Br_6$ (or $Cs_8Ag_3Bi_3Sn_2Br_{24}$) with respect to the structure with the lowest energy; N_G = 21.

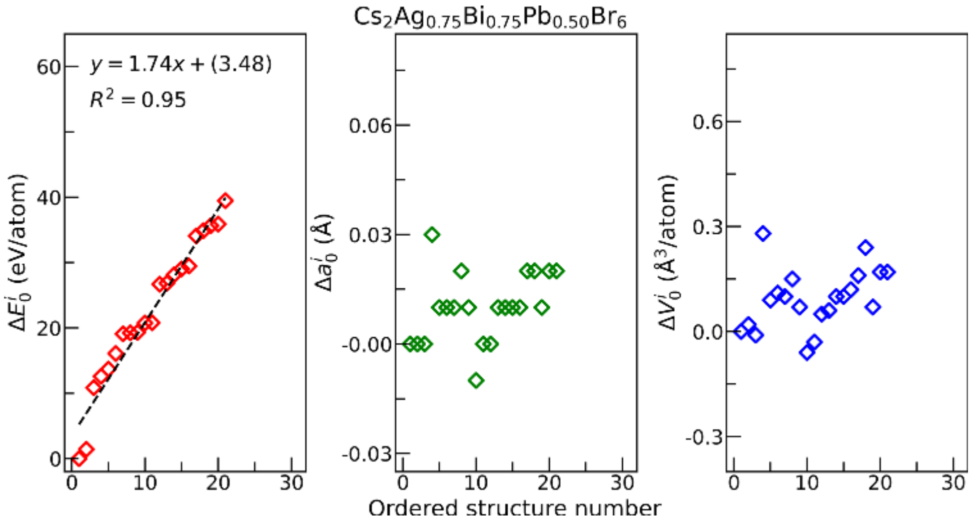

Figure S-24. Relative total energy (ΔE^i_{tot}), relative lattice constant at equilibrium (Δa^i_0), and relative equilibrium volume (ΔV^i_0), of the optimized structures of $Cs_2Ag_{0.75}Bi_{0.75}Pb_{0.50}Br_6$ (or $Cs_8Ag_3Bi_3Pb_2Br_{24}$) with respect to the structure with the lowest energy; N_G = 21.

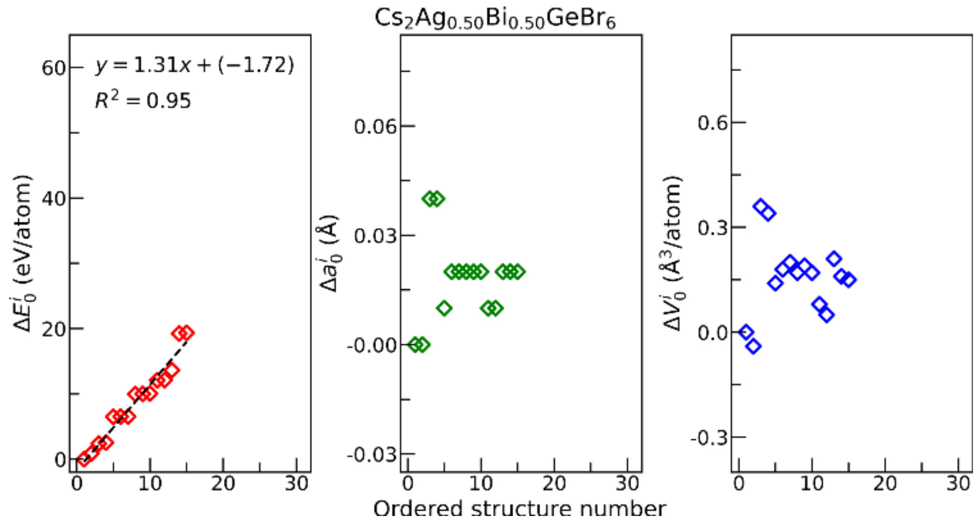

Figure S-25. Relative total energy (ΔE_{tot}^i), relative lattice constant at equilibrium (Δa_0^i), and relative equilibrium volume (ΔV_0^i), of the optimized structures of $Cs_2Ag_{0.50}Bi_{0.50}GeBr_6$ (or $Cs_8Ag_2Bi_2Ge_4Br_{24}$) with respect to the structure with the lowest energy; N_G = 15.

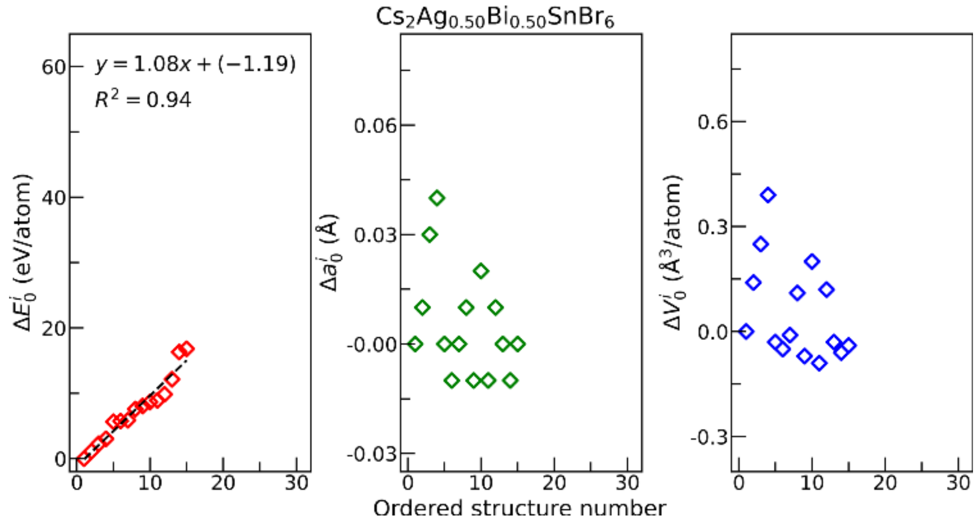

Figure S-26. Relative total energy (ΔE_{tot}^i), relative lattice constant at equilibrium (Δa_0^i), and relative equilibrium volume (ΔV_0^i), of the optimized structures of $Cs_2Ag_{0.50}Bi_{0.50}SnBr_6$ (or $Cs_8Ag_2Bi_2Sn_4Br_{24}$) with respect to the structure with the lowest energy; N_G = 15.

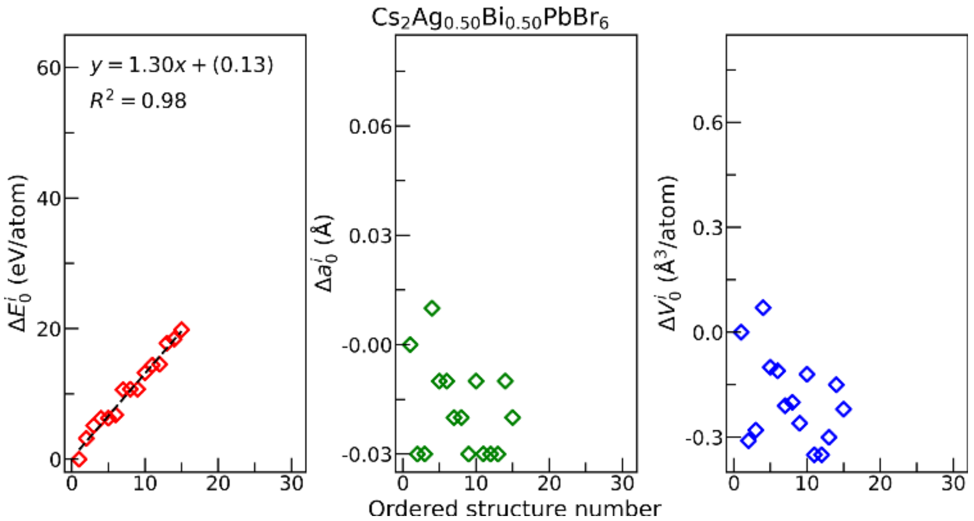

Figure S-27. Relative total energy (ΔE^i_{tot}), relative lattice constant at equilibrium (Δa^i_0), and relative equilibrium volume (ΔV^i_0), of the optimized structures of $Cs_2Ag_{0.50}Bi_{0.50}PbBr_6$ (or $Cs_8Ag_2Bi_2Pb_4Br_{24}$) with respect to the structure with the lowest energy; N_G = 15.

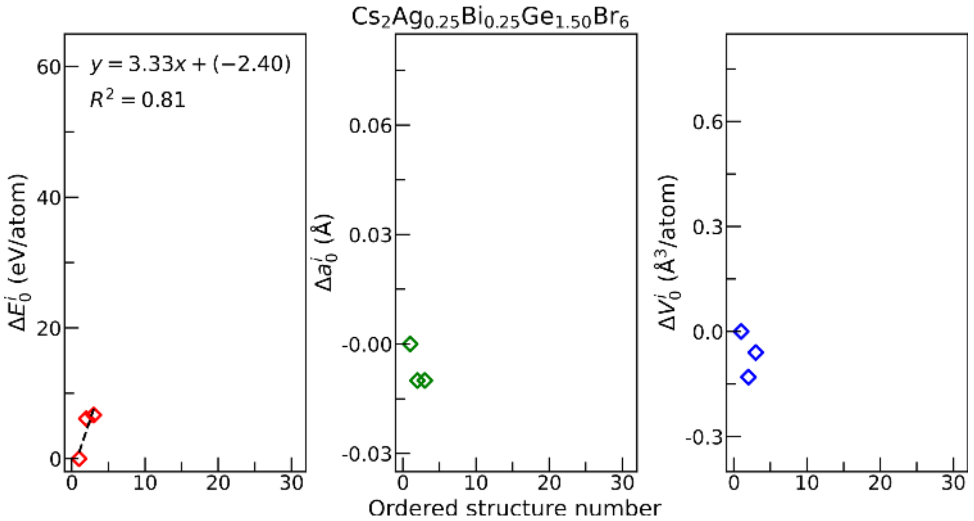

Figure S-28. Relative total energy (ΔE^i_{tot}), relative lattice constant at equilibrium (Δa^i_0), and relative equilibrium volume (ΔV^i_0), of the optimized structures of $Cs_2Ag_{0.25}Bi_{0.25}Ge_{1.50}Br_6$ (or $Cs_8AgBiGe_6Br_{24}$) with respect to the structure with the lowest energy; N_G = 3.

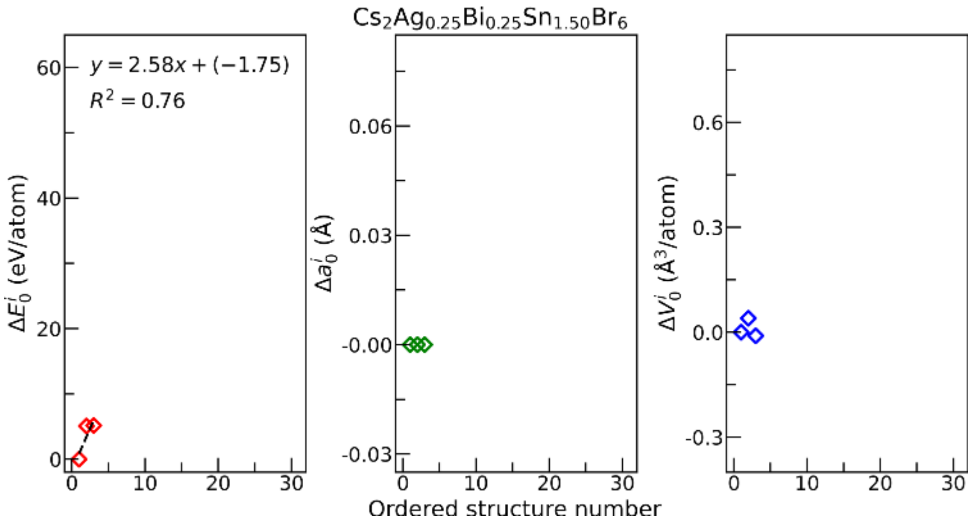

Figure S-29. Relative total energy (ΔE^i_{tot}), relative lattice constant at equilibrium (Δa^i_0), and relative equilibrium volume (ΔV^i_0), of the optimized structures of $Cs_2Ag_{0.25}Bi_{0.25}Sn_{1.5}Br_6$ (or $Cs_8AgBiSn_6Br_{24}$) with respect to the structure with the lowest energy; $N_G = 3$.

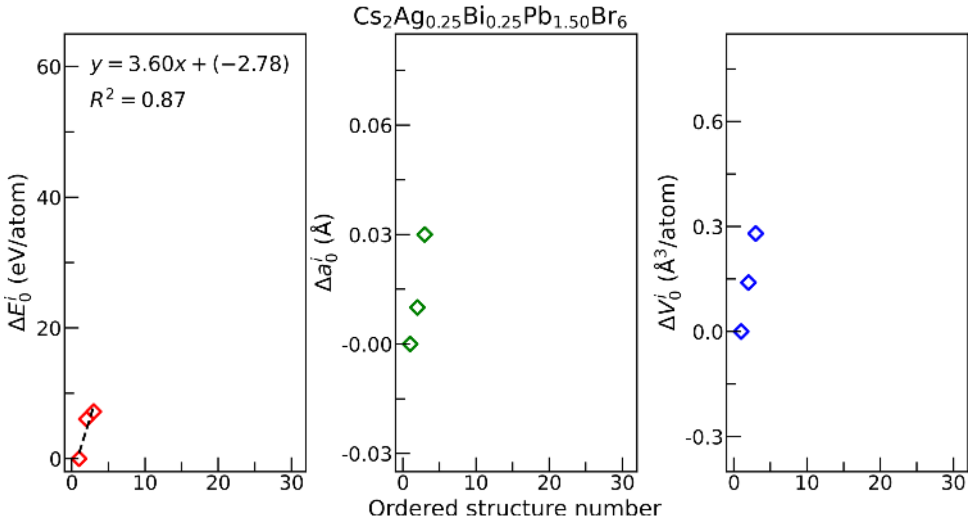

Figure S-30. Relative total energy (ΔE^i_{tot}), relative lattice constant at equilibrium (Δa^i_0), and relative equilibrium volume (ΔV^i_0), of the optimized structures of $Cs_2Ag_{0.25}Bi_{0.25}Pb_{1.5}Br_6$ (or $Cs_8AgBiPb_6Br_{24}$) with respect to the structure with the lowest energy; $N_G = 3$.

S-6.7 Mixtures with Two Divalent Metals

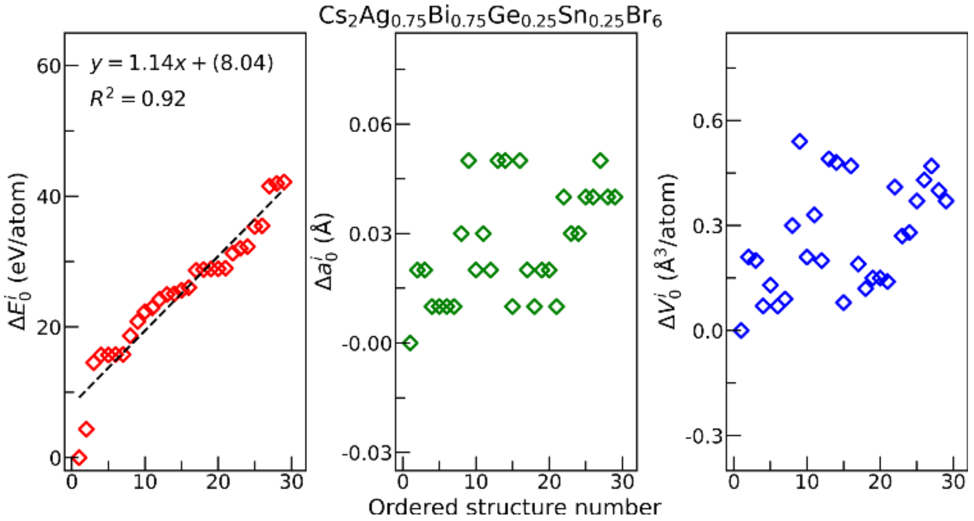

Figure S-31. Relative total energy (ΔE^i_{tot}), relative lattice constant at equilibrium (Δa^i_0), and relative equilibrium volume (ΔV^i_0), of the optimized structures of $Cs_2Ag_{0.75}Bi_{0.75}Ge_{0.25}Sn_{0.25}Br_6$ (or $Cs_8Ag_3Bi_3GeSnBr_{24}$) with respect to the structure with the lowest energy; $N_G = 29$.

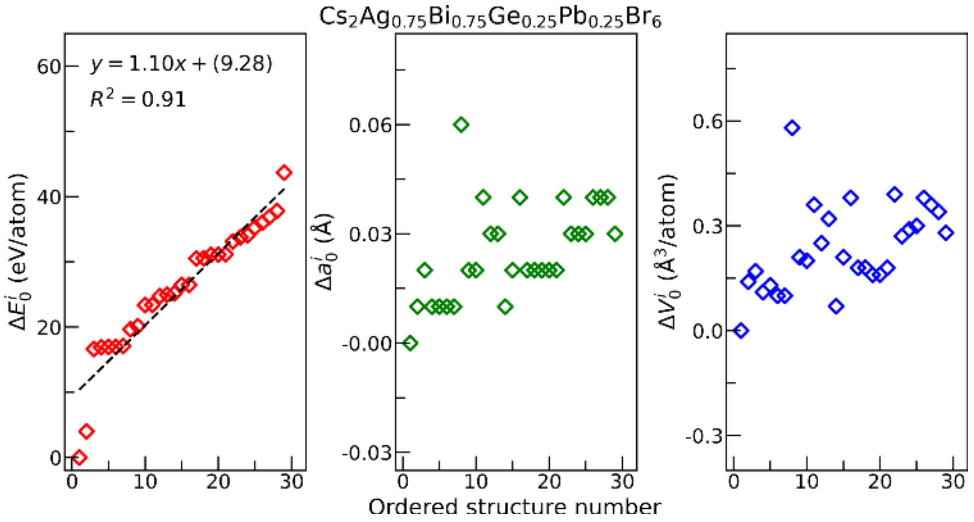

Figure S-32. Relative total energy (ΔE^i_{tot}), relative lattice constant at equilibrium (Δa^i_0), and relative equilibrium volume (ΔV^i_0), of the optimized structures of $Cs_2Ag_{0.75}Bi_{0.75}Ge_{0.25}Pb_{0.25}Br_6$ (or $Cs_8Ag_3Bi_3GePbBr_{24}$) with respect to the structure with the lowest energy; $N_G = 29$.

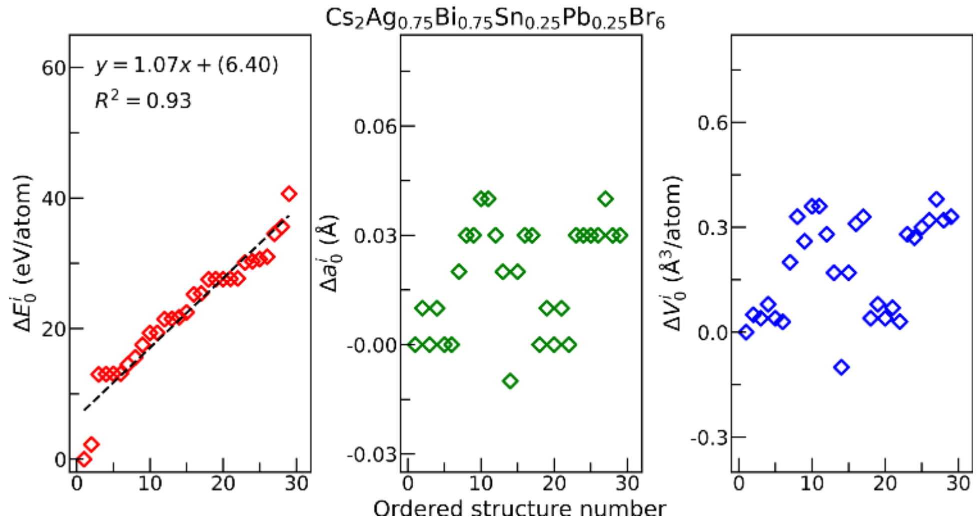

Figure S-33. Relative total energy (ΔE_{tot}^i), relative lattice constant at equilibrium (Δa_0^i), and relative equilibrium volume (ΔV_0^i), of the optimized structures of $Cs_2Ag_{0.75}Bi_{0.75}Sn_{0.25}Pb_{0.25}Br_6$ (or $Cs_8Ag_3Bi_3SnPbBr_{24}$) with respect to the structure with the lowest energy; N_G = 29.

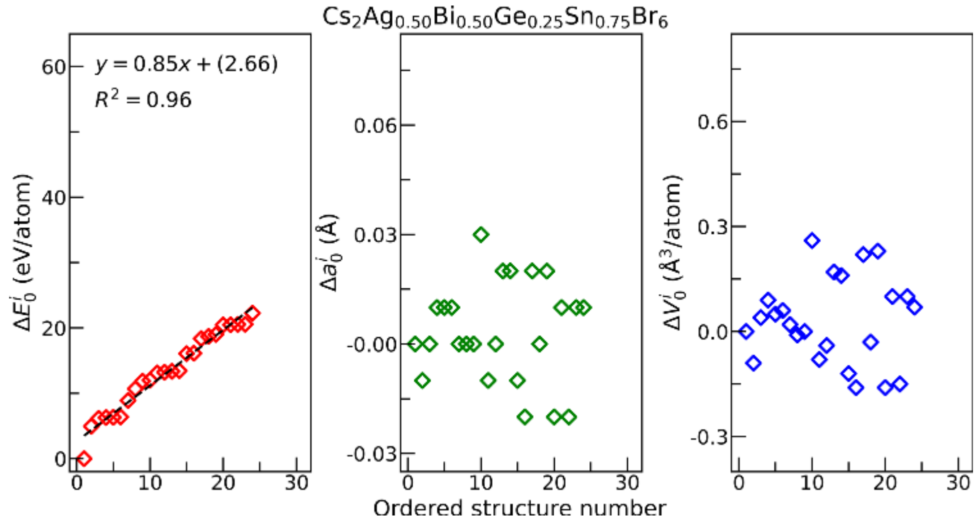

Figure S-34. Relative total energy (ΔE_{tot}^i), relative lattice constant at equilibrium (Δa_0^i), and relative equilibrium volume (ΔV_0^i), of the optimized structures of $Cs_2Ag_{0.50}Bi_{0.50}Ge_{0.25}Sn_{0.75}Br_6$ (or $Cs_8Ag_2Bi_2GeSn_3Br_{24}$) with respect to the structure with the lowest energy; N_G = 24.

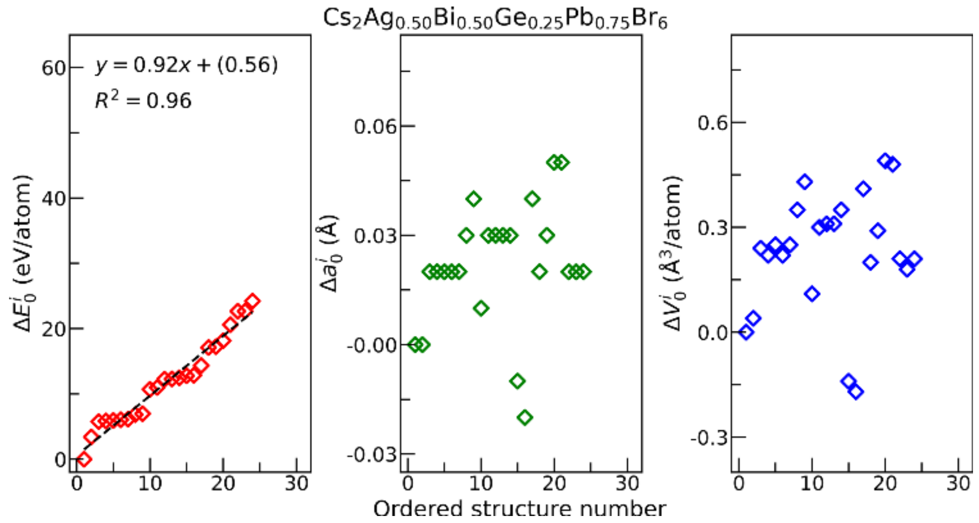

Figure S-35. Relative total energy (ΔE^i_{tot}), relative lattice constant at equilibrium (Δa^i_0), and relative equilibrium volume (ΔV^i_0), of the optimized structures of $Cs_2Ag_{0.50}Bi_{0.50}Ge_{0.25}Pb_{0.75}Br_6$ (or $Cs_8Ag_2Bi_2GePb_3Br_{24}$) with respect to the structure with the lowest energy; N_G = 24.

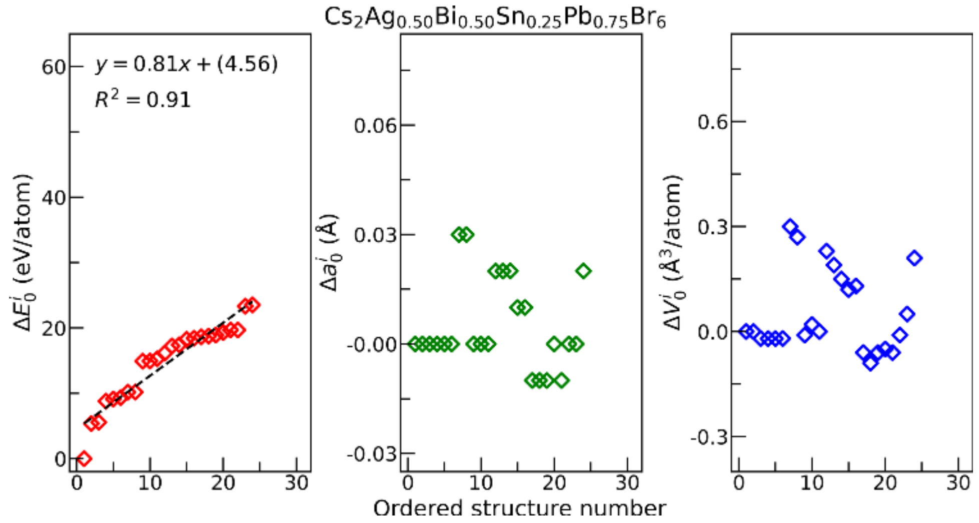

Figure S-36. Relative total energy (ΔE^i_{tot}), relative lattice constant at equilibrium (Δa^i_0), and relative equilibrium volume (ΔV^i_0), of the optimized structures of $Cs_2Ag_{0.50}Bi_{0.50}Sn_{0.25}Pb_{0.75}Br_6$ (or $Cs_8Ag_2Bi_2SnPb_3Br_{24}$) with respect to the structure with the lowest energy; N_G = 24.

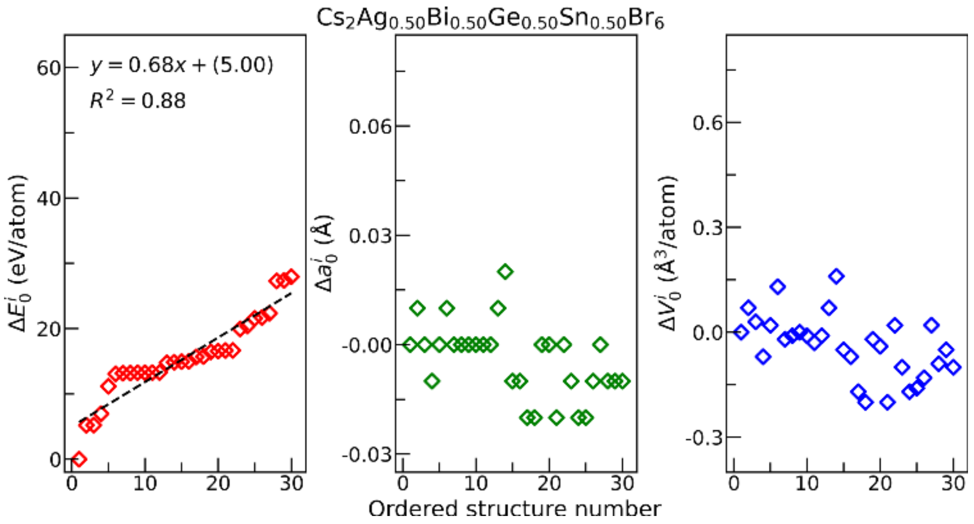

Figure S-37. Relative total energy (ΔE_{tot}^i), relative lattice constant at equilibrium (Δa_0^i), and relative equilibrium volume (ΔV_0^i), of the optimized structures of $Cs_2Ag_{0.50}Bi_{0.50}Ge_{0.50}Sn_{0.50}Br_6$ (or $Cs_8Ag_2Bi_2Ge_2Sn_2Br_{24}$) with respect to the structure with the lowest energy; N_G = 30.

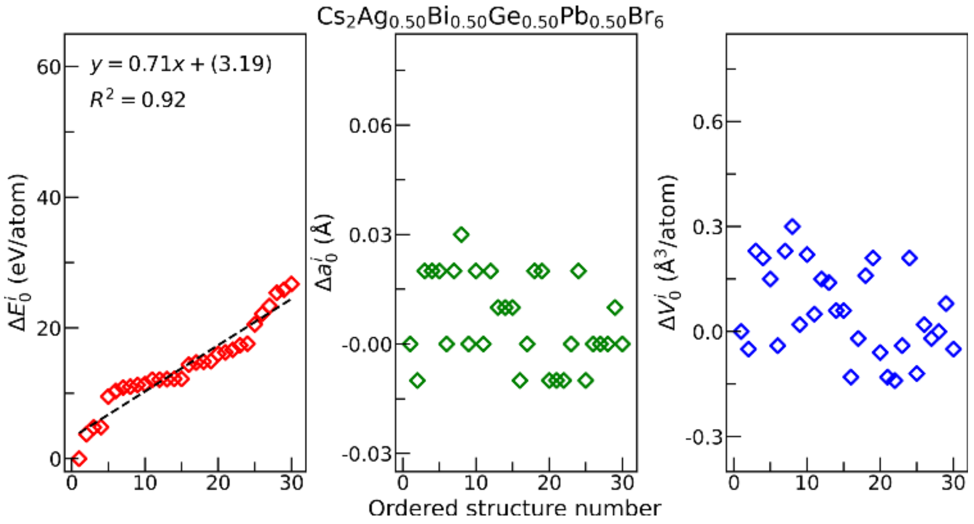

Figure S-38. Relative total energy (ΔE_{tot}^i), relative lattice constant at equilibrium (Δa_0^i), and relative equilibrium volume (ΔV_0^i), of the optimized structures of $Cs_2Ag_{0.50}Bi_{0.50}Ge_{0.50}Pb_{0.50}Br_6$ (or $Cs_8Ag_2Bi_2Ge_2Pb_2Br_{24}$) with respect to the structure with the lowest energy; N_G = 30.

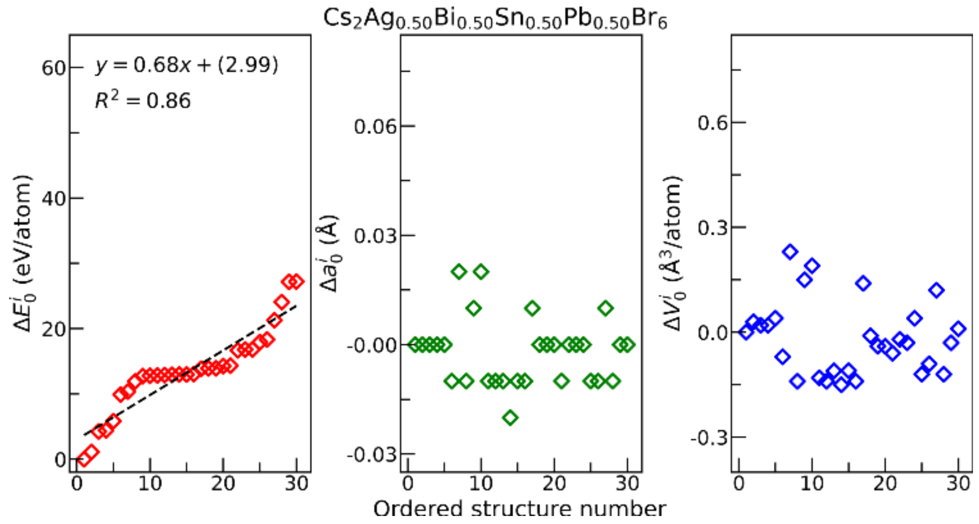

Figure S-39. Relative total energy (ΔE^i_{tot}), relative lattice constant at equilibrium (Δa^i_0), and relative equilibrium volume (ΔV^i_0), of the optimized structures of $Cs_2Ag_{0.50}Bi_{0.50}Sn_{0.50}Pb_{0.50}Br_6$ (or $Cs_8Ag_2Bi_2Sn_2Pb_2Br_{24}$) with respect to the structure with the lowest energy; N_G = 30.

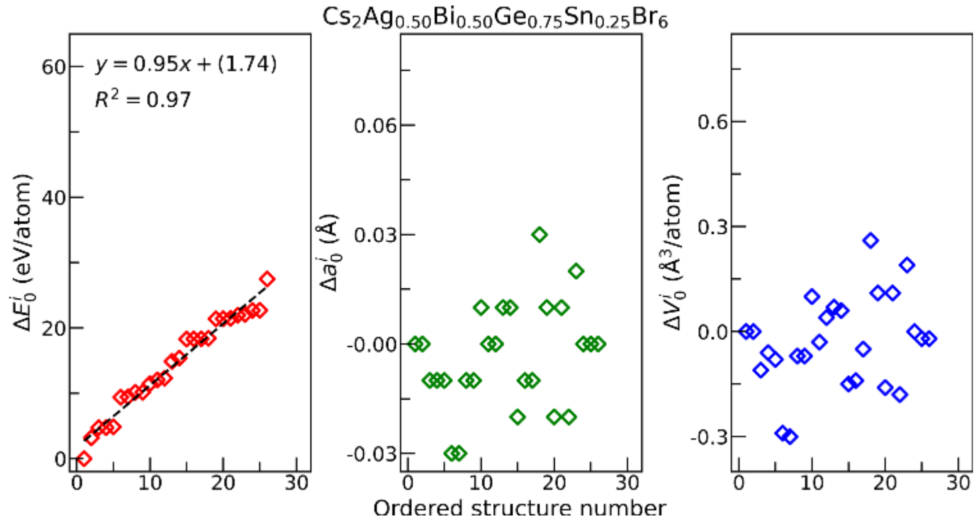

Figure S-40. Relative total energy (ΔE^i_{tot}), relative lattice constant at equilibrium (Δa^i_0), and relative equilibrium volume (ΔV^i_0), of the optimized structures of $Cs_2Ag_{0.50}Bi_{0.50}Ge_{0.75}Sn_{0.25}Br_6$ (or $Cs_8Ag_2Bi_2Ge_3SnBr_{24}$) with respect to the structure with the lowest energy; N_G = 26.

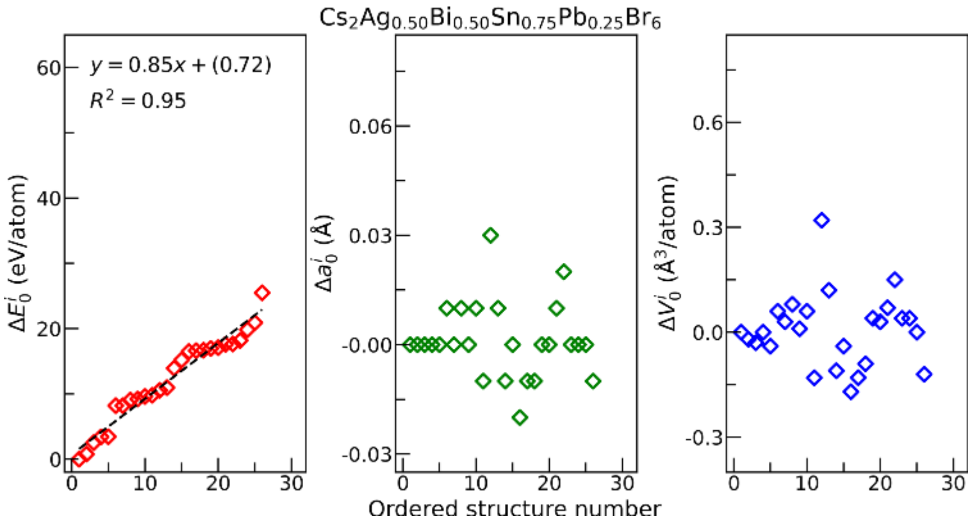

Figure S-41. Relative total energy (ΔE_{tot}^i), relative lattice constant at equilibrium (Δa_0^i), and relative equilibrium volume (ΔV_0^i), of the optimized structures of $Cs_2Ag_{0.50}Bi_{0.50}Sn_{0.75}Pb_{0.25}Br_6$ (or $Cs_8Ag_2Bi_2Sn_3PbBr_{24}$) with respect to the structure with the lowest energy; $N_G = 26$.

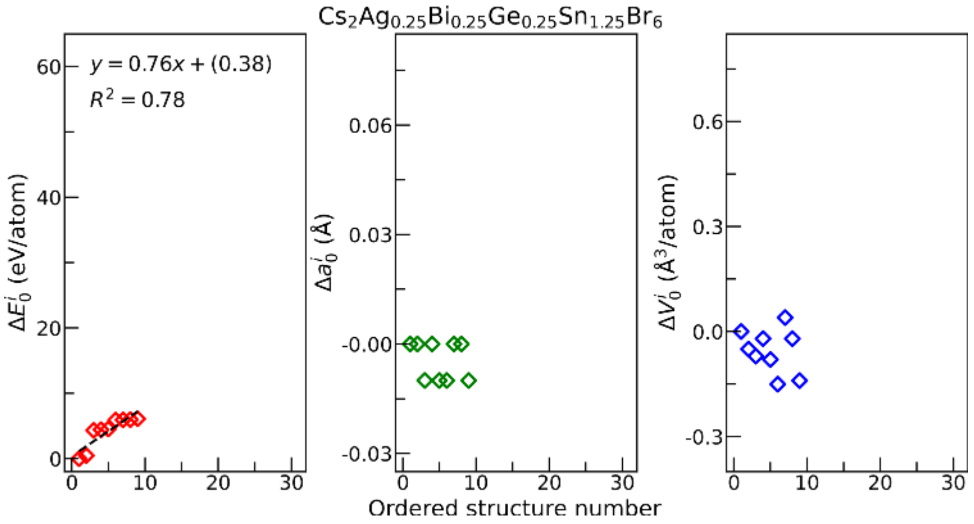

Figure S-42. Relative total energy (ΔE_{tot}^i), relative lattice constant at equilibrium (Δa_0^i), and relative equilibrium volume (ΔV_0^i), of the optimized structures of $Cs_2Ag_{0.25}Bi_{0.25}Ge_{0.25}Sn_{1.25}Br_6$ (or $Cs_8AgBiGeSn_5Br_{24}$) with respect to the structure with the lowest energy; $N_G = 9$.

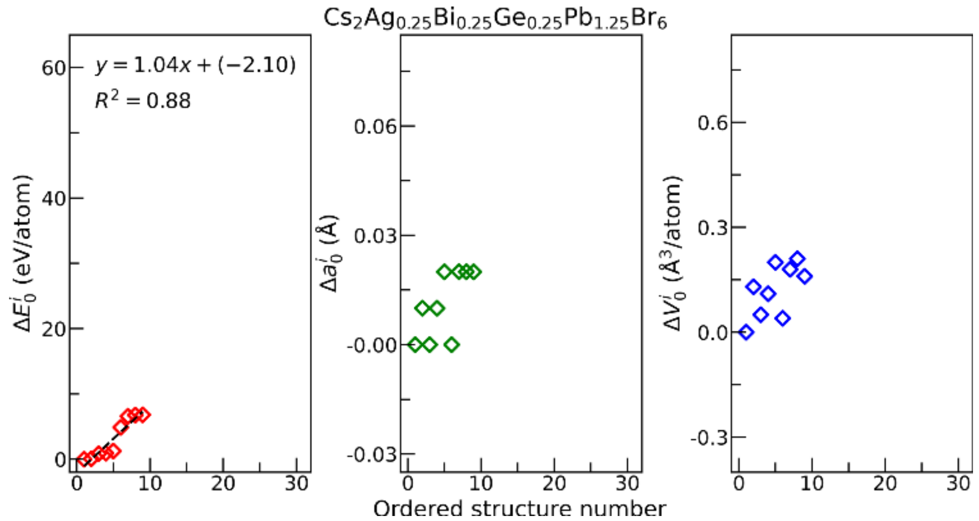

Figure S-43. Relative total energy (ΔE_{tot}^{i}), relative lattice constant at equilibrium (Δa_0^i), and relative equilibrium volume (ΔV_0^i), of the optimized structures of $Cs_2Ag_{0.25}Bi_{0.25}Ge_{0.25}Pb_{1.25}Br_6$ (or $Cs_8AgBiGePb_5Br_{24}$) with respect to the structure with the lowest energy; $N_G = 9$.

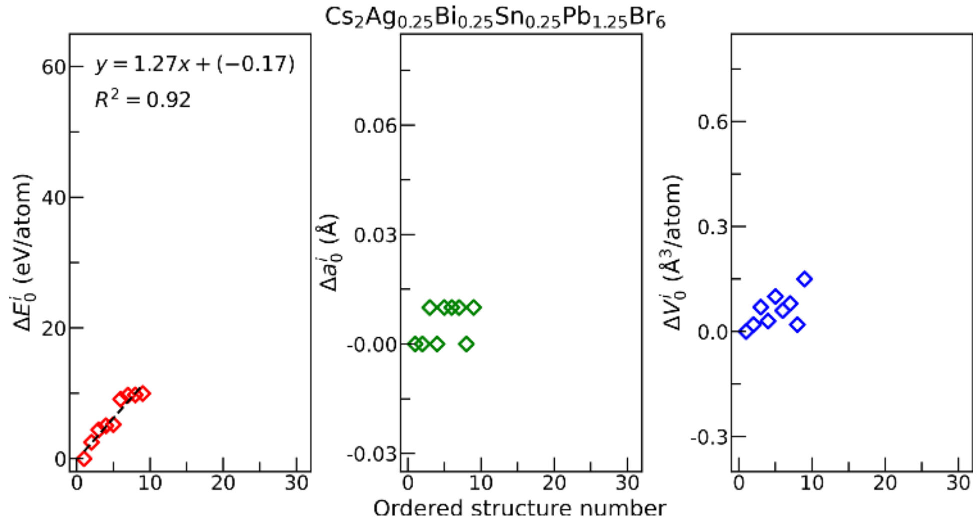

Figure S-44. Relative total energy (ΔE_{tot}^{i}), relative lattice constant at equilibrium (Δa_0^i), and relative equilibrium volume (ΔV_0^i), of the optimized structures of $Cs_2Ag_{0.25}Bi_{0.25}Sn_{0.25}Pb_{1.25}Br_6$ (or $Cs_8AgBiSnPb_5Br_{24}$) with respect to the structure with the lowest energy; $N_G = 9$.

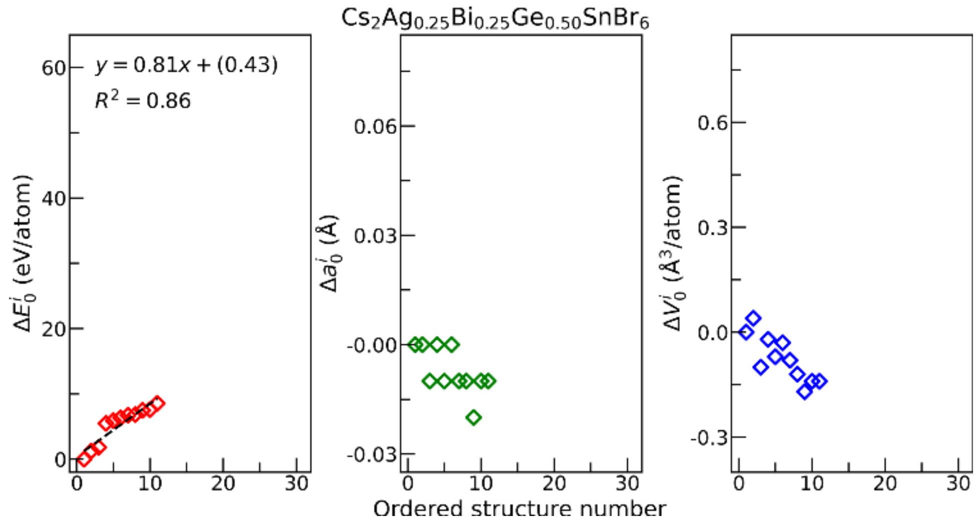

Figure S-45. Relative total energy (ΔE_{tot}^i), relative lattice constant at equilibrium (Δa_0^i), and relative equilibrium volume (ΔV_0^i), of the optimized structures of $Cs_2Ag_{0.25}Bi_{0.25}Ge_{0.50}Sn_{1.00}Br_6$ (or $Cs_8AgBiGe_2Sn_4Br_{24}$) with respect to the structure with the lowest energy; $N_G = 11$.

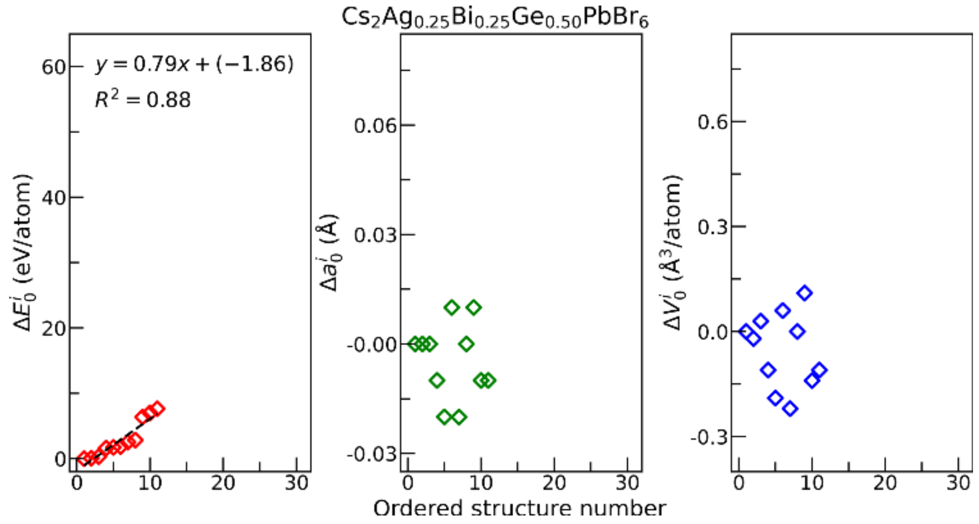

Figure S-46. Relative total energy (ΔE_{tot}^i), relative lattice constant at equilibrium (Δa_0^i), and relative equilibrium volume (ΔV_0^i), of the optimized structures of $Cs_2Ag_{0.25}Bi_{0.25}Ge_{0.50}Pb_{1.00}Br_6$ (or $Cs_8AgBiGe_2Pb_4Br_{24}$) with respect to the structure with the lowest energy; $N_G = 11$.

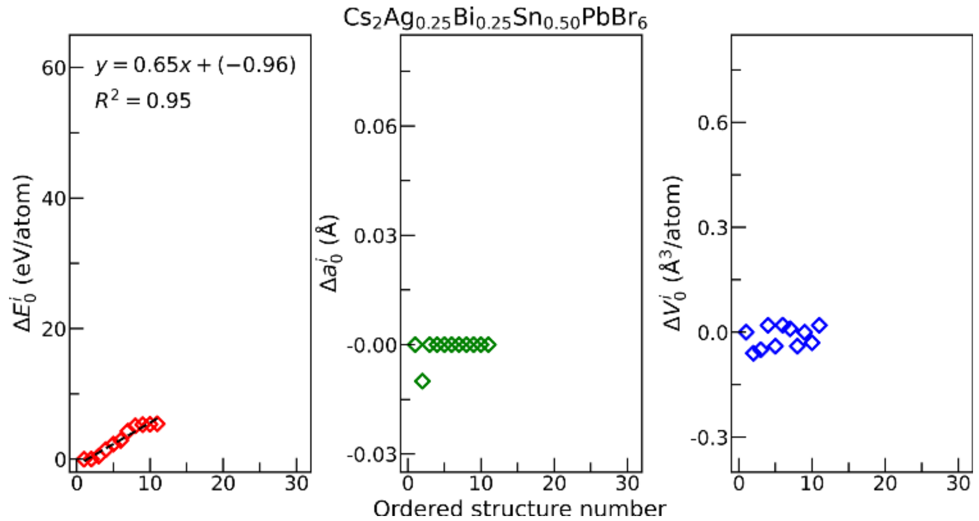

Figure S-47. Relative total energy (ΔE_{tot}^i), relative lattice constant at equilibrium (Δa_0^i), and relative equilibrium volume (ΔV_0^i), of the optimized structures of $Cs_2Ag_{0.25}Bi_{0.25}Sn_{0.50}Pb_{1.00}Br_6$ (or $Cs_8AgBiSn_2Pb_4Br_{24}$) with respect to the structure with the lowest energy; N_G = 11.

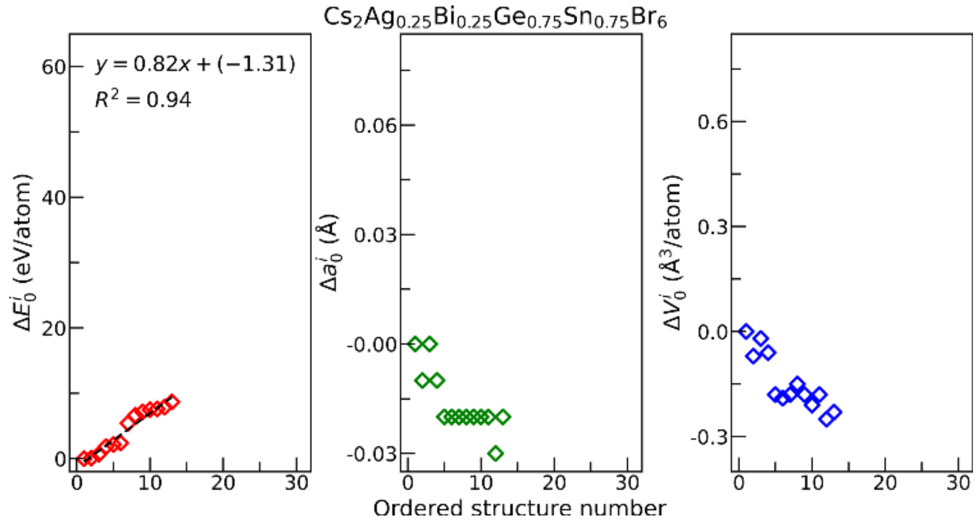

Figure S-48. Relative total energy (ΔE_{tot}^i), relative lattice constant at equilibrium (Δa_0^i), and relative equilibrium volume (ΔV_0^i), of the optimized structures of $Cs_2Ag_{0.25}Bi_{0.25}Ge_{0.75}Sn_{0.75}Br_6$ (or $Cs_8AgBiGe_3Sn_3Br_{24}$) with respect to the structure with the lowest energy; N_G = 13.

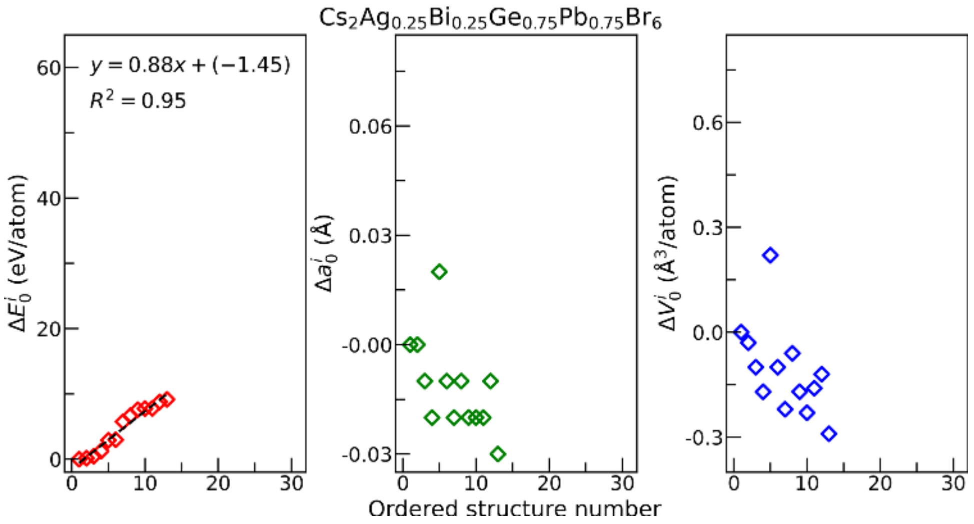

Figure S-49. Relative total energy (ΔE^{i}_{tot}), relative lattice constant at equilibrium (Δa^{i}_{0}), and relative equilibrium volume (ΔV^{i}_{0}), of the optimized structures of $Cs_2Ag_{0.25}Bi_{0.25}Ge_{0.75}Pb_{0.75}Br_6$ (or $Cs_8AgBiGe_3Pb_3Br_{24}$) with respect to the structure with the lowest energy; N_G = 13.

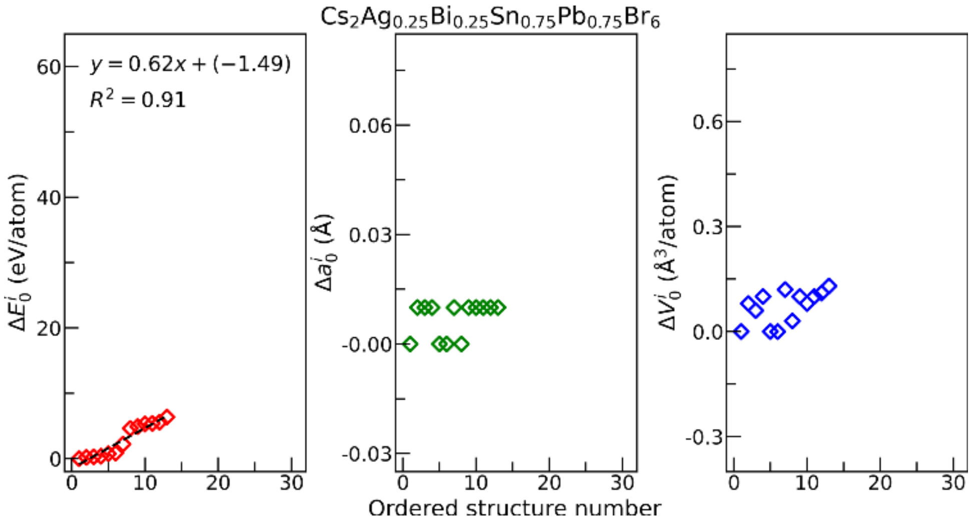

Figure S-50. Relative total energy (ΔE^{i}_{tot}), relative lattice constant at equilibrium (Δa^{i}_{0}), and relative equilibrium volume (ΔV^{i}_{0}), of the optimized structures of $Cs_2Ag_{0.25}Bi_{0.25}Sn_{0.75}Pb_{0.75}Br_6$ (or $Cs_8AgBiSn_3Pb_3Br_{24}$) with respect to the structure with the lowest energy; N_G = 13.

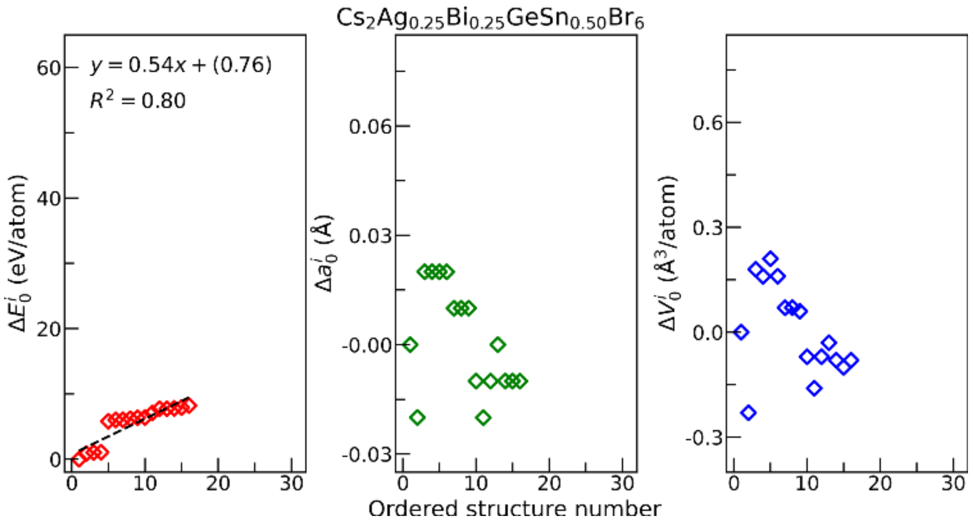

Figure S-51. Relative total energy (ΔE_{tot}^{i}), relative lattice constant at equilibrium (Δa_0^i), and relative equilibrium volume (ΔV_0^i), of the optimized structures of $Cs_2Ag_{0.25}Bi_{0.25}Ge_{1.00}Sn_{0.50}Br_6$ (or $Cs_8AgBiGe_4Sn_2Br_{24}$) with respect to the structure with the lowest energy; N_G = 16.

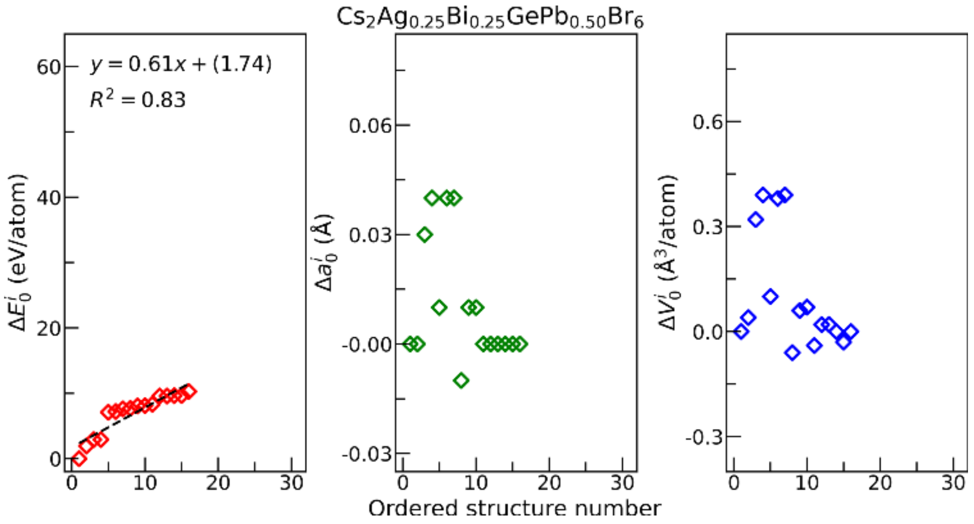

Figure S-52. Relative total energy (ΔE_{tot}^{i}), relative lattice constant at equilibrium (Δa_0^i), and relative equilibrium volume (ΔV_0^i), of the optimized structures of $Cs_2Ag_{0.25}Bi_{0.25}Ge_{1.00}Pb_{0.50}Br_6$ (or $Cs_8AgBiGe_4Pb_2Br_{24}$) with respect to the structure with the lowest energy; N_G = 16.

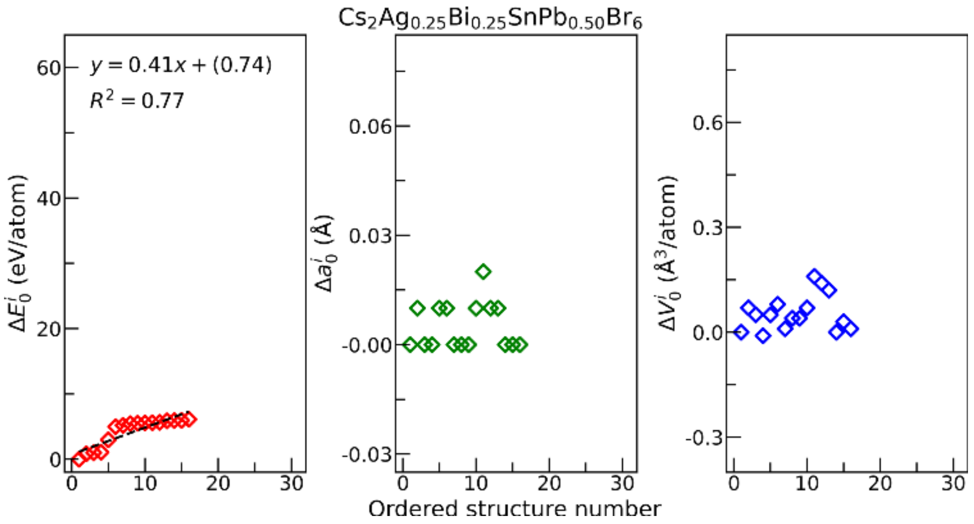

Figure S-53. Relative total energy (ΔE_{tot}^i), relative lattice constant at equilibrium (Δa_0^i), and relative equilibrium volume (ΔV_0^i), of the optimized structures of $Cs_2Ag_{0.25}Bi_{0.25}Sn_{1.00}Pb_{0.50}Br_6$ (or $Cs_8AgBiSn_4Pb_2Br_{24}$) with respect to the structure with the lowest energy; N_G = 16.

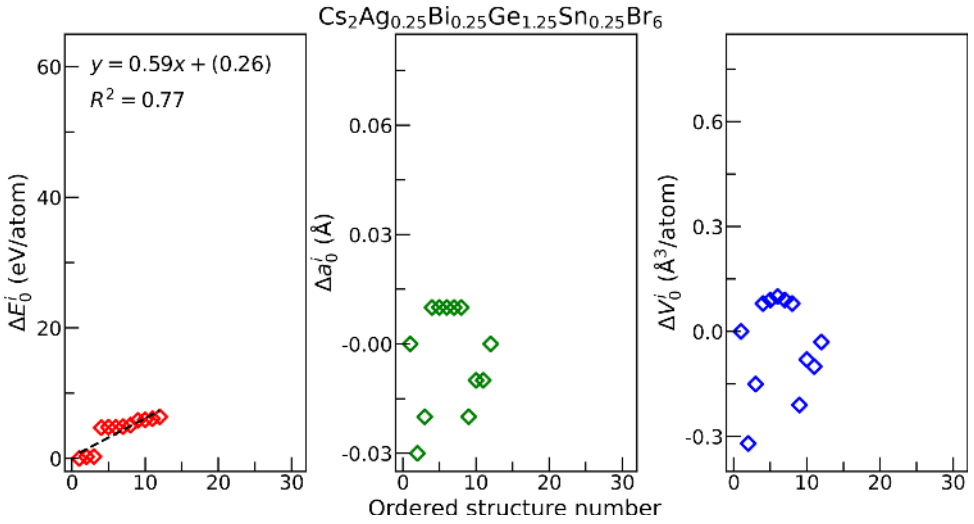

Figure S-54. Relative total energy (ΔE_{tot}^i), relative lattice constant at equilibrium (Δa_0^i), and relative equilibrium volume (ΔV_0^i), of the optimized structures of $Cs_2Ag_{0.25}Bi_{0.25}Ge_{1.25}Sn_{0.25}Br_6$ (or $Cs_8AgBiGe_5SnBr_{24}$) with respect to the structure with the lowest energy; N_G = 12.

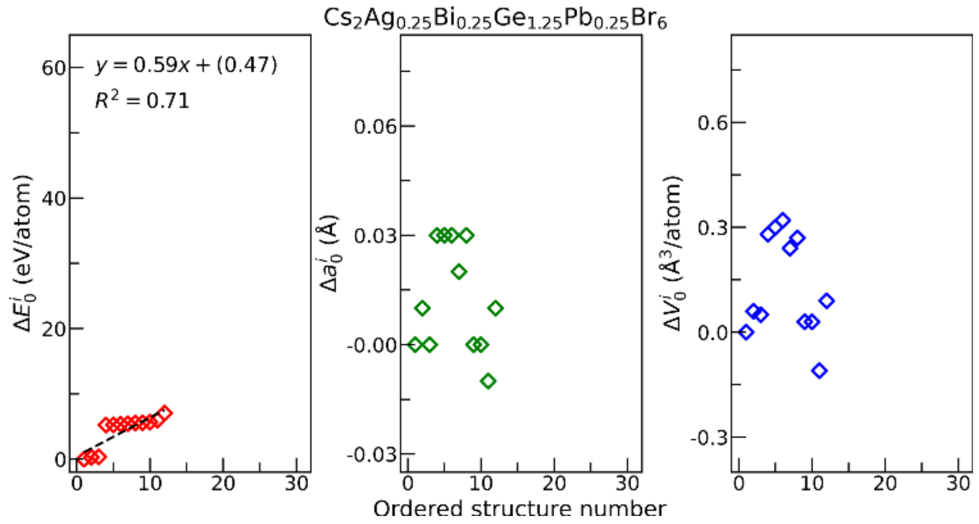

Figure S-55. Relative total energy (ΔE_{tot}^i), relative lattice constant at equilibrium (Δa_0^i), and relative equilibrium volume (ΔV_0^i), of the optimized structures of $Cs_2Ag_{0.25}Bi_{0.25}Ge_{1.25}Pb_{0.25}Br_6$ (or $Cs_8AgBiGe_5PbBr_{24}$) with respect to the structure with the lowest energy; N_G = 12.

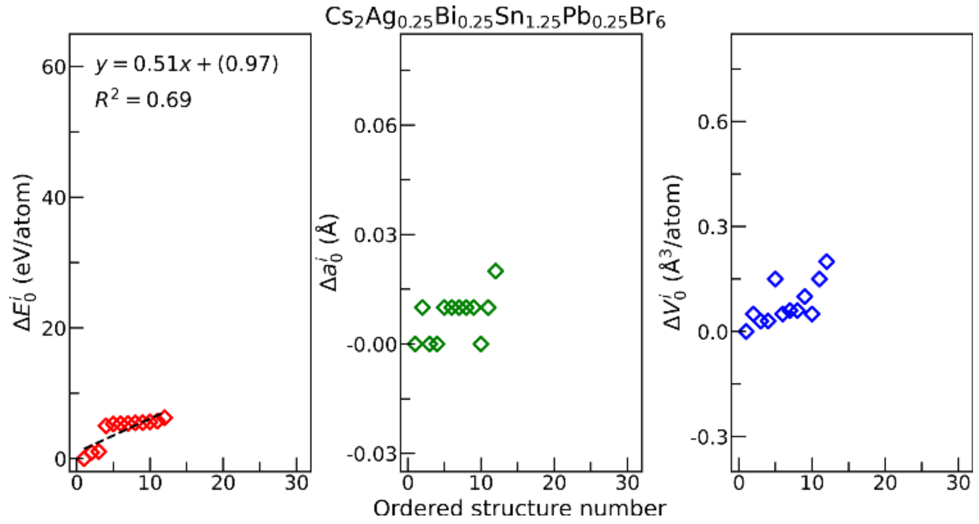

Figure S-56. Relative total energy (ΔE_{tot}^i), relative lattice constant at equilibrium (Δa_0^i), and relative equilibrium volume (ΔV_0^i), of the optimized structures of $Cs_2Ag_{0.25}Bi_{0.25}Sn_{1.25}Pb_{0.25}Br_6$ (or $Cs_8AgBiSn_5PbBr_{24}$) with respect to the structure with the lowest energy; N_G = 12.

S-7 CONVERGENCE OF THE K-MESH FOR DENSITY OF STATES AND COHP CALCLUATIONS

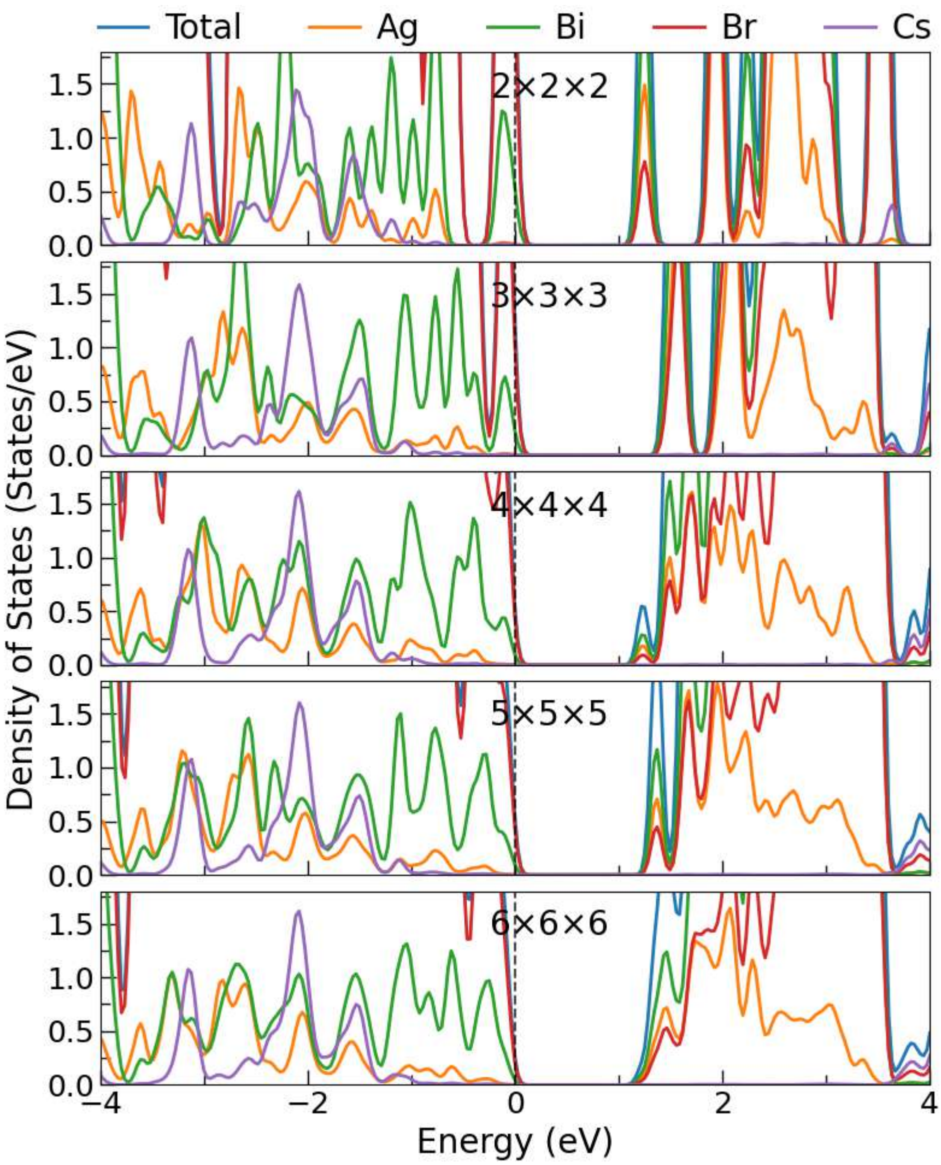

Figure S-57. Density of states for each species of $Cs_2AgBiBr_6$ and for different samplings of **k**-points within the first Brillouin zone.

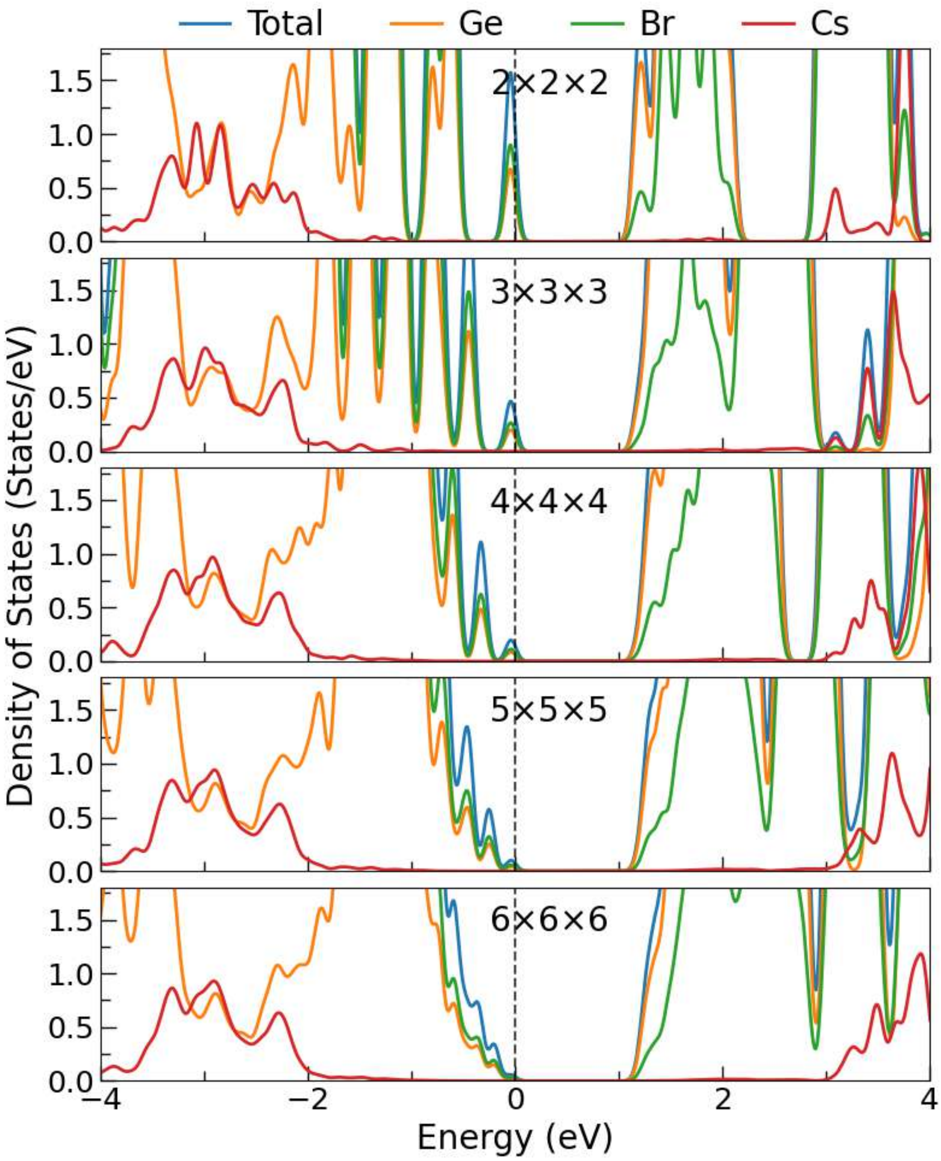

Figure S-58. Density of states for each species of $CsGeBr_3$ and for different samplings of **k**-points within the first Brillouin zone.

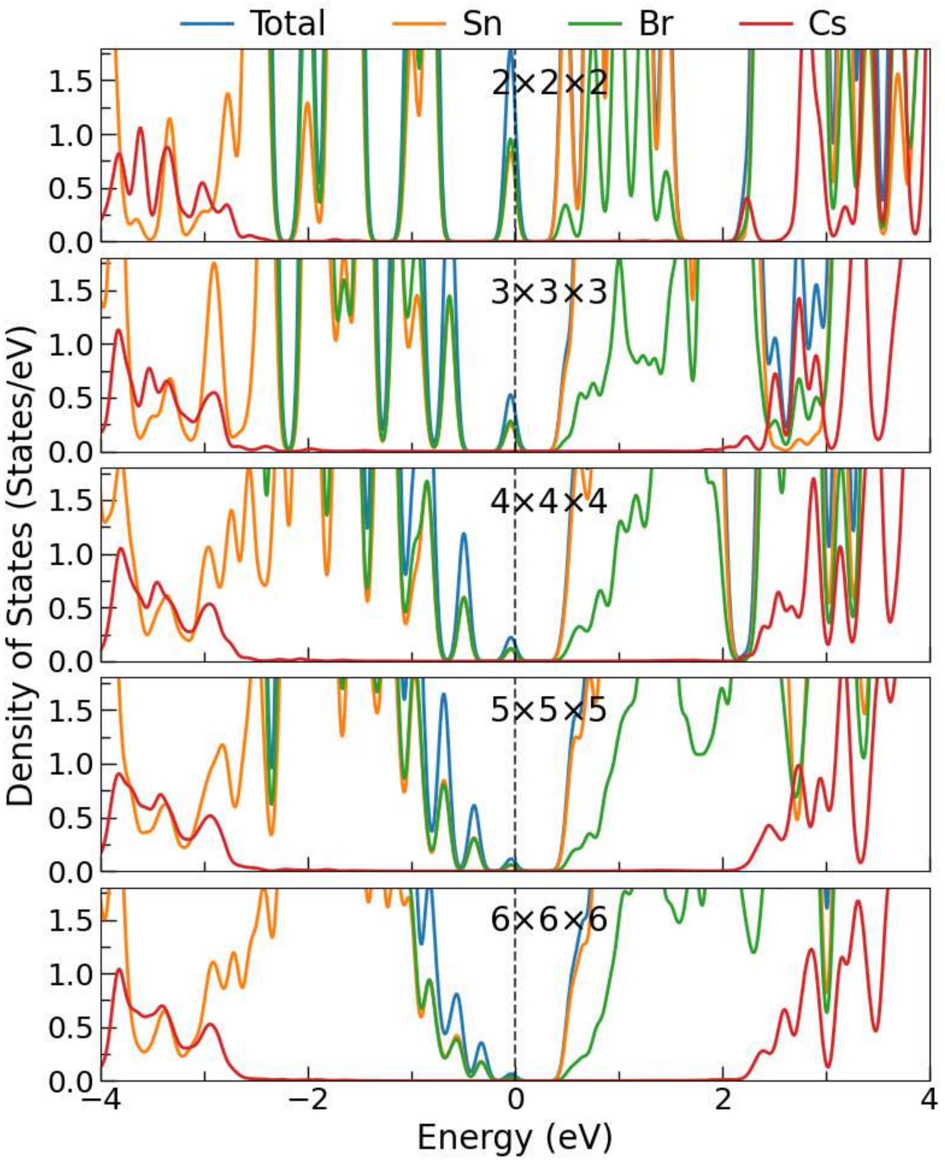

Figure S-59. Density of states for each species of $CsSnBr_3$ and for different samplings of **k**-points within the first Brillouin zone.

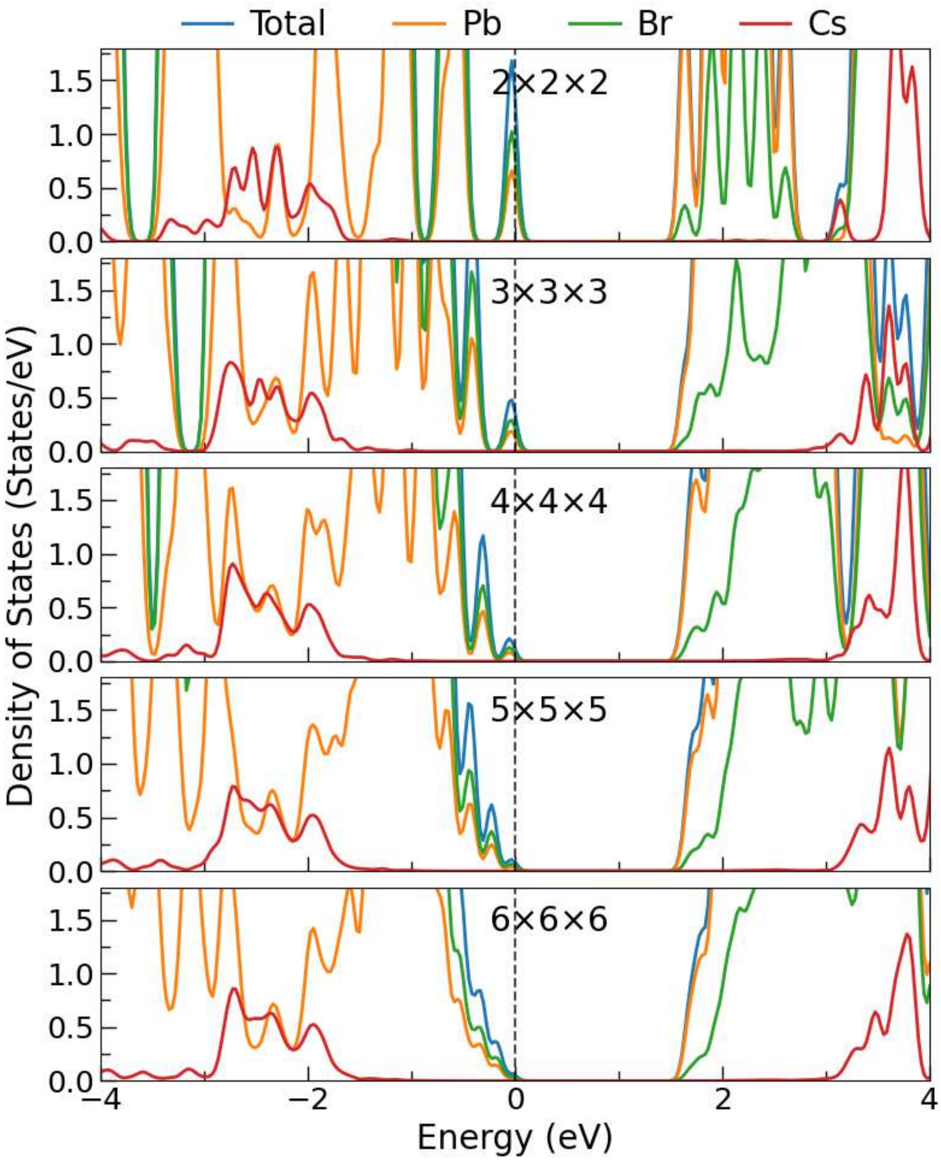

Figure S-60. Density of states for each species of $CsPbBr_3$ and for different samplings of **k**-points within the first Brillouin zone.

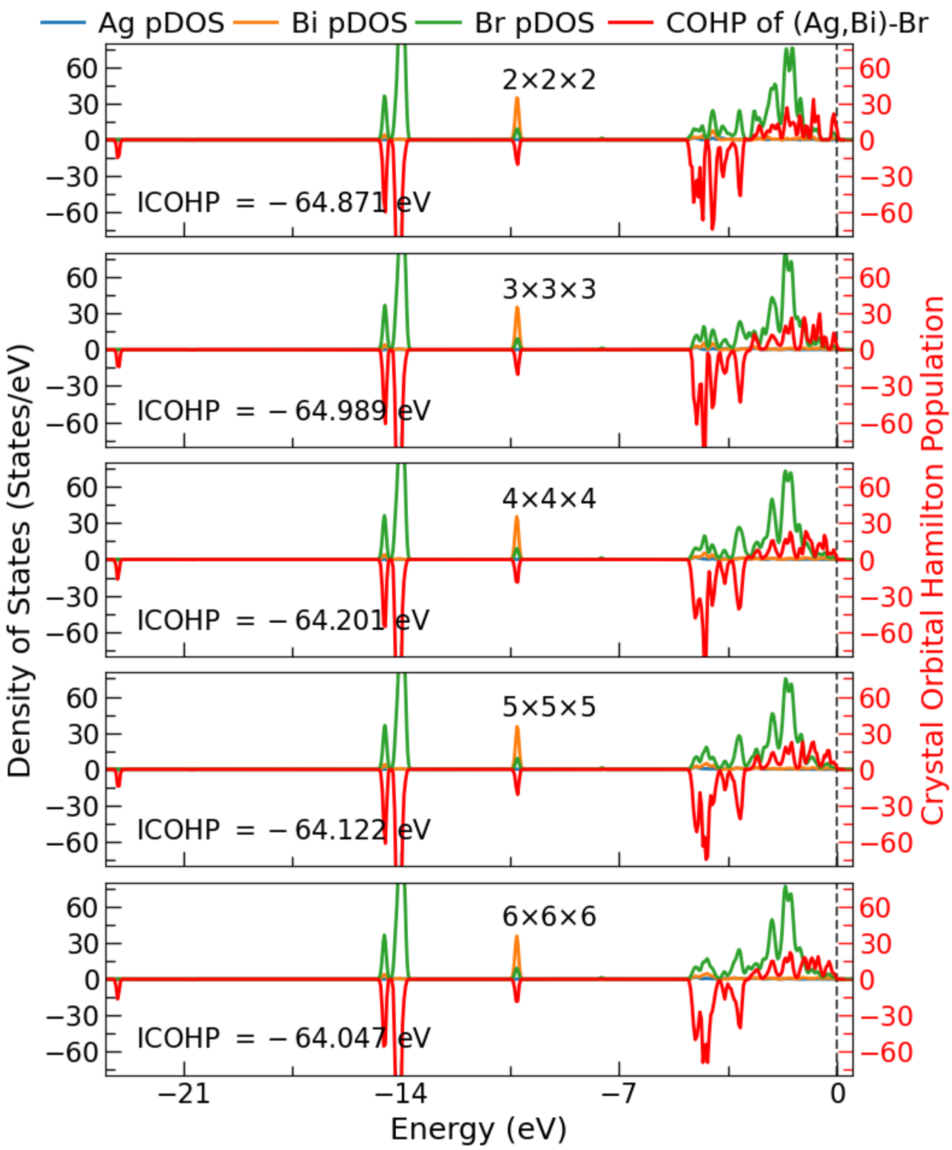

Figure S-61. Projected density of states (pDOS) of Ag, Bi, and Br, and Crystal Orbital Hamilton Population (COHP) of both Ag – Br and Bi – Br metal-halide bonds, or simply (Ag,Bi) – Br of $Cs_2AgBiBr_6$ and for different samplings of **k**-points within the first Brillouin zone. Integrated COHP (ICOHP) energy values of (Ag,Bi) – Br bonds are also shown for each **k**-mesh.

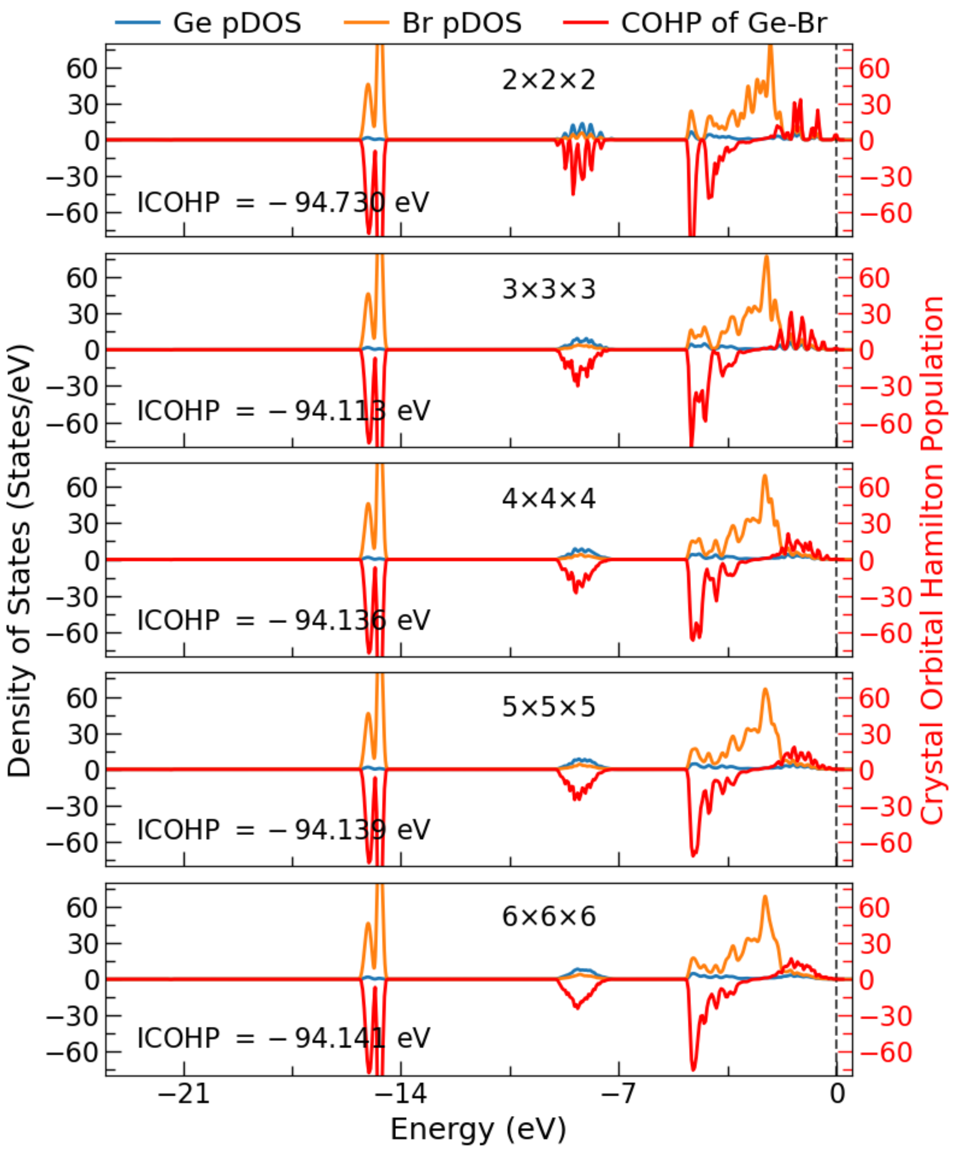

Figure S-62. Projected density of states (pDOS) of Ge and Br, and Crystal Orbital Hamilton Population (COHP) of Ge – Br metal-halide bonds of $CsGeBr_3$ and for different samplings of **k**-points within the first Brillouin zone. Integrated COHP (ICOHP) energy values of Ge – Br bonds are also shown for each **k**-mesh.

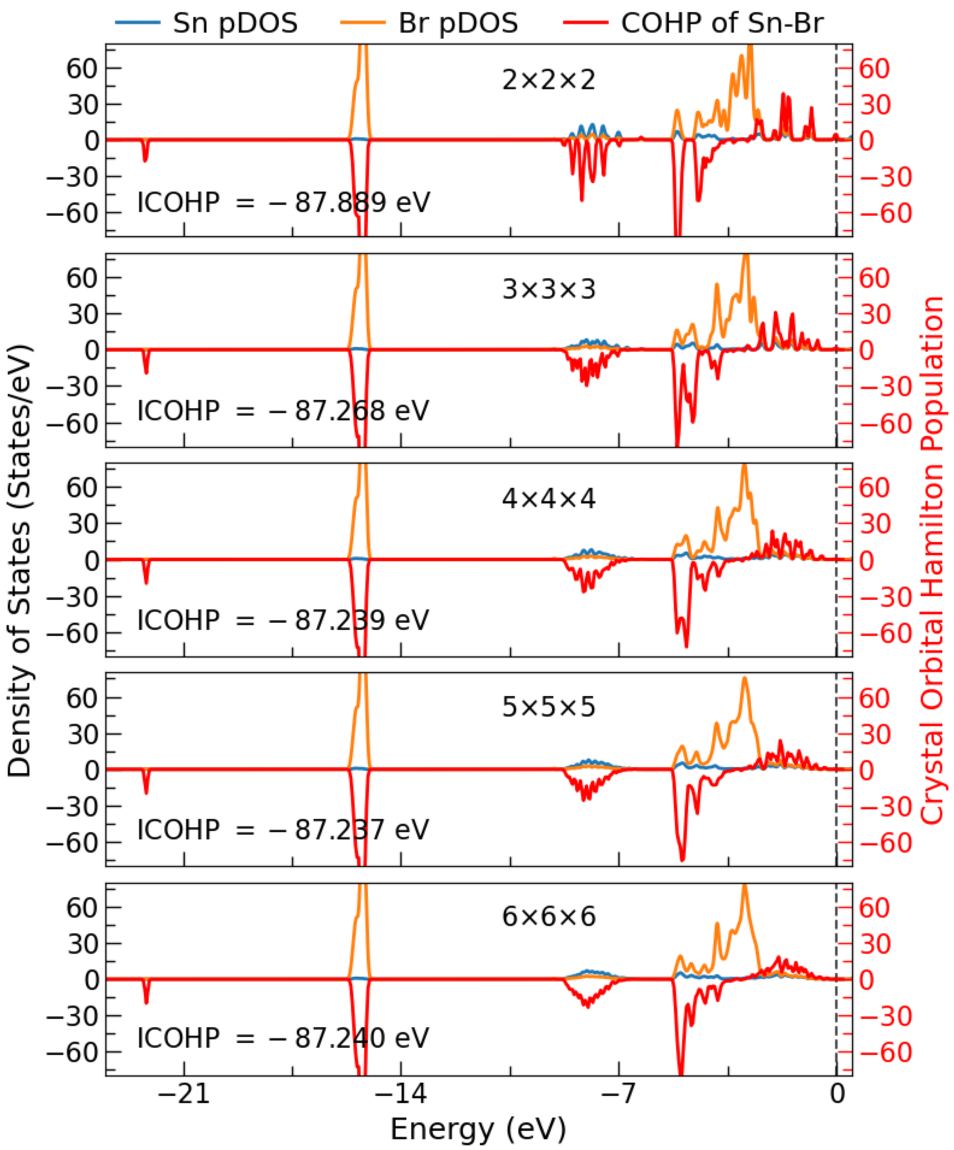

Figure S-63. Projected density of states (pDOS) of Sn and Br, and Crystal Orbital Hamilton Population (COHP) of Sn – Br metal-halide bonds of $CsSnBr_3$ and for different samplings of **k**-points within the first Brillouin zone. Integrated COHP (ICOHP) energy values of Sn – Br bonds are also shown for each **k**-mesh.

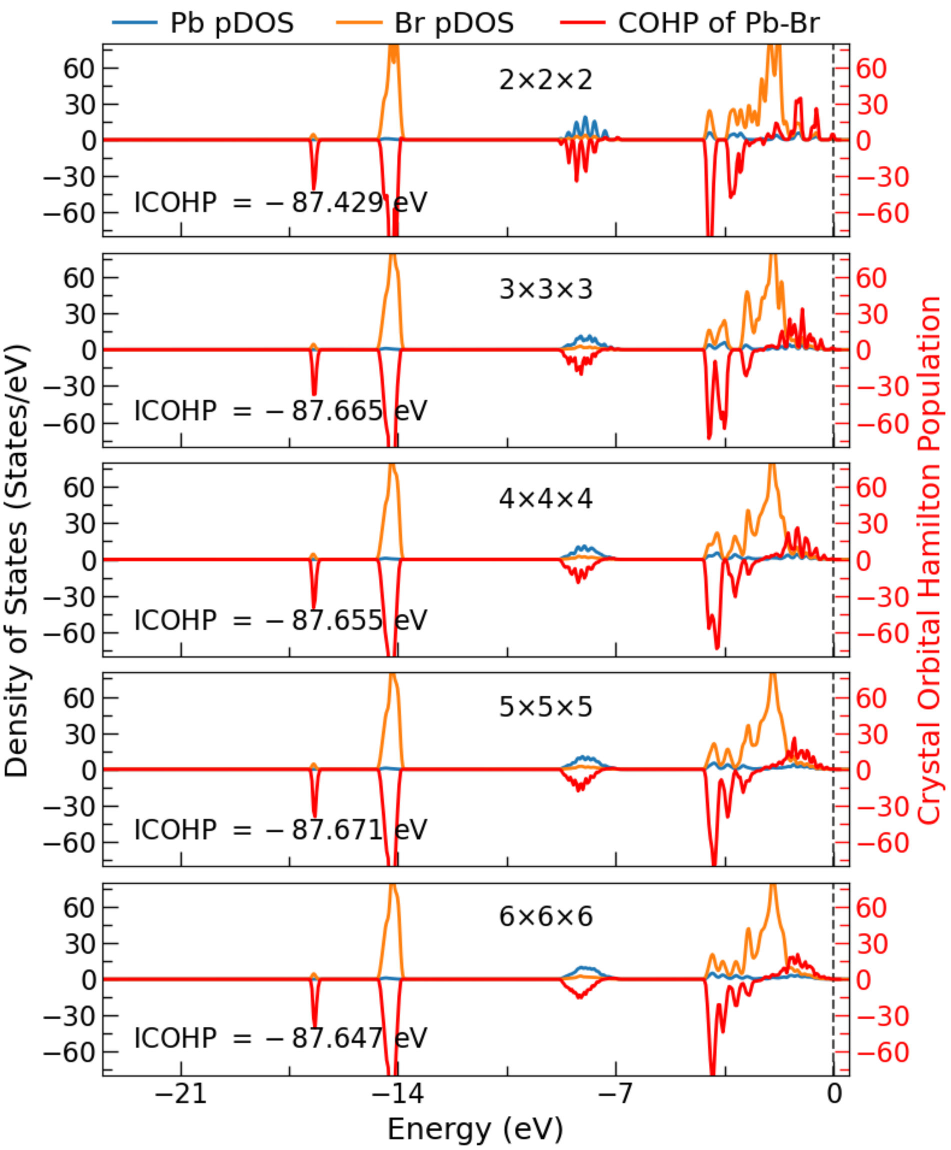

Figure S-64. Projected density of states (pDOS) of Pb and Br, and Crystal Orbital Hamilton Population (COHP) of Pb – Br metal-halide bonds of $CsPbBr_3$ and for different samplings of **k**-points within the first Brillouin zone. Integrated COHP (ICOHP) energy values of Pb – Br bonds are also shown for each **k**-mesh.

S-8 ADDITIONAL DATA ON THE PHYSICAL-CHEMICAL PROPERTIES

S-8.1 Energy of Atomic Species

Table S-11. Total energy (E_{tot}), magnetic moment (m), energy of both the highest occupied energy level (HOEL) and lowest unoccupied energy level (LUEL) identified as E_{HOEL} and E_{LUEL}, respectively, and occupation number of both the HOEL ($N^{occ.}_{\text{HOEL}}$) and LUEL ($N^{occ.}_{\text{LUEL}}$).

Atom	E_{tot}	m	E_{HOEL}	$N^{occ.}_{\text{HOEL}}$	E_{LUEL}	$N^{occ.}_{\text{LUEL}}$
Ag	−0.198 859	1.000	−4.6669	1.000	−3.8923	0.000
Bi	−1.349 430	3.000	−5.1025	1.000	−3.2841	0.000
Br	−0.269 834	1.000	−7.3722	1.000	−7.1385	0.000
Cs	−1.811 099	1.000	−2.1417	1.000	−1.4268	0.000
Ge	−0.796 430	2.000	−4.3304	1.000	−4.1821	0.000
Sn	−0.674 819	2.000	−4.0405	1.000	−3.9546	0.000
Pb	−0.615 211	2.000	−3.8341	1.000	−3.7802	0.000

S-8.2 Energy Analysis of the Lowest Structures

Table S-12. Total energy (E_{tot}), excess energy (E_{exc}), cohesive energy (E_{coh}), and energy of the cumulative integrated crystal orbital Hamilton population (E_{CICOHP}) of the metal-halide bonds ($B-X$) only. E_{tot} is given in electronvolts (eV), E_{exc} is given in eV (first column) and meV/Cs (second column), E_{coh} is given in eV (first column) and eV/atom (second column), and E_{CICOHP} is given in eV (first column) and eV/$B-X$ (second column), which means, electronvolts per metal-halide bond. All energies were calculated only for the lowest-structure configuration of each compound (identified here by the metal composition where the subscripts indicate the number of atoms within the unit-cell).

Metal composition	E_{tot}	E_{exc}		E_{coh}		E_{CICOHP}	
Ag_4Bi_4	−142.721 303	0.000	0.0	−115.56	−2.89	−65.08	−1.36
Ge_8	−150.596 386	0.000	0.0	−123.26	−3.08	−95.14	−1.98
Sn_8	−149.189 459	0.000	0.0	−122.83	−3.07	−88.24	−1.84
Pb_8	−149.830 275	0.000	0.0	−123.94	−3.10	−87.65	−1.83
$Ag_3Bi_3Ge_2$	−144.243 780	0.112	56.0	−117.04	−2.93	−72.04	−1.50
$Ag_3Bi_3Sn_2$	−143.965 636	0.093	46.5	−117.01	−2.93	−71.50	−1.49
$Ag_3Bi_3Pb_2$	−144.026 719	0.118	59.0	−117.19	−2.93	−71.55	−1.49
$Ag_2Bi_2Ge_4$	−145.840 512	0.205	102.5	−118.59	−2.96	−79.36	−1.65

Continued on next page

Table S-12 – Continued from previous page

Metal composition	E_{tot}	E_{exc}		E_{coh}		E_{CICOHP}	
$Ag_2Bi_2Sn_4$	−145.231 428	0.181	90.5	−118.47	−2.96	−78.04	−1.63
$Ag_2Bi_2Pb_4$	−145.565 596	0.178	89.0	−119.04	−2.98	−73.66	−1.53
$AgBiGe_6$	−148.211 053	0.104	52.0	−120.92	−3.02	−87.20	−1.82
$AgBiSn_6$	−147.173 587	0.100	50.0	−120.61	−3.02	−83.06	−1.73
$AgBiPb_6$	−147.768 541	0.071	35.5	−121.56	−3.04	−79.62	−1.66
Ag_3Bi_3GeSn	−144.156 503	0.089	44.5	−117.08	−2.93	−71.79	−1.50
Ag_3Bi_3GePb	−144.243 466	0.088	44.0	−117.22	−2.93	−71.53	−1.49
Ag_3Bi_3SnPb	−144.045 965	0.093	46.5	−117.15	−2.93	−71.56	−1.49
$Ag_2Bi_2GeSn_3$	−145.755 297	0.094	47.0	−118.87	−2.97	−78.05	−1.63
$Ag_2Bi_2GePb_3$	−145.873 286	0.125	62.5	−119.17	−2.98	−77.67	−1.62
$Ag_2Bi_2SnPb_3$	−145.912 067	0.071	35.5	−119.33	−2.98	−74.55	−1.55
$Ag_2Bi_2Ge_2Sn_2$	−145.867 275	0.110	55.0	−118.86	−2.97	−78.53	−1.64
$Ag_2Bi_2Ge_2Pb_2$	−145.915 708	0.138	69.0	−119.03	−2.98	−78.33	−1.63
$Ag_2Bi_2Sn_2Pb_2$	−145.655 254	0.115	57.5	−119.01	−2.98	−77.23	−1.61
$Ag_2Bi_2Ge_3Sn$	−146.011 729	0.118	59.0	−118.89	−2.97	−79.11	−1.65
$Ag_2Bi_2Ge_3Pb$	−146.007 773	0.139	69.5	−118.94	−2.97	−78.86	−1.64
$Ag_2Bi_2Sn_3Pb$	−145.578 870	0.114	57.0	−118.88	−2.97	−77.48	−1.61
$AgBiGeSn_5$	−147.308 722	0.110	55.0	−120.63	−3.02	−83.98	−1.75
$AgBiGePb_5$	−147.575 013	0.143	71.5	−121.19	−3.03	−79.86	−1.66
$AgBiSnPb_5$	−147.673 676	0.075	37.5	−121.41	−3.04	−79.55	−1.66
$AgBiGe_2Sn_4$	−147.505 338	0.105	52.5	−120.70	−3.02	−85.01	−1.77
$AgBiGe_2Pb_4$	−147.678 043	0.142	71.0	−121.11	−3.03	−80.20	−1.67
$AgBiSn_2Pb_4$	−147.420 846	0.118	59.0	−121.10	−3.03	−82.68	−1.72
$AgBiGe_3Sn_3$	−147.666 178	0.108	54.0	−120.74	−3.02	−85.25	−1.78
$AgBiGe_3Pb_3$	−147.798 303	0.136	68.0	−121.05	−3.03	−84.88	−1.77
$AgBiSn_3Pb_3$	−147.361 932	0.113	56.5	−120.98	−3.02	−82.70	−1.72
$AgBiGe_4Sn_2$	−147.854 411	0.105	52.5	−120.81	−3.02	−85.60	−1.78
$AgBiGe_4Pb_2$	−147.982 340	0.113	56.5	−121.05	−3.03	−85.34	−1.78
$AgBiSn_4Pb_2$	−147.320 549	0.103	51.5	−120.88	−3.02	−82.91	−1.73
$AgBiGe_5Sn$	−147.980 873	0.118	59.0	−120.81	−3.02	−86.13	−1.79
$AgBiGe_5Pb$	−148.018 624	0.128	64.0	−120.91	−3.02	−85.79	−1.79
$AgBiSn_5Pb$	−147.249 324	0.101	50.5	−120.75	−3.02	−82.95	−1.73

S-8.2.1 Further Energy Analysis of the Lowest Structures: Cumulative ICOHP

Table S-13. Energy of the cumulative integrated crystal orbital Hamilton population (E_{CICOHP}) of each metal-halide bond type Ag – Br, Bi – Br, Ge – Br, and Sn – Br, and Pb – Br within each perovskite mixture. Therefore, all energy values (given in eV) indicate the total covalent energy attributed to each metal-halide bond type per unit-cell. All energies were calculated only for the lowest-structure configuration of each compound (identified here by the metal composition where the subscripts indicate the number of atoms within the unit-cell).

Metal composition	Ag – Br	Bi – Br	Ge – Br	Sn – Br	Pb – Br	Total
Ag_4Bi_4	−20.17	−44.91	0.00	0.00	0.00	−65.08
Ge_8	0.00	0.00	−95.14	0.00	0.00	−95.14
Sn_8	0.00	0.00	0.00	−88.24	0.00	−88.24
Pb_8	0.00	0.00	0.00	0.00	−87.65	−87.65
$Ag_3Bi_3Ge_2$	−14.69	−34.40	−22.95	0.00	0.00	−72.04
$Ag_3Bi_3Sn_2$	−14.33	−34.26	0.00	−22.90	0.00	−71.50
$Ag_3Bi_3Pb_2$	−14.21	−34.47	0.00	0.00	−22.87	−71.55
$Ag_2Bi_2Ge_4$	−9.14	−24.00	−46.22	0.00	0.00	−79.36
$Ag_2Bi_2Sn_4$	−8.95	−23.85	0.00	−45.24	0.00	−78.04
$Ag_2Bi_2Pb_4$	−8.74	−23.34	0.00	0.00	−41.57	−73.66
$AgBiGe_6$	−4.59	−11.86	−70.75	0.00	0.00	−87.20
$AgBiSn_6$	−4.39	−11.70	0.00	−66.96	0.00	−83.06
$AgBiPb_6$	−4.37	−11.83	0.00	0.00	−63.43	−79.62
Ag_3Bi_3GeSn	−14.75	−34.31	−12.17	−10.56	0.00	−71.79
Ag_3Bi_3GePb	−14.66	−34.26	−12.15	0.00	−10.45	−71.53
Ag_3Bi_3SnPb	−14.36	−34.24	0.00	−12.46	−10.51	−71.56
$Ag_2Bi_2GeSn_3$	−9.42	−22.98	−11.78	−33.87	0.00	−78.05
$Ag_2Bi_2GePb_3$	−9.38	−23.08	−11.61	0.00	−33.60	−77.67
$Ag_2Bi_2SnPb_3$	−9.23	−22.97	0.00	−11.16	−31.20	−74.55
$Ag_2Bi_2Ge_2Sn_2$	−9.44	−23.05	−23.00	−23.04	0.00	−78.53
$Ag_2Bi_2Ge_2Pb_2$	−9.35	−23.22	−22.88	0.00	−22.89	−78.33
$Ag_2Bi_2Sn_2Pb_2$	−9.17	−22.98	0.00	−22.51	−22.55	−77.23
$Ag_2Bi_2Ge_3Sn$	−9.48	−23.16	−34.47	−12.01	0.00	−79.11
$Ag_2Bi_2Ge_3Pb$	−9.41	−23.29	−34.33	0.00	−11.84	−78.86
$Ag_2Bi_2Sn_3Pb$	−9.20	−23.07	0.00	−33.47	−11.74	−77.48
$AgBiGeSn_5$	−4.43	−11.79	−11.51	−56.26	0.00	−83.98
$AgBiGePb_5$	−4.34	−11.85	−11.33	0.00	−52.33	−79.86
$AgBiSnPb_5$	−4.39	−11.58	0.00	−11.05	−52.53	−79.55
$AgBiGe_2Sn_4$	−4.51	−11.87	−23.23	−45.39	0.00	−85.01
$AgBiGe_2Pb_4$	−4.31	−11.93	−22.40	0.00	−41.56	−80.20

Continued on next page

Table S-13 – Continued from previous page

Metal composition	Ag−Br	Bi−Br	Ge−Br	Sn−Br	Pb−Br	Total
$AgBiSn_2Pb_4$	−4.36	−11.93	0.00	−22.30	−44.09	−82.68
$AgBiGe_3Sn_3$	−4.57	−11.82	−35.13	−33.74	0.00	−85.25
$AgBiGe_3Pb_3$	−4.59	−11.96	−35.04	0.00	−33.29	−84.88
$AgBiSn_3Pb_3$	−4.41	−11.77	0.00	−33.74	−32.77	−82.70
$AgBiGe_4Sn_2$	−4.65	−11.79	−47.27	−21.89	0.00	−85.60
$AgBiGe_4Pb_2$	−4.70	−11.78	−47.25	0.00	−21.61	−85.34
$AgBiSn_4Pb_2$	−4.43	−11.67	0.00	−45.35	−21.45	−82.91
$AgBiGe_5Sn$	−4.50	−11.79	−58.33	−11.50	0.00	−86.13
$AgBiGe_5Pb$	−4.51	−11.83	−58.26	0.00	−11.20	−85.79
$AgBiSn_5Pb$	−4.37	−11.62	0.00	−55.77	−11.18	−82.95

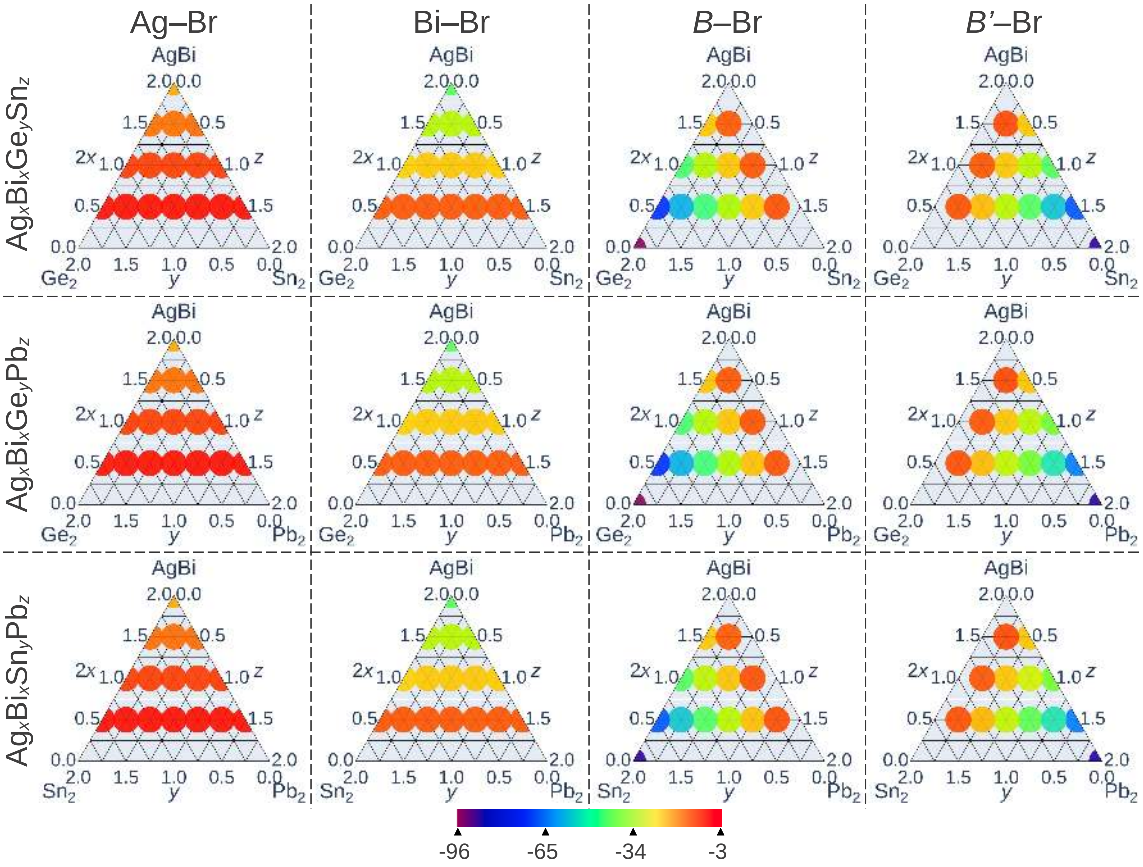

Figure S-65. Diagram of the CICOHP energies for each mixture studied and each metal-halide bond type. Based on the chemical formula $Cs_2Ag_xBi_yB_yB'_zBr_6$ only the metal composition is given at the left. For $Ag_xBi_yGe_ySn_z$: B = Ge and B' = Sn; $Ag_xBi_yGe_yPb_z$: B = Ge and B' = Pb; and $Ag_xBi_ySn_yPb_z$: B = Sn and B' = Pb. All energies are given in eV.

Table S-14. Average energy of the cumulative integrated crystal orbital Hamilton population (E_{CICOHP}) of each metal-halide bond type Ag–Br, Bi–Br, Ge–Br, and Sn–Br, and Pb–Br within each perovskite mixture. Therefore, all energy values (given in eV) indicate the average covalent energy attributed to each metal-halide bond type. All energies were calculated only for the lowest-structure configuration of each compound (identified here by the metal composition where the subscripts indicate the number of atoms within the unit-cell).

Metal composition	Ag–Br	Bi–Br	Ge–Br	Sn–Br	Pb–Br	Total/48
Ag_4Bi_4	−0.84	−1.87	0.00	0.00	0.00	−1.36
Ge_8	0.00	0.00	−1.98	0.00	0.00	−1.98
Sn_8	0.00	0.00	0.00	−1.84	0.00	−1.84
Pb_8	0.00	0.00	0.00	0.00	−1.83	−1.83
$Ag_3Bi_3Ge_2$	−0.82	−1.91	−1.91	0.00	0.00	−1.50
$Ag_3Bi_3Sn_2$	−0.80	−1.90	0.00	−1.91	0.00	−1.49
$Ag_3Bi_3Pb_2$	−0.79	−1.92	0.00	0.00	−1.91	−1.49
$Ag_2Bi_2Ge_4$	−0.76	−2.00	−1.93	0.00	0.00	−1.65
$Ag_2Bi_2Sn_4$	−0.75	−1.99	0.00	−1.88	0.00	−1.63
$Ag_2Bi_2Pb_4$	−0.73	−1.95	0.00	0.00	−1.73	−1.53
$AgBiGe_6$	−0.76	−1.98	−1.97	0.00	0.00	−1.82
$AgBiSn_6$	−0.73	−1.95	0.00	−1.86	0.00	−1.73
$AgBiPb_6$	−0.73	−1.97	0.00	0.00	−1.76	−1.66
Ag_3Bi_3GeSn	−0.82	−1.91	−2.03	−1.76	0.00	−1.50
Ag_3Bi_3GePb	−0.81	−1.90	−2.02	0.00	−1.74	−1.49
Ag_3Bi_3SnPb	−0.80	−1.90	0.00	−2.08	−1.75	−1.49
$Ag_2Bi_2GeSn_3$	−0.79	−1.91	−1.96	−1.88	0.00	−1.63
$Ag_2Bi_2GePb_3$	−0.78	−1.92	−1.93	0.00	−1.87	−1.62
$Ag_2Bi_2SnPb_3$	−0.77	−1.91	0.00	−1.86	−1.73	−1.55
$Ag_2Bi_2Ge_2Sn_2$	−0.79	−1.92	−1.92	−1.92	0.00	−1.64
$Ag_2Bi_2Ge_2Pb_2$	−0.78	−1.93	−1.91	0.00	−1.91	−1.63
$Ag_2Bi_2Sn_2Pb_2$	−0.76	−1.92	0.00	−1.88	−1.88	−1.61
$Ag_2Bi_2Ge_3Sn$	−0.79	−1.93	−1.91	−2.00	0.00	−1.65
$Ag_2Bi_2Ge_3Pb$	−0.78	−1.94	−1.91	0.00	−1.97	−1.64
$Ag_2Bi_2Sn_3Pb$	−0.77	−1.92	0.00	−1.86	−1.96	−1.61
$AgBiGeSn_5$	−0.74	−1.96	−1.92	−1.88	0.00	−1.75
$AgBiGePb_5$	−0.72	−1.98	−1.89	0.00	−1.74	−1.66
$AgBiSnPb_5$	−0.73	−1.93	0.00	−1.84	−1.75	−1.66
$AgBiGe_2Sn_4$	−0.75	−1.98	−1.94	−1.89	0.00	−1.77
$AgBiGe_2Pb_4$	−0.72	−1.99	−1.87	0.00	−1.73	−1.67

Continued on next page

Table S-14 – Continued from previous page

Metal composition	Ag–Br	Bi–Br	Ge–Br	Sn–Br	Pb–Br	Total/48
$AgBiSn_2Pb_4$	−0.73	−1.99	0.00	−1.86	−1.84	−1.72
$AgBiGe_3Sn_3$	−0.76	−1.97	−1.95	−1.87	0.00	−1.78
$AgBiGe_3Pb_3$	−0.76	−1.99	−1.95	0.00	−1.85	−1.77
$AgBiSn_3Pb_3$	−0.73	−1.96	0.00	−1.87	−1.82	−1.72
$AgBiGe_4Sn_2$	−0.78	−1.96	−1.97	−1.82	0.00	−1.78
$AgBiGe_4Pb_2$	−0.78	−1.96	−1.97	0.00	−1.80	−1.78
$AgBiSn_4Pb_2$	−0.74	−1.95	0.00	−1.89	−1.79	−1.73
$AgBiGe_5Sn$	−0.75	−1.97	−1.94	−1.92	0.00	−1.79
$AgBiGe_5Pb$	−0.75	−1.97	−1.94	0.00	−1.87	−1.79
$AgBiSn_5Pb$	−0.73	−1.94	0.00	−1.86	−1.86	−1.73

S-8.3 Electronic and Optical Bandgaps

Table S-15. Bandgap at Γ point (E_g^{Γ}), indirect fundamental ($E_g^{f\text{-}i}$), and direct fundamental bandgap ($E_g^{f\text{-}d}$) at PBE+D3 and PBE+D3+SOC levels. Besides, the bandgap at Γ point calculated with the HSE06 functional on optimized structres at PBE+D3 level, and the optical bandgap (E_g^{optical}) obtained from the absorption coefficient calculated at PBE+D3 is also shown (first energy value when $\alpha = (1/3)(\alpha_{xx} + \alpha_{yy} + \alpha_{zz}) > 10^0\text{cm}^{-1}$). All bandgaps are given in eV and were calculated only for the lowest-structure configuration of each compound (identified here by the metal composition where the subscripts indicate the number of atoms within the unit-cell).

Metal	PBE+D3			PBE+D3+SOC			HSE06	PBE+D3
composition	E_g^{Γ}	$E_g^{f\text{-}i}$	$E_g^{f\text{-}d}$	E_g^{Γ}	$E_g^{f\text{-}i}$	$E_g^{f\text{-}d}$	E_g^{Γ}	$E_g^{optical}$
Ag_4Bi_4	2.28	1.28	2.23	1.26	1.07	1.26	3.12	2.22
Ge_8	1.22	1.22	1.22	1.14	1.14	1.14	1.97	1.19
Sn_8	0.54	0.54	0.54	0.19	0.19	0.19	1.14	0.04
Pb_8	1.68	1.68	1.68	0.56	0.56	0.56	2.40	1.64
$Ag_3Bi_3Ge_2$	0.79	0.79	0.79	0.40	0.40	0.40	1.47	0.75
$Ag_3Bi_3Sn_2$	0.67	0.49	0.67	0.16	0.16	0.16	1.25	0.64
$Ag_3Bi_3Pb_2$	1.13	0.84	1.13	0.39	0.39	0.39	1.86	1.11
$Ag_2Bi_2Ge_4$	0.79	0.79	0.79	0.24	0.24	0.24	1.48	0.79
$Ag_2Bi_2Sn_4$	0.53	0.53	0.53	0.07	−0.01	0.01	1.11	0.54

Continued on next page

Table S-15 – Continued from previous page

Metal	PBE+D3			PBE+D3+SOC			HSE06	PBE+D3
composition	E_g^{Γ}	$E_g^{f\text{-}i}$	$E_g^{f\text{-}d}$	E_g^{Γ}	$E_g^{f\text{-}i}$	$E_g^{f\text{-}d}$	E_g^{Γ}	$E_g^{optical}$
$Ag_2Bi_2Pb_4$	1.04	1.04	1.04	0.30	0.30	0.30	1.77	1.00
$AgBiGe_6$	0.77	0.77	0.77	0.26	0.26	0.26	1.45	0.75
$AgBiSn_6$	0.27	0.27	0.27	0.28	0.15	0.17	0.76	0.25
$AgBiPb_6$	1.17	1.17	1.17	0.42	0.42	0.42	1.98	1.14
Ag_3Bi_3GeSn	0.55	0.55	0.55	0.17	0.17	0.17	1.16	0.54
Ag_3Bi_3GePb	0.84	0.82	0.84	0.45	0.45	0.45	1.57	0.82
Ag_3Bi_3SnPb	0.96	0.78	0.96	0.43	0.43	0.43	1.63	0.93
$Ag_2Bi_2GeSn_3$	0.44	0.44	0.44	0.12	0.12	0.12	0.99	0.43
$Ag_2Bi_2GePb_3$	1.02	1.02	1.02	0.59	0.59	0.59	1.70	1.00
$Ag_2Bi_2SnPb_3$	1.19	1.19	1.19	0.68	0.68	0.68	1.83	1.18
$Ag_2Bi_2Ge_2Sn_2$	0.53	0.53	0.53	0.20	0.20	0.20	1.09	0.50
$Ag_2Bi_2Ge_2Pb_2$	0.97	0.97	0.97	0.56	0.56	0.56	1.65	0.93
$Ag_2Bi_2Sn_2Pb_2$	0.75	0.75	0.75	0.25	0.25	0.25	1.32	0.71
$Ag_2Bi_2Ge_3Sn$	0.72	0.72	0.72	0.38	0.38	0.38	1.32	0.68
$Ag_2Bi_2Ge_3Pb$	0.89	0.89	0.89	0.52	0.52	0.52	1.55	0.86
$Ag_2Bi_2Sn_3Pb$	0.59	0.59	0.59	0.12	0.12	0.12	1.15	0.57
$AgBiGeSn_5$	0.43	0.43	0.43	0.10	0.01	0.01	0.95	0.39
$AgBiGePb_5$	1.06	1.06	1.06	0.26	0.26	0.26	1.89	1.04
$AgBiSnPb_5$	1.01	1.01	1.01	0.39	0.39	0.39	1.70	1.00
$AgBiGe_2Sn_4$	0.57	0.57	0.57	0.03	0.03	0.03	1.16	0.54
$AgBiGe_2Pb_4$	1.10	1.10	1.10	0.29	0.29	0.29	1.87	1.07
$AgBiSn_2Pb_4$	0.64	0.64	0.64	0.08	0.07	0.08	1.21	0.61
$AgBiGe_3Sn_3$	0.44	0.44	0.44	0.04	0.02	0.02	1.01	0.43
$AgBiGe_3Pb_3$	0.81	0.81	0.81	0.28	0.27	0.28	1.57	0.79
$AgBiSn_3Pb_3$	0.48	0.48	0.48	0.12	0.10	0.11	1.03	0.46
$AgBiGe_4Sn_2$	0.47	0.47	0.47	0.02	0.00	0.00	1.10	0.43
$AgBiGe_4Pb_2$	0.71	0.71	0.71	0.22	0.22	0.22	1.48	0.68
$AgBiSn_4Pb_2$	0.40	0.40	0.40	0.16	0.14	0.15	0.92	0.39
$AgBiGe_5Sn$	0.52	0.52	0.52	0.02	0.02	0.02	1.08	0.50
$AgBiGe_5Pb$	0.80	0.80	0.80	0.26	0.26	0.26	1.44	0.79
$AgBiSn_5Pb$	0.43	0.43	0.43	0.14	0.12	0.12	0.95	0.39

Table S-16. Energy of the scissor operator χ^{HSE06} defined as $\chi^{\text{HSE06}} = E_g^{\Gamma\text{-HSE06}} - E_g^{\Gamma\text{-PBE+D3}}$. Besides, the corrected bandgap $E_g^{\text{PBE+D3+SOC+}\chi^{\text{HSE06}}} = E_g^{\text{PBE+D3+SOC}} + \chi^{\text{HSE06}}$ is also shown. All energy values are given in eV and were calculated only for the lowest-structure configuration of each compound (identified here by the metal composition where the subscripts indicate the number of atoms within the unit-cell)

Metal composition	HSE06 E_g^{Γ}	PBE+D3 E_g^{Γ}	χ^{HSE06}	PBE+D3+SOC $E_g^{f\text{-}i}$	PBE+D3+SOC+χ^{HSE06} E_g^{f}
Ag_4Bi_4	3.12	2.28	0.84	1.07	1.92
Ge_8	1.97	1.22	0.75	1.14	1.88
Sn_8	1.14	0.54	0.60	0.19	0.79
Pb_8	2.40	1.68	0.72	0.56	1.29
$Ag_3Bi_3Ge_2$	1.47	0.79	0.67	0.40	1.07
$Ag_3Bi_3Sn_2$	1.25	0.67	0.59	0.16	0.75
$Ag_3Bi_3Pb_2$	1.86	1.13	0.73	0.39	1.12
$Ag_2Bi_2Ge_4$	1.48	0.79	0.69	0.24	0.93
$Ag_2Bi_2Sn_4$	1.11	0.53	0.58	−0.01	0.57
$Ag_2Bi_2Pb_4$	1.77	1.04	0.73	0.30	1.04
$AgBiGe_6$	1.45	0.77	0.68	0.26	0.94
$AgBiSn_6$	0.76	0.27	0.50	0.15	0.64
$AgBiPb_6$	1.98	1.17	0.80	0.42	1.23
Ag_3Bi_3GeSn	1.16	0.55	0.61	0.17	0.78
Ag_3Bi_3GePb	1.57	0.84	0.72	0.45	1.17
Ag_3Bi_3SnPb	1.63	0.96	0.67	0.43	1.10
$Ag_2Bi_2GeSn_3$	0.99	0.44	0.54	0.12	0.66
$Ag_2Bi_2GePb_3$	1.70	1.02	0.68	0.59	1.27
$Ag_2Bi_2SnPb_3$	1.83	1.19	0.64	0.68	1.32
$Ag_2Bi_2Ge_2Sn_2$	1.09	0.53	0.56	0.20	0.77
$Ag_2Bi_2Ge_2Pb_2$	1.65	0.97	0.68	0.56	1.23
$Ag_2Bi_2Sn_2Pb_2$	1.32	0.75	0.57	0.25	0.82
$Ag_2Bi_2Ge_3Sn$	1.32	0.72	0.59	0.38	0.98
$Ag_2Bi_2Ge_3Pb$	1.55	0.89	0.66	0.52	1.17
$Ag_2Bi_2Sn_3Pb$	1.15	0.59	0.55	0.12	0.67
$AgBiGeSn_5$	0.95	0.43	0.52	0.01	0.53
$AgBiGePb_5$	1.89	1.06	0.83	0.26	1.09
$AgBiSnPb_5$	1.70	1.01	0.68	0.39	1.07
$AgBiGe_2Sn_4$	1.16	0.57	0.59	0.03	0.62
$AgBiGe_2Pb_4$	1.87	1.10	0.77	0.29	1.06
$AgBiSn_2Pb_4$	1.21	0.64	0.57	0.07	0.64

Continued on next page

Table S-16 – Continued from previous page

Metal composition	HSE06 E_g^{Γ}	PBE+D3 E_g^{Γ}	χ^{HSE06}	PBE+D3+SOC $E_g^{f\text{-}i}$	PBE+D3+SOC+χ^{HSE06} E_g^{f}
$AgBiGe_3Sn_3$	1.01	0.44	0.57	0.02	0.59
$AgBiGe_3Pb_3$	1.57	0.81	0.76	0.27	1.03
$AgBiSn_3Pb_3$	1.03	0.48	0.55	0.10	0.65
$AgBiGe_4Sn_2$	1.10	0.47	0.63	0.00	0.64
$AgBiGe_4Pb_2$	1.48	0.71	0.77	0.22	0.99
$AgBiSn_4Pb_2$	0.92	0.40	0.52	0.14	0.67
$AgBiGe_5Sn$	1.08	0.52	0.57	0.02	0.58
$AgBiGe_5Pb$	1.44	0.80	0.64	0.26	0.91
$AgBiSn_5Pb$	0.95	0.43	0.52	0.12	0.64

Table S-17. Energy of the scissor operator χ defined as $\chi = E_g^{\text{PBE+D3+SOC+}\chi^{\text{HSE06}}} - E_g^{\text{PBE+D3}}$, where only the indirect fundamental bandgaps were considered. Besides, the corrected bandgap $E_g^{optical+\chi} = E_g^{optical} + \chi$ is also shown. All energy values are given in eV and were calculated only for the lowest-structure configuration of each compound (identified here by the metal composition where the subscripts indicate the number of atoms within the unit-cell)

Metal composition	PBE+D3+SOC+HSE06 E_g^{f}	PBE+D3 $E_g^{f\text{-}i}$	χ	PBE+D3 $E_g^{optical}$	PBE+D3+χ $E_g^{optical}$
Ag_4Bi_4	1.92	1.28	0.63	2.22	2.85
Ge_8	1.88	1.22	0.66	1.19	1.85
Sn_8	0.79	0.54	0.26	0.04	0.29
Pb_8	1.29	1.68	−0.39	1.64	1.25
$Ag_3Bi_3Ge_2$	1.07	0.79	0.28	0.75	1.03
$Ag_3Bi_3Sn_2$	0.75	0.49	0.25	0.64	0.90
$Ag_3Bi_3Pb_2$	1.12	0.84	0.28	1.11	1.39
$Ag_2Bi_2Ge_4$	0.93	0.79	0.13	0.79	0.92
$Ag_2Bi_2Sn_4$	0.57	0.53	0.05	0.54	0.58
$Ag_2Bi_2Pb_4$	1.04	1.04	0.00	1.00	1.00
$AgBiGe_6$	0.94	0.77	0.17	0.75	0.92
$AgBiSn_6$	0.64	0.27	0.38	0.25	0.63
$AgBiPb_6$	1.23	1.17	0.05	1.14	1.20

Continued on next page

Table S-17 – Continued from previous page

Metal composition	PBE+D3+SOC+HSE06 E_g^f	PBE+D3 $E_g^{f\text{-}i}$	χ	PBE+D3 $E_g^{optical}$	PBE+D3+χ $E_g^{optical}$
Ag_3Bi_3GeSn	0.78	0.55	0.23	0.54	0.77
Ag_3Bi_3GePb	1.17	0.82	0.35	0.82	1.17
Ag_3Bi_3SnPb	1.10	0.78	0.32	0.93	1.25
$Ag_2Bi_2GeSn_3$	0.66	0.44	0.22	0.43	0.65
$Ag_2Bi_2GePb_3$	1.27	1.02	0.25	1.00	1.25
$Ag_2Bi_2SnPb_3$	1.32	1.19	0.13	1.18	1.31
$Ag_2Bi_2Ge_2Sn_2$	0.77	0.53	0.24	0.50	0.74
$Ag_2Bi_2Ge_2Pb_2$	1.23	0.97	0.26	0.93	1.19
$Ag_2Bi_2Sn_2Pb_2$	0.82	0.75	0.07	0.71	0.79
$Ag_2Bi_2Ge_3Sn$	0.98	0.72	0.26	0.68	0.94
$Ag_2Bi_2Ge_3Pb$	1.17	0.89	0.28	0.86	1.15
$Ag_2Bi_2Sn_3Pb$	0.67	0.59	0.07	0.57	0.64
$AgBiGeSn_5$	0.53	0.43	0.10	0.39	0.49
$AgBiGePb_5$	1.09	1.06	0.03	1.04	1.06
$AgBiSnPb_5$	1.07	1.01	0.06	1.00	1.05
$AgBiGe_2Sn_4$	0.62	0.57	0.05	0.54	0.59
$AgBiGe_2Pb_4$	1.06	1.10	−0.04	1.07	1.03
$AgBiSn_2Pb_4$	0.64	0.64	0.00	0.61	0.61
$AgBiGe_3Sn_3$	0.59	0.44	0.15	0.43	0.58
$AgBiGe_3Pb_3$	1.03	0.81	0.22	0.79	1.01
$AgBiSn_3Pb_3$	0.65	0.48	0.17	0.46	0.64
$AgBiGe_4Sn_2$	0.64	0.47	0.17	0.43	0.60
$AgBiGe_4Pb_2$	0.99	0.71	0.28	0.68	0.96
$AgBiSn_4Pb_2$	0.67	0.40	0.27	0.39	0.66
$AgBiGe_5Sn$	0.58	0.52	0.07	0.50	0.57
$AgBiGe_5Pb$	0.91	0.80	0.11	0.79	0.90
$AgBiSn_5Pb$	0.64	0.43	0.20	0.39	0.59

S-8.4 Bowing Parameter of Fundamental and Optical Band Gap

The bowing parameter for binary alloys can be calculated through Equation 3. Since, ternary alloys were studied in this work, the bowing parameter was calculated for

fixed concentrations of Ag_xBi_x, at constant x, and varying only the concentration of the divalent metals (Equation 4). Results from this analysis are shown in Table S-18.

$$E_g(A_xB_{1-x}) = (1-x)E_g(A) + xE_g(B) - bx(1-x); \quad (3)$$

$$E_g(\mathrm{Ag}_x\mathrm{Bi}_xB_yB'_z) = \frac{y}{2}E_g(\mathrm{Ag}_x\mathrm{Bi}_xB_y) + \frac{z}{2}E_g(\mathrm{Ag}_x\mathrm{Bi}_xB'_z) - \frac{1}{2}byz. \quad (4)$$

Table S-18. Bowing parameter (b) obtained for the fundamental ($E_g^{\mathrm{PBE+D3+SOC+}\chi^{\mathrm{HSE06}}}$) and the optical ($E_g^{optical+\chi}$) band gap for mixtures at fixed AgBi concentration through Equation 4. The number of structures that constitute the particular $\mathrm{Ag}_x\mathrm{Bi}_xB_yB'_z$ mixture within our 40-atoms unit-cell models is specified by $N_{\mathrm{Str.}}$ taking into account the constriction $2x + y + z = 2$.

Mixture	B	B'	$N_{\mathrm{Str.}}$	$E_g^{\mathrm{PBE+D3+SOC+}\chi^{\mathrm{HSE06}}}$ b	$E_g^{optical+\chi}$ b
$\mathrm{Ag}_{0.75}\mathrm{Bi}_{0.75}B_yB'_z$	Ge	Sn	3	0.520	0.780
	Ge	Pb	3	−0.300	0.160
	Sn	Pb	3	−0.660	−0.420
$\mathrm{Ag}_{0.50}\mathrm{Bi}_{0.50}B_yB'_z$	Ge	Sn	5	−0.235	−0.108
	Ge	Pb	5	−1.125	−1.111
	Sn	Pb	5	−0.565	−0.522
$\mathrm{Ag}_{0.25}\mathrm{Bi}_{0.25}B_yB'_z$	Ge	Sn	7	0.933	0.985
	Sn	Pb	7	0.320	0.318
	Sn	Pb	7	1.090	1.099

S-8.5 Band structures at PBE+D3, PBE+D3+SOC, and PBE+D3+SOC+χ^{HSE06} levels

S-8.5.1 Pristine Compounds

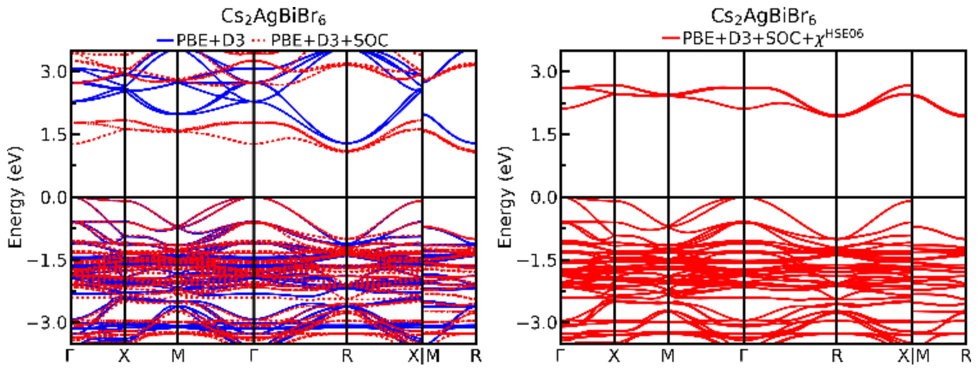

Figure S-66. Band structures of $Cs_2AgBiBr_6$ at PBE+D3, PBE+D3+SOC, and PBE+D3+SOC+χ^{HSE06} levels.

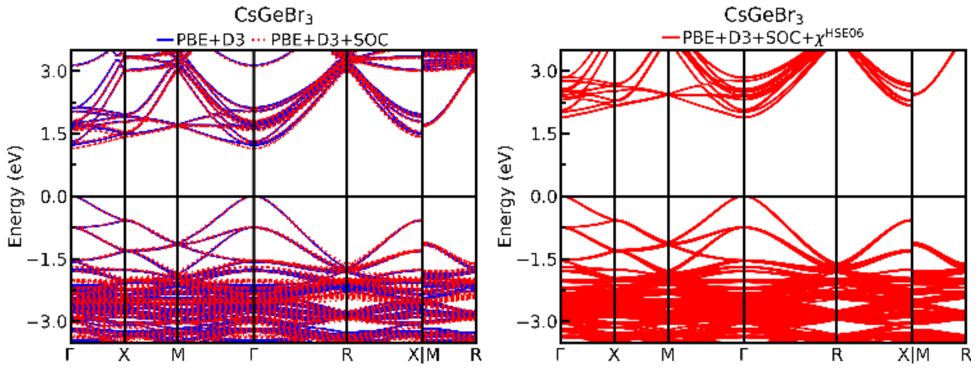

Figure S-67. Band structures of $CsGeBr_3$ at PBE+D3, PBE+D3+SOC, and PBE+D3+SOC+χ^{HSE06} levels.

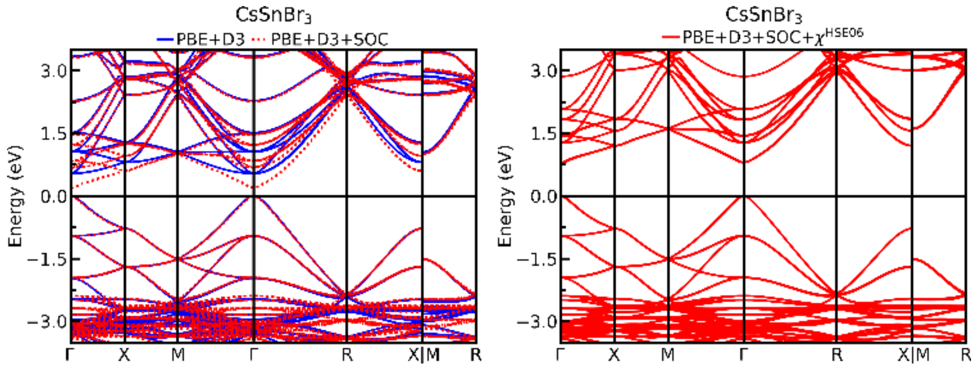

Figure S-68. Band structures of $CsSnBr_3$ at PBE+D3, PBE+D3+SOC, and PBE+D3+SOC+χ^{HSE06} levels.

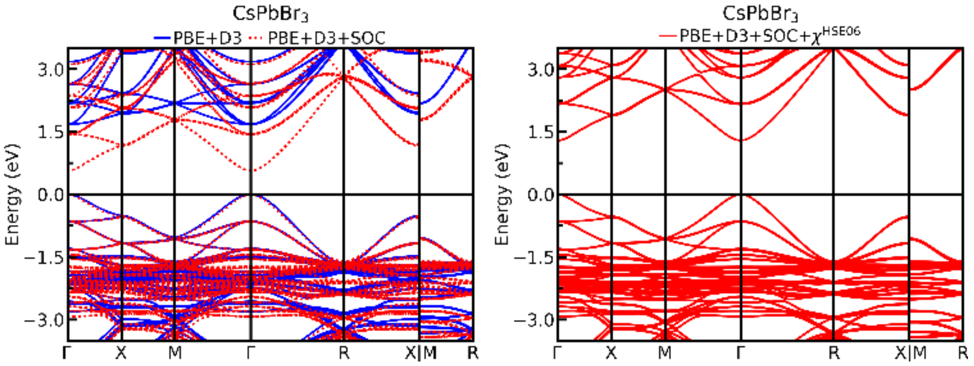

Figure S-69. Band structures of $CsPbBr_3$ at PBE+D3, PBE+D3+SOC, and PBE+D3+SOC+χ^{HSE06} levels.

S-8.5.2 Mixtures with One Divalent Metal

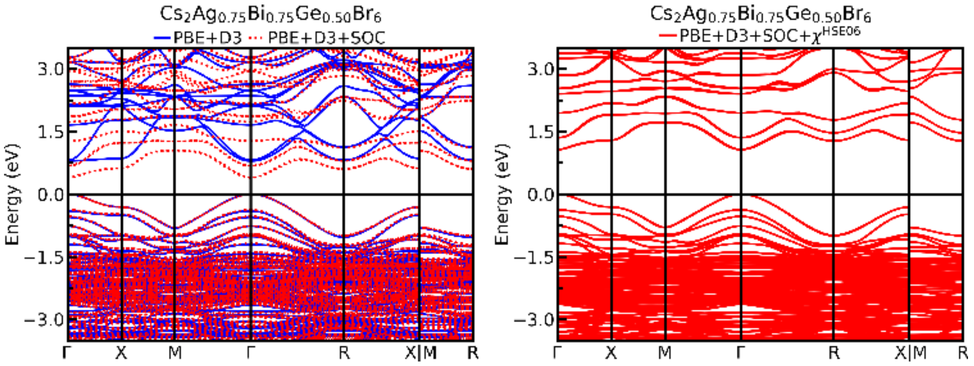

Figure S-70. Band structures of $Cs_2Ag_{0.75}Bi_{0.75}Ge_{0.50}Br_6$ at PBE+D3, PBE+D3+SOC, and PBE+D3+SOC+χ^{HSE06} levels.

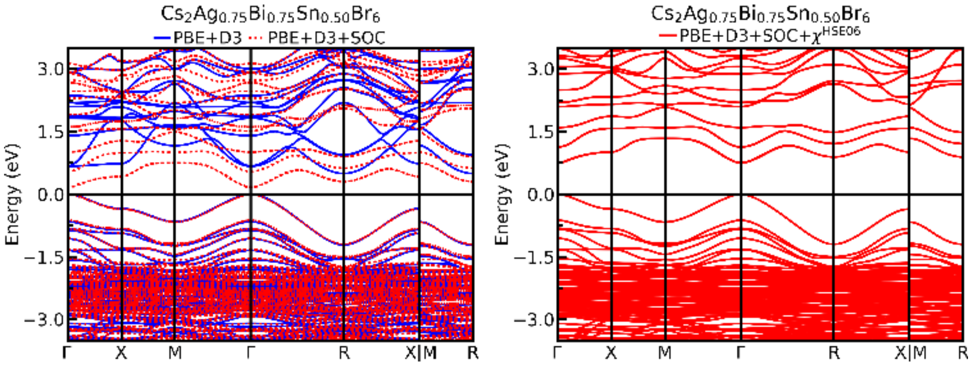

Figure S-71. Band structures of $Cs_2Ag_{0.75}Bi_{0.75}Sn_{0.50}Br_6$ at PBE+D3, PBE+D3+SOC, and PBE+D3+SOC+χ^{HSE06} levels.

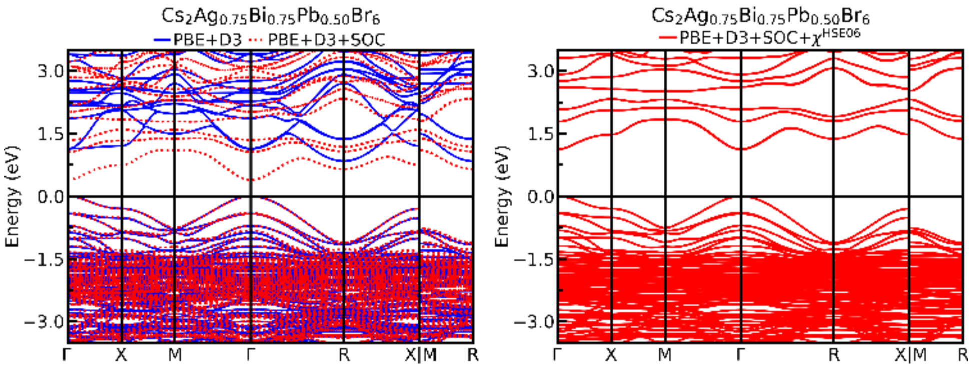

Figure S-72. Band structures of $Cs_2Ag_{0.75}Bi_{0.75}Pb_{0.50}Br_6$ at PBE+D3, PBE+D3+SOC, and PBE+D3+SOC+χ^{HSE06} levels.

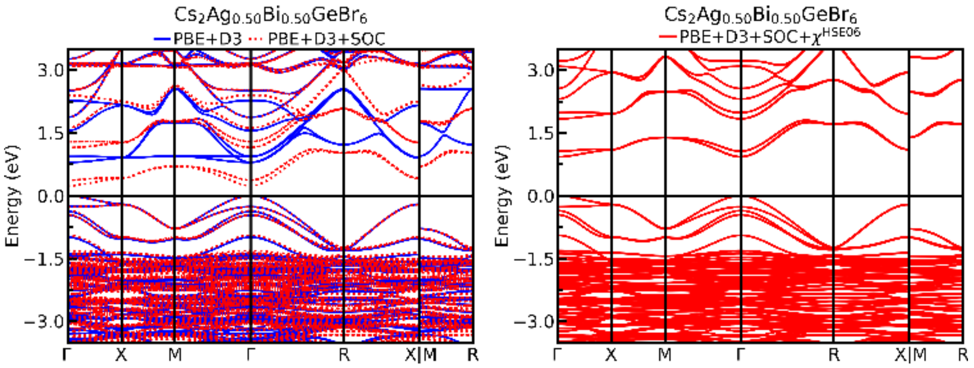

Figure S-73. Band structures of $Cs_2Ag_{0.50}Bi_{0.50}GeBr_6$ at PBE+D3, PBE+D3+SOC, and PBE+D3+SOC+χ^{HSE06} levels.

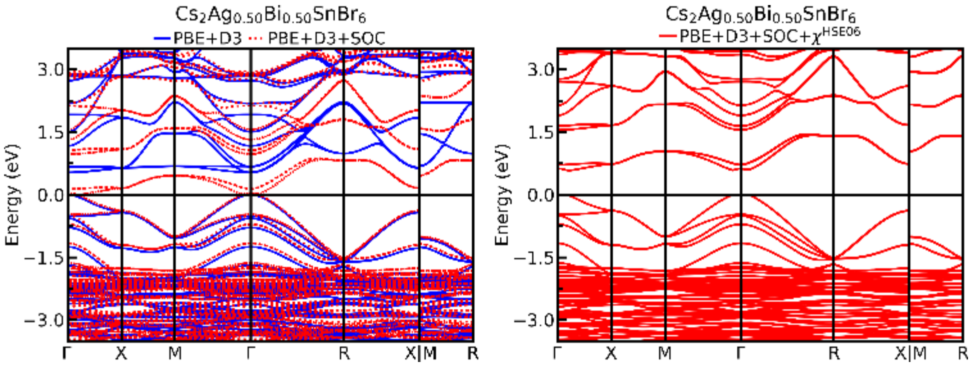

Figure S-74. Band structures of $Cs_2Ag_{0.50}Bi_{0.50}SnBr_6$ at PBE+D3, PBE+D3+SOC, and PBE+D3+SOC+χ^{HSE06} levels.

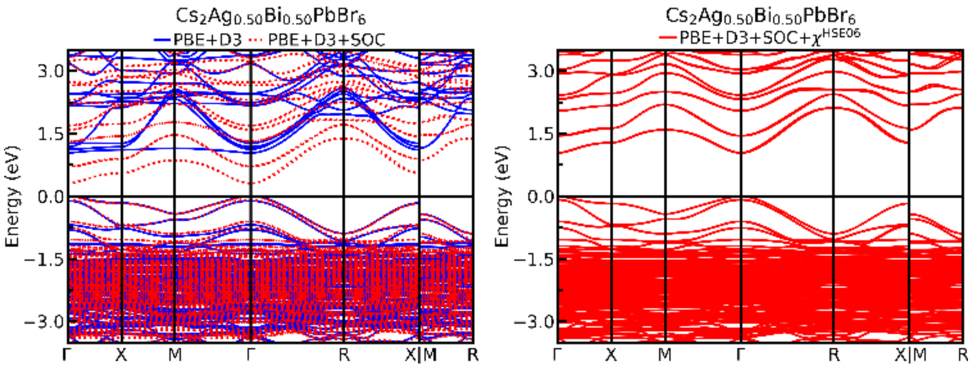

Figure S-75. Band structures of $Cs_2Ag_{0.50}Bi_{0.50}PbBr_6$ at PBE+D3, PBE+D3+SOC, and PBE+D3+SOC+χ^{HSE06} levels.

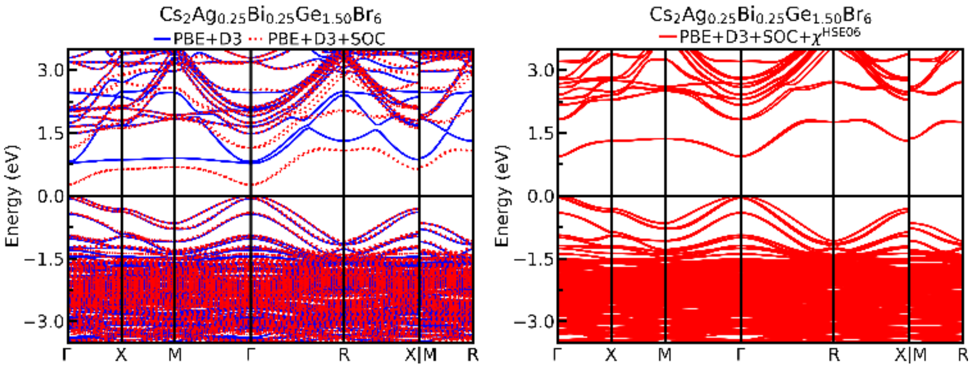

Figure S-76. Band structures of $Cs_2Ag_{0.25}Bi_{0.25}Ge_{1.50}Br_6$ at PBE+D3, PBE+D3+SOC, and PBE+D3+SOC+χ^{HSE06} levels.

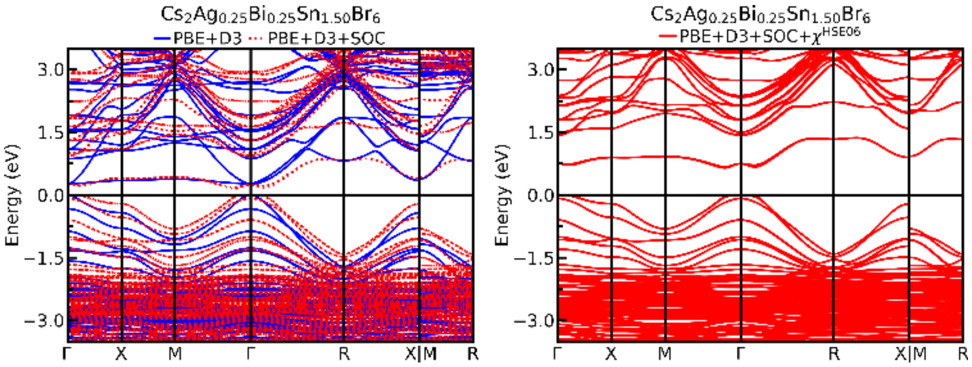

Figure S-77. Band structures of $Cs_2Ag_{0.25}Bi_{0.25}Sn_{1.50}Br_6$ at PBE+D3, PBE+D3+SOC, and PBE+D3+SOC+χ^{HSE06} levels.

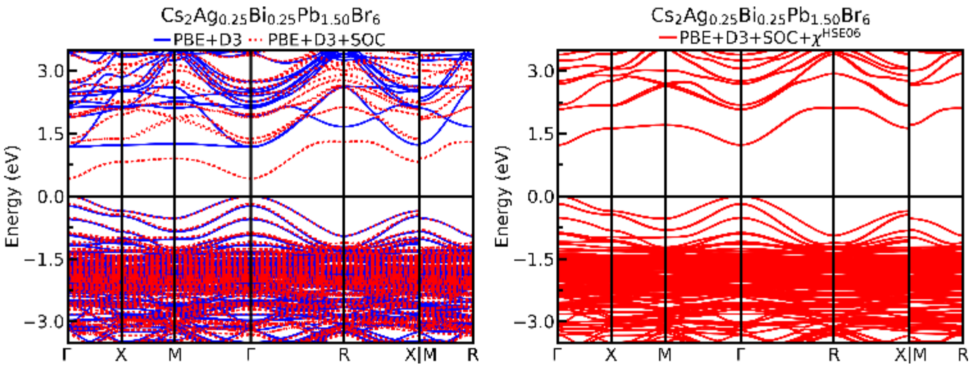

Figure S-78. Band structures of $Cs_2Ag_{0.25}Bi_{0.25}Pb_{1.50}Br_6$ at PBE+D3, PBE+D3+SOC, and PBE+D3+SOC+χ^{HSE06} levels.

S-8.5.3 Mixtures with Two Divalent Metals

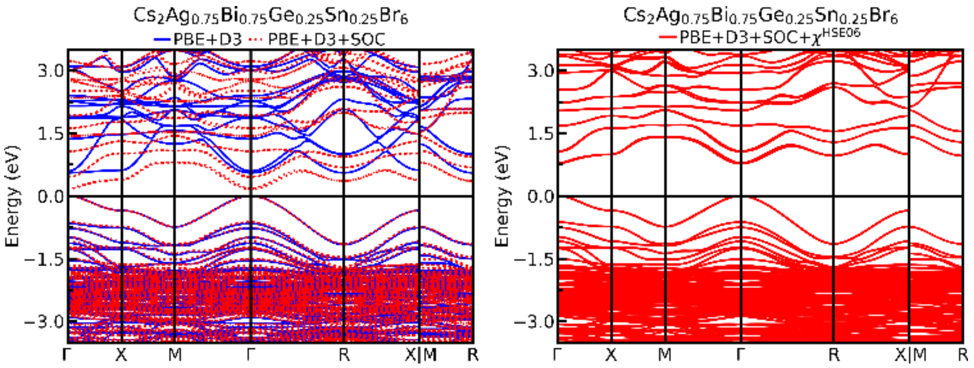

Figure S-79. Band structures of $Cs_2Ag_{0.75}Bi_{0.75}Ge_{0.25}Sn_{0.25}Br_6$ at PBE+D3, PBE+D3+SOC, and PBE+D3+SOC+χ^{HSE06} levels.

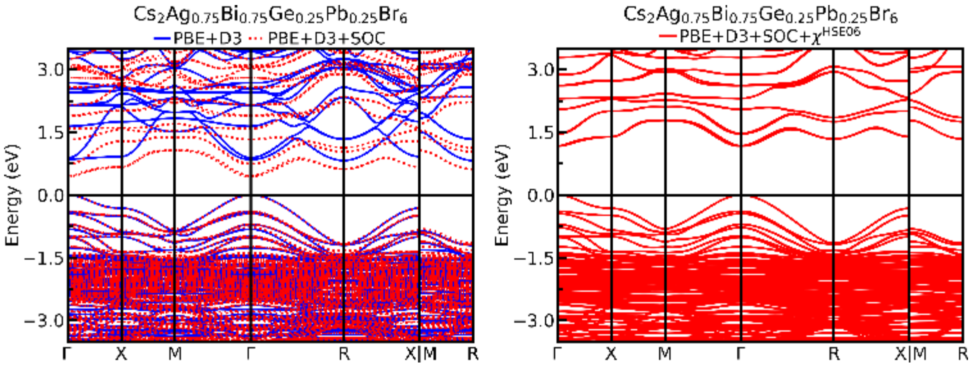

Figure S-80. Band structures of $Cs_2Ag_{0.75}Bi_{0.75}Ge_{0.25}Pb_{0.25}Br_6$ at PBE+D3, PBE+D3+SOC, and PBE+D3+SOC+χ^{HSE06} levels.

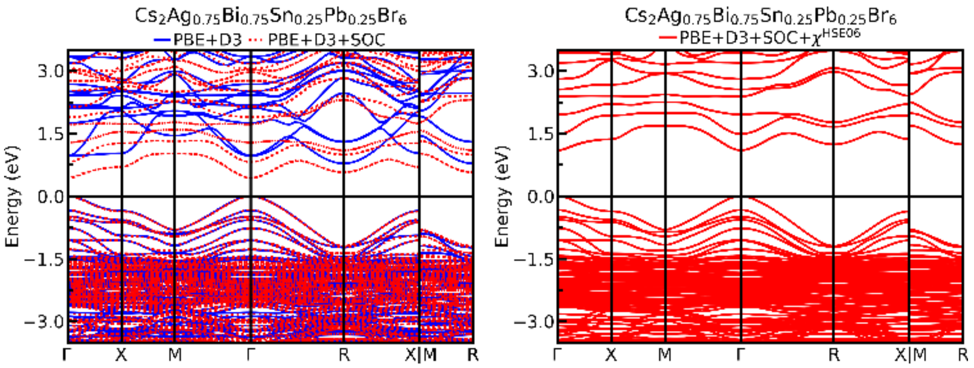

Figure S-81. Band structures of $Cs_2Ag_{0.75}Bi_{0.75}Sn_{0.25}Pb_{0.25}Br_6$ at PBE+D3, PBE+D3+SOC, and PBE+D3+SOC+χ^{HSE06} levels.

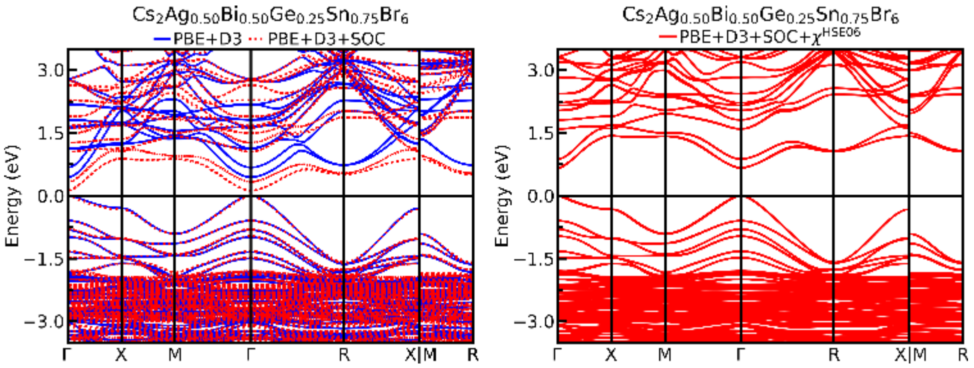

Figure S-82. Band structures of $Cs_2Ag_{0.50}Bi_{0.50}Ge_{0.25}Sn_{0.75}Br_6$ at PBE+D3, PBE+D3+SOC, and PBE+D3+SOC+χ^{HSE06} levels.

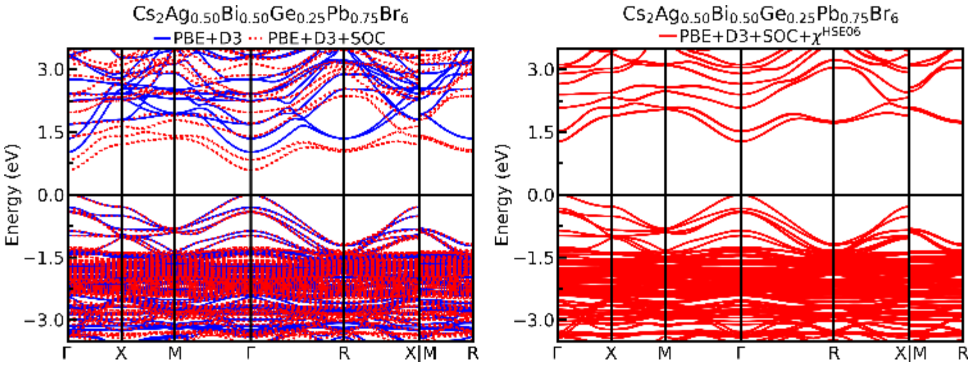

Figure S-83. Band structures of $Cs_2Ag_{0.50}Bi_{0.50}Ge_{0.25}Pb_{0.75}Br_6$ at PBE+D3, PBE+D3+SOC, and PBE+D3+SOC+χ^{HSE06} levels.

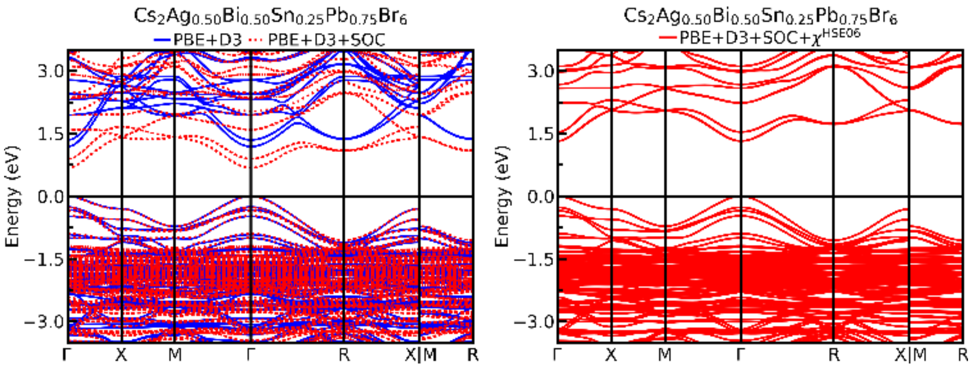

Figure S-84. Band structures of $Cs_2Ag_{0.50}Bi_{0.50}Sn_{0.25}Pb_{0.75}Br_6$ at PBE+D3, PBE+D3+SOC, and PBE+D3+SOC+χ^{HSE06} levels.

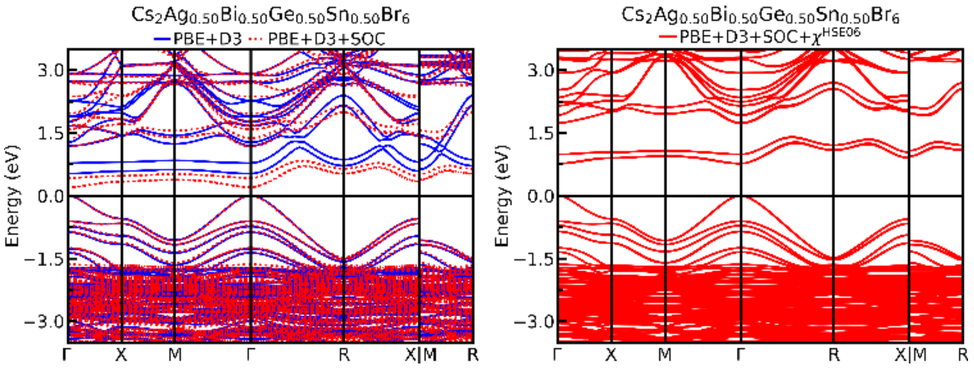

Figure S-85. Band structures of $Cs_2Ag_{0.50}Bi_{0.50}Ge_{0.50}Sn_{0.50}Br_6$ at PBE+D3, PBE+D3+SOC, and PBE+D3+SOC+χ^{HSE06} levels.

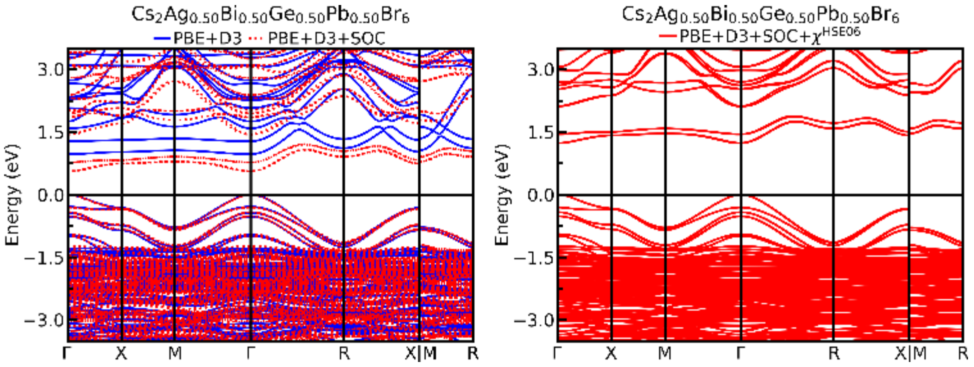

Figure S-86. Band structures of $Cs_2Ag_{0.50}Bi_{0.50}Ge_{0.50}Pb_{0.50}Br_6$ at PBE+D3, PBE+D3+SOC, and PBE+D3+SOC+χ^{HSE06} levels.

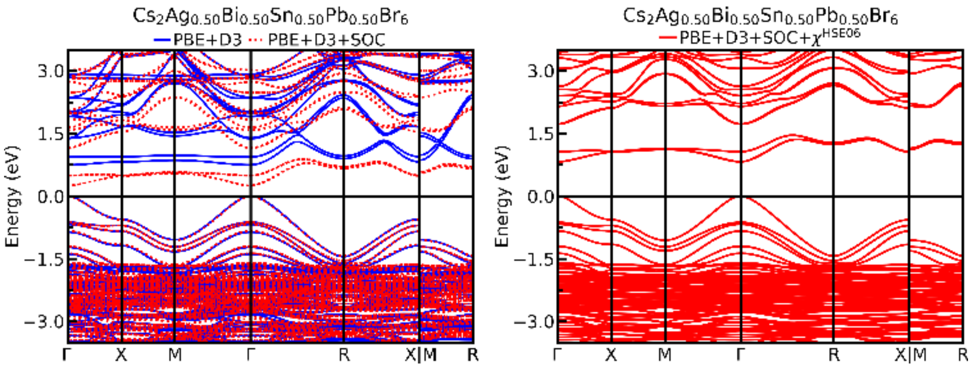

Figure S-87. Band structures of $Cs_2Ag_{0.50}Bi_{0.50}Sn_{0.50}Pb_{0.50}Br_6$ at PBE+D3, PBE+D3+SOC, and PBE+D3+SOC+χ^{HSE06} levels.

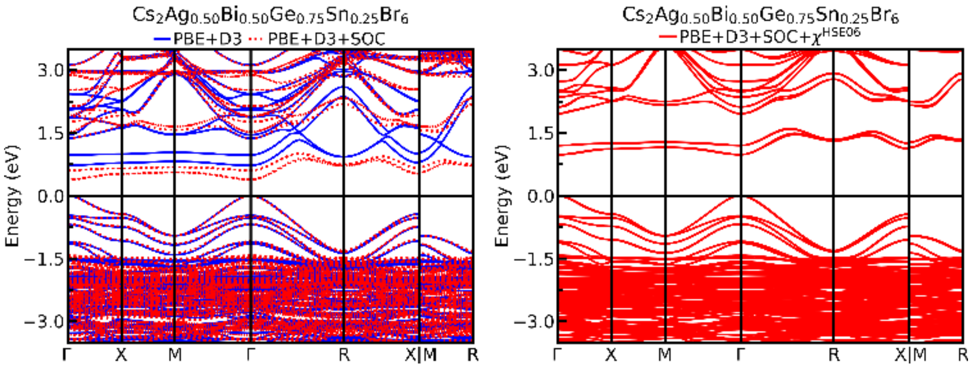

Figure S-88. Band structures of $Cs_2Ag_{0.50}Bi_{0.50}Ge_{0.75}Sn_{0.25}Br_6$ at PBE+D3, PBE+D3+SOC, and PBE+D3+SOC+χ^{HSE06} levels.

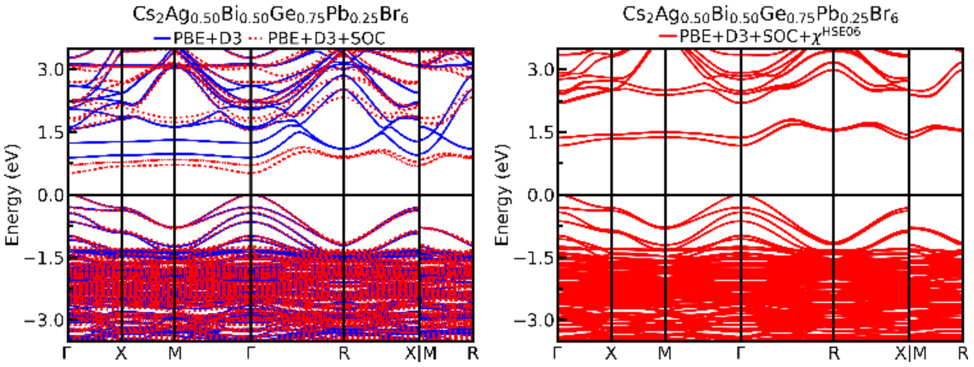

Figure S-89. Band structures of $Cs_2Ag_{0.50}Bi_{0.50}Ge_{0.75}Pb_{0.25}Br_6$ at PBE+D3, PBE+D3+SOC, and PBE+D3+SOC+χ^{HSE06} levels.

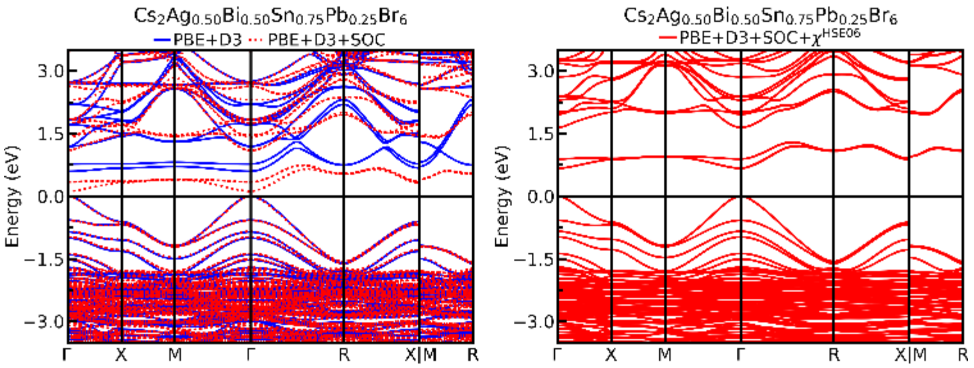

Figure S-90. Band structures of $Cs_2Ag_{0.50}Bi_{0.50}Sn_{0.75}Pb_{0.25}Br_6$ at PBE+D3, PBE+D3+SOC, and PBE+D3+SOC+χ^{HSE06} levels.

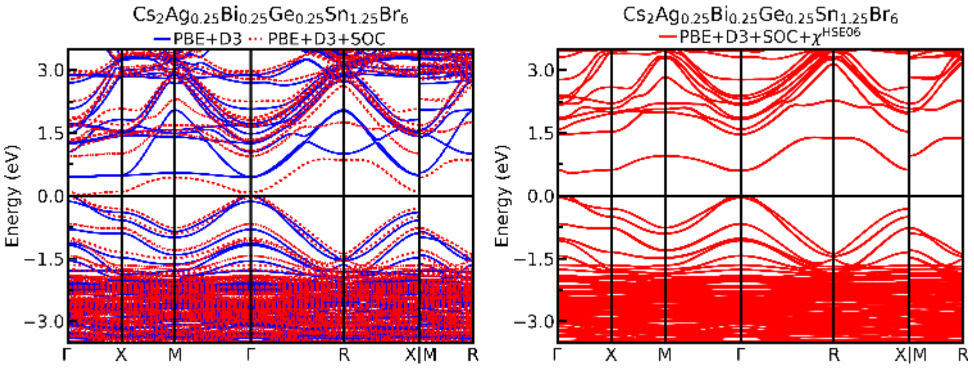

Figure S-91. Band structures of $Cs_2Ag_{0.25}Bi_{0.25}Ge_{0.25}Sn_{1.25}Br_6$ at PBE+D3, PBE+D3+SOC, and PBE+D3+SOC+χ^{HSE06} levels.

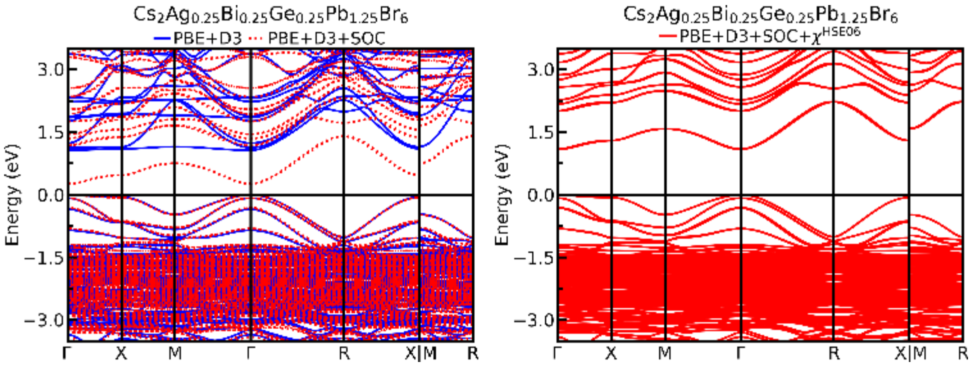

Figure S-92. Band structures of $Cs_2Ag_{0.25}Bi_{0.25}Ge_{0.25}Pb_{1.25}Br_6$ at PBE+D3, PBE+D3+SOC, and PBE+D3+SOC+χ^{HSE06} levels.

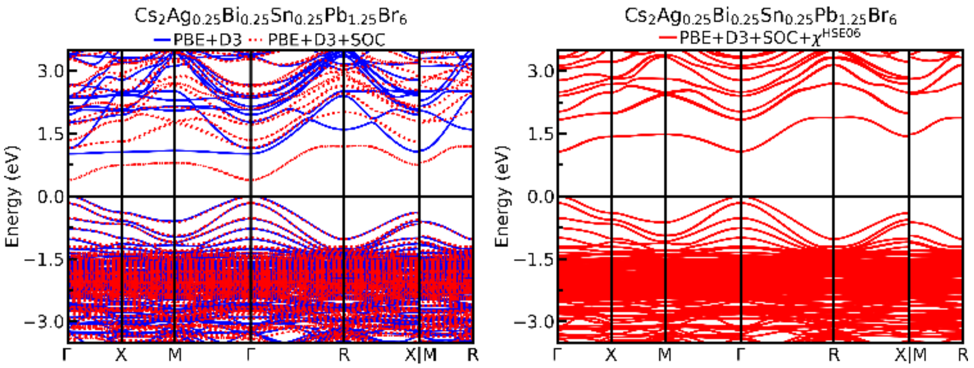

Figure S-93. Band structures of $Cs_2Ag_{0.25}Bi_{0.25}Sn_{0.25}Pb_{1.25}Br_6$ at PBE+D3, PBE+D3+SOC, and PBE+D3+SOC+χ^{HSE06} levels.

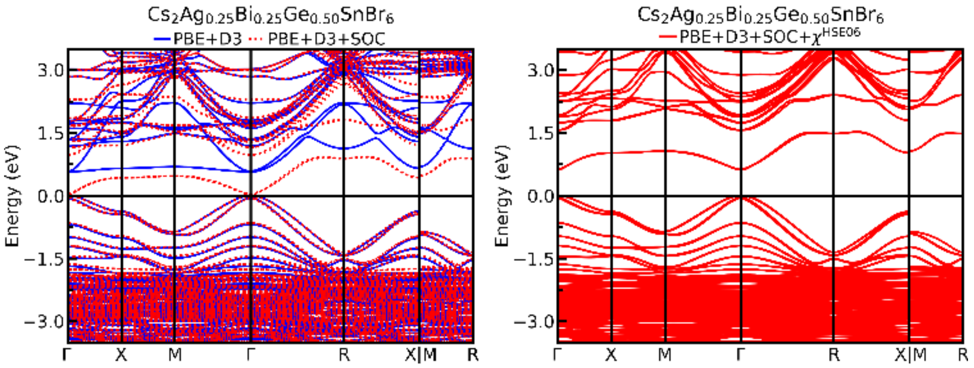

Figure S-94. Band structures of $Cs_2Ag_{0.25}Bi_{0.25}Ge_{0.50}SnBr_6$ at PBE+D3, PBE+D3+SOC, and PBE+D3+SOC+χ^{HSE06} levels.

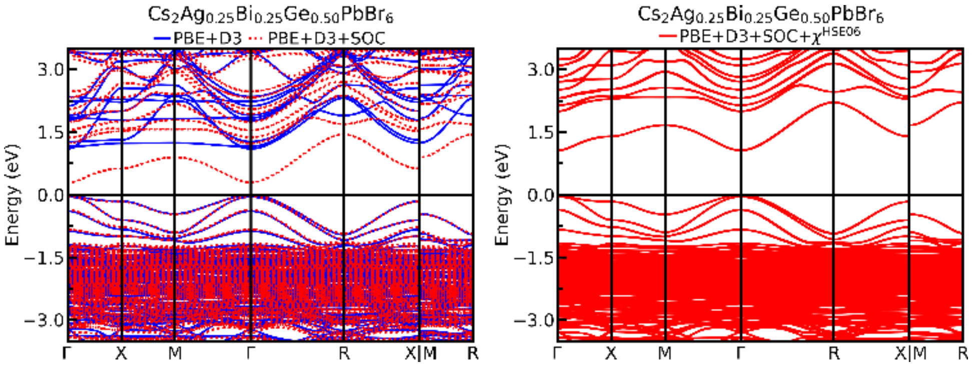

Figure S-95. Band structures of $Cs_2Ag_{0.25}Bi_{0.25}Ge_{0.50}PbBr_6$ at PBE+D3, PBE+D3+SOC, and PBE+D3+SOC+χ^{HSE06} levels.

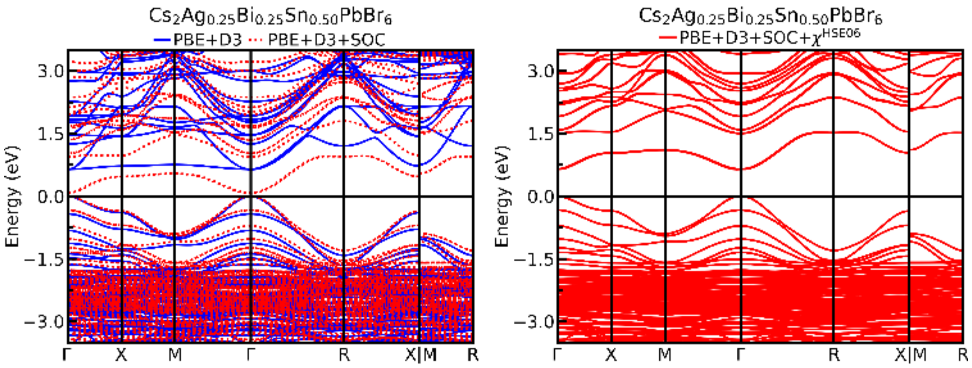

Figure S-96. Band structures of $Cs_2Ag_{0.25}Bi_{0.25}Sn_{0.50}PbBr_6$ at PBE+D3, PBE+D3+SOC, and PBE+D3+SOC+χ^{HSE06} levels.

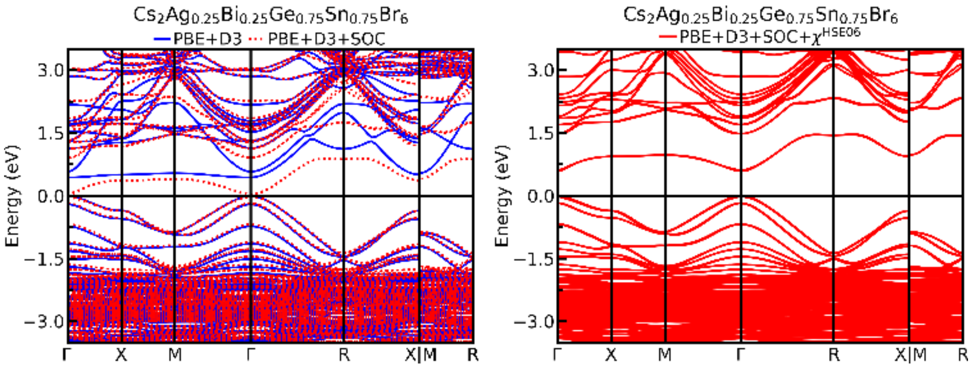

Figure S-97. Band structures of $Cs_2Ag_{0.25}Bi_{0.25}Ge_{0.75}Sn_{0.75}Br_6$ at PBE+D3, PBE+D3+SOC, and PBE+D3+SOC+χ^{HSE06} levels.

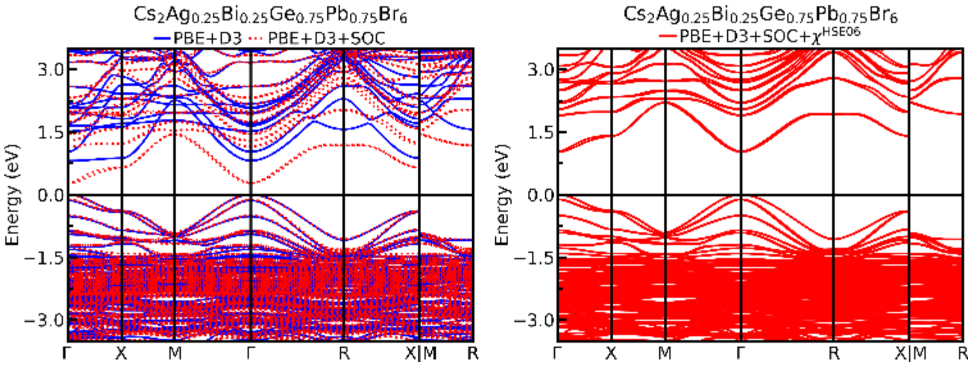

Figure S-98. Band structures of $Cs_2Ag_{0.25}Bi_{0.25}Ge_{0.75}Pb_{0.75}Br_6$ at PBE+D3, PBE+D3+SOC, and PBE+D3+SOC+χ^{HSE06} levels.

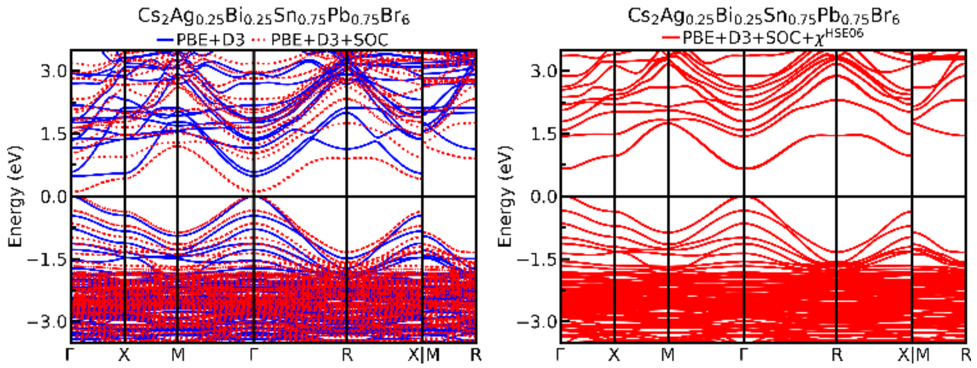

Figure S-99. Band structures of $Cs_2Ag_{0.25}Bi_{0.25}Sn_{0.75}Pb_{0.75}Br_6$ at PBE+D3, PBE+D3+SOC, and PBE+D3+SOC+χ^{HSE06} levels.

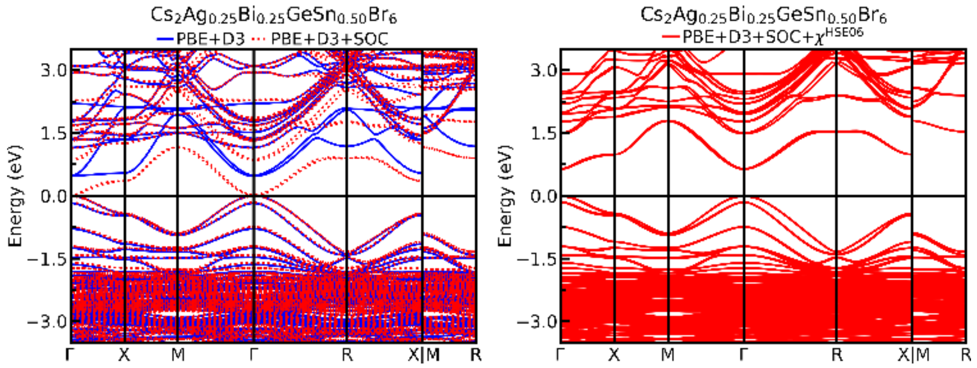

Figure S-100. Band structures of $Cs_2Ag_{0.25}Bi_{0.25}GeSn_{0.50}Br_6$ at PBE+D3, PBE+D3+SOC, and PBE+D3+SOC+χ^{HSE06} levels.

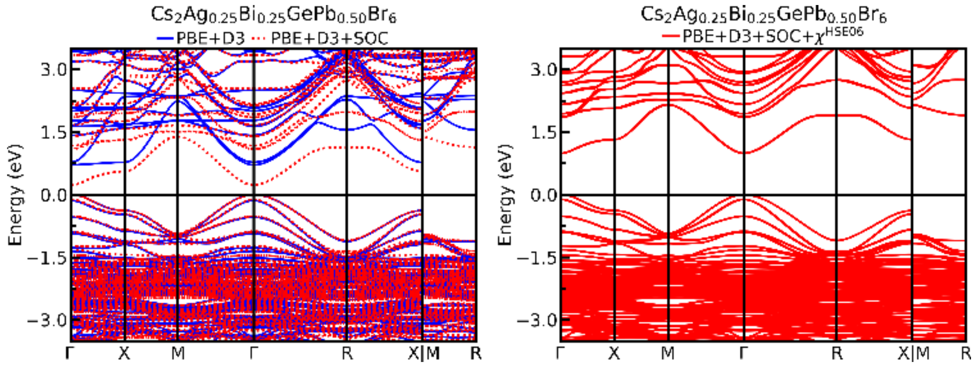

Figure S-101. Band structures of $Cs_2Ag_{0.25}Bi_{0.25}GePb_{0.50}Br_6$ at PBE+D3, PBE+D3+SOC, and PBE+D3+SOC+χ^{HSE06} levels.

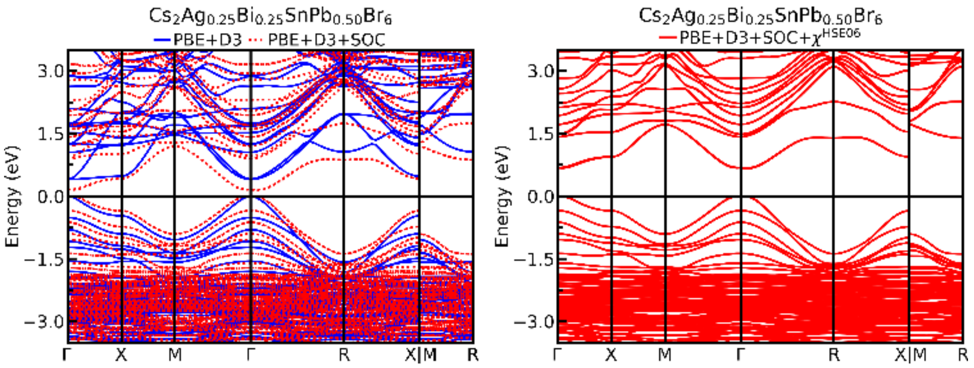

Figure S-102. Band structures of $Cs_2Ag_{0.25}Bi_{0.25}SnPb_{0.50}Br_6$ at PBE+D3, PBE+D3+SOC, and PBE+D3+SOC+χ^{HSE06} levels.

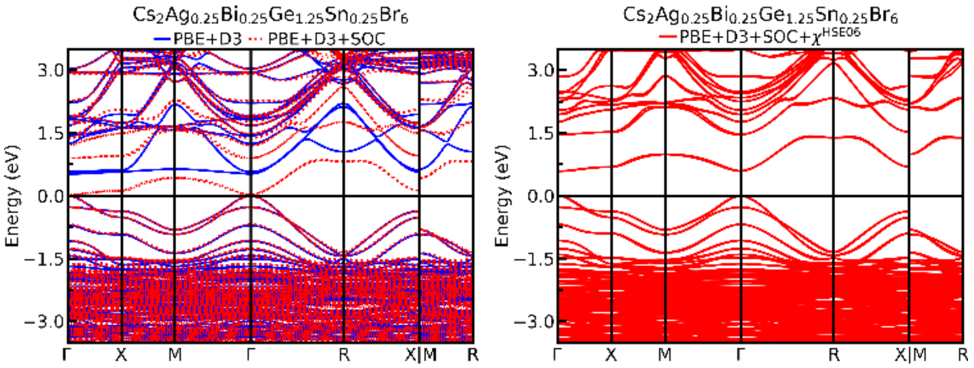

Figure S-103. Band structures of $Cs_2Ag_{0.25}Bi_{0.25}Ge_{1.25}Sn_{0.25}Br_6$ at PBE+D3, PBE+D3+SOC, and PBE+D3+SOC+χ^{HSE06} levels.

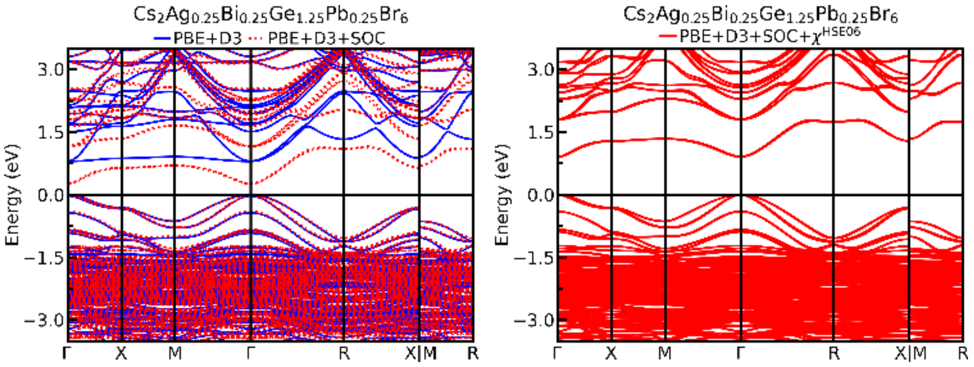

Figure S-104. Band structures of $Cs_2Ag_{0.25}Bi_{0.25}Ge_{1.25}Pb_{0.25}Br_6$ at PBE+D3, PBE+D3+SOC, and PBE+D3+SOC+χ^{HSE06} levels.

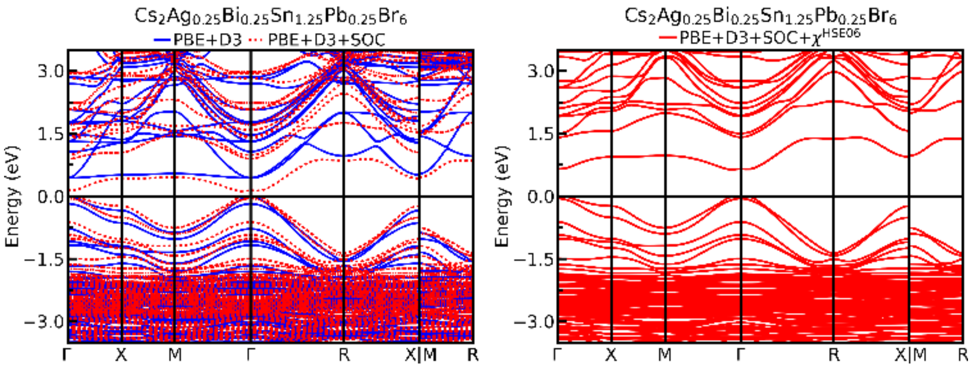

Figure S-105. Band structures of $Cs_2Ag_{0.25}Bi_{0.25}Sn_{1.25}Pb_{0.25}Br_6$ at PBE+D3, PBE+D3+SOC, and PBE+D3+SOC+χ^{HSE06} levels.

S-8.6 Density of States at PBE+D3 level

S-8.6.1 Pristine Compounds

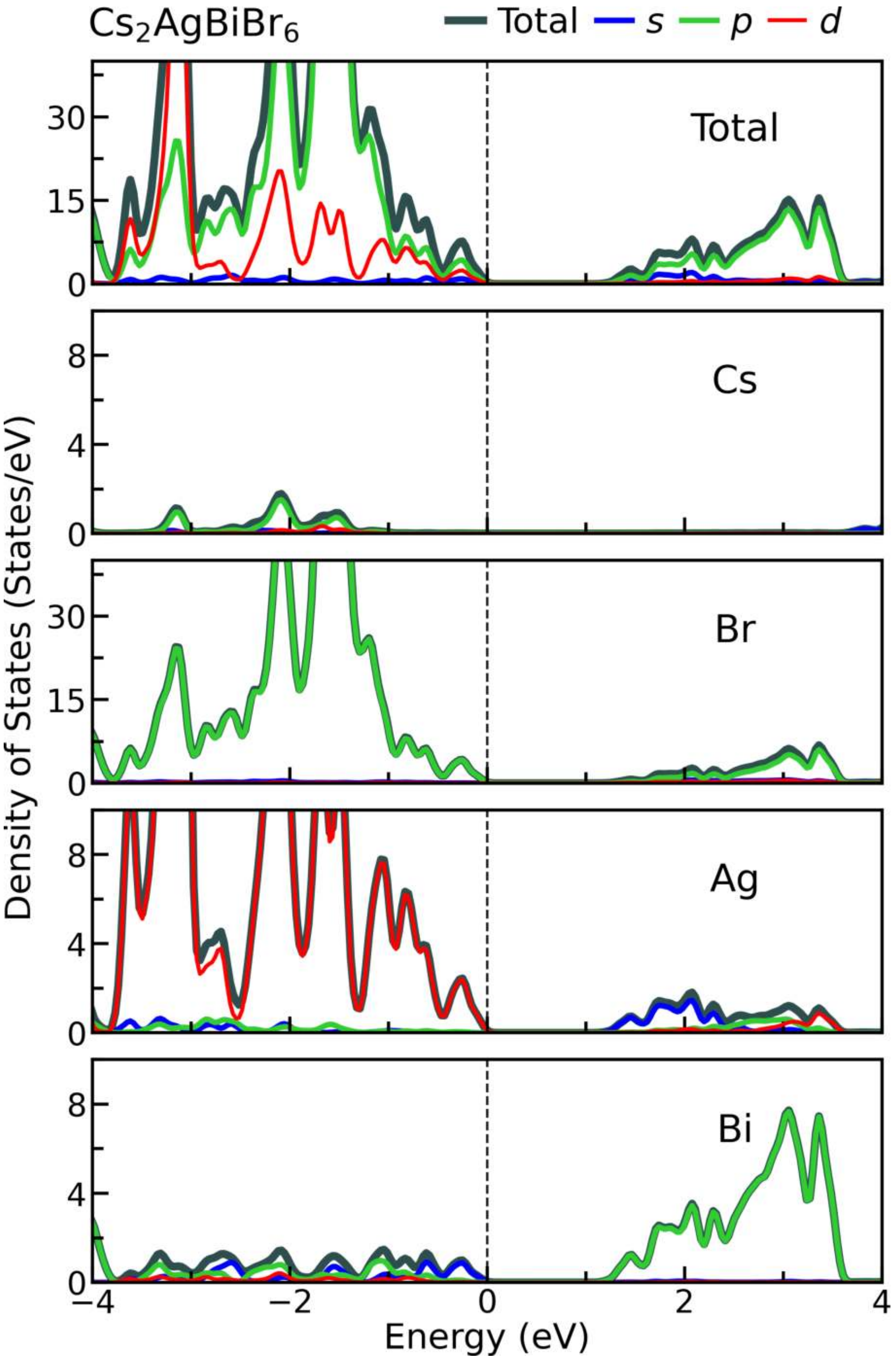

Figure S-106. Density of states of $Cs_2AgBiBr_6$ at PBE+D3 level, for each atomic species and projected only on *s* and *p* orbitals.

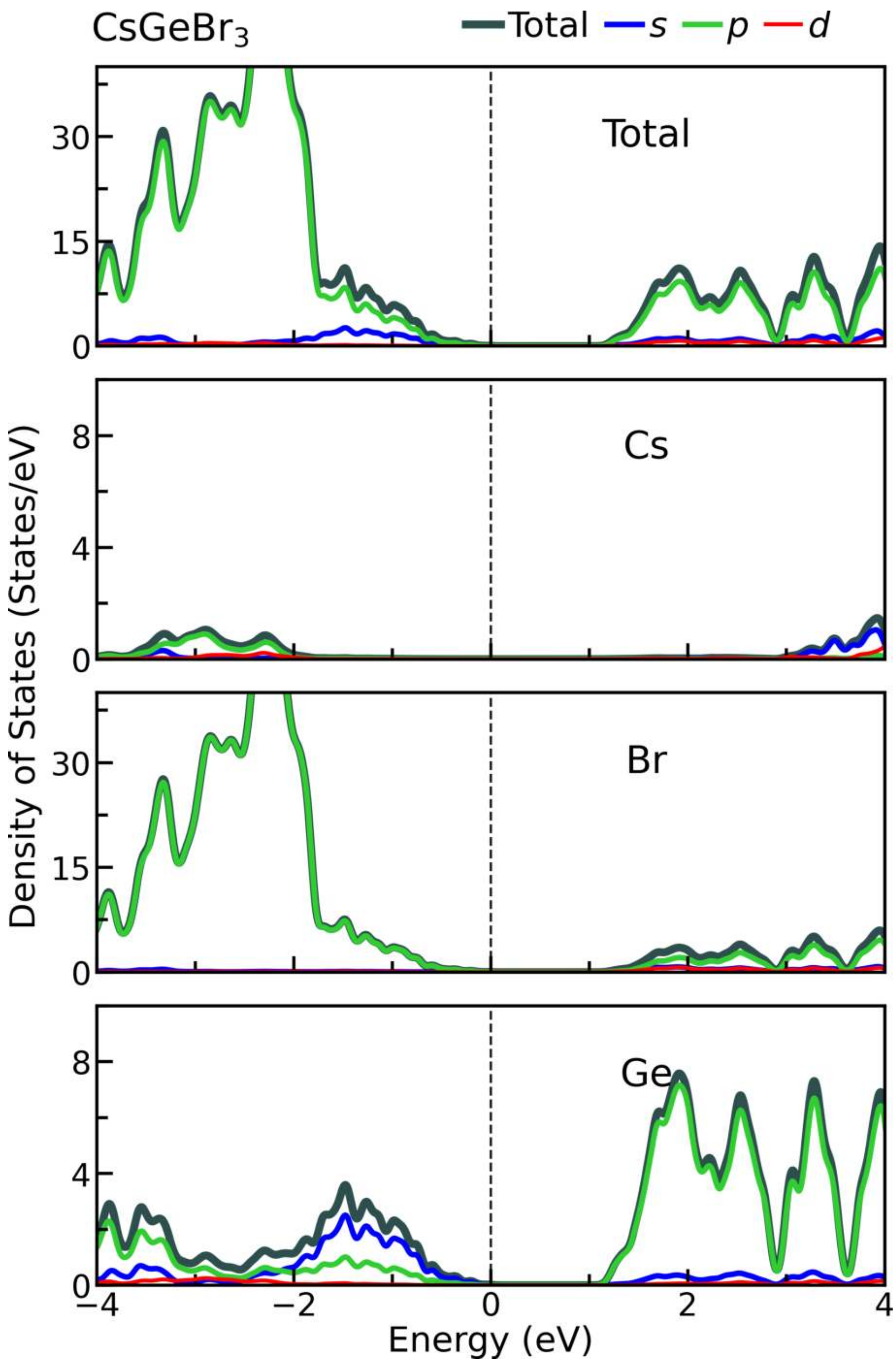

Figure S-107. Density of states of $CsGeBr_3$ at PBE+D3 level, for each atomic species and projected only on s and p orbitals.

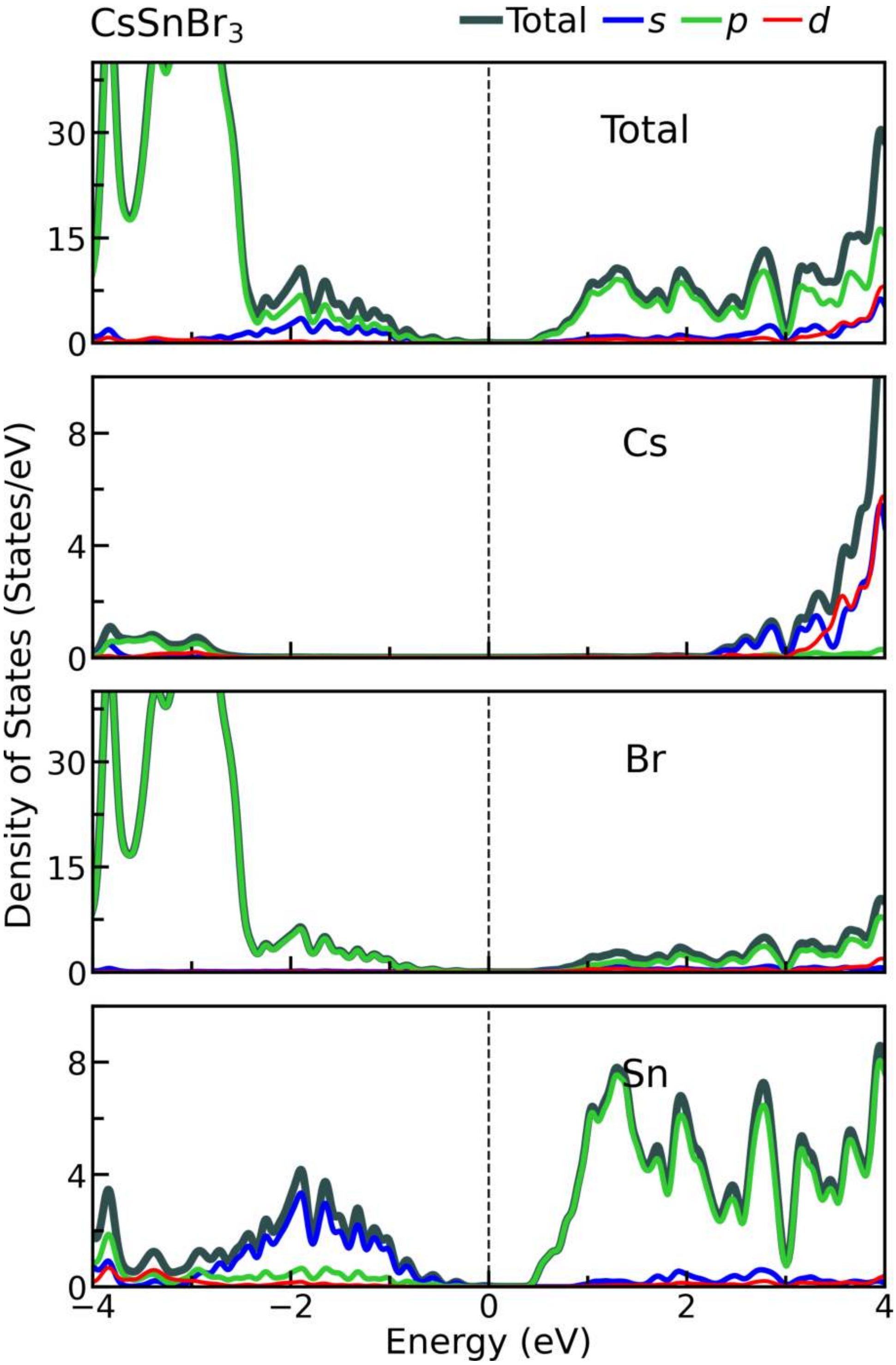

Figure S-108. Density of states of $CsSnBr_3$ at PBE+D3 level, for each atomic species and projected only on s and p orbitals.

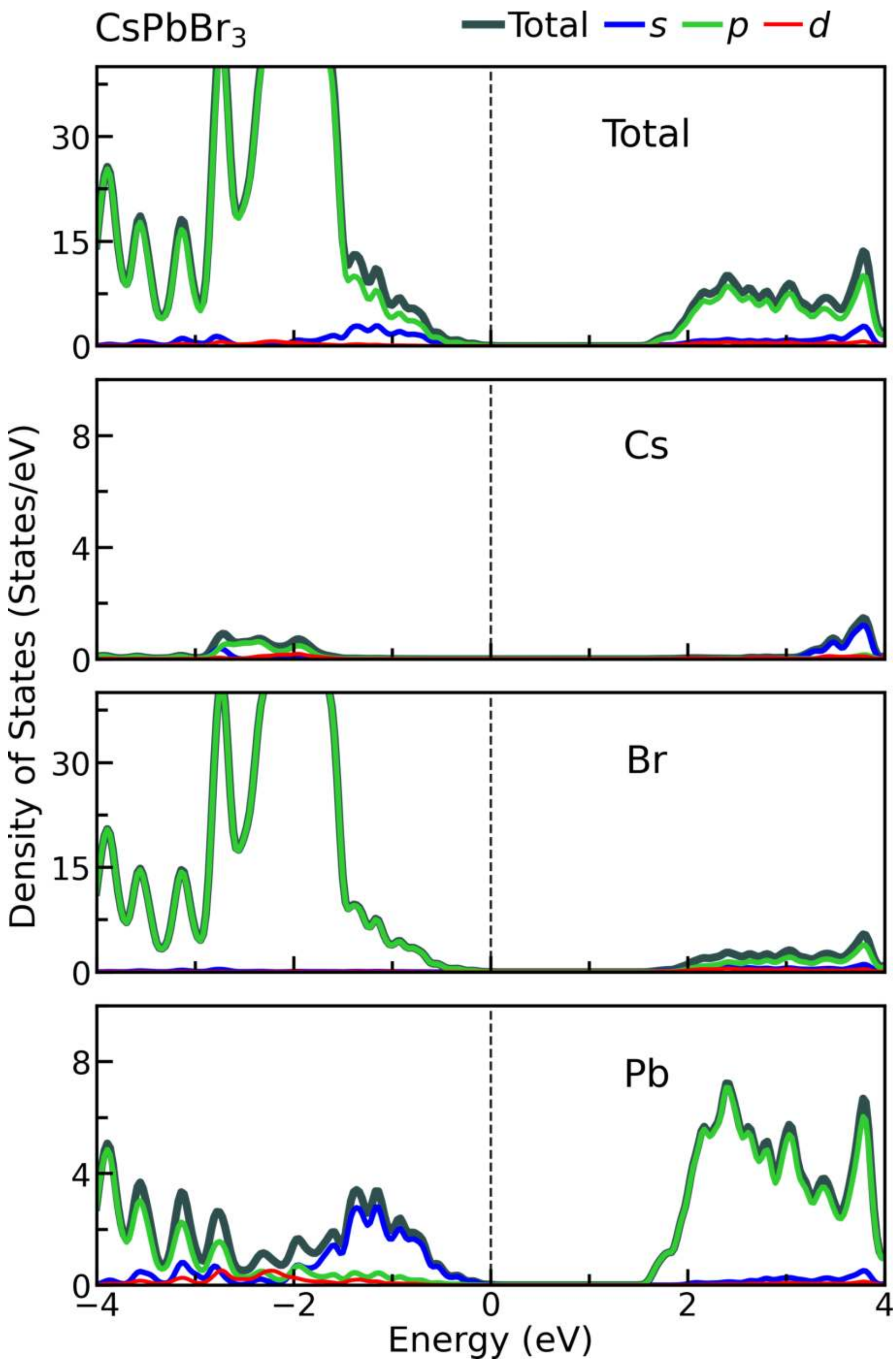

Figure S-109. Density of states of $CsPbBr_3$ at PBE+D3 level, for each atomic species and projected only on *s* and *p* orbitals.

S-8.6.2 Resume for Mixtures with One and Two Divalent Metals

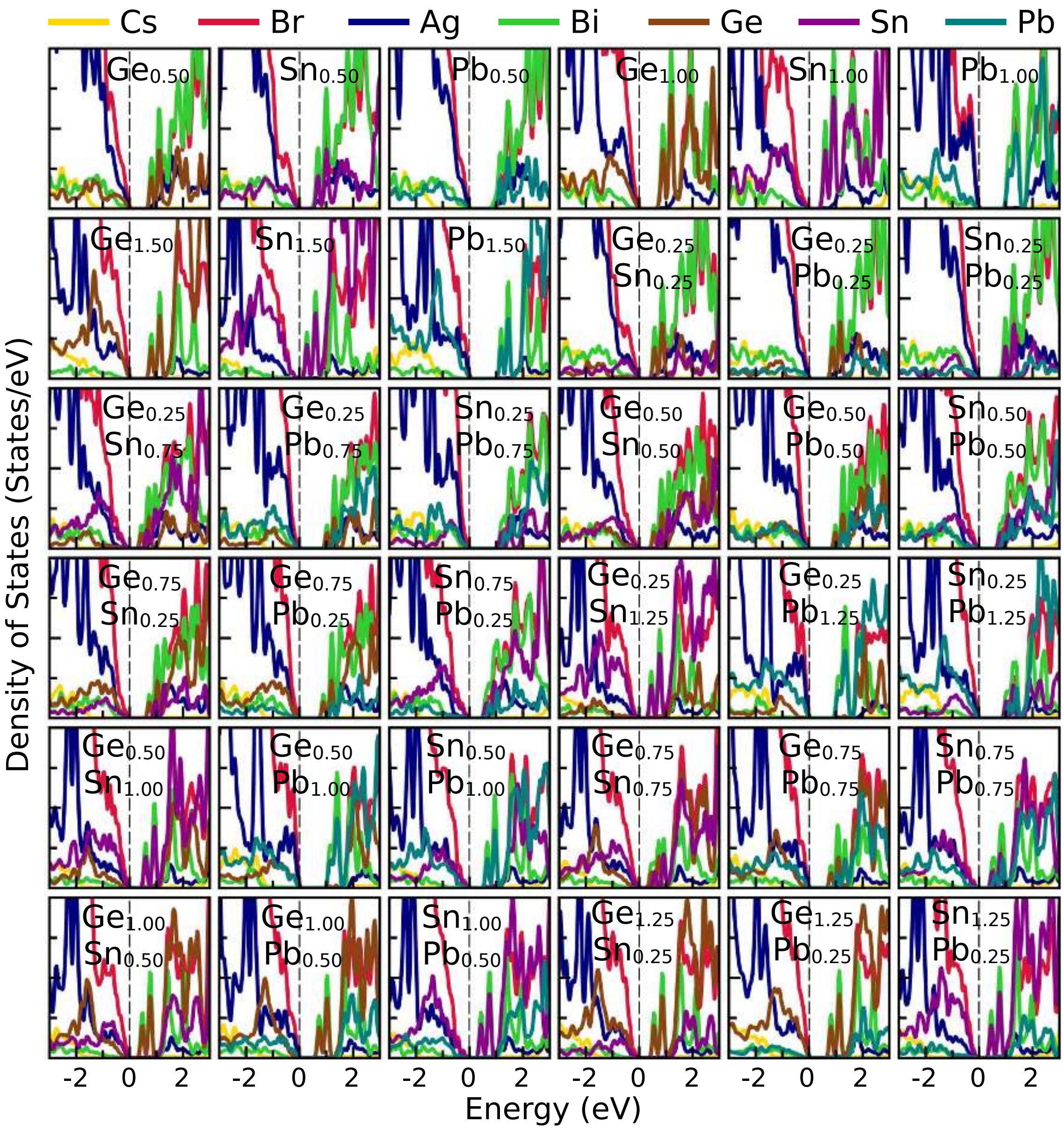

Figure S-110. Density of states of all mixtures with one and two divalent metals. For simplicity only the divalent metal within the mixture is mentioned in each compound. For mixtures with one divalent metal, i.e., $Cs_2Ag_xBi_xB_yBr_6$, the Ag and Bi composition is obtained through $x = 2 - y$, and for mixtures with two divalent metals, i.e., $Cs_2Ag_xBi_xB_yB'_zBr_6$, the Ag and Bi composition is obtained through $x = 2 - y - z$.

S-8.6.3 Mixtures with One Divalent Metal

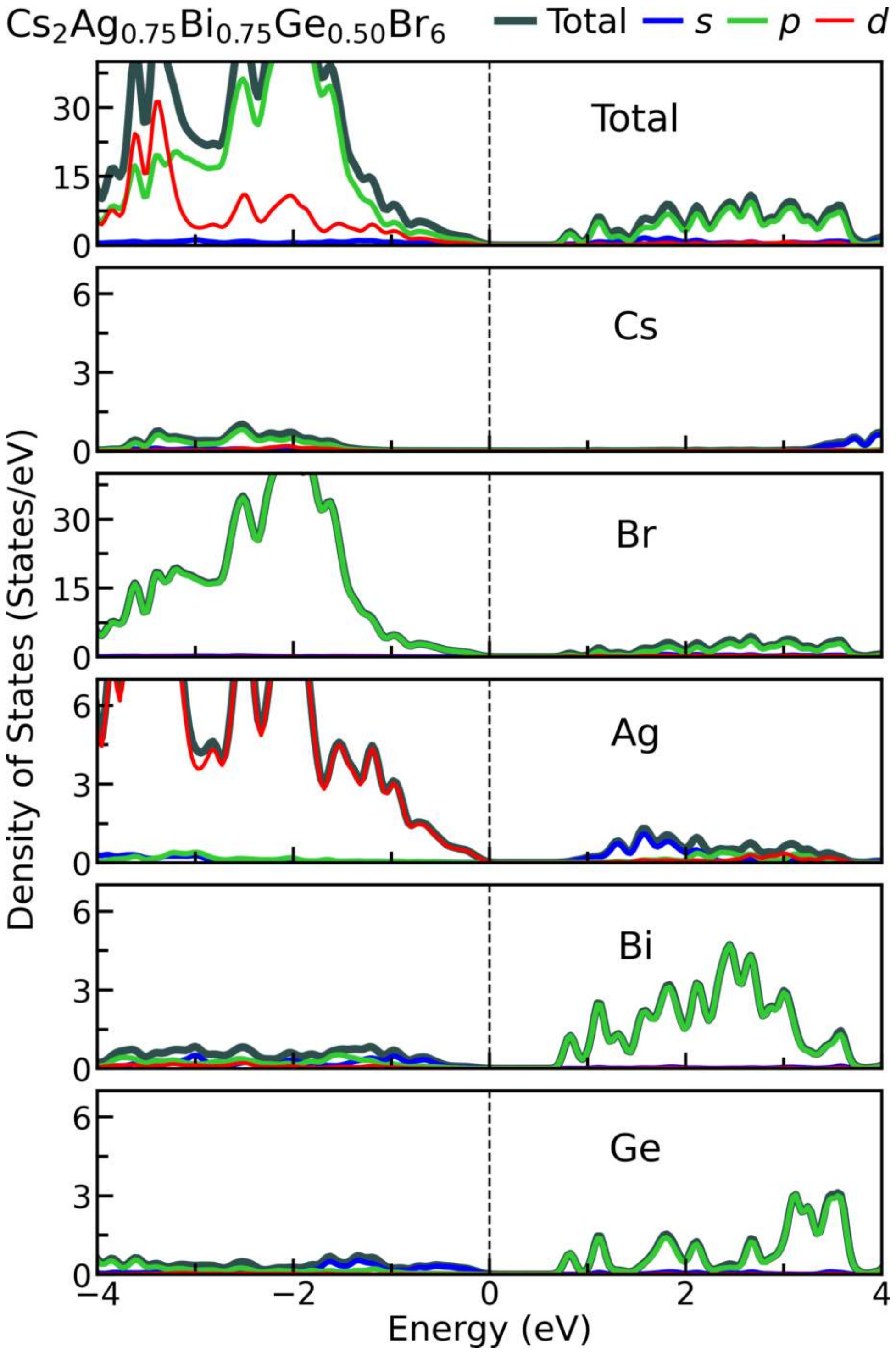

Figure S-111. Density of states of $Cs_2Ag_{0.75}Bi_{0.75}Ge_{0.50}Br_6$ at PBE+D3 level, for each atomic species and projected only on *s* and *p* orbitals.

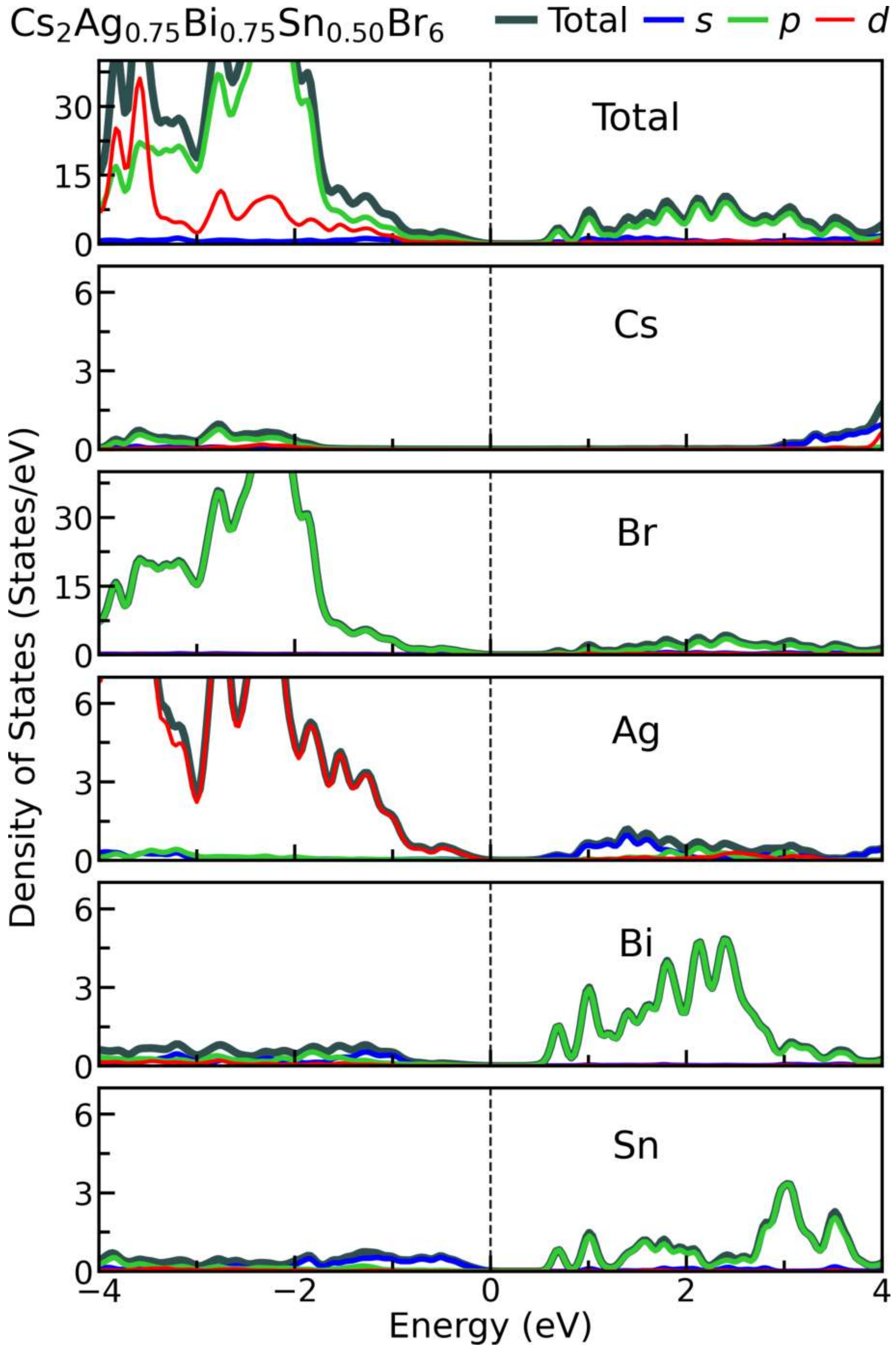

Figure S-112. Density of states of $Cs_2Ag_{0.75}Bi_{0.75}Sn_{0.50}Br_6$ at PBE+D3 level, for each atomic species and projected only on *s* and *p* orbitals.

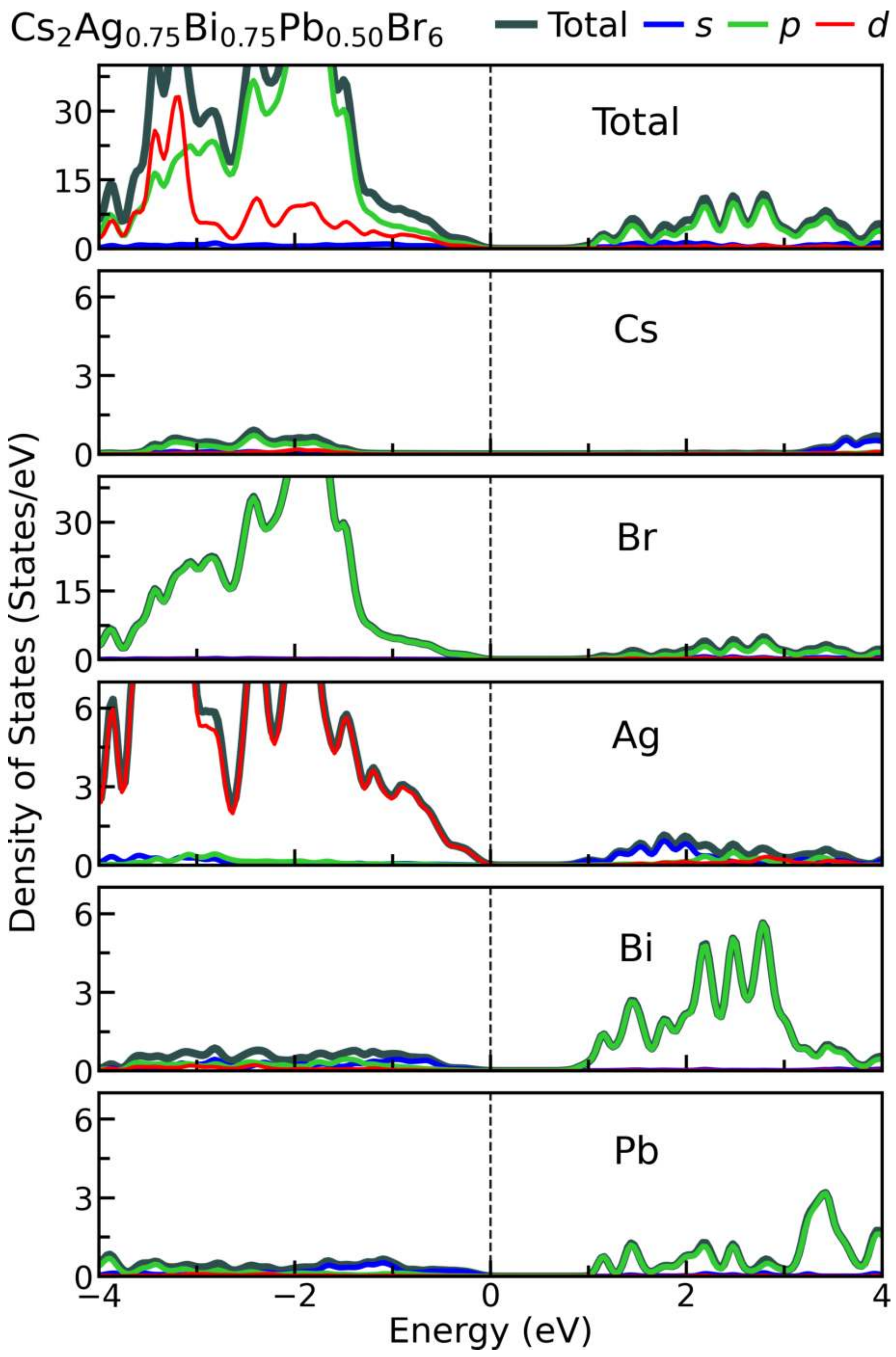

Figure S-113. Density of states of $Cs_2Ag_{0.75}Bi_{0.75}Pb_{0.50}Br_6$ at PBE+D3 level, for each atomic species and projected only on s and p orbitals.

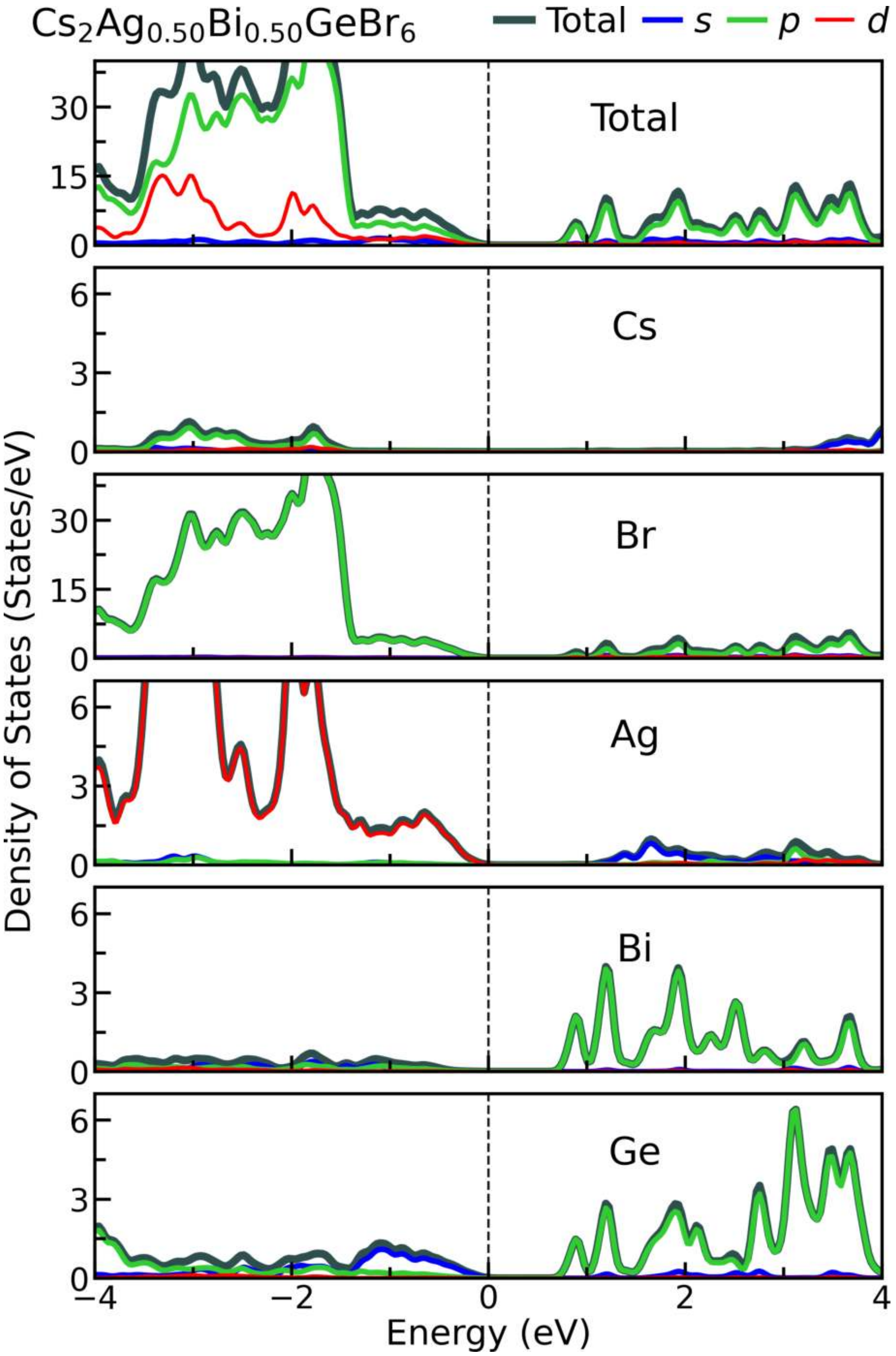

Figure S-114. Density of states of $Cs_2Ag_{0.50}Bi_{0.50}GeBr_6$ at PBE+D3 level, for each atomic species and projected only on *s* and *p* orbitals.

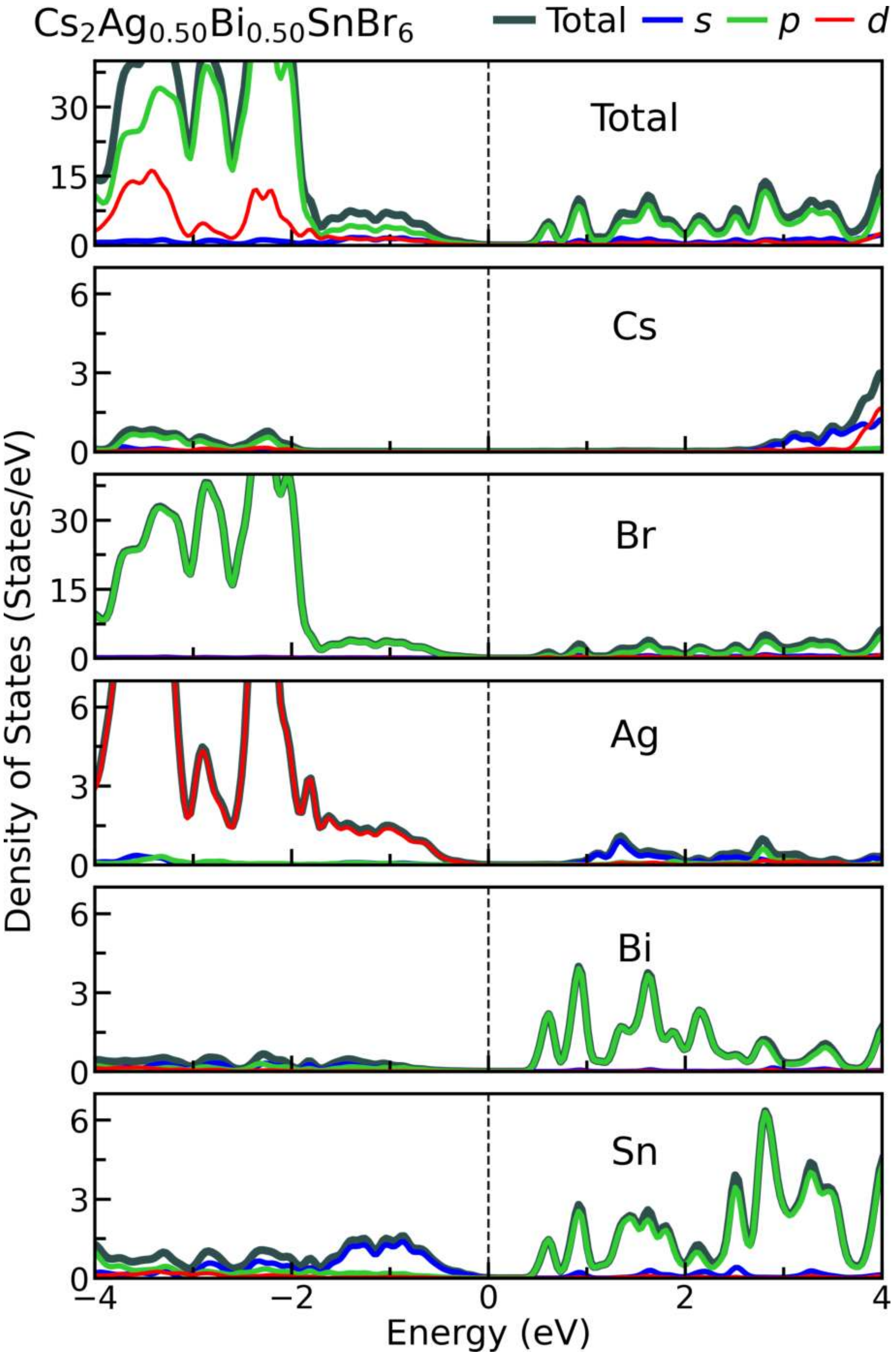

Figure S-115. Density of states of $Cs_2Ag_{0.50}Bi_{0.50}SnBr_6$ at PBE+D3 level, for each atomic species and projected only on s and p orbitals.

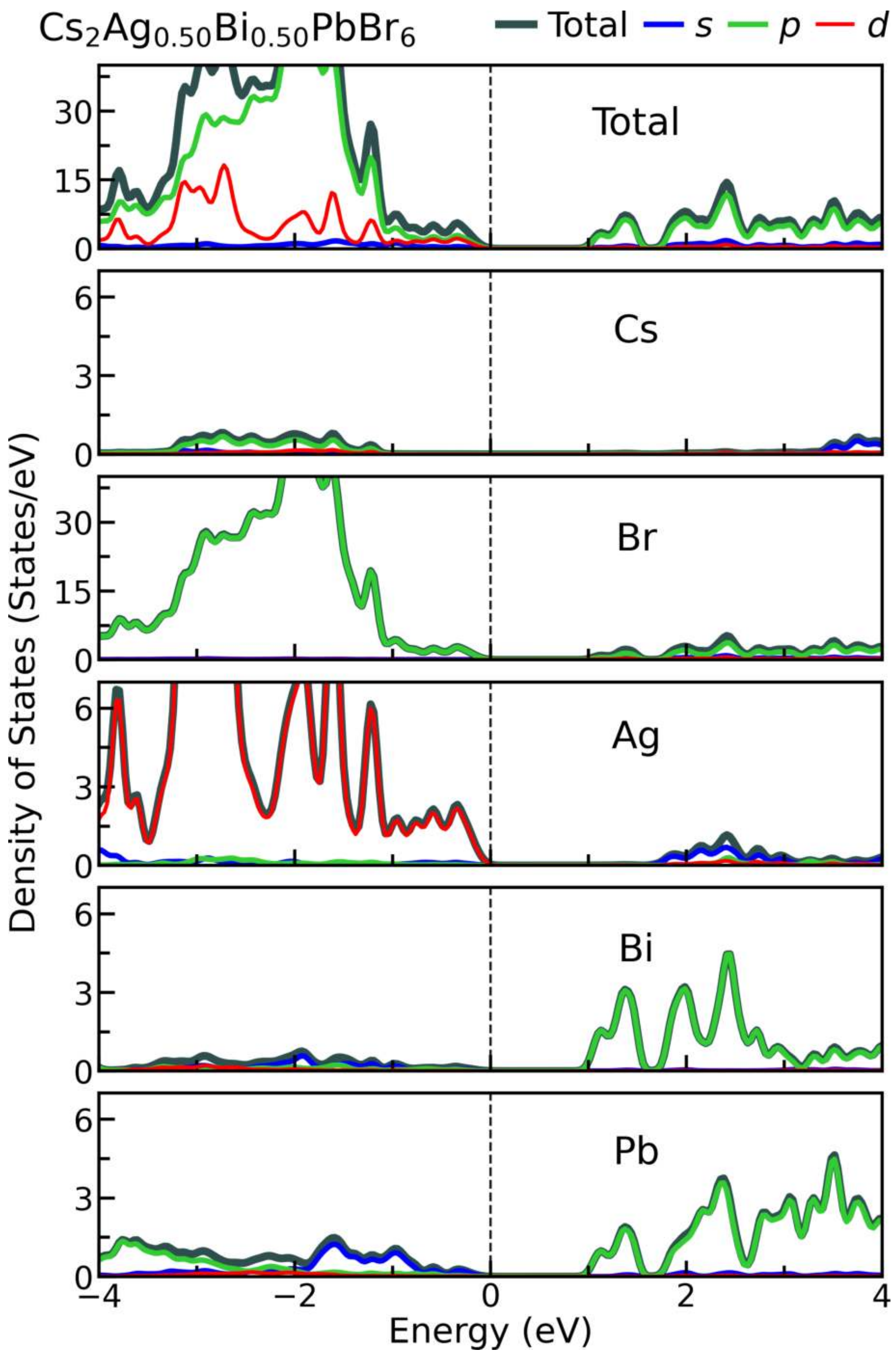

Figure S-116. Density of states of $Cs_2Ag_{0.50}Bi_{0.50}PbBr_6$ at PBE+D3 level, for each atomic species and projected only on s and p orbitals.

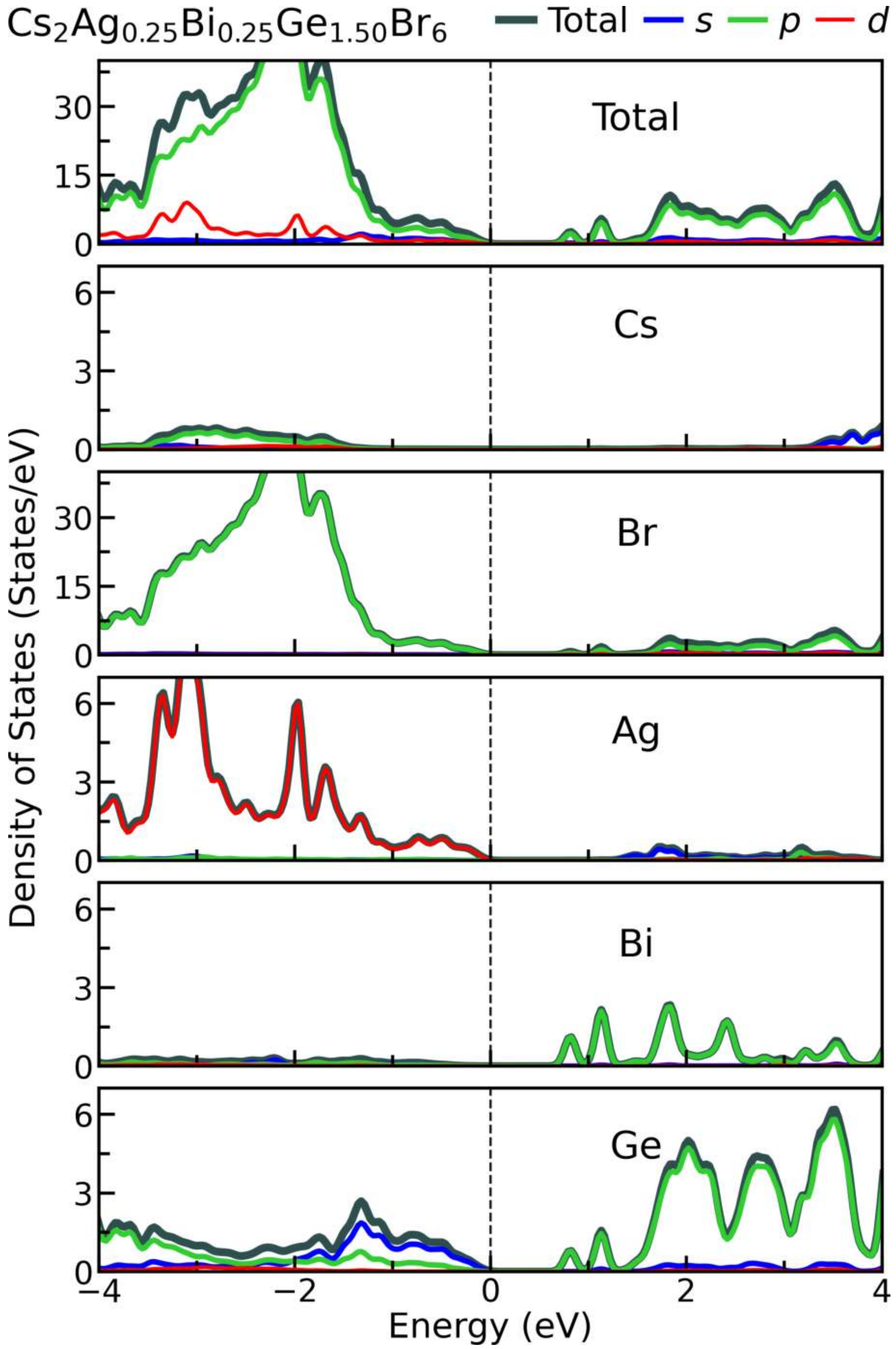

Figure S-117. Density of states of $Cs_2Ag_{0.25}Bi_{0.25}Ge_{1.50}Br_6$ at PBE+D3 level, for each atomic species and projected only on s and p orbitals.

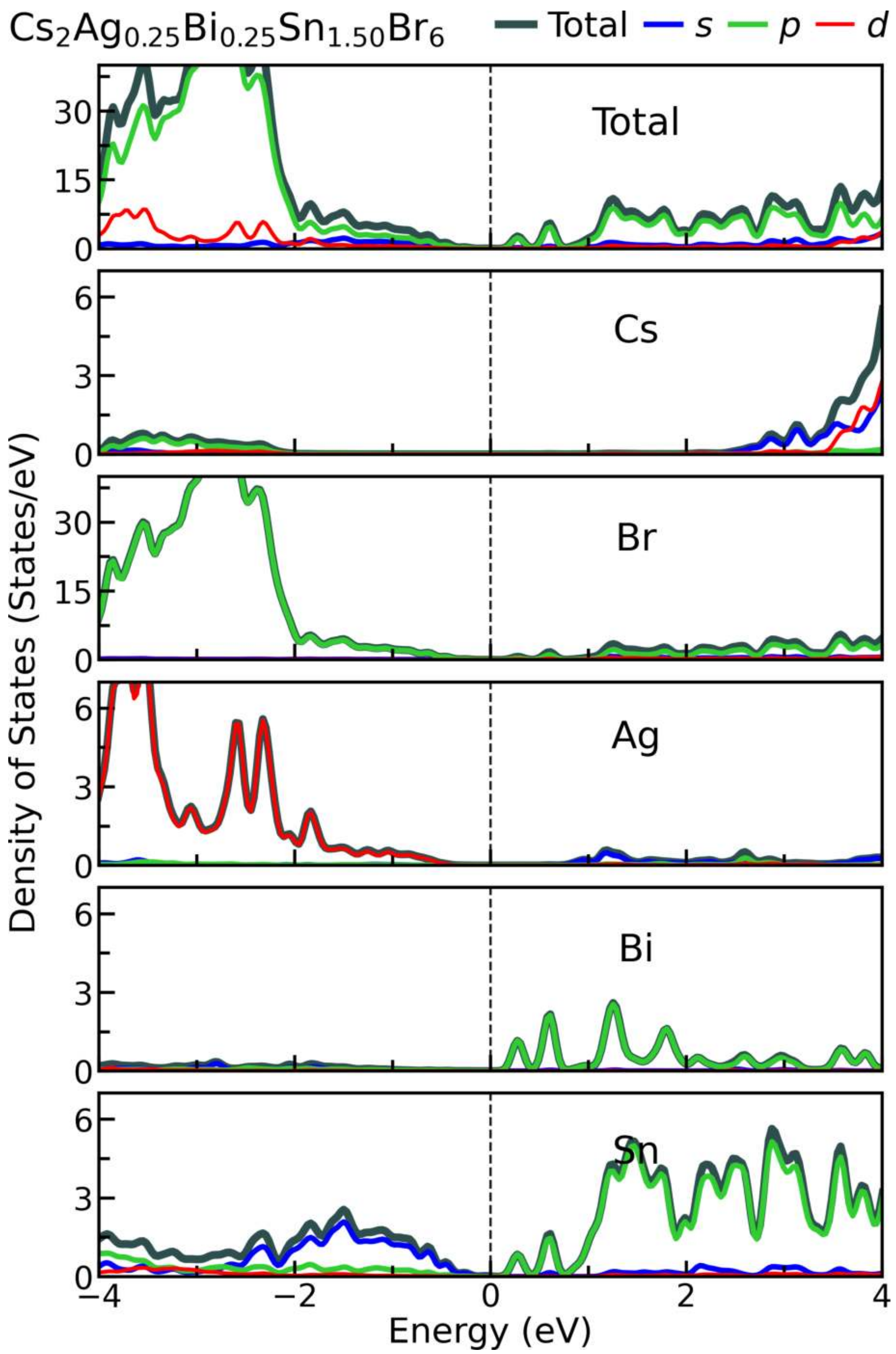

Figure S-118. Density of states of $Cs_2Ag_{0.25}Bi_{0.25}Sn_{1.50}Br_6$ at PBE+D3 level, for each atomic species and projected only on s and p orbitals.

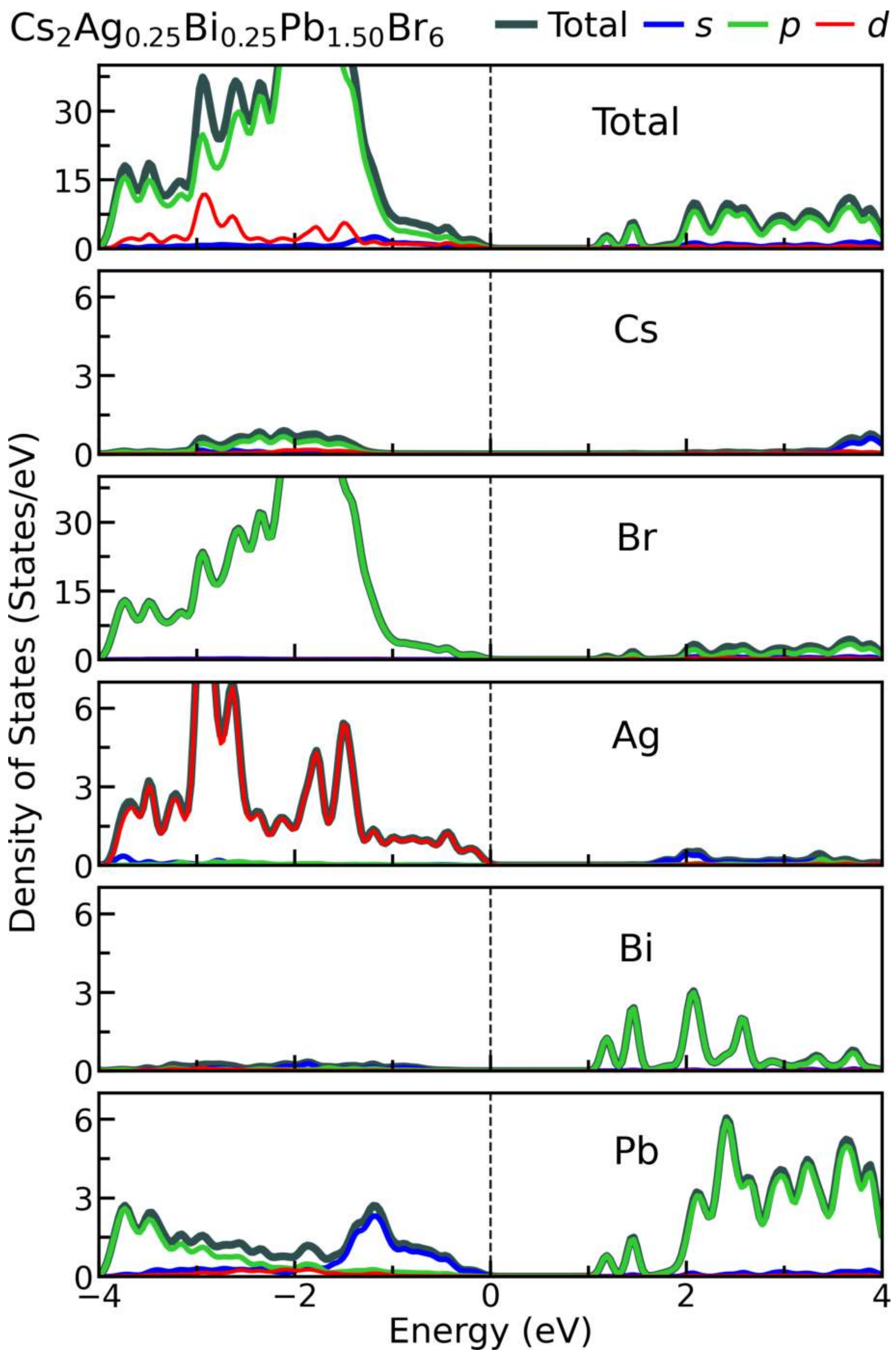

Figure S-119. Density of states of $Cs_2Ag_{0.25}Bi_{0.25}Pb_{1.50}Br_6$ at PBE+D3 level, for each atomic species and projected only on *s* and *p* orbitals.

S-8.6.4 Mixtures with Two Divalent Metals

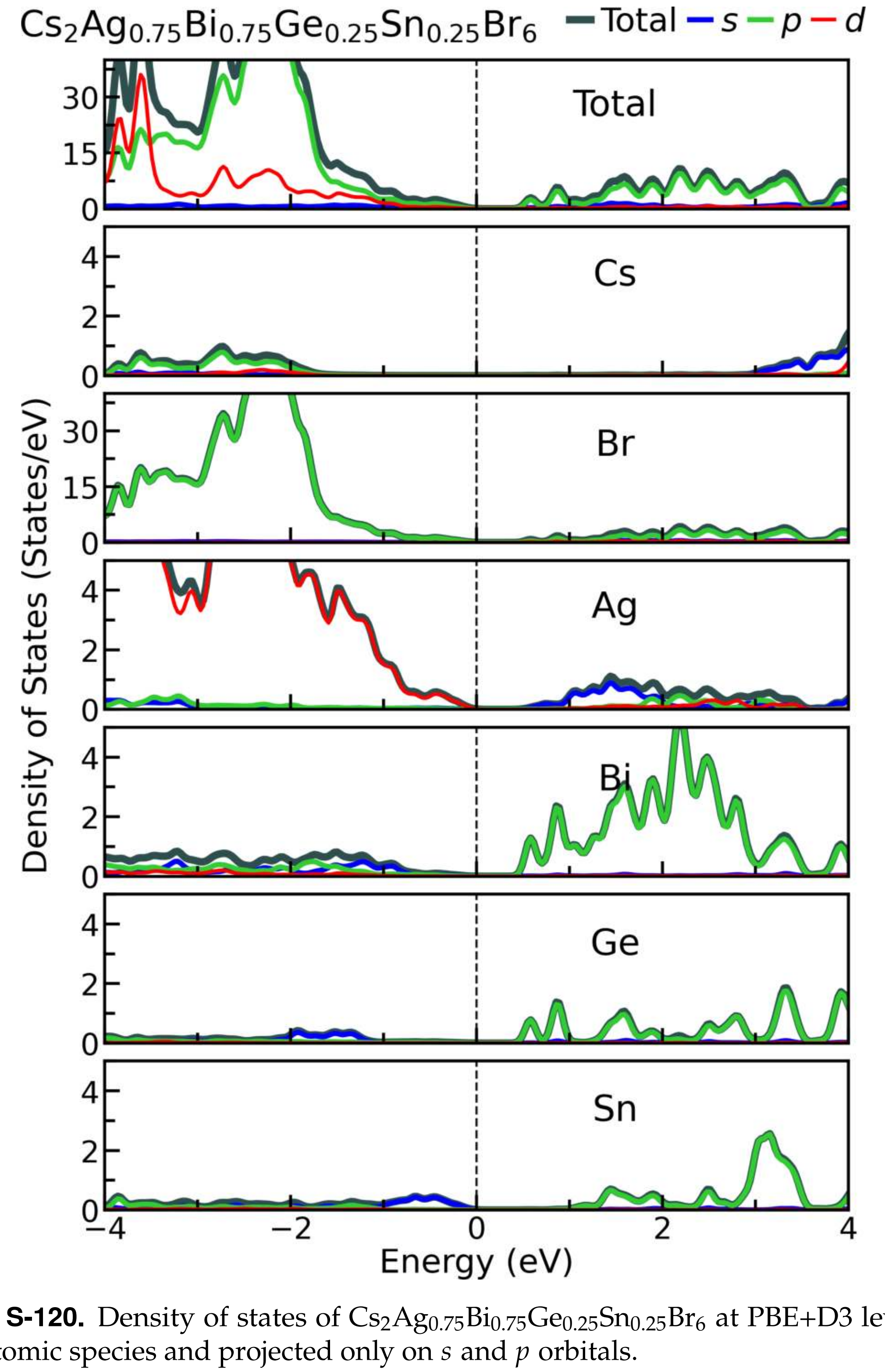

Figure S-120. Density of states of $Cs_2Ag_{0.75}Bi_{0.75}Ge_{0.25}Sn_{0.25}Br_6$ at PBE+D3 level, for each atomic species and projected only on s and p orbitals.

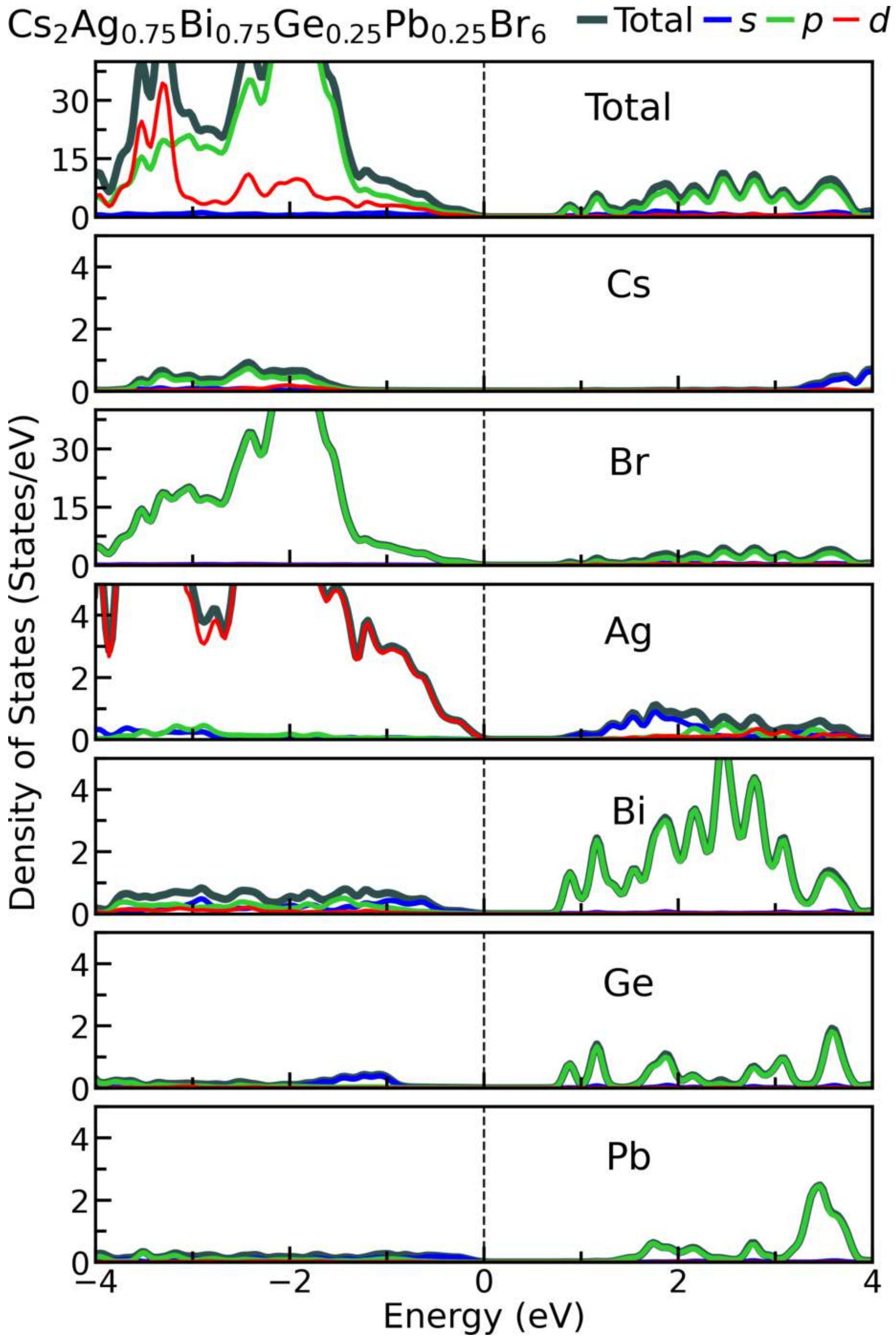

Figure S-121. Density of states of $Cs_2Ag_{0.75}Bi_{0.75}Ge_{0.25}Pb_{0.25}Br_6$ at PBE+D3 level, for each atomic species and projected only on *s* and *p* orbitals.

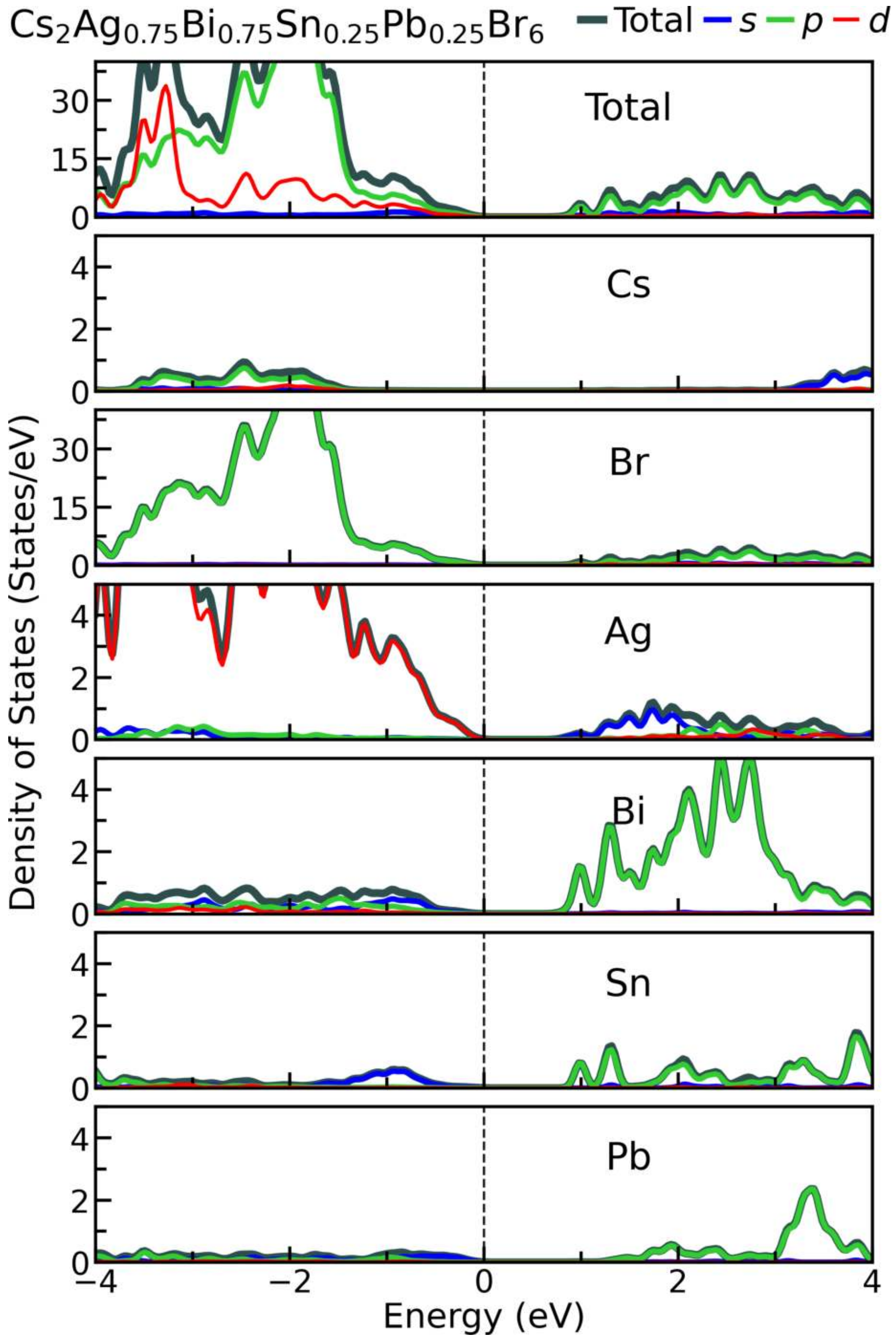

Figure S-122. Density of states of $Cs_2Ag_{0.75}Bi_{0.75}Sn_{0.25}Pb_{0.25}Br_6$ at PBE+D3 level, for each atomic species and projected only on *s* and *p* orbitals.

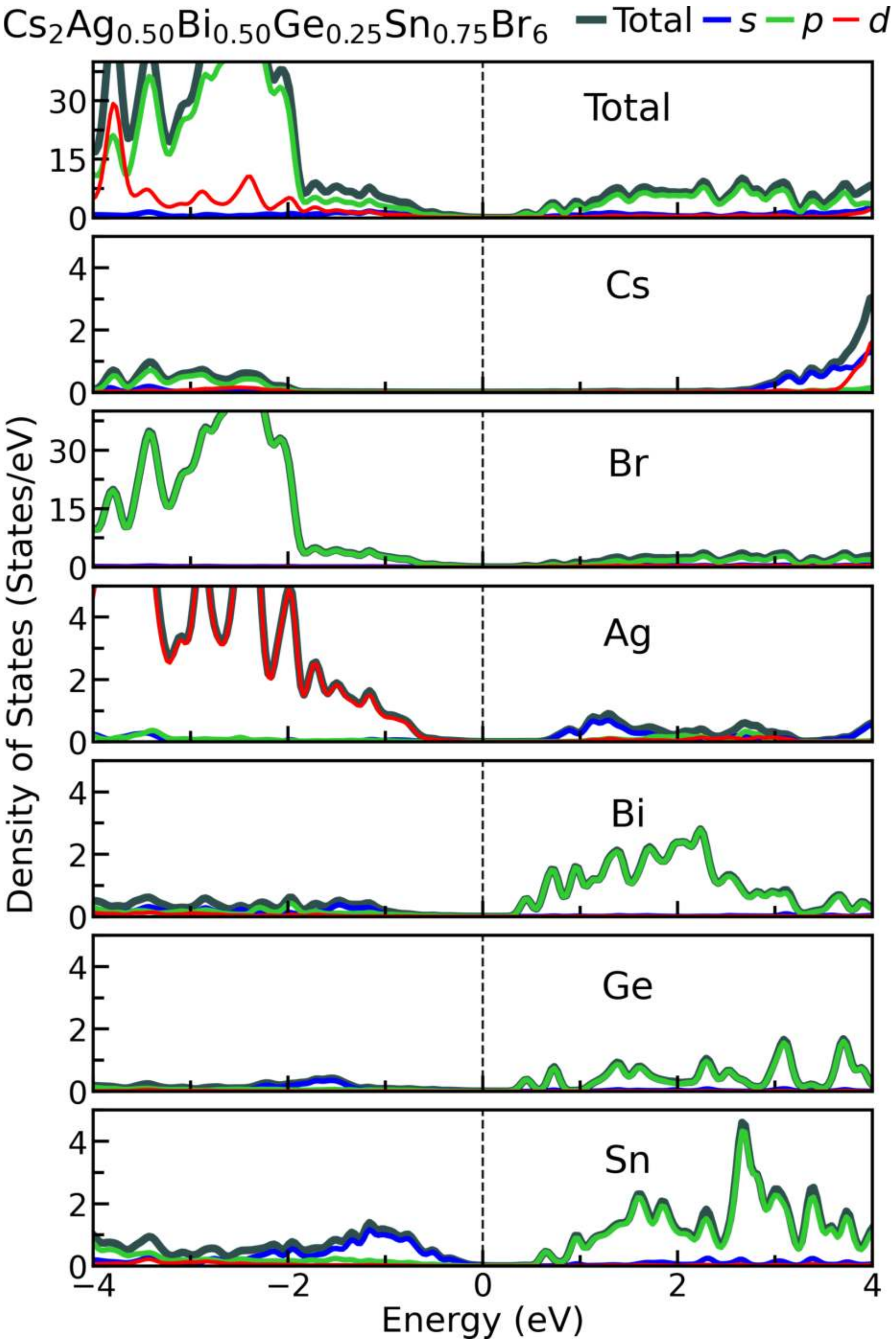

Figure S-123. Density of states of $Cs_2Ag_{0.50}Bi_{0.50}Ge_{0.25}Sn_{0.75}Br_6$ at PBE+D3 level, for each atomic species and projected only on *s* and *p* orbitals.

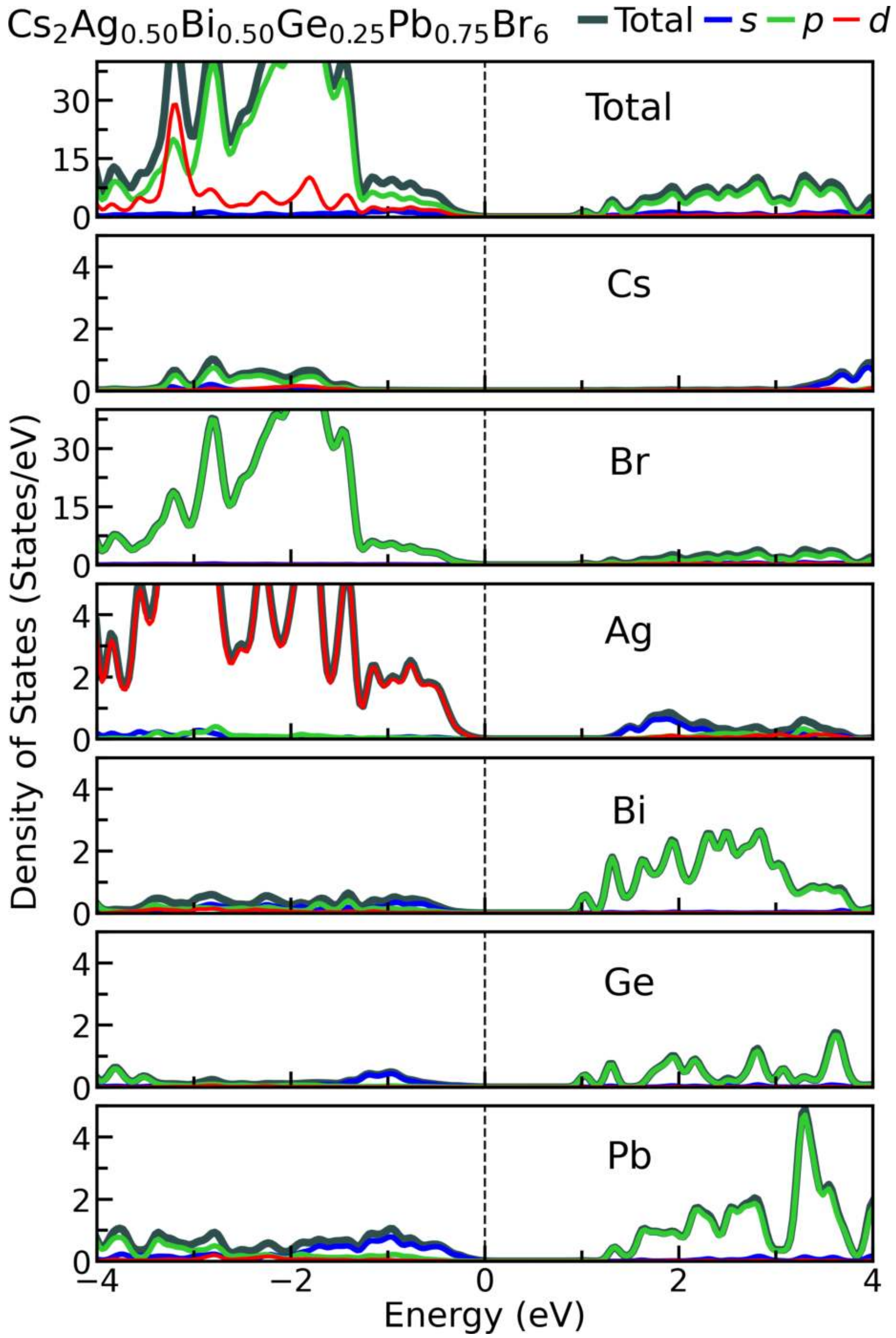

Figure S-124. Density of states of $Cs_2Ag_{0.50}Bi_{0.50}Ge_{0.25}Pb_{0.75}Br_6$ at PBE+D3 level, for each atomic species and projected only on *s* and *p* orbitals.

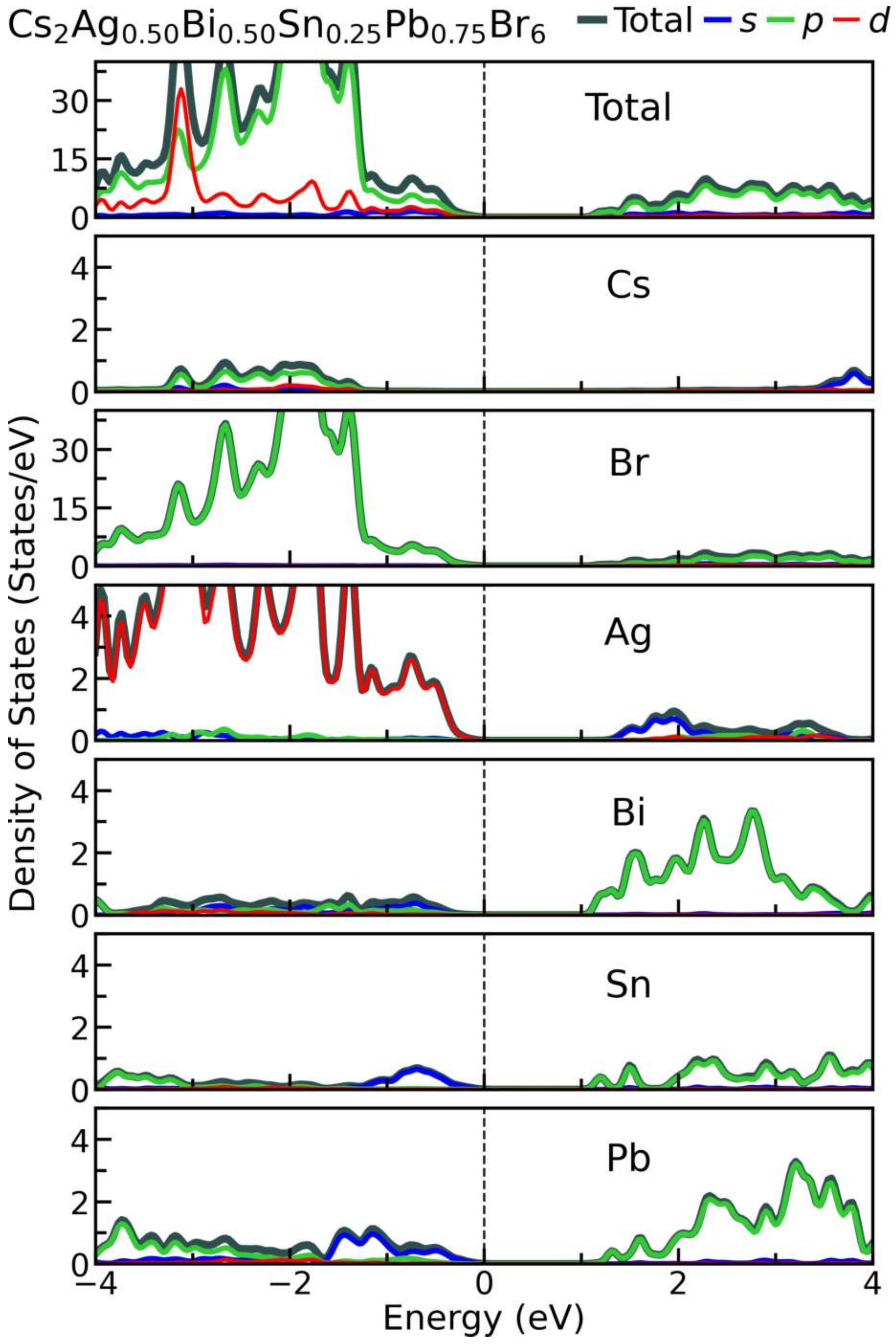

Figure S-125. Density of states of $Cs_2Ag_{0.50}Bi_{0.50}Sn_{0.25}Pb_{0.75}Br_6$ at PBE+D3 level, for each atomic species and projected only on *s* and *p* orbitals.

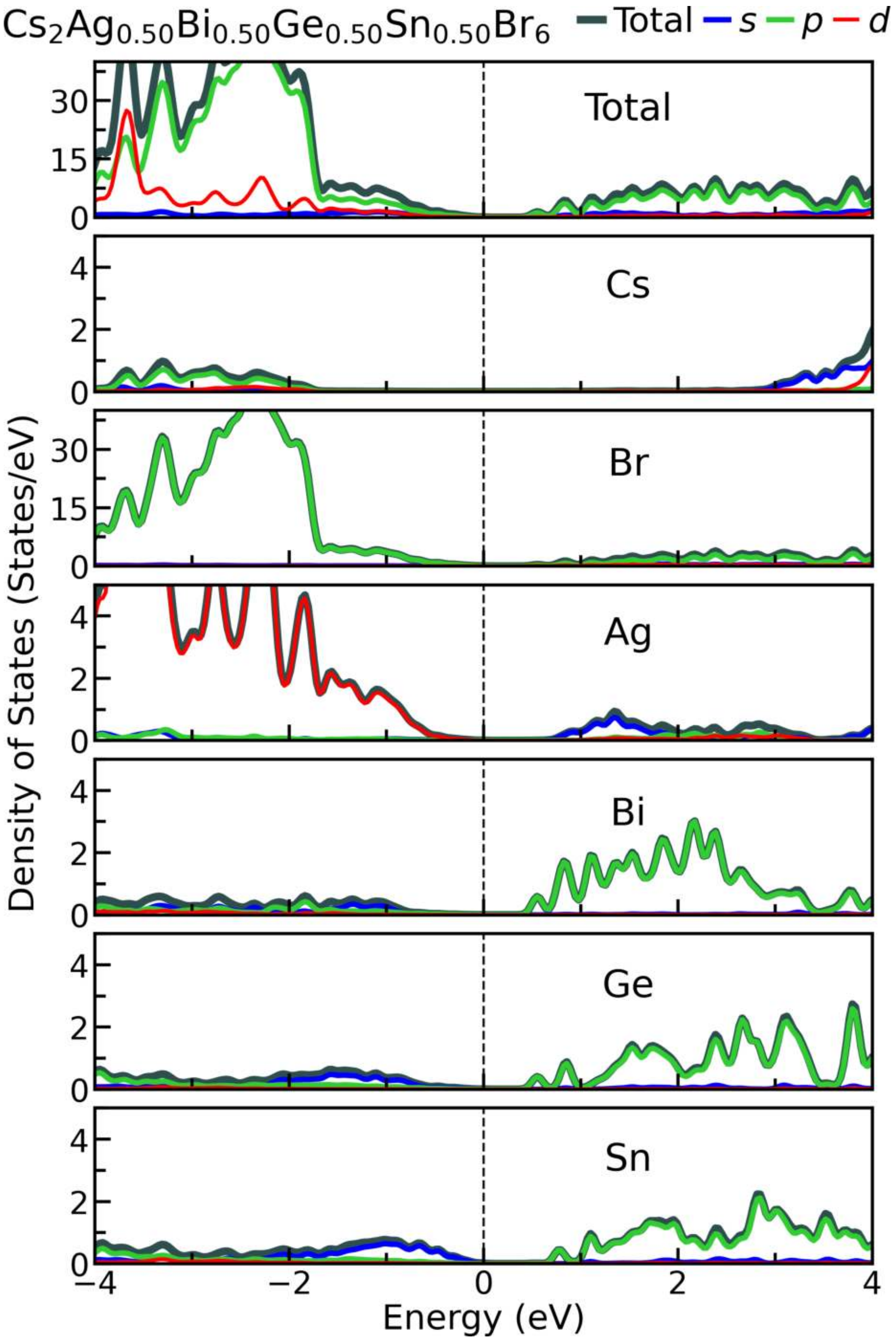

Figure S-126. Density of states of $Cs_2Ag_{0.50}Bi_{0.50}Ge_{0.50}Sn_{0.50}Br_6$ at PBE+D3 level, for each atomic species and projected only on *s* and *p* orbitals.

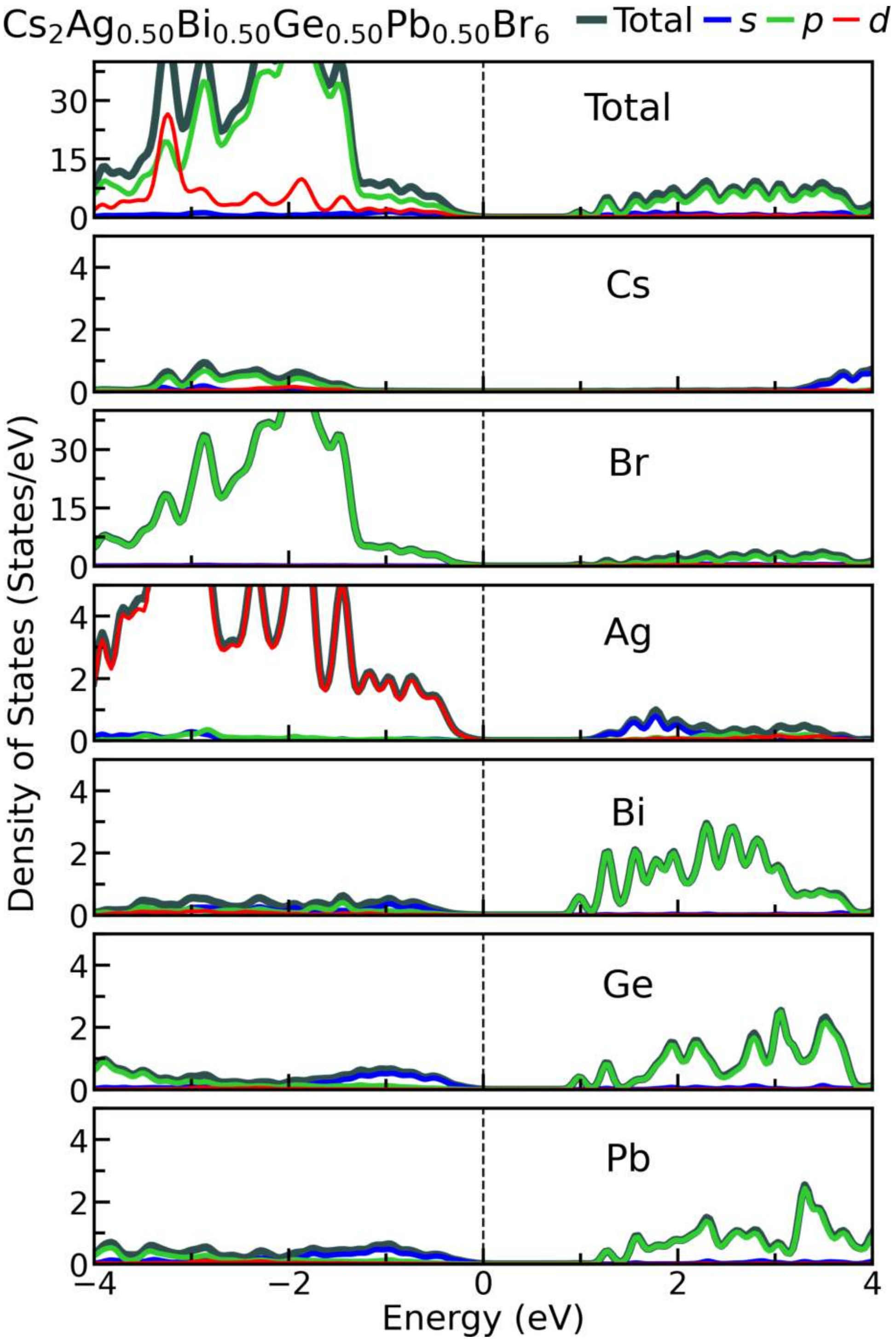

Figure S-127. Density of states of $Cs_2Ag_{0.50}Bi_{0.50}Ge_{0.50}Pb_{0.50}Br_6$ at PBE+D3 level, for each atomic species and projected only on *s* and *p* orbitals.

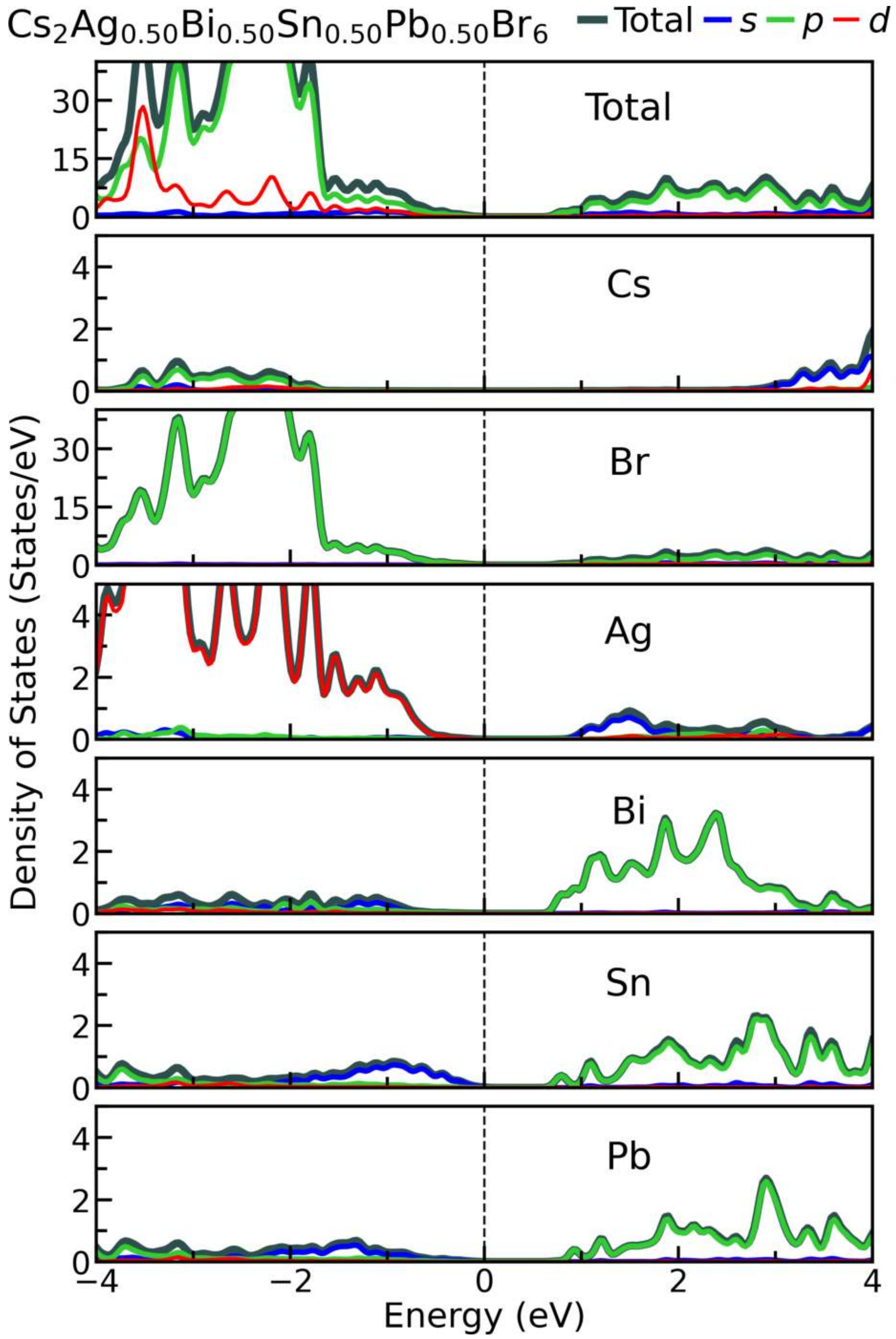

Figure S-128. Density of states of $Cs_2Ag_{0.50}Bi_{0.50}Sn_{0.50}Pb_{0.50}Br_6$ at PBE+D3 level, for each atomic species and projected only on *s* and *p* orbitals.

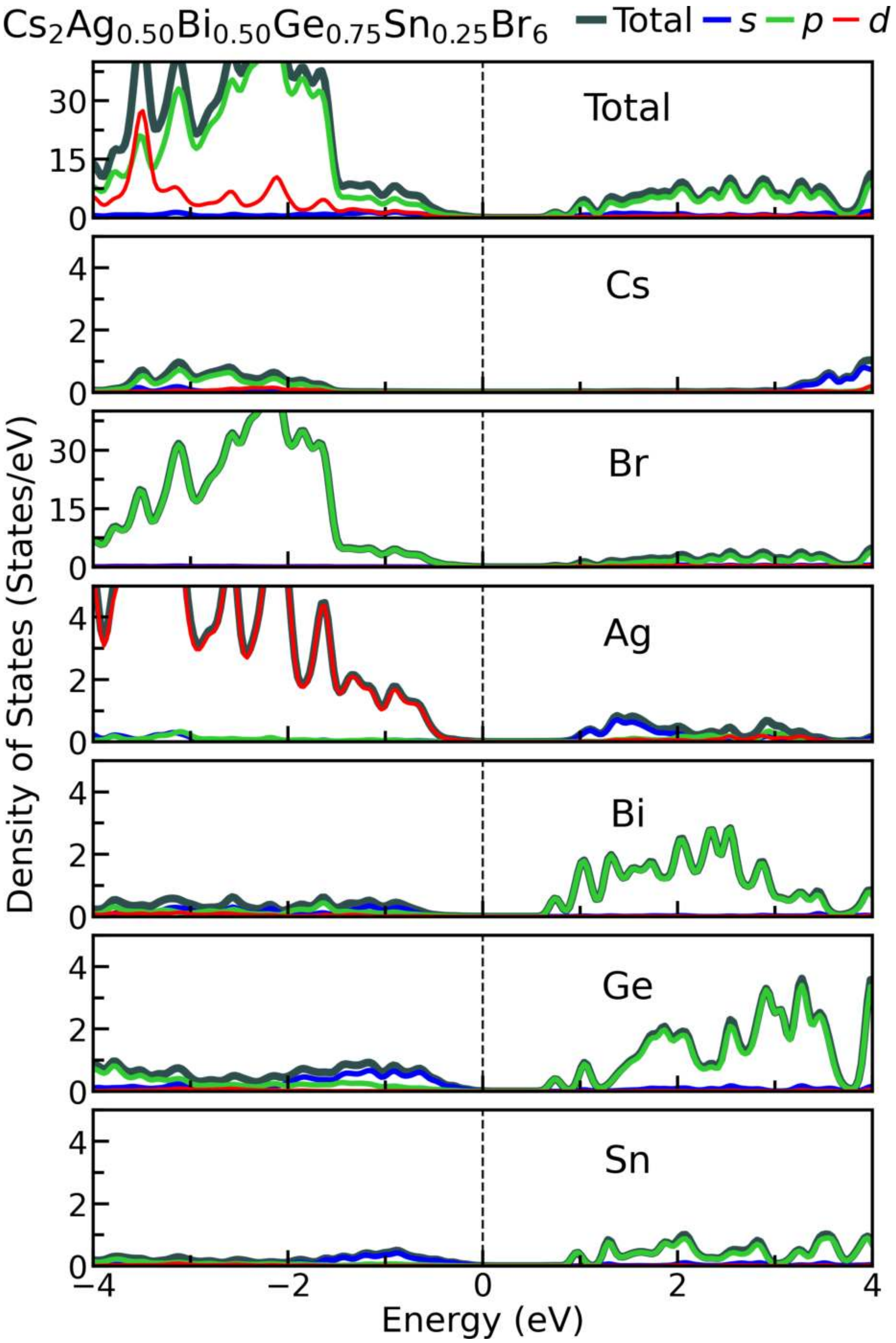

Figure S-129. Density of states of $Cs_2Ag_{0.50}Bi_{0.50}Ge_{0.75}Sn_{0.25}Br_6$ at PBE+D3 level, for each atomic species and projected only on *s* and *p* orbitals.

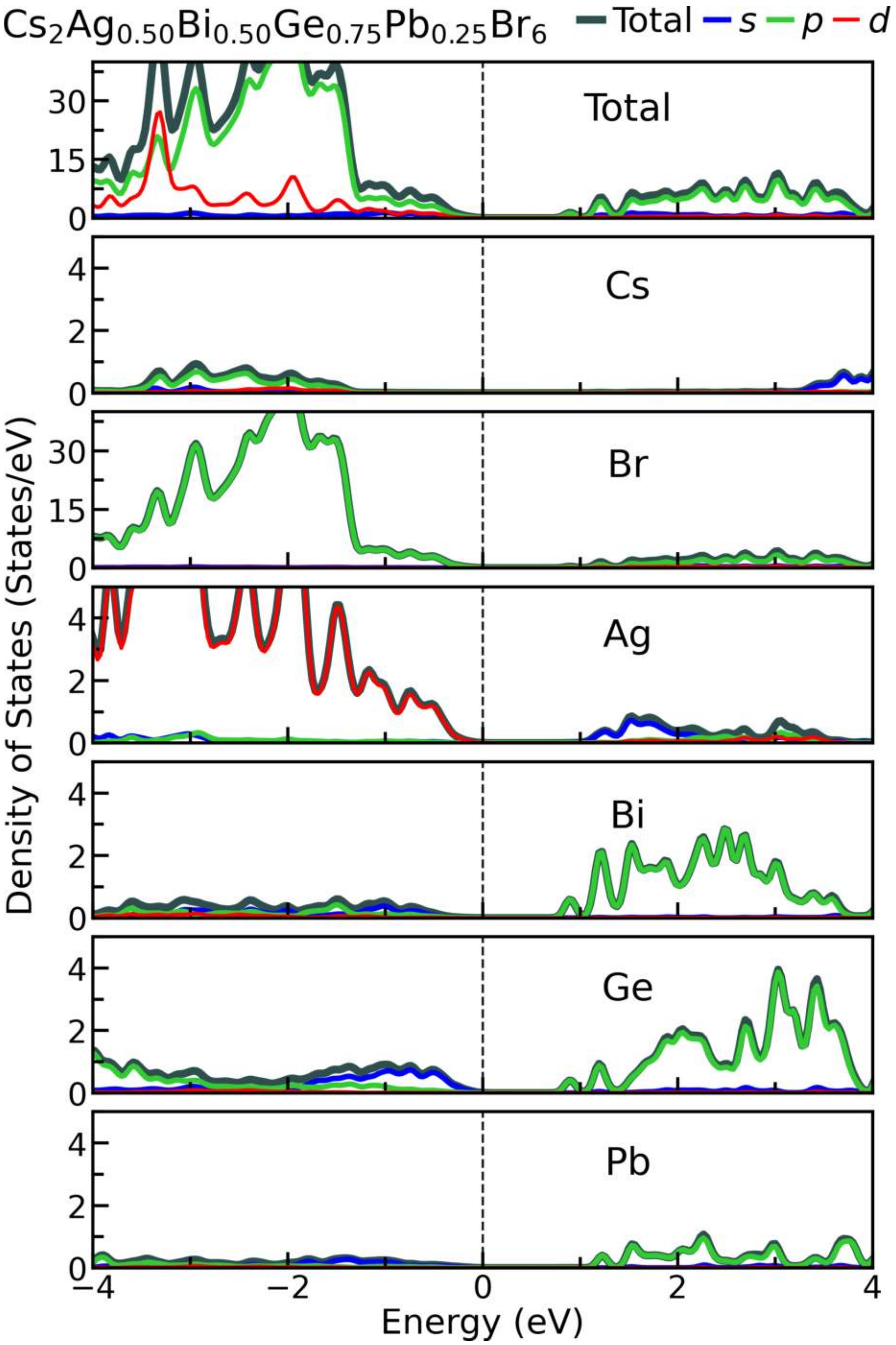

Figure S-130. Density of states of $Cs_2Ag_{0.50}Bi_{0.50}Ge_{0.75}Pb_{0.25}Br_6$ at PBE+D3 level, for each atomic species and projected only on *s* and *p* orbitals.

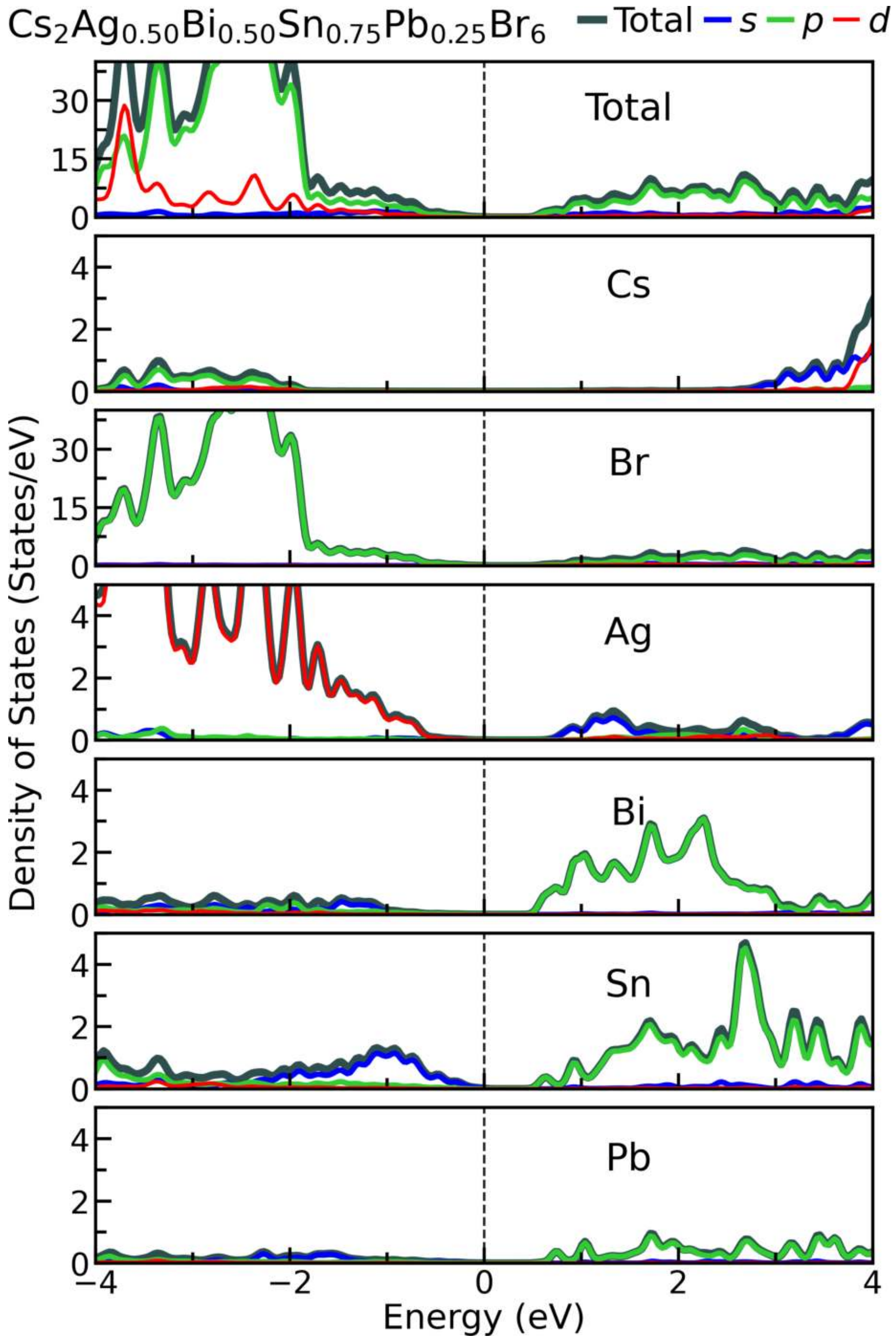

Figure S-131. Density of states of $Cs_2Ag_{0.50}Bi_{0.50}Sn_{0.75}Pb_{0.25}Br_6$ at PBE+D3 level, for each atomic species and projected only on *s* and *p* orbitals.

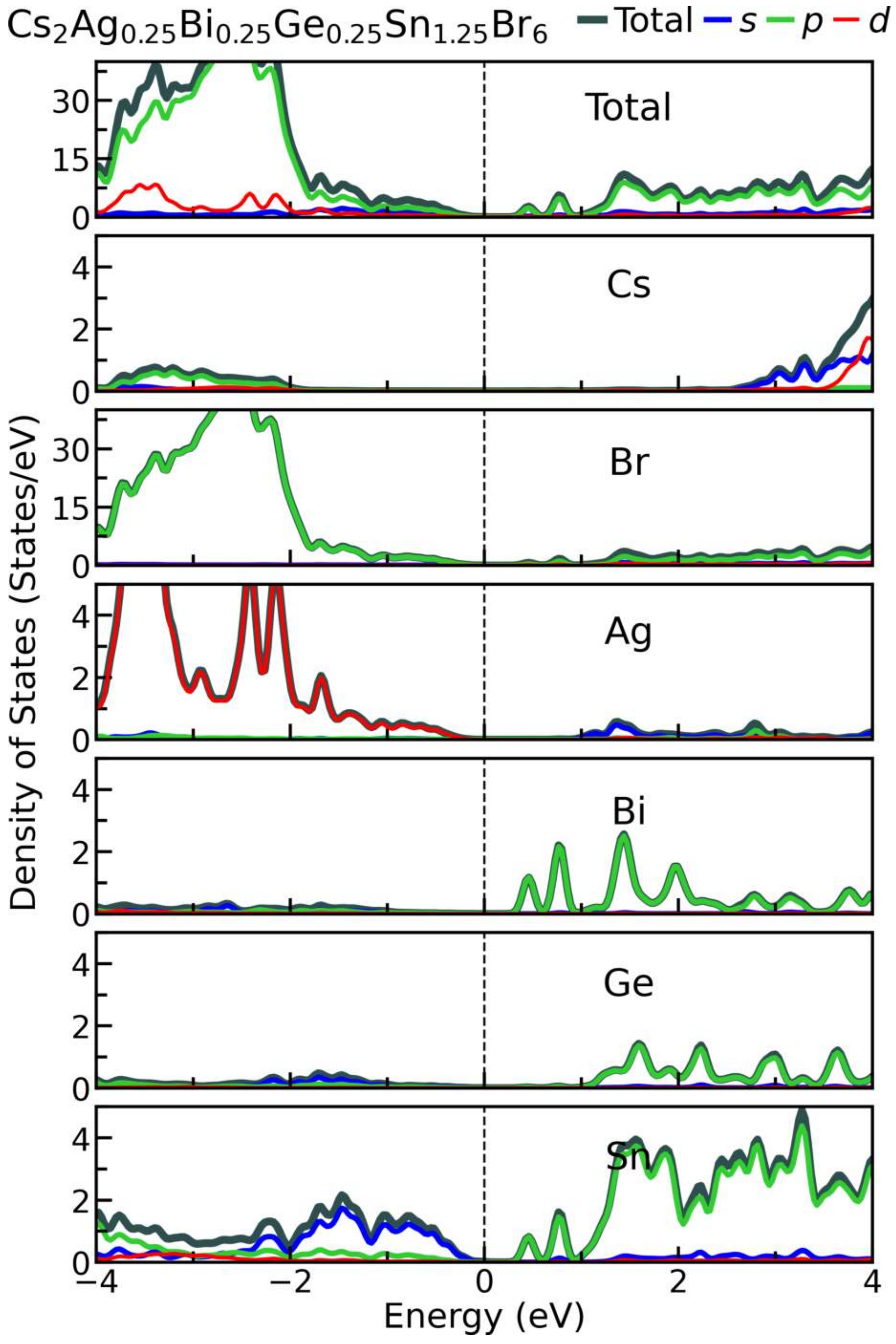

Figure S-132. Density of states of $Cs_2Ag_{0.25}Bi_{0.25}Ge_{0.25}Sn_{1.25}Br_6$ at PBE+D3 level, for each atomic species and projected only on *s* and *p* orbitals.

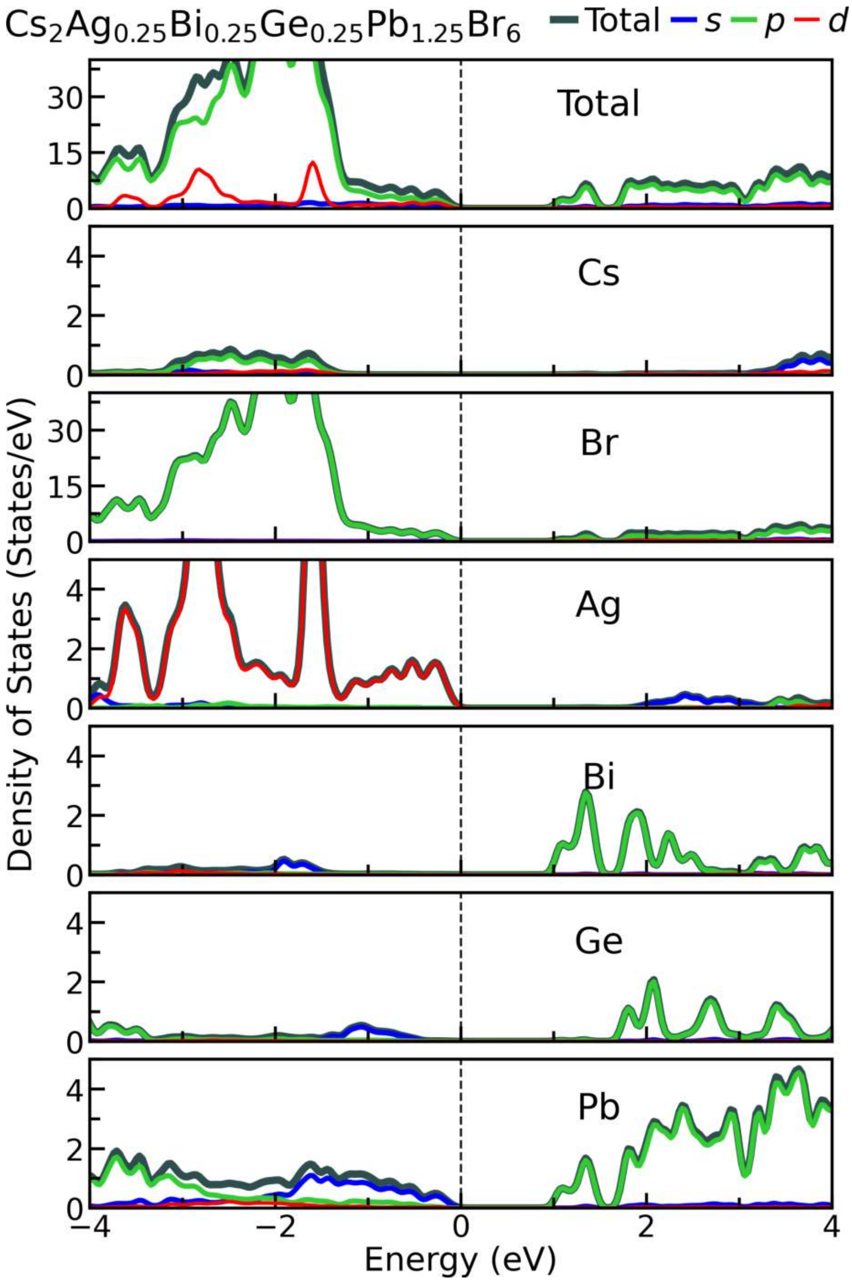

Figure S-133. Density of states of $Cs_2Ag_{0.25}Bi_{0.25}Ge_{0.25}Pb_{1.25}Br_6$ at PBE+D3 level, for each atomic species and projected only on *s* and *p* orbitals.

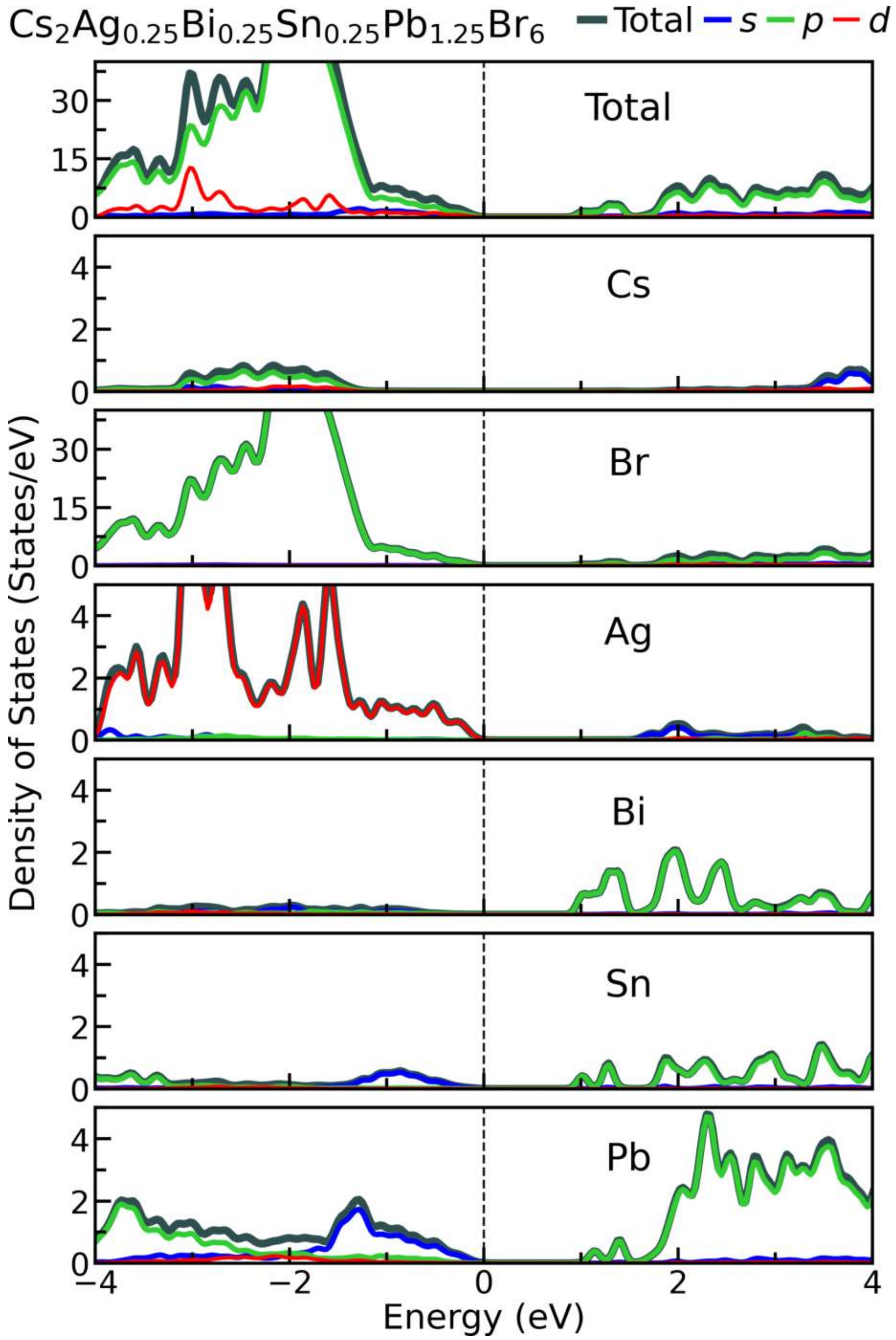

Figure S-134. Density of states of $Cs_2Ag_{0.25}Bi_{0.25}Sn_{0.25}Pb_{1.25}Br_6$ at PBE+D3 level, for each atomic species and projected only on *s* and *p* orbitals.

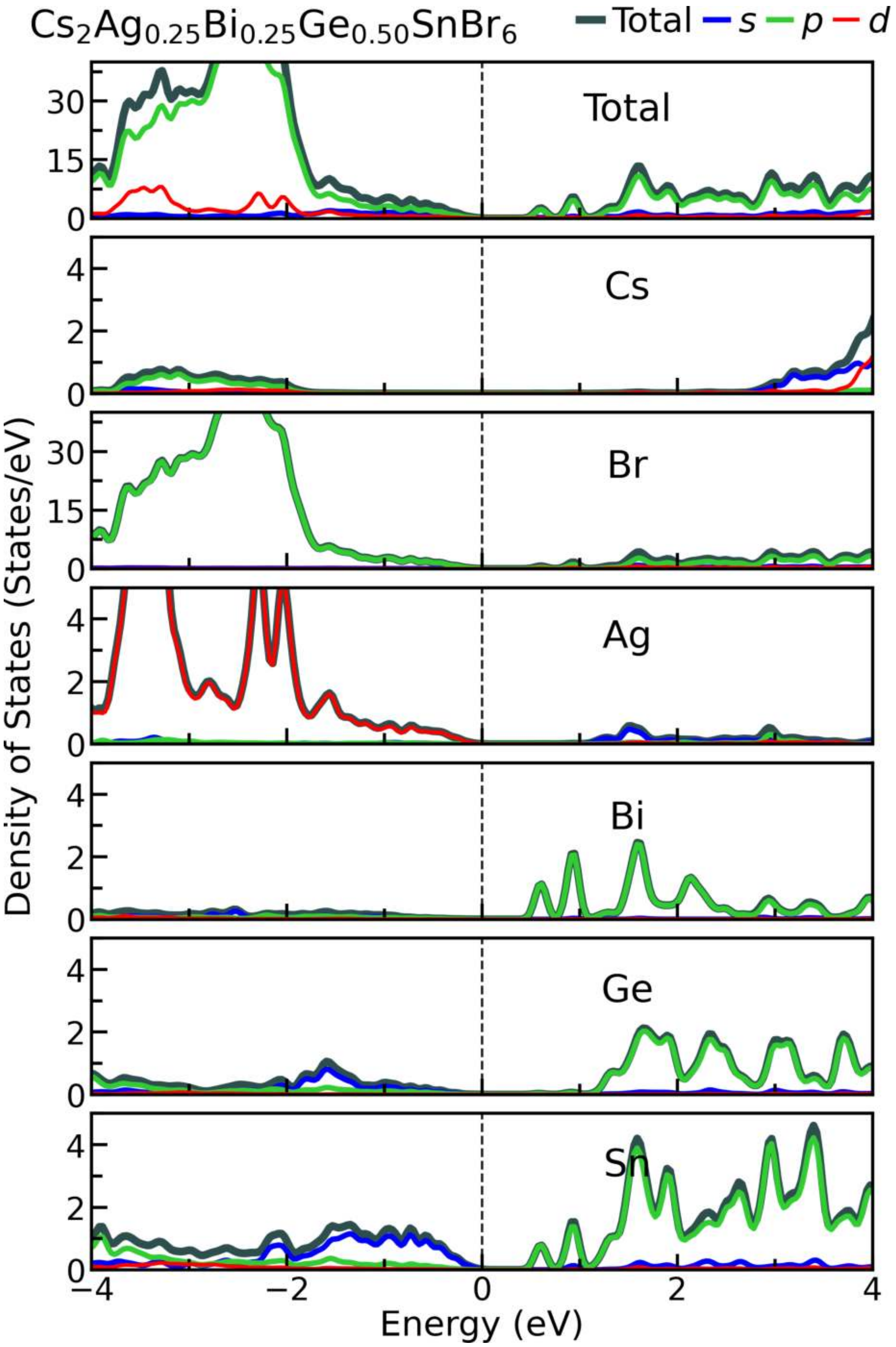

Figure S-135. Density of states of $Cs_2Ag_{0.25}Bi_{0.25}Ge_{0.50}SnBr_6$ at PBE+D3 level, for each atomic species and projected only on *s* and *p* orbitals.

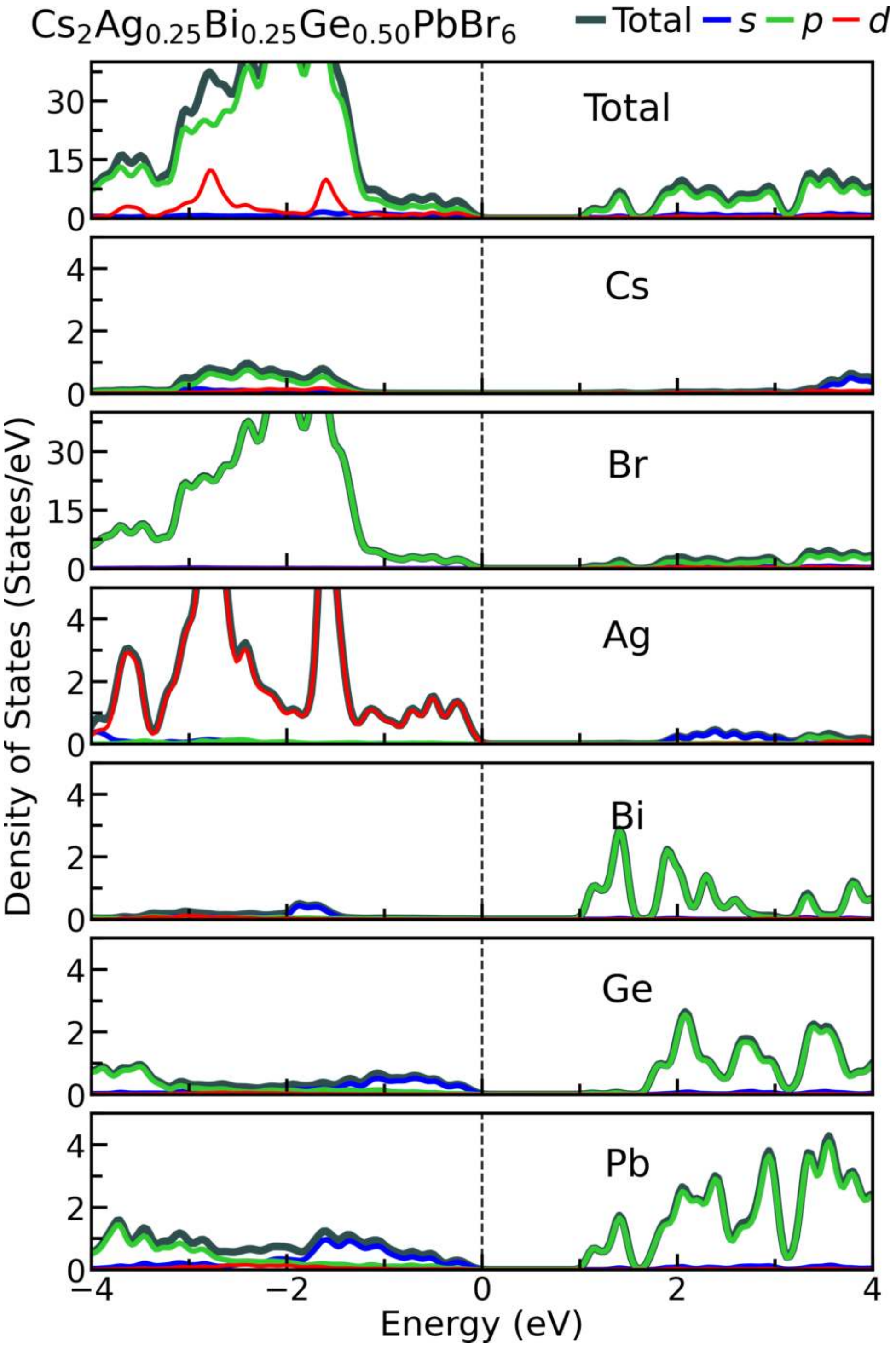

Figure S-136. Density of states of $Cs_2Ag_{0.25}Bi_{0.25}Ge_{0.50}PbBr_6$ at PBE+D3 level, for each atomic species and projected only on *s* and *p* orbitals.

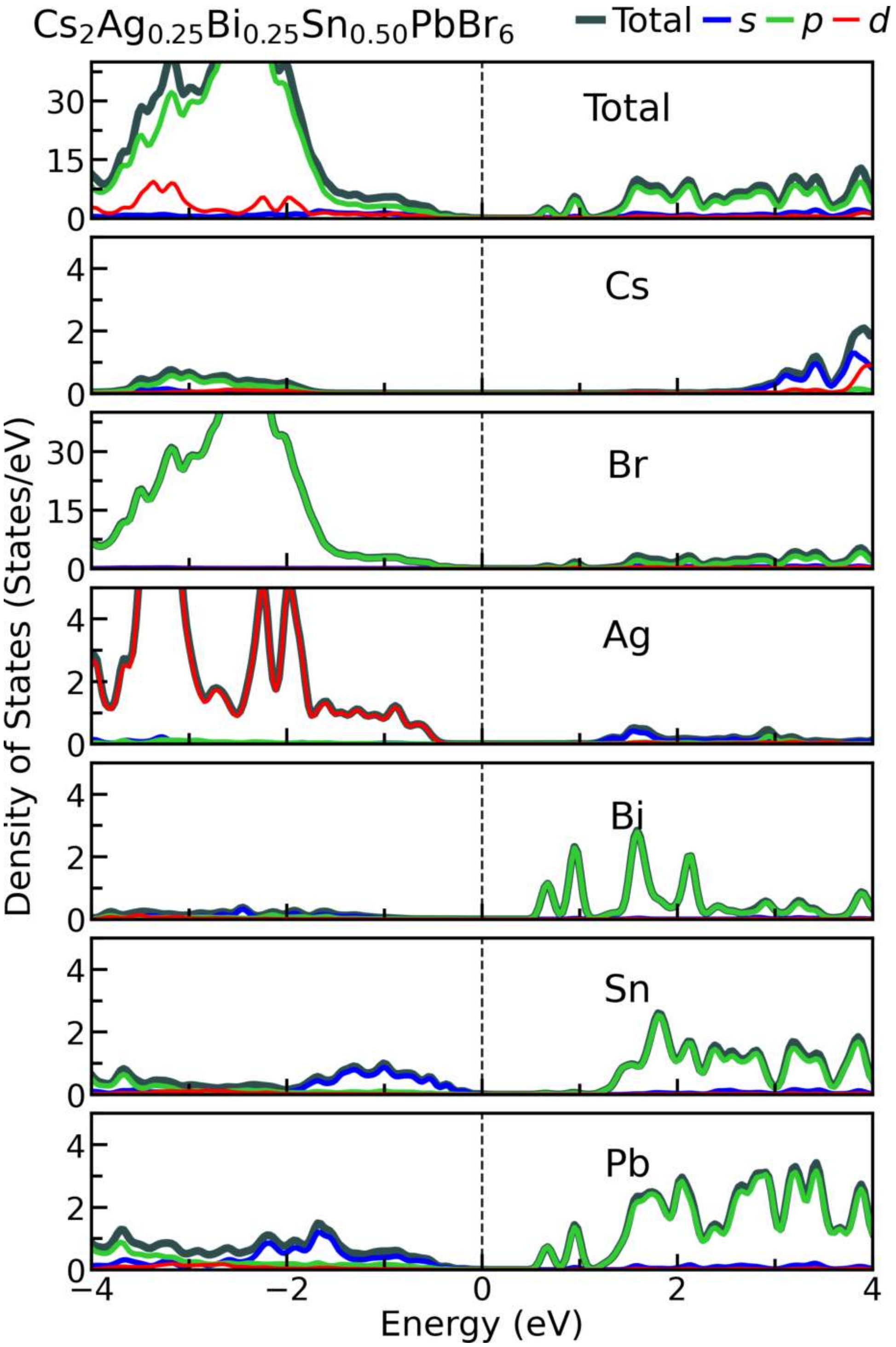

Figure S-137. Density of states of $Cs_2Ag_{0.25}Bi_{0.25}Sn_{0.50}PbBr_6$ at PBE+D3 level, for each atomic species and projected only on *s* and *p* orbitals.

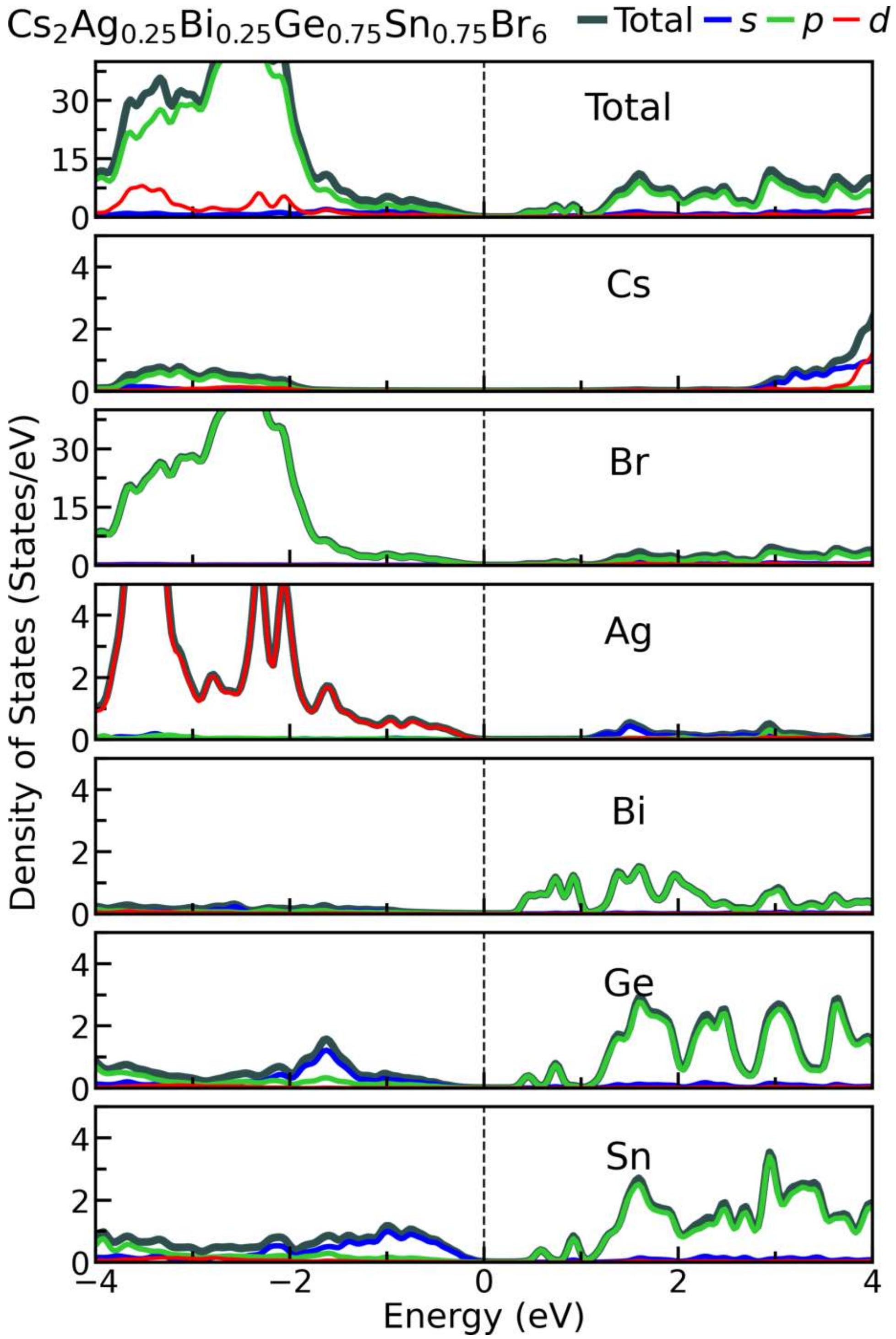

Figure S-138. Density of states of $Cs_2Ag_{0.25}Bi_{0.25}Ge_{0.75}Sn_{0.75}Br_6$ at PBE+D3 level, for each atomic species and projected only on *s* and *p* orbitals.

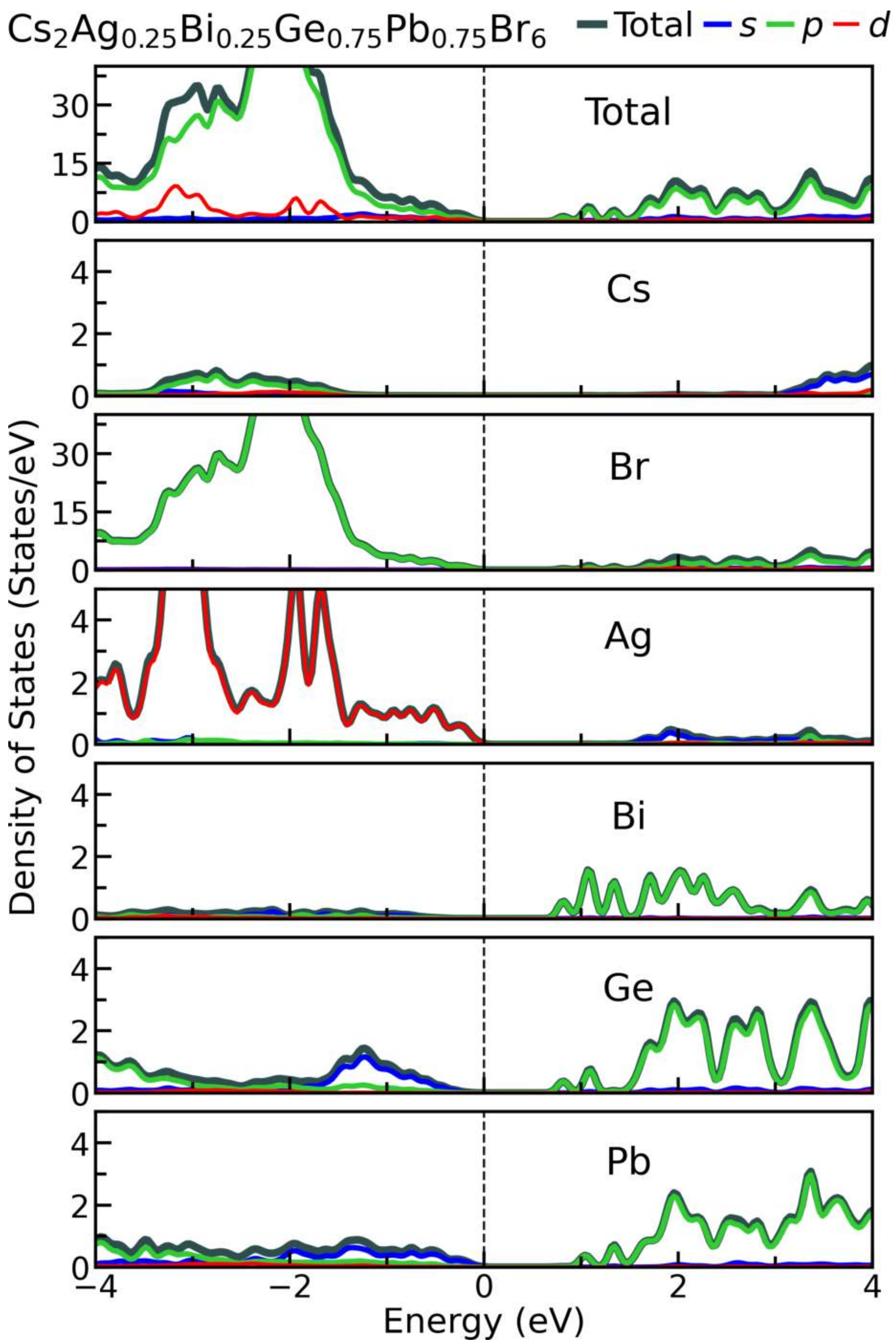

Figure S-139. Density of states of $Cs_2Ag_{0.25}Bi_{0.25}Ge_{0.75}Pb_{0.75}Br_6$ at PBE+D3 level, for each atomic species and projected only on *s* and *p* orbitals.

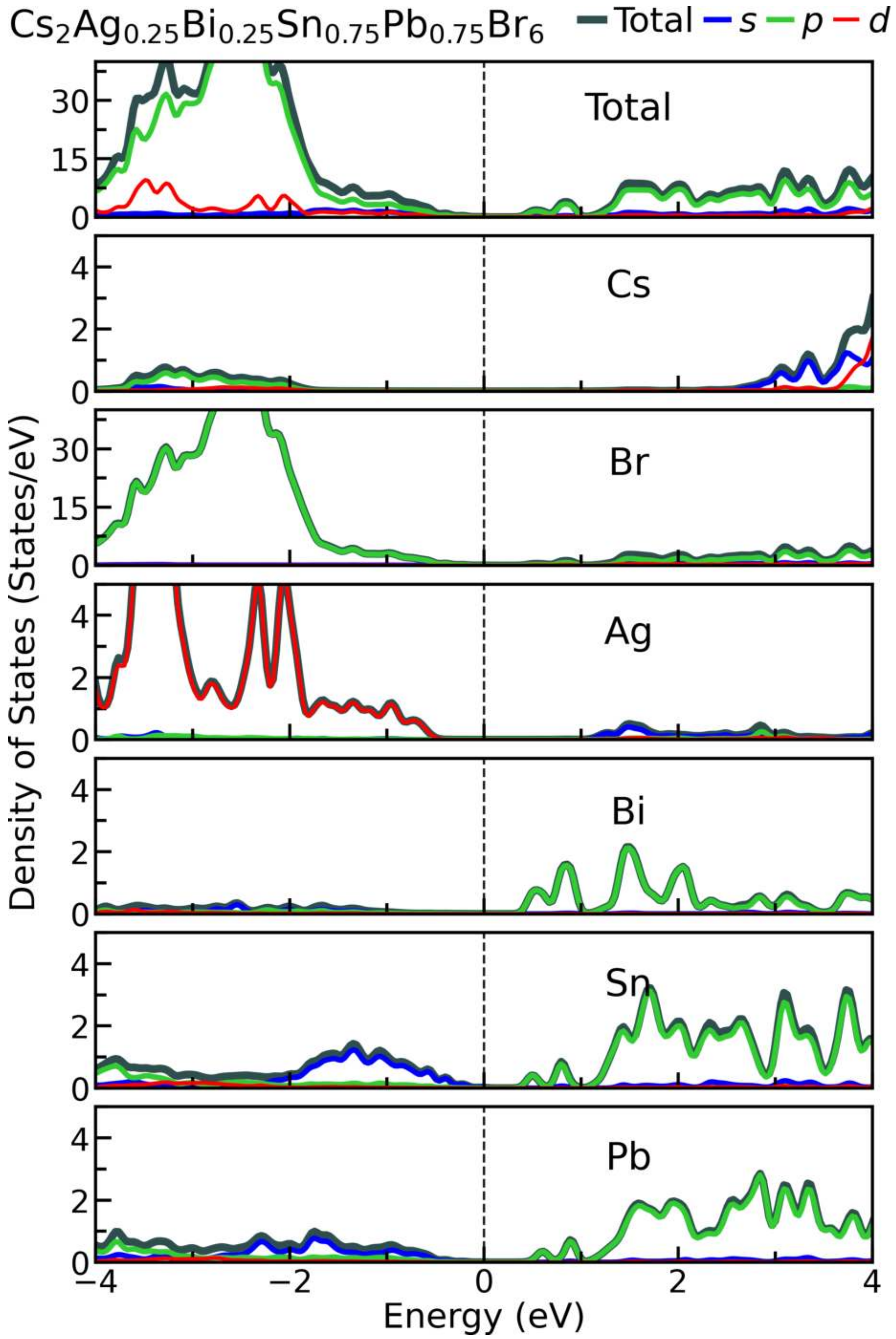

Figure S-140. Density of states of $Cs_2Ag_{0.25}Bi_{0.25}Sn_{0.75}Pb_{0.75}Br_6$ at PBE+D3 level, for each atomic species and projected only on *s* and *p* orbitals.

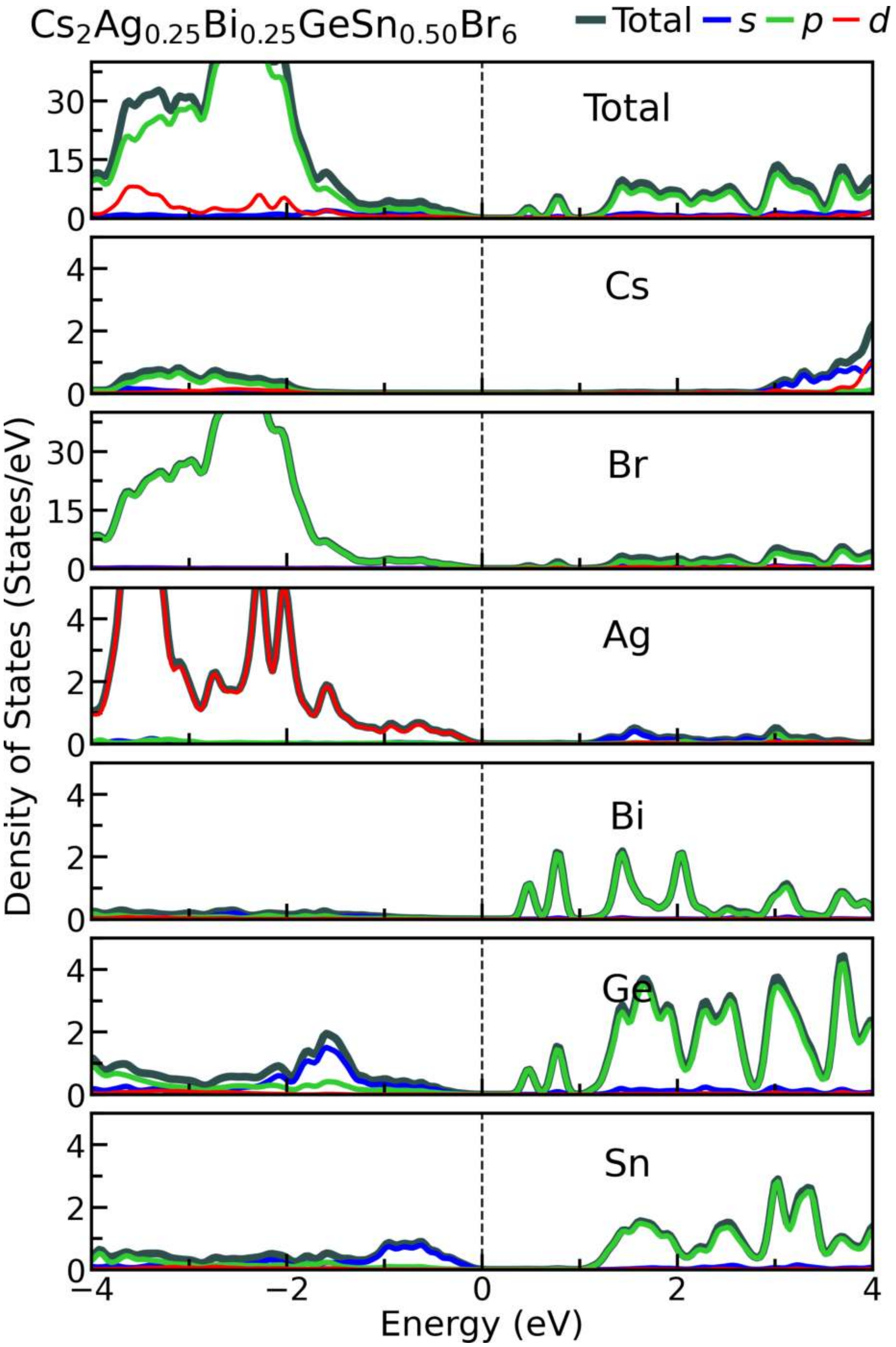

Figure S-141. Density of states of $Cs_2Ag_{0.25}Bi_{0.25}GeSn_{0.50}Br_6$ at PBE+D3 level, for each atomic species and projected only on *s* and *p* orbitals.

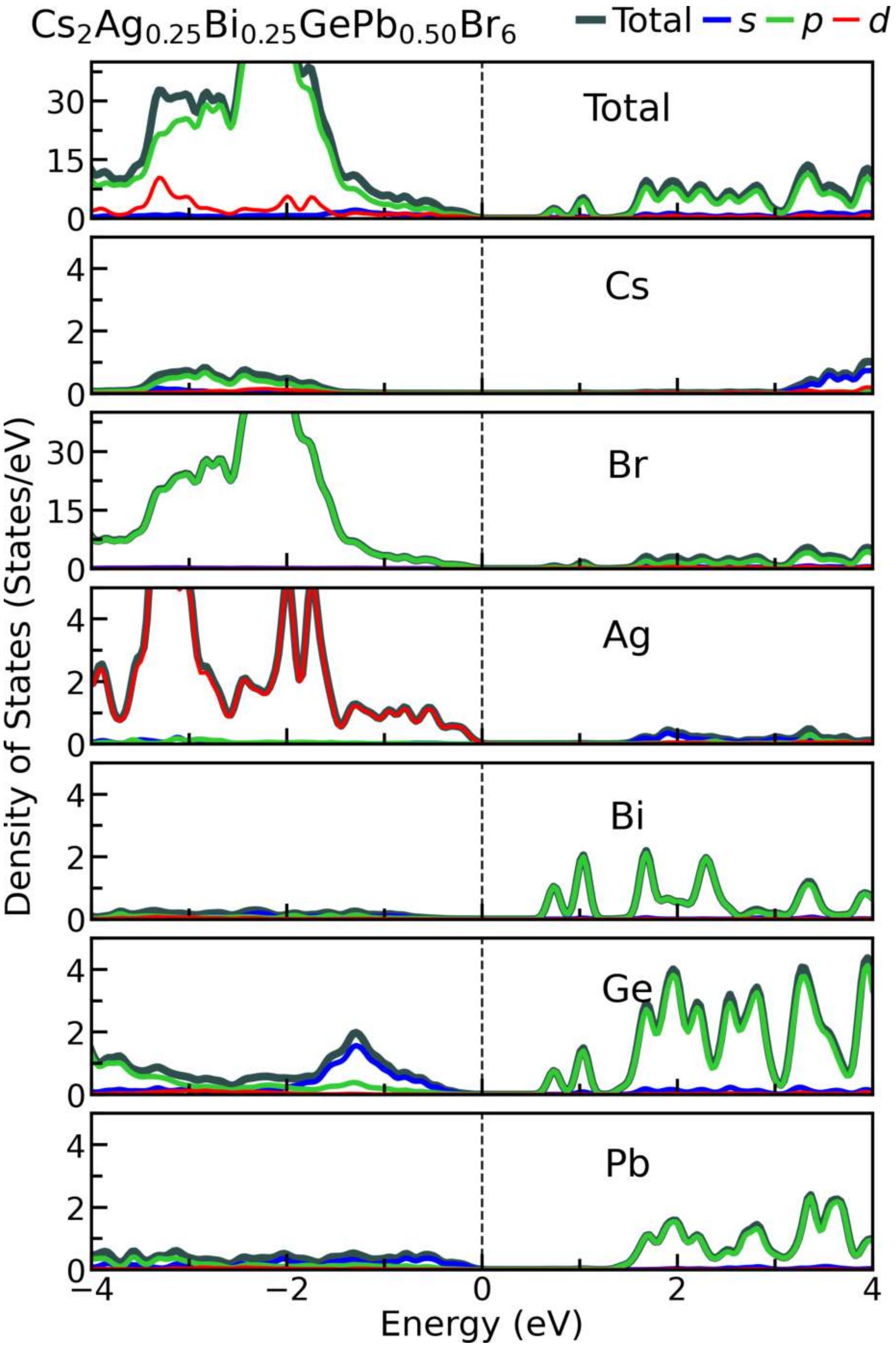

Figure S-142. Density of states of $Cs_2Ag_{0.25}Bi_{0.25}GePb_{0.50}Br_6$ at PBE+D3 level, for each atomic species and projected only on *s* and *p* orbitals.

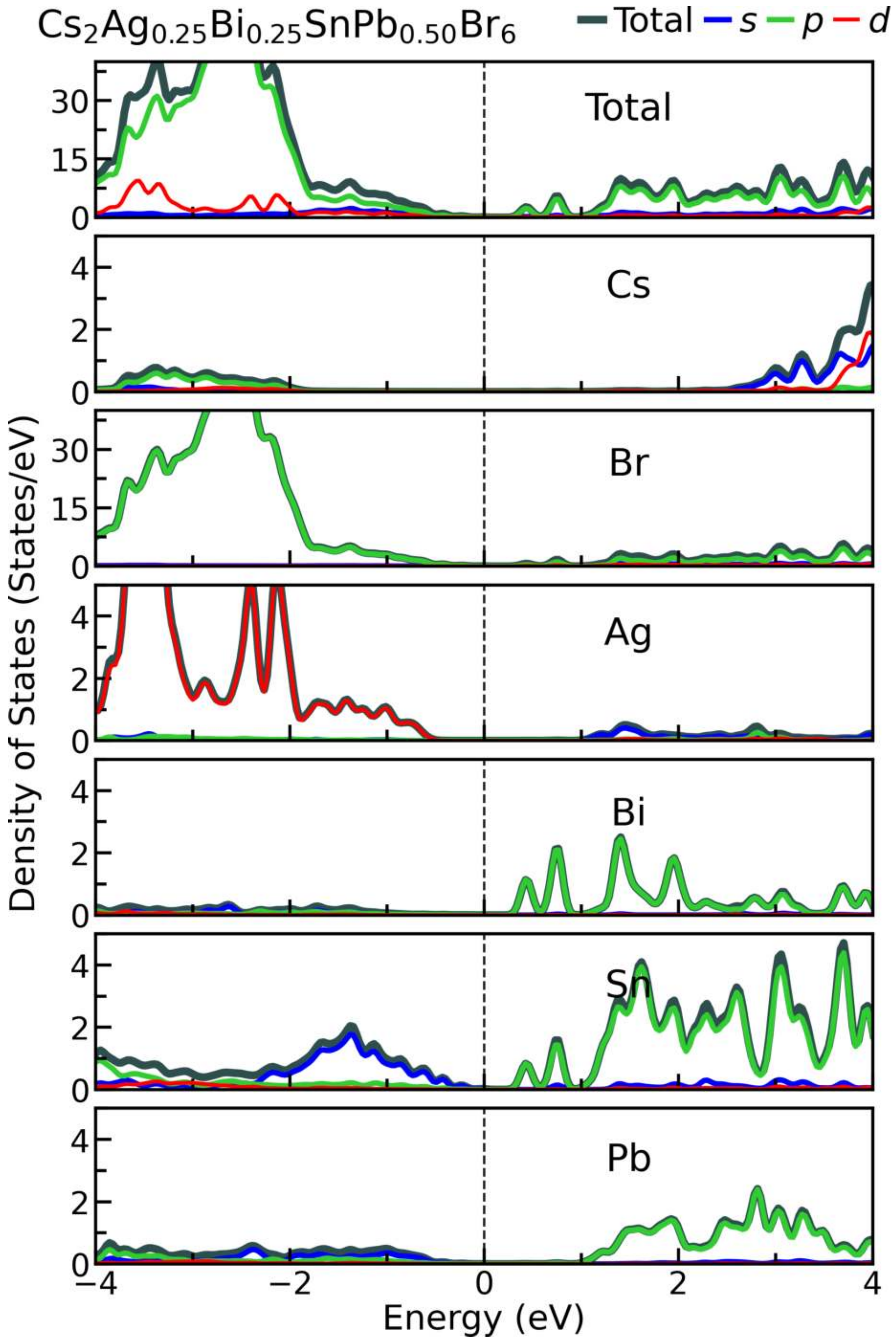

Figure S-143. Density of states of $Cs_2Ag_{0.25}Bi_{0.25}SnPb_{0.50}Br_6$ at PBE+D3 level, for each atomic species and projected only on *s* and *p* orbitals.

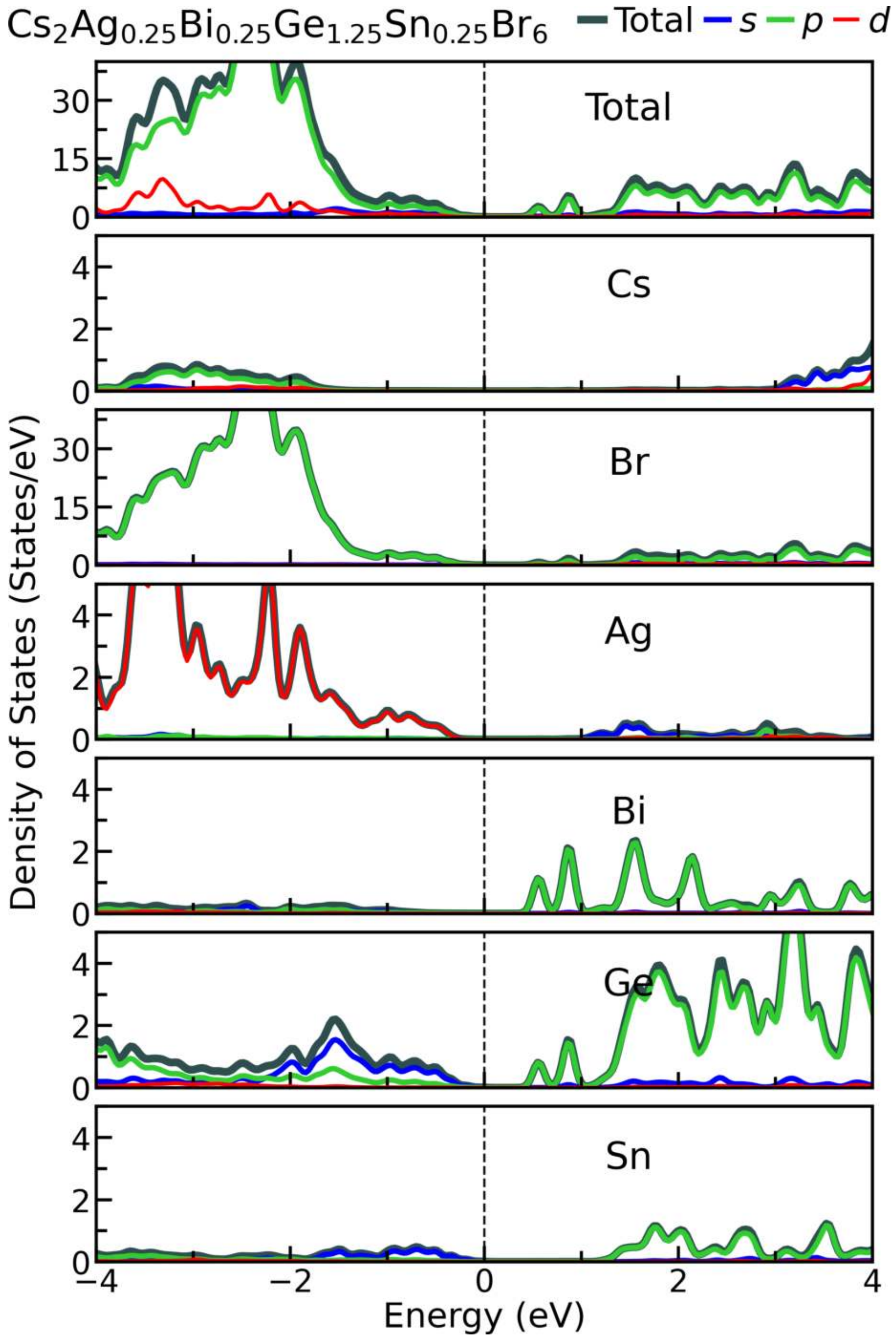

Figure S-144. Density of states of $Cs_2Ag_{0.25}Bi_{0.25}Ge_{1.25}Sn_{0.25}Br_6$ at PBE+D3 level, for each atomic species and projected only on *s* and *p* orbitals.

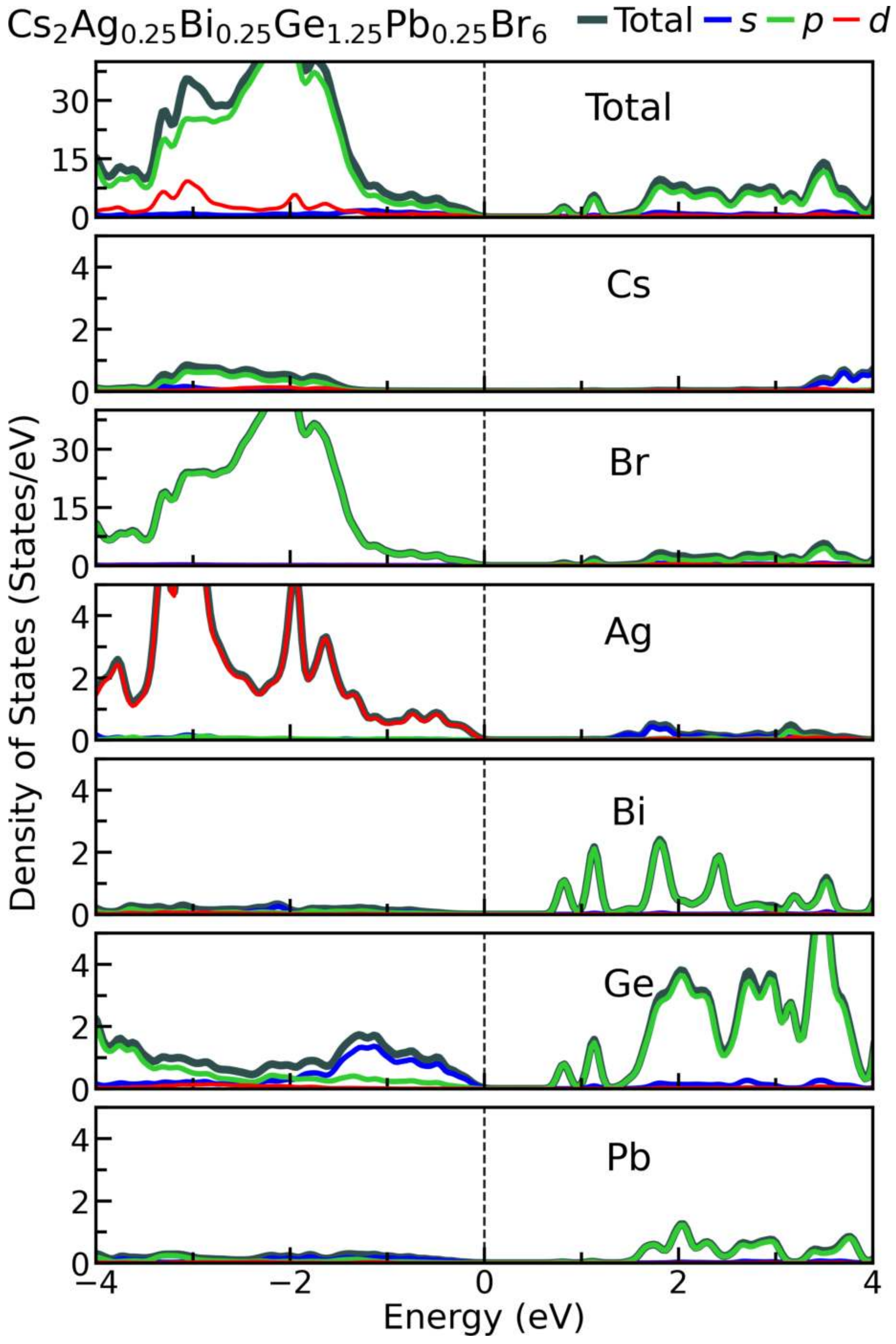

Figure S-145. Density of states of $Cs_2Ag_{0.25}Bi_{0.25}Ge_{1.25}Pb_{0.25}Br_6$ at PBE+D3 level, for each atomic species and projected only on *s* and *p* orbitals.

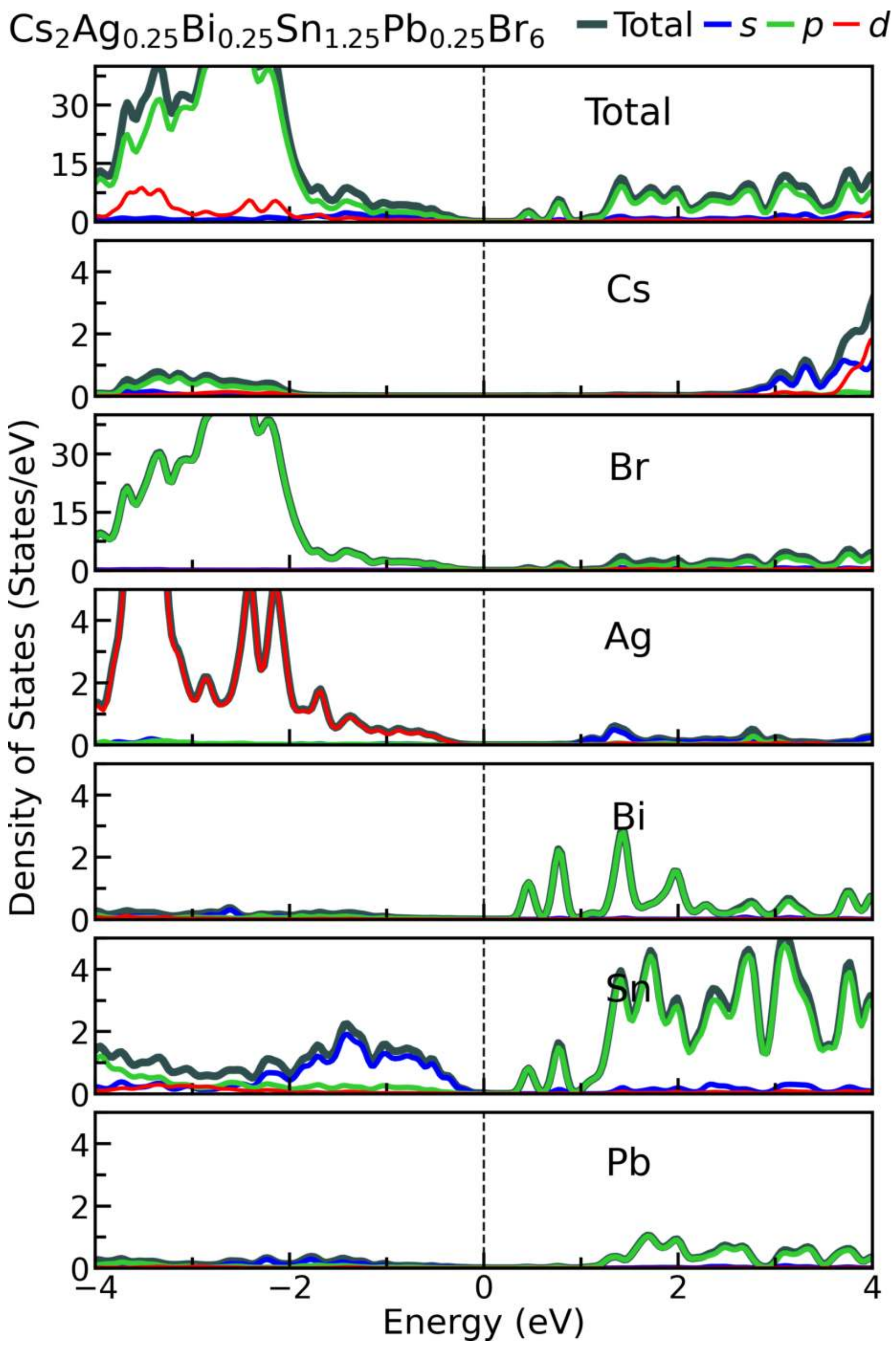

Figure S-146. Density of states of $Cs_2Ag_{0.25}Bi_{0.25}Sn_{1.25}Pb_{0.25}Br_6$ at PBE+D3 level, for each atomic species and projected only on *s* and *p* orbitals.

S-8.7 Absorption Coefficient and Optical Band Gap

S-8.7.1 Pristine Compounds

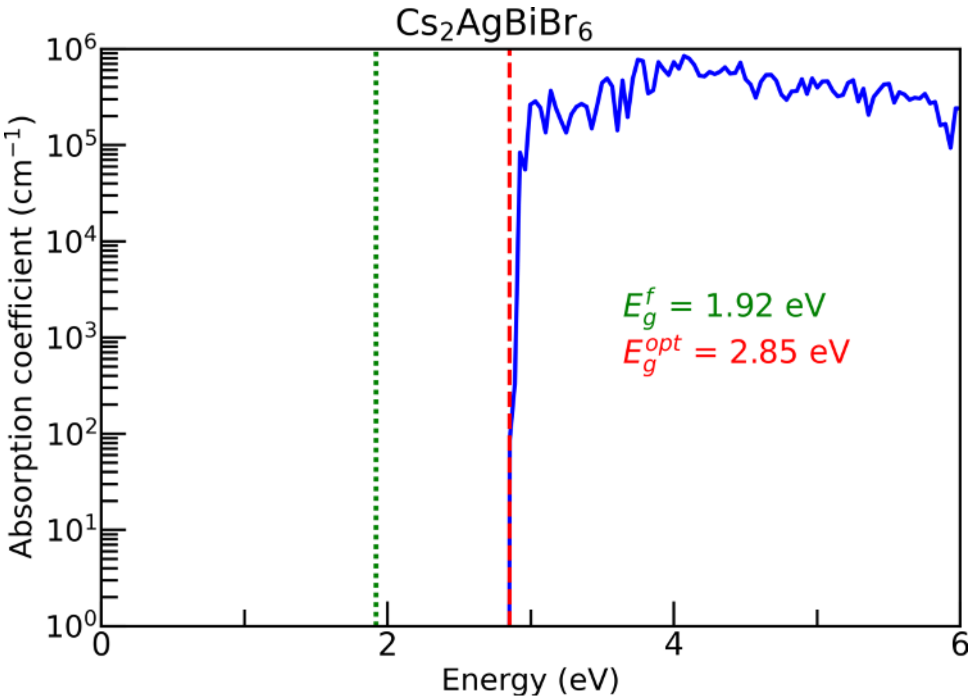

Figure S-147. Absorption coefficient at PBE+D3+χ level of $Cs_2AgBiBr_6$. In green-dotted and red-dashed lines are indicated the optical ($E_g^{optical+\chi}$) and fundamental band gaps (E_g^f at PBE+D3+SOC+χ^{HSE06} level), here placed as insets simply as E_g^{opt} and E_g^f, respectively.

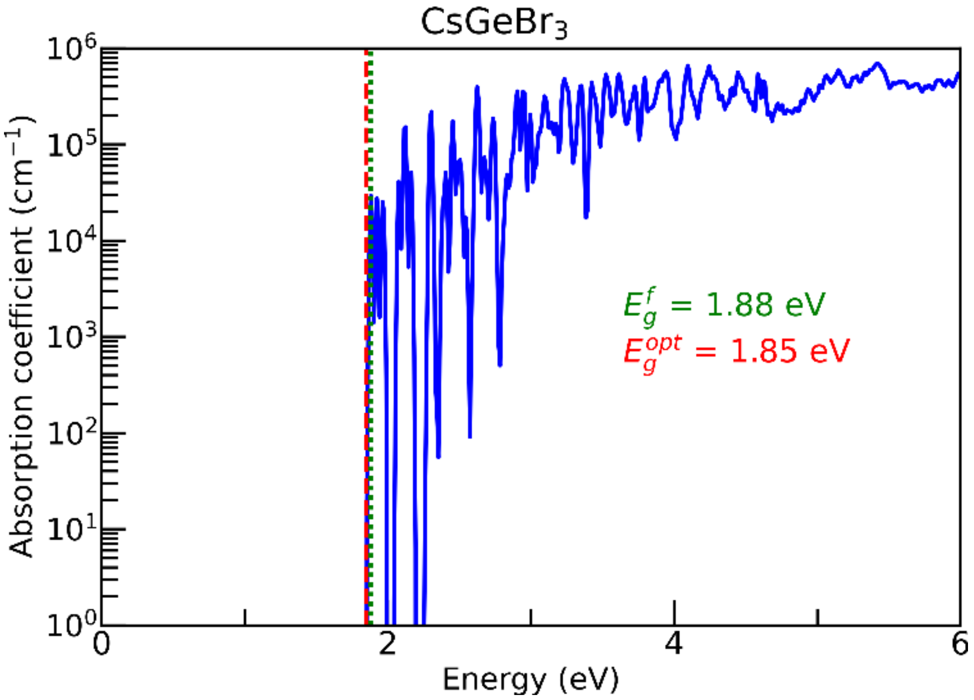

Figure S-148. Absorption coefficient at PBE+D3 + χ level of $CsGeBr_3$. In green-dotted and red-dashed lines are indicated the optical ($E_g^{optical+\chi}$) and fundamental band gaps (E_g^f at PBE+D3+SOC+χ^{HSE06} level), here placed as insets simply as E_g^{opt} and E_g^f, respectively.

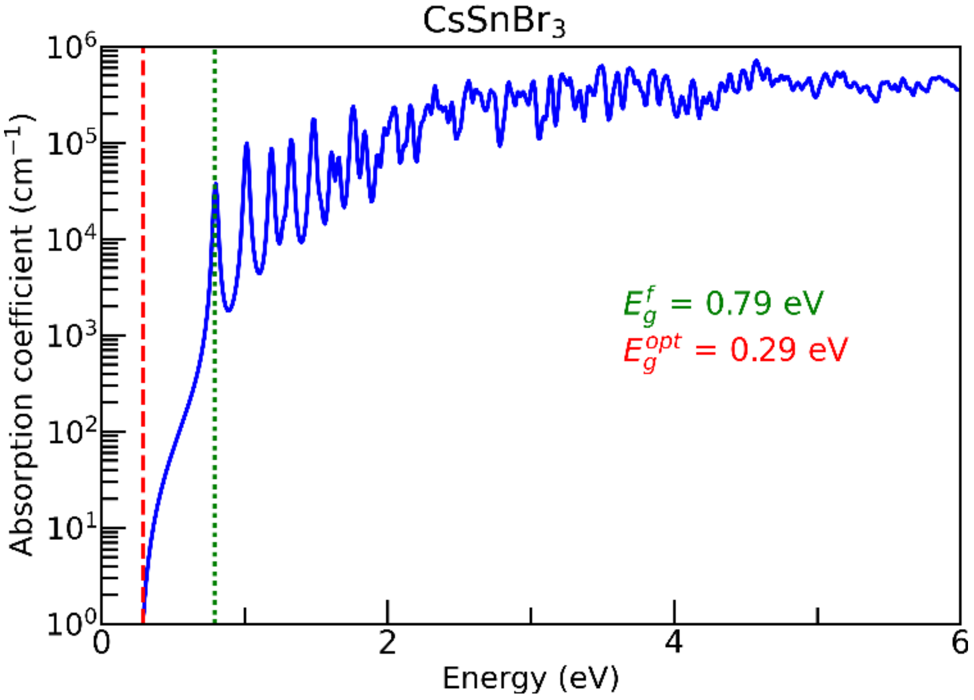

Figure S-149. Absorption coefficient at PBE+D3 + χ level of $CsSnBr_3$. In green-dotted and red-dashed lines are indicated the optical ($E_g^{optical+\chi}$) and fundamental band gaps (E_g^f at PBE+D3+SOC+χ^{HSE06} level), here placed as insets simply as E_g^{opt} and E_g^f, respectively.

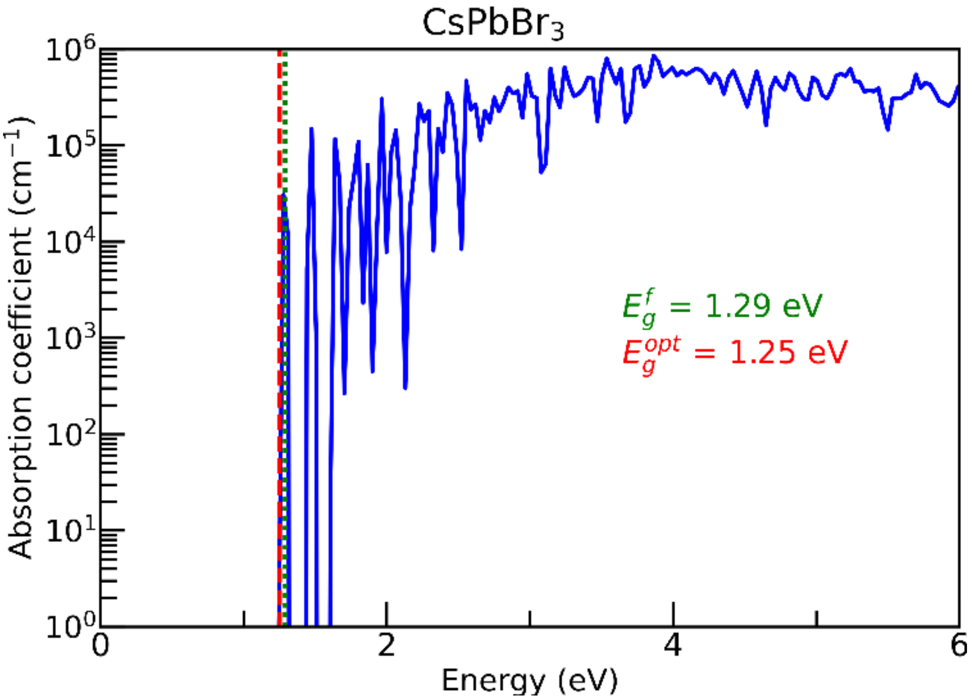

Figure S-150. Absorption coefficient at PBE+D3 + χ level of $CsPbBr_3$. In green-dotted and red-dashed lines are indicated the optical ($E_g^{optical+\chi}$) and fundamental band gaps (E_g^f at PBE+D3+SOC+χ^{HSE06} level), here placed as insets simply as E_g^{opt} and E_g^f, respectively.

S-8.7.2 Mixtures with One Divalent Metal

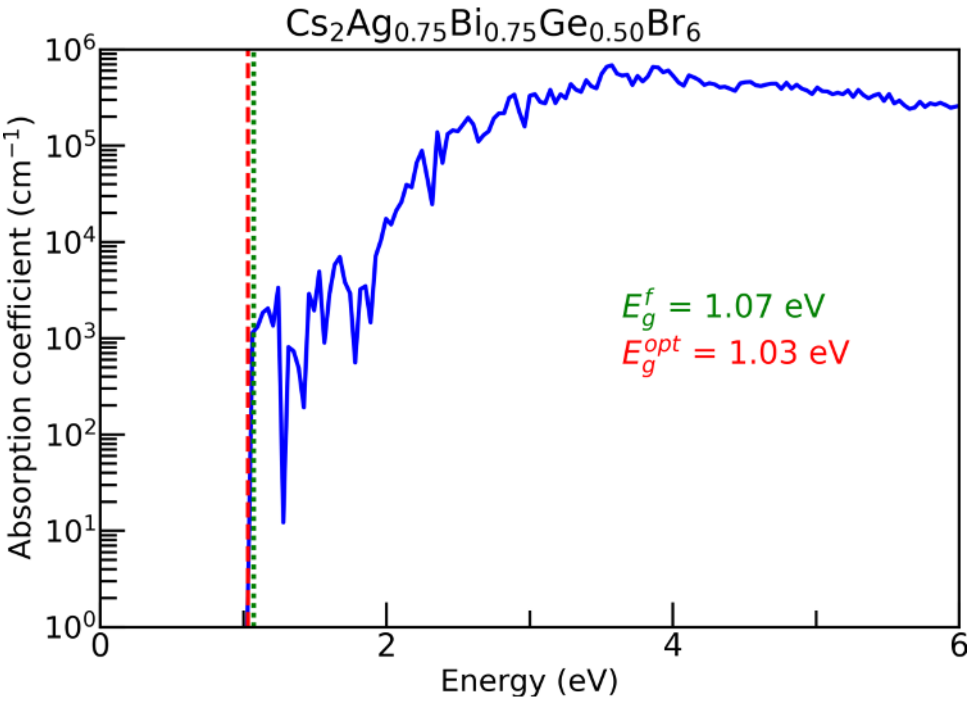

Figure S-151. Absorption coefficient at PBE+D3 + χ level of $Cs_2Ag_{0.75}Bi_{0.75}Ge_{0.50}Br_6$. In green-dotted and red-dashed lines are indicated the optical ($E_g^{optical+\chi}$) and fundamental band gaps (E_g^f at PBE+D3+SOC+χ^{HSE06} level), here placed as insets simply as E_g^{opt} and E_g^f, respectively.

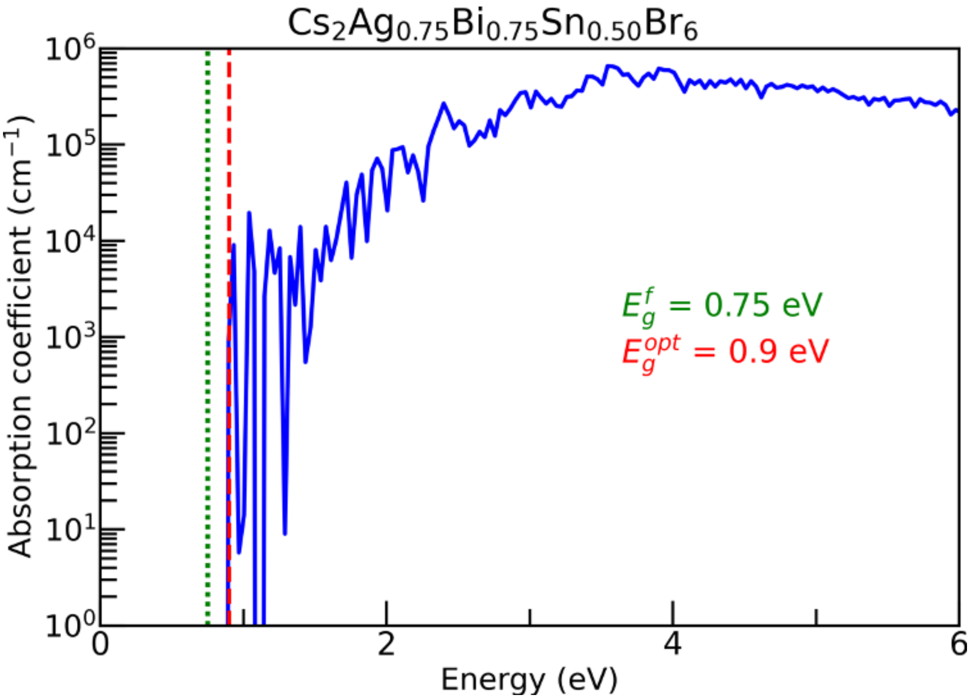

Figure S-152. Absorption coefficient at PBE+D3 + χ level of $Cs_2Ag_{0.75}Bi_{0.75}Sn_{0.50}Br_6$. In green-dotted and red-dashed lines are indicated the optical ($E_g^{optical+\chi}$) and fundamental band gaps (E_g^f at PBE+D3+SOC+χ^{HSE06} level), here placed as insets simply as E_g^{opt} and E_g^f, respectively.

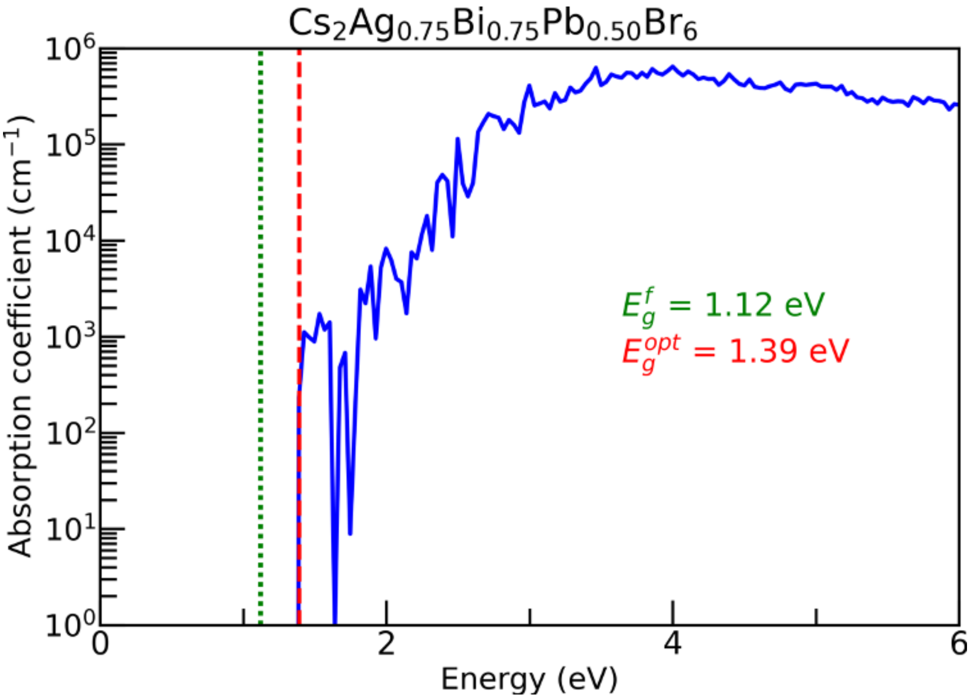

Figure S-153. Absorption coefficient at PBE+D3 + χ level of $Cs_2Ag_{0.75}Bi_{0.75}Pb_{0.50}Br_6$. In green-dotted and red-dashed lines are indicated the optical ($E_g^{optical+\chi}$) and fundamental band gaps (E_g^f at PBE+D3+SOC+χ^{HSE06} level), here placed as insets simply as E_g^{opt} and E_g^f, respectively.

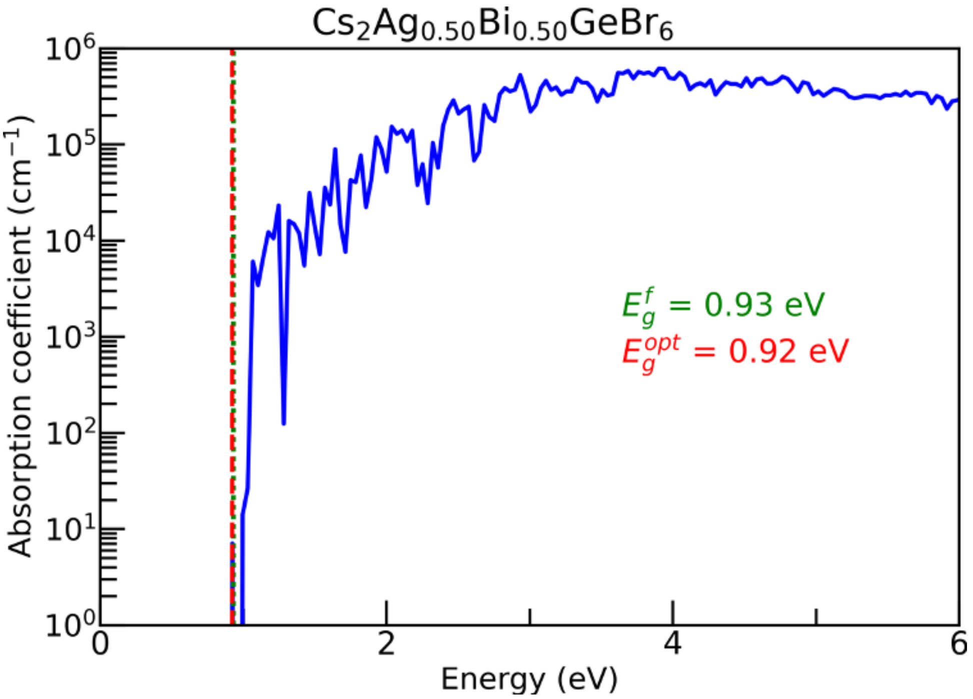

Figure S-154. Absorption coefficient at PBE+D3 + χ level of $Cs_2Ag_{0.50}Bi_{0.50}GeBr_6$. In green-dotted and red-dashed lines are indicated the optical ($E_g^{optical+\chi}$) and fundamental band gaps (E_g^f at PBE+D3+SOC+χ^{HSE06} level), here placed as insets simply as E_g^{opt} and E_g^f, respectively.

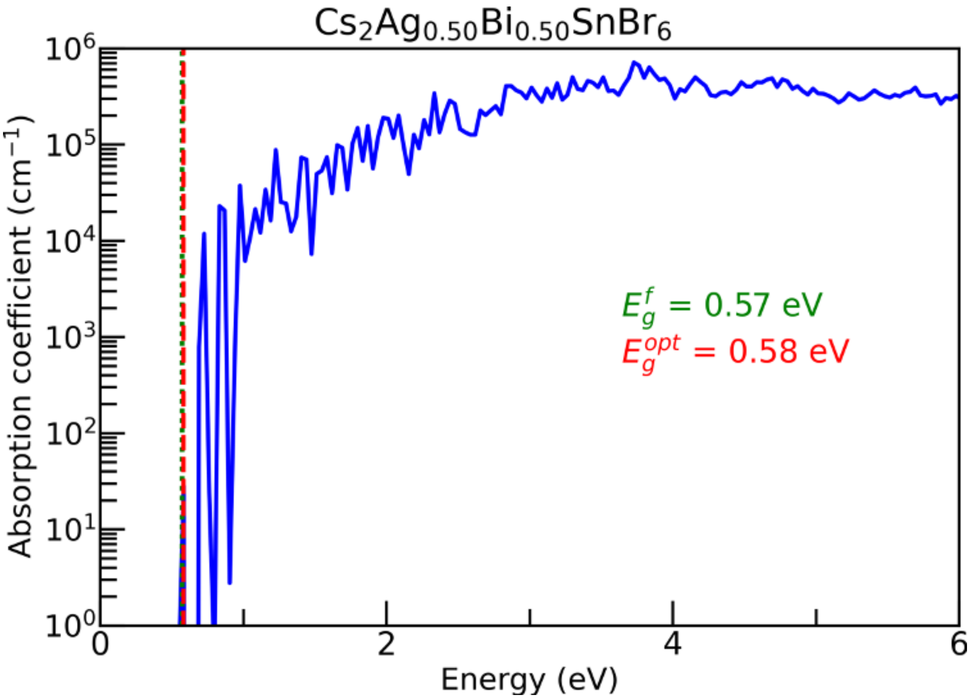

Figure S-155. Absorption coefficient at PBE+D3 + χ level of $Cs_2Ag_{0.50}Bi_{0.50}SnBr_6$. In green-dotted and red-dashed lines are indicated the optical ($E_g^{optical+\chi}$) and fundamental band gaps (E_g^f at PBE+D3+SOC+χ^{HSE06} level), here placed as insets simply as E_g^{opt} and E_g^f, respectively.

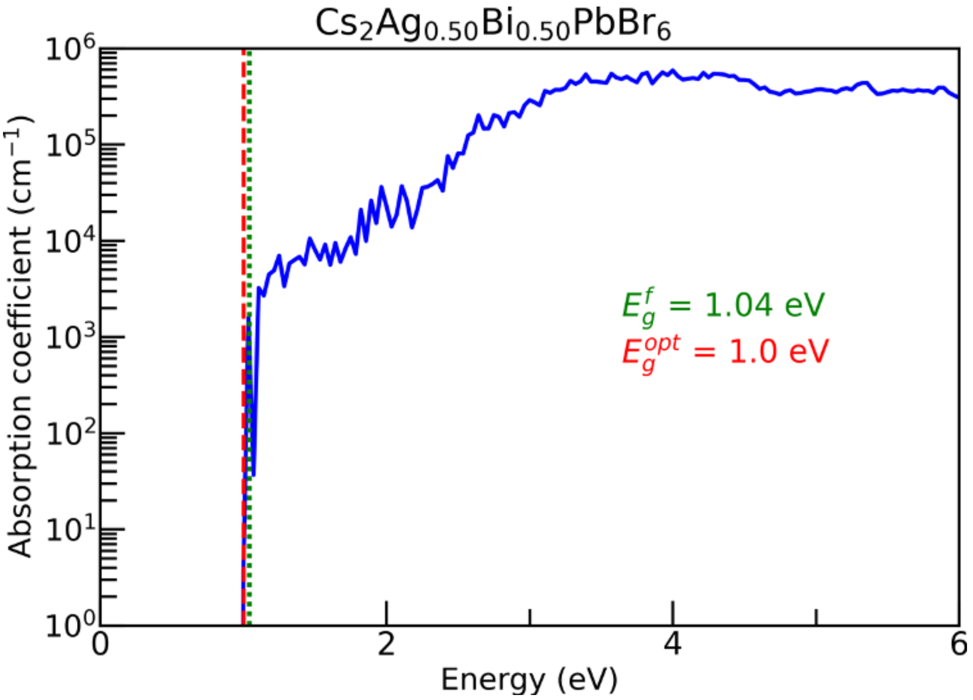

Figure S-156. Absorption coefficient at PBE+D3 + χ level of $Cs_2Ag_{0.50}Bi_{0.50}PbBr_6$. In green-dotted and red-dashed lines are indicated the optical ($E_g^{optical+\chi}$) and fundamental band gaps (E_g^f at PBE+D3+SOC+χ^{HSE06} level), here placed as insets simply as E_g^{opt} and E_g^f, respectively.

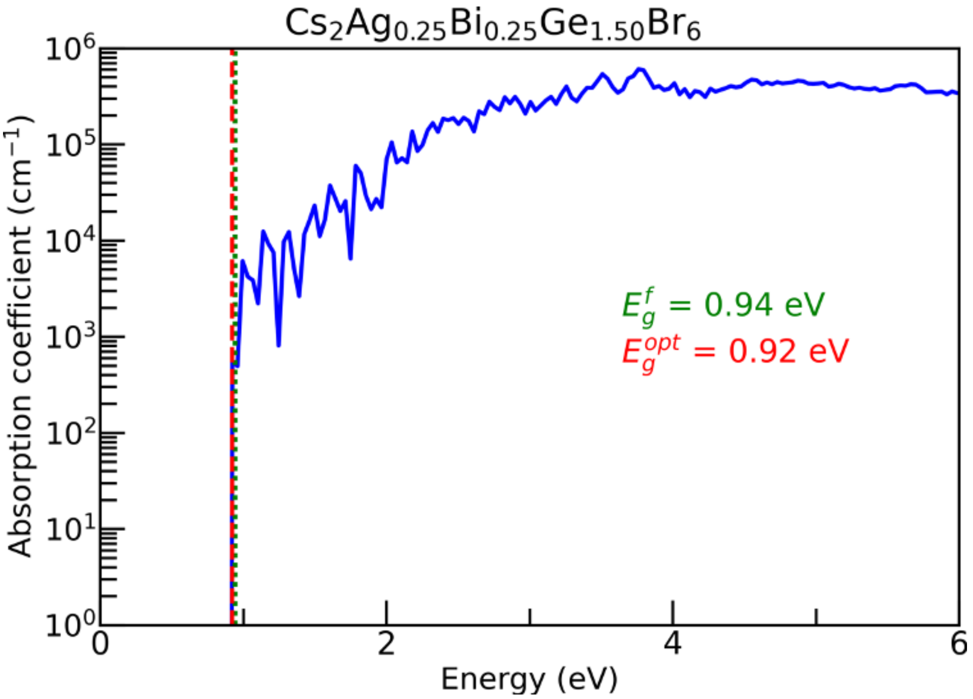

Figure S-157. Absorption coefficient at PBE+D3 + χ level of $Cs_2Ag_{0.25}Bi_{0.25}Ge_{1.50}Br_6$. In green-dotted and red-dashed lines are indicated the optical ($E_g^{optical+\chi}$) and fundamental band gaps (E_g^f at PBE+D3+SOC+χ^{HSE06} level), here placed as insets simply as E_g^{opt} and E_g^f, respectively.

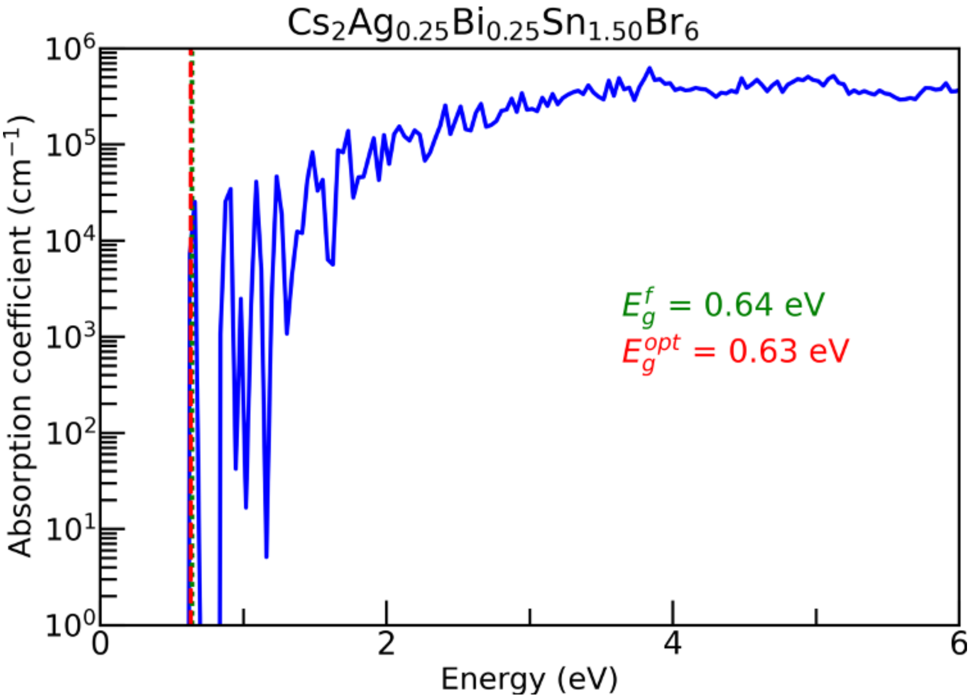

Figure S-158. Absorption coefficient at PBE+D3 + χ level of $Cs_2Ag_{0.25}Bi_{0.25}Sn_{1.50}Br_6$. In green-dotted and red-dashed lines are indicated the optical ($E_g^{optical+\chi}$) and fundamental band gaps (E_g^f at PBE+D3+SOC+χ^{HSE06} level), here placed as insets simply as E_g^{opt} and E_g^f, respectively.

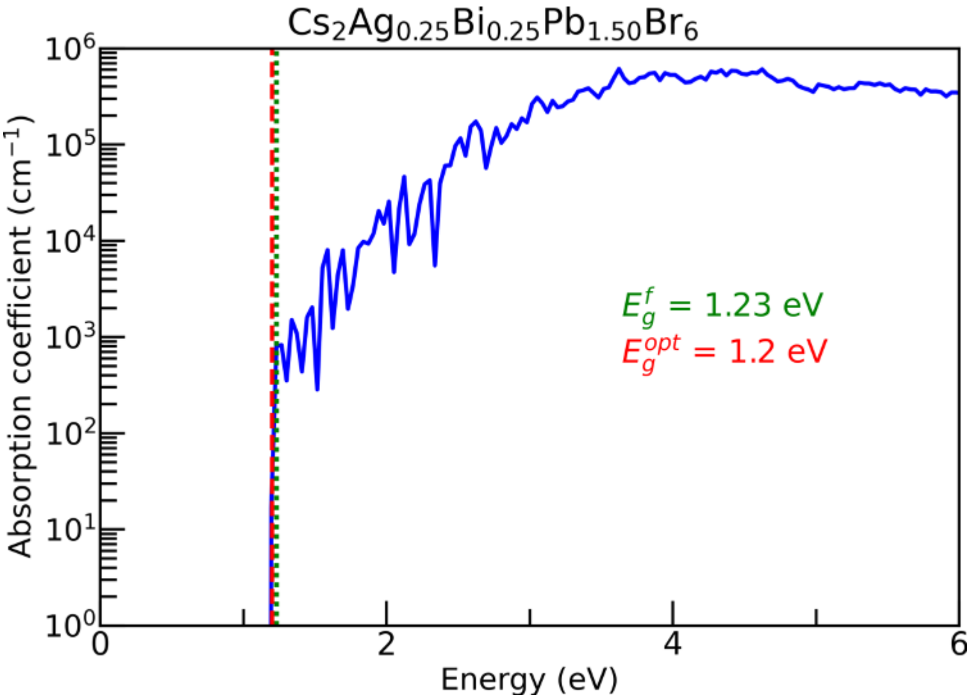

Figure S-159. Absorption coefficient at PBE+D3 + χ level of $Cs_2Ag_{0.25}Bi_{0.25}Pb_{1.50}Br_6$. In green-dotted and red-dashed lines are indicated the optical ($E_g^{optical+\chi}$) and fundamental band gaps (E_g^f at PBE+D3+SOC+χ^{HSE06} level), here placed as insets simply as E_g^{opt} and E_g^f, respectively.

S-8.7.3 Mixtures with Two Divalent Metals

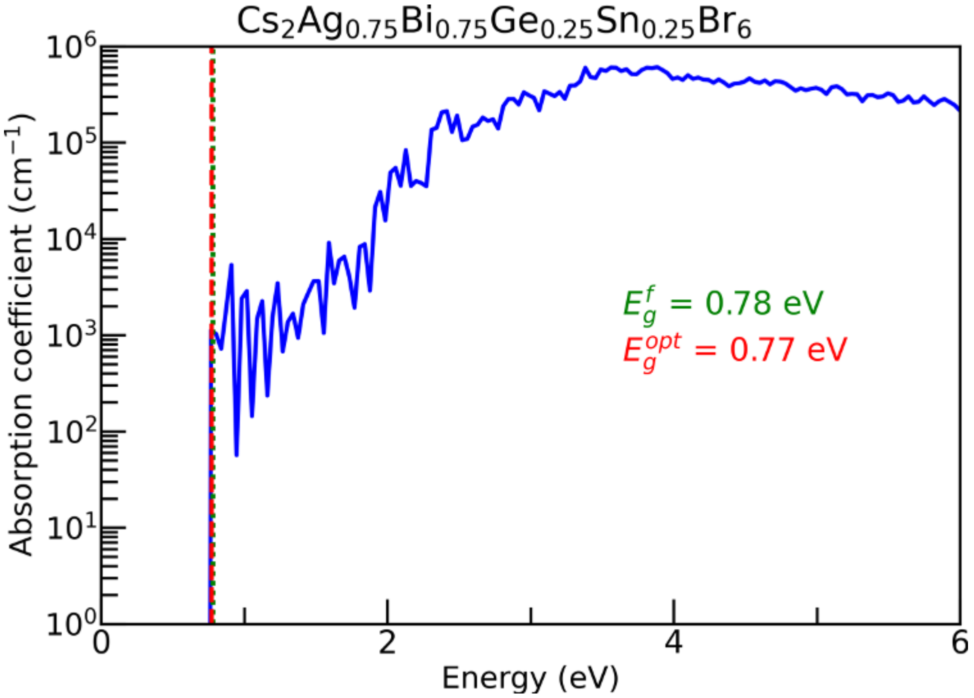

Figure S-160. Absorption coefficient at PBE+D3 + χ level of $Cs_2Ag_{0.75}Bi_{0.75}Ge_{0.25}Sn_{0.25}Br_6$. In green-dotted and red-dashed lines are indicated the optical ($E_g^{optical+\chi}$) and fundamental band gaps (E_g^f at PBE+D3+SOC+χ^{HSE06} level), here placed as insets simply as E_g^{opt} and E_g^f, respectively.

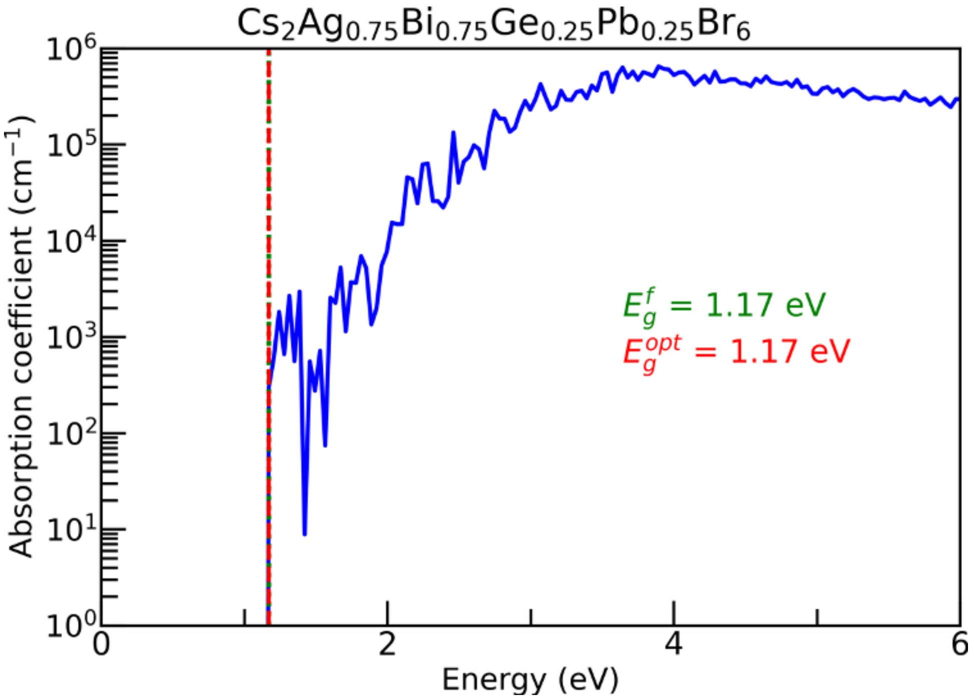

Figure S-161. Absorption coefficient at PBE+D3 + χ level of $Cs_2Ag_{0.75}Bi_{0.75}Ge_{0.25}Pb_{0.25}Br_6$. In green-dotted and red-dashed lines are indicated the optical ($E_g^{optical+\chi}$) and fundamental band gaps (E_g^f at PBE+D3+SOC+χ^{HSE06} level), here placed as insets simply as E_g^{opt} and E_g^f, respectively.

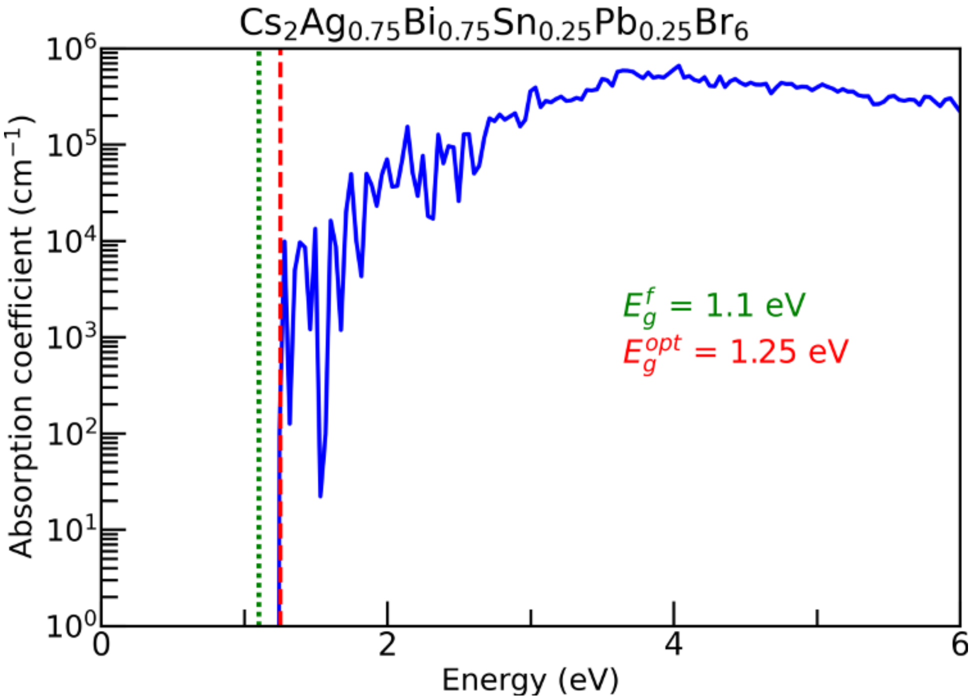

Figure S-162. Absorption coefficient at PBE+D3 + χ level of $Cs_2Ag_{0.75}Bi_{0.75}Sn_{0.25}Pb_{0.25}Br_6$. In green-dotted and red-dashed lines are indicated the optical ($E_g^{optical+\chi}$) and fundamental band gaps (E_g^f at PBE+D3+SOC+χ^{HSE06} level), here placed as insets simply as E_g^{opt} and E_g^f, respectively.

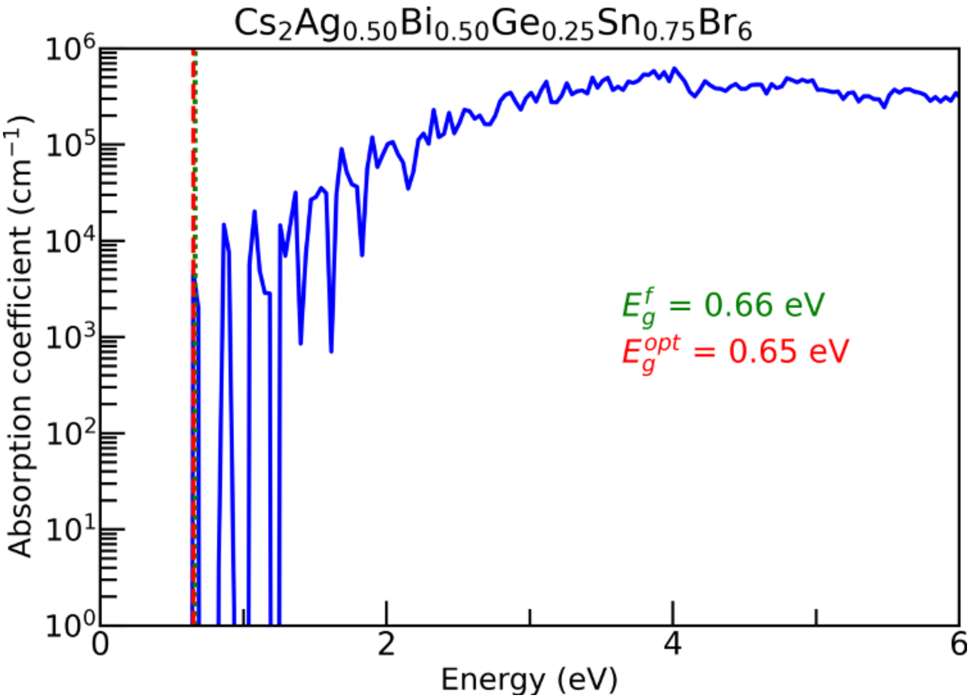

Figure S-163. Absorption coefficient at PBE+D3 + χ level of $Cs_2Ag_{0.50}Bi_{0.50}Ge_{0.25}Sn_{0.75}Br_6$. In green-dotted and red-dashed lines are indicated the optical ($E_g^{optical+\chi}$) and fundamental band gaps (E_g^f at PBE+D3+SOC+χ^{HSE06} level), here placed as insets simply as E_g^{opt} and E_g^f, respectively.

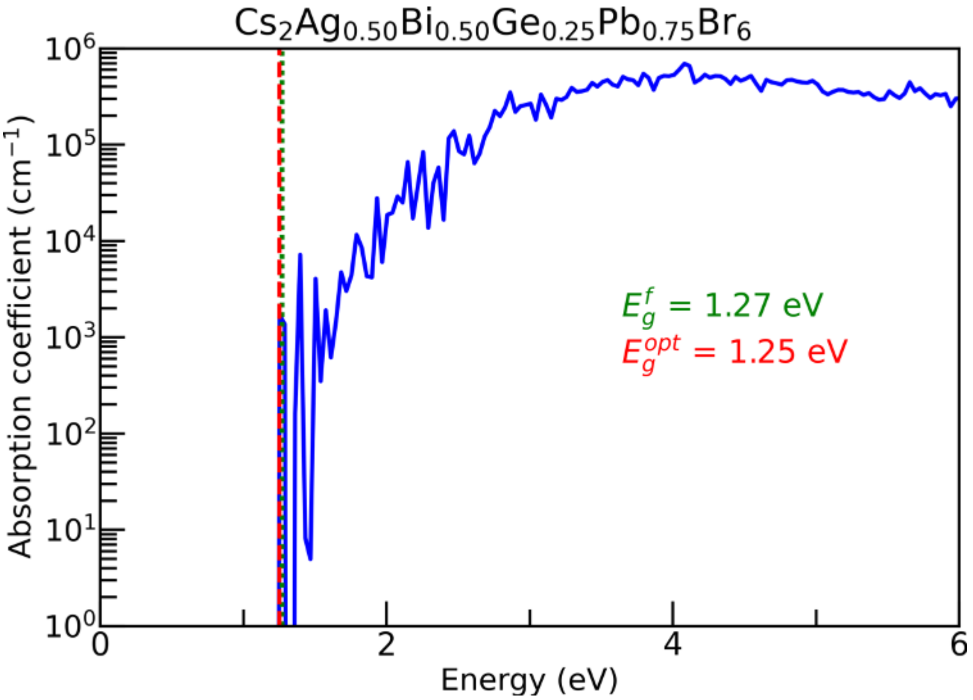

Figure S-164. Absorption coefficient at PBE+D3 + χ level of $Cs_2Ag_{0.50}Bi_{0.50}Ge_{0.25}Pb_{0.75}Br_6$. In green-dotted and red-dashed lines are indicated the optical ($E_g^{optical+\chi}$) and fundamental band gaps (E_g^f at PBE+D3+SOC+χ^{HSE06} level), here placed as insets simply as E_g^{opt} and E_g^f, respectively.

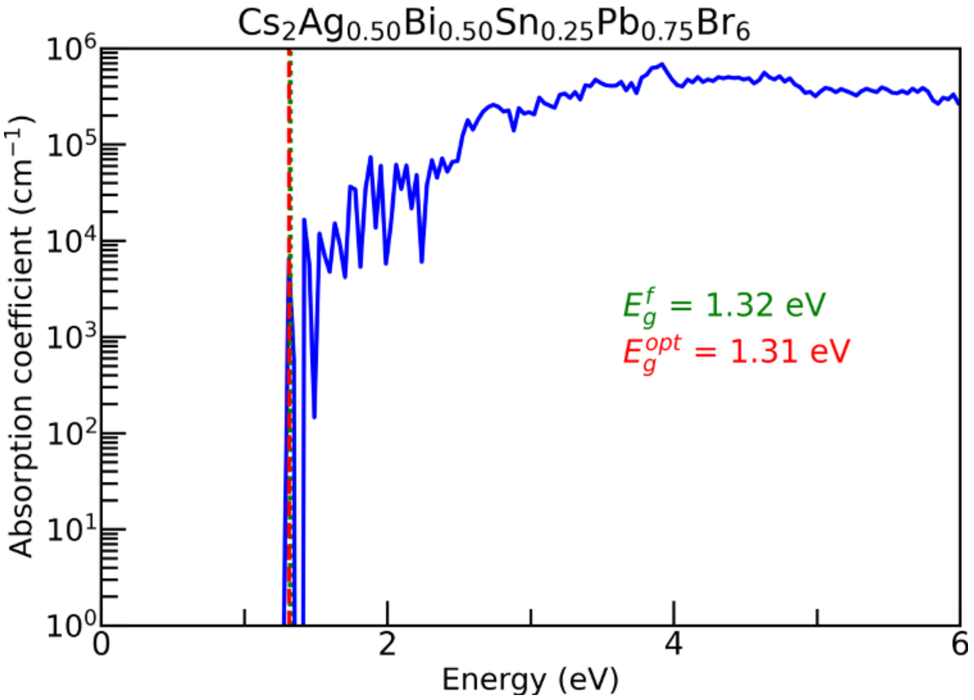

Figure S-165. Absorption coefficient at PBE+D3 + χ level of $Cs_2Ag_{0.50}Bi_{0.50}Sn_{0.25}Pb_{0.75}Br_6$. In green-dotted and red-dashed lines are indicated the optical ($E_g^{optical+\chi}$) and fundamental band gaps (E_g^f at PBE+D3+SOC+χ^{HSE06} level), here placed as insets simply as E_g^{opt} and E_g^f, respectively.

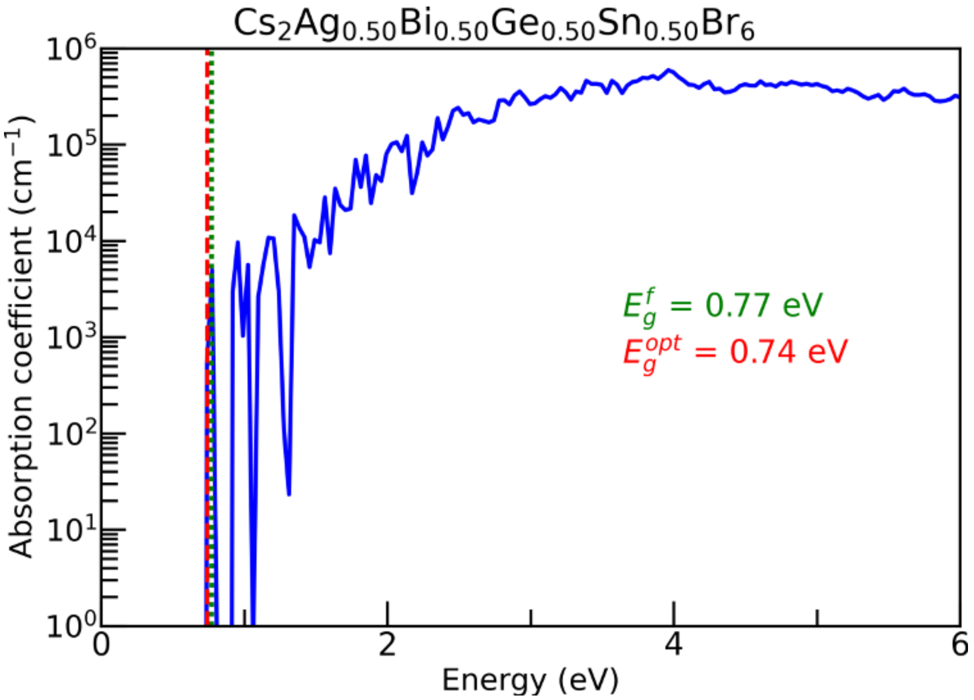

Figure S-166. Absorption coefficient at PBE+D3 + χ level of $Cs_2Ag_{0.50}Bi_{0.50}Ge_{0.50}Sn_{0.50}Br_6$. In green-dotted and red-dashed lines are indicated the optical ($E_g^{optical+\chi}$) and fundamental band gaps (E_g^f at PBE+D3+SOC+χ^{HSE06} level), here placed as insets simply as E_g^{opt} and E_g^f, respectively.

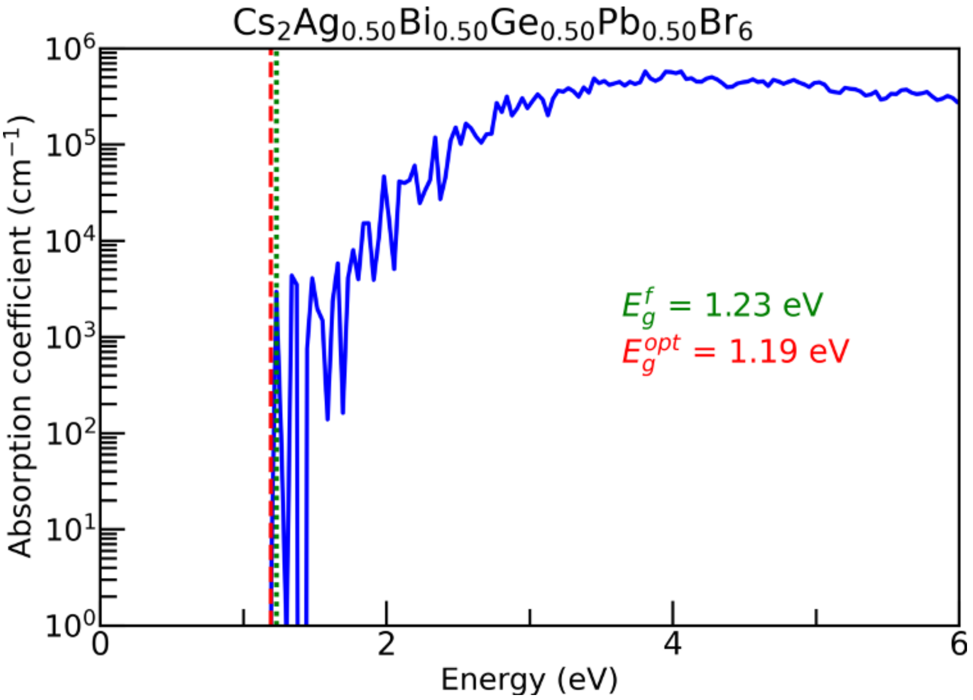

Figure S-167. Absorption coefficient at PBE+D3 + χ level of $Cs_2Ag_{0.50}Bi_{0.50}Ge_{0.50}Pb_{0.50}Br_6$. In green-dotted and red-dashed lines are indicated the optical ($E_g^{optical+\chi}$) and fundamental band gaps (E_g^f at PBE+D3+SOC+χ^{HSE06} level), here placed as insets simply as E_g^{opt} and E_g^f, respectively.

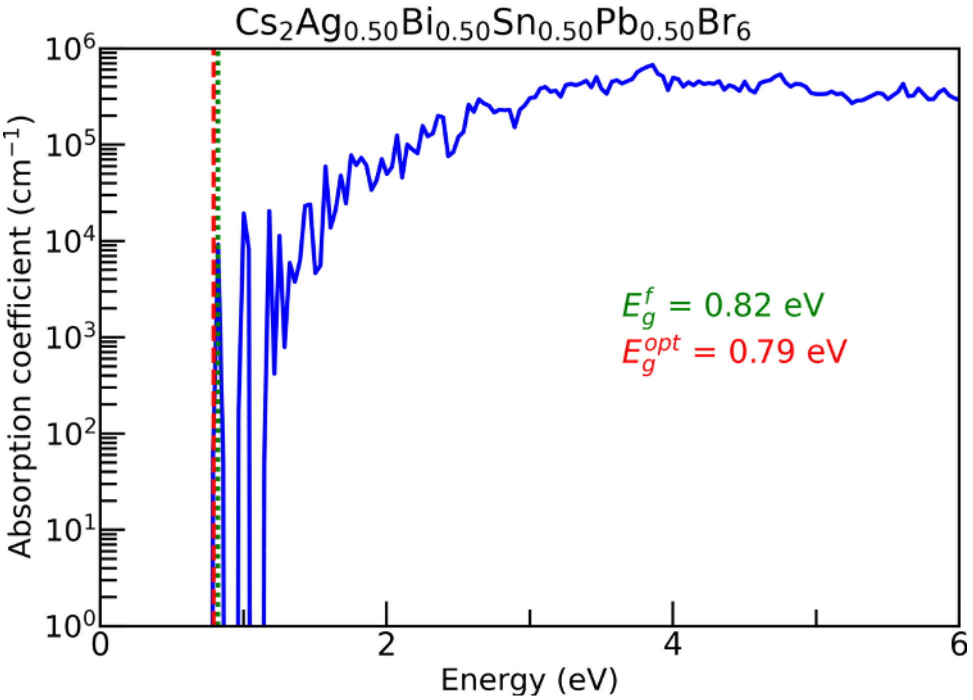

Figure S-168. Absorption coefficient at PBE+D3 + χ level of $Cs_2Ag_{0.50}Bi_{0.50}Sn_{0.50}Pb_{0.50}Br_6$. In green-dotted and red-dashed lines are indicated the optical ($E_g^{optical+\chi}$) and fundamental band gaps (E_g^f at PBE+D3+SOC+χ^{HSE06} level), here placed as insets simply as E_g^{opt} and E_g^f, respectively.

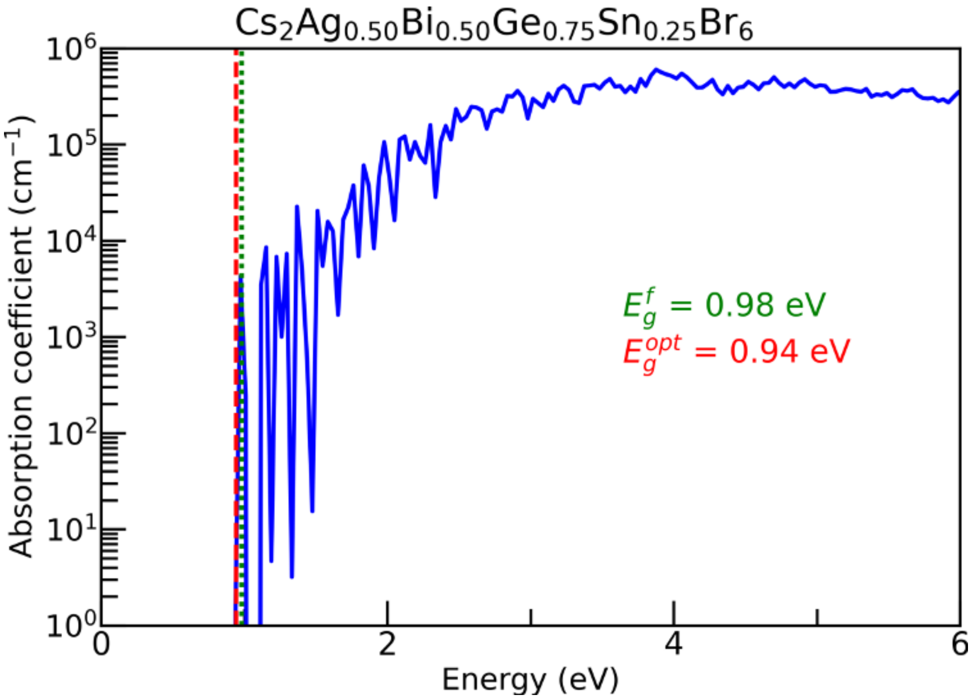

Figure S-169. Absorption coefficient at PBE+D3 + χ level of $Cs_2Ag_{0.50}Bi_{0.50}Ge_{0.75}Sn_{0.25}Br_6$. In green-dotted and red-dashed lines are indicated the optical ($E_g^{optical+\chi}$) and fundamental band gaps (E_g^f at PBE+D3+SOC+χ^{HSE06} level), here placed as insets simply as E_g^{opt} and E_g^f, respectively.

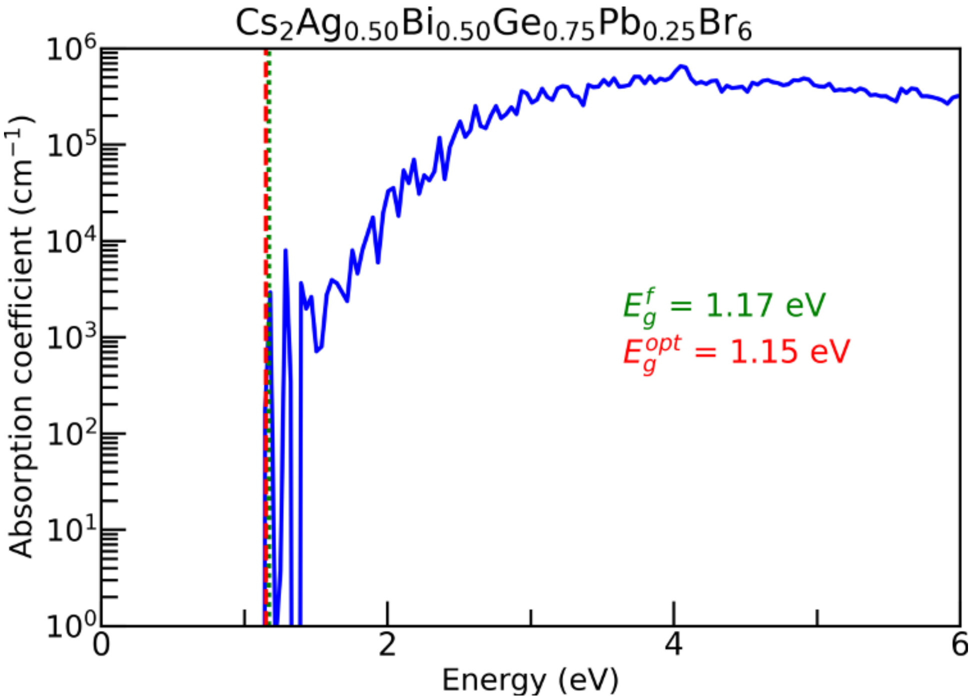

Figure S-170. Absorption coefficient at PBE+D3 + χ level of $Cs_2Ag_{0.50}Bi_{0.50}Ge_{0.75}Pb_{0.25}Br_6$. In green-dotted and red-dashed lines are indicated the optical ($E_g^{optical+\chi}$) and fundamental band gaps (E_g^f at PBE+D3+SOC+χ^{HSE06} level), here placed as insets simply as E_g^{opt} and E_g^f, respectively.

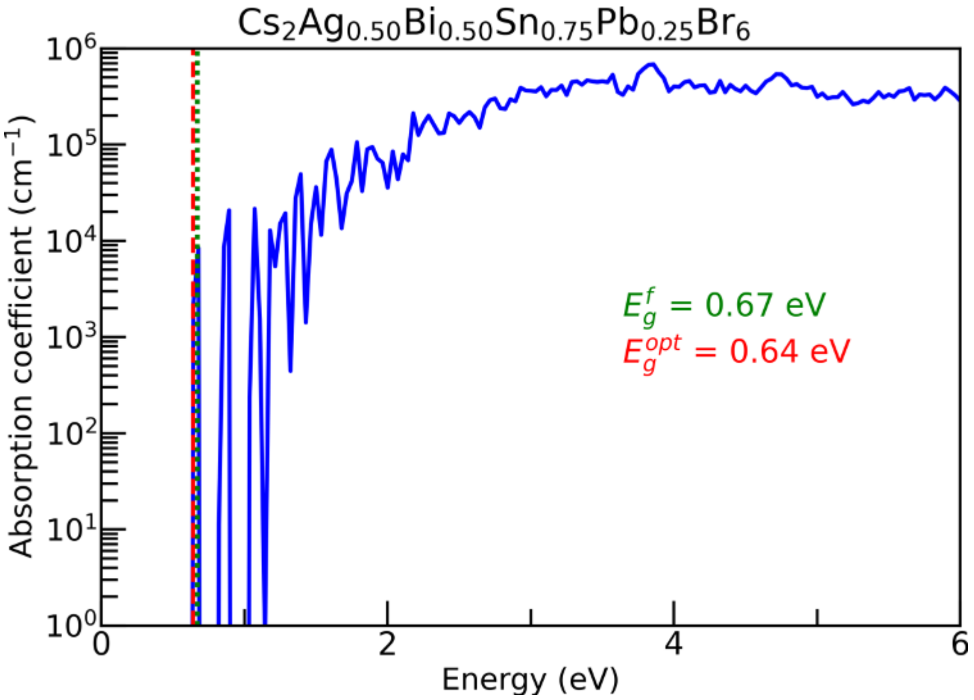

Figure S-171. Absorption coefficient at PBE+D3 + χ level of $Cs_2Ag_{0.50}Bi_{0.50}Sn_{0.75}Pb_{0.25}Br_6$. In green-dotted and red-dashed lines are indicated the optical ($E_g^{optical+\chi}$) and fundamental band gaps (E_g^f at PBE+D3+SOC+χ^{HSE06} level), here placed as insets simply as E_g^{opt} and E_g^f, respectively.

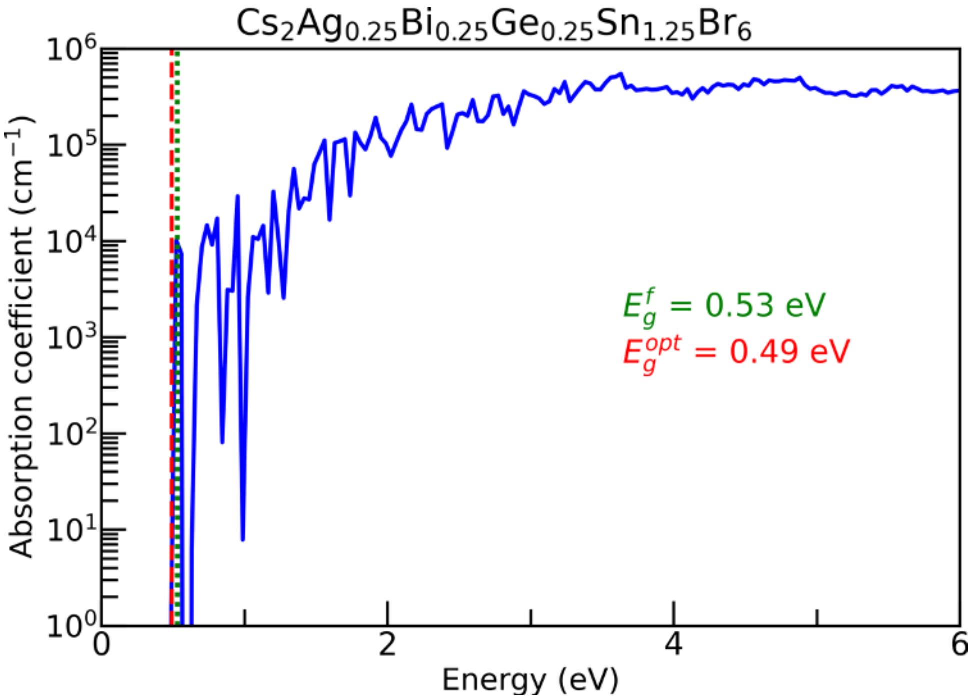

Figure S-172. Absorption coefficient at PBE+D3 + χ level of $Cs_2Ag_{0.25}Bi_{0.25}Ge_{0.25}Sn_{1.25}Br_6$. In green-dotted and red-dashed lines are indicated the optical ($E_g^{optical+\chi}$) and fundamental band gaps (E_g^f at PBE+D3+SOC+χ^{HSE06} level), here placed as insets simply as E_g^{opt} and E_g^f, respectively.

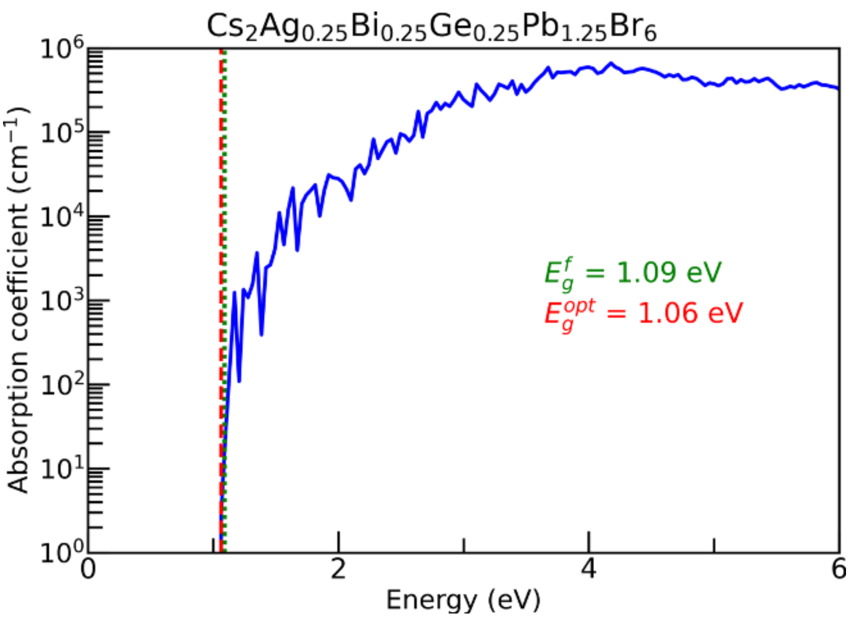

Figure S-173. Absorption coefficient at PBE+D3 + χ level of $Cs_2Ag_{0.25}Bi_{0.25}Ge_{0.25}Pb_{1.25}Br_6$. In green-dotted and red-dashed lines are indicated the optical ($E_g^{optical+\chi}$) and fundamental band gaps (E_g^f at PBE+D3+SOC+χ^{HSE06} level), here placed as insets simply as E_g^{opt} and E_g^f, respectively.

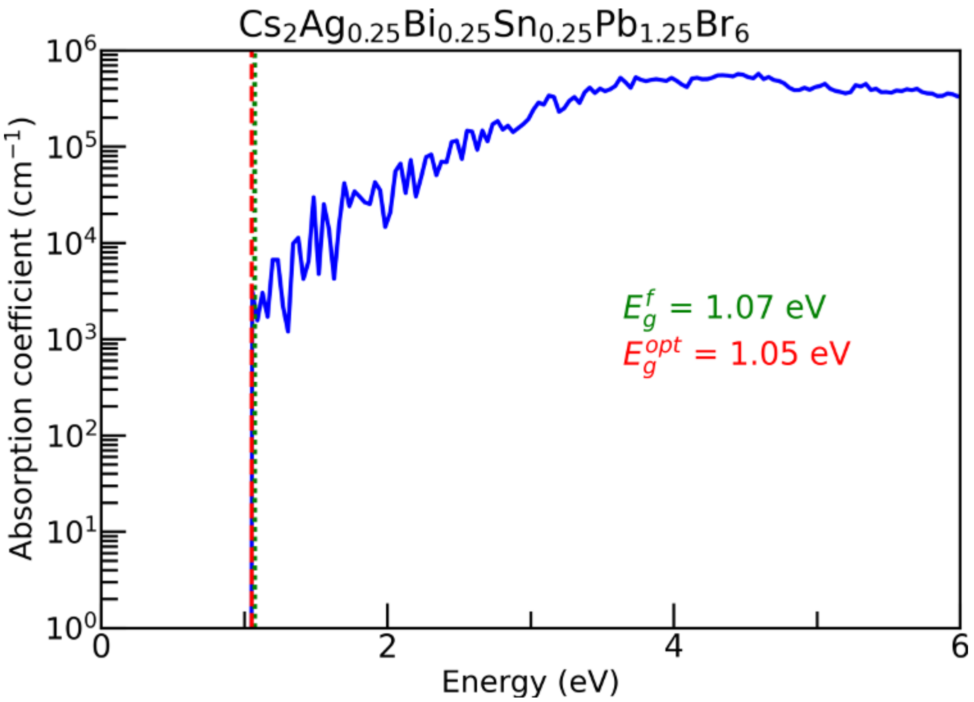

Figure S-174. Absorption coefficient at PBE+D3 + χ level of $Cs_2Ag_{0.25}Bi_{0.25}Sn_{0.25}Pb_{1.25}Br_6$. In green-dotted and red-dashed lines are indicated the optical ($E_g^{optical+\chi}$) and fundamental band gaps (E_g^f at PBE+D3+SOC+χ^{HSE06} level), here placed as insets simply as E_g^{opt} and E_g^f, respectively.

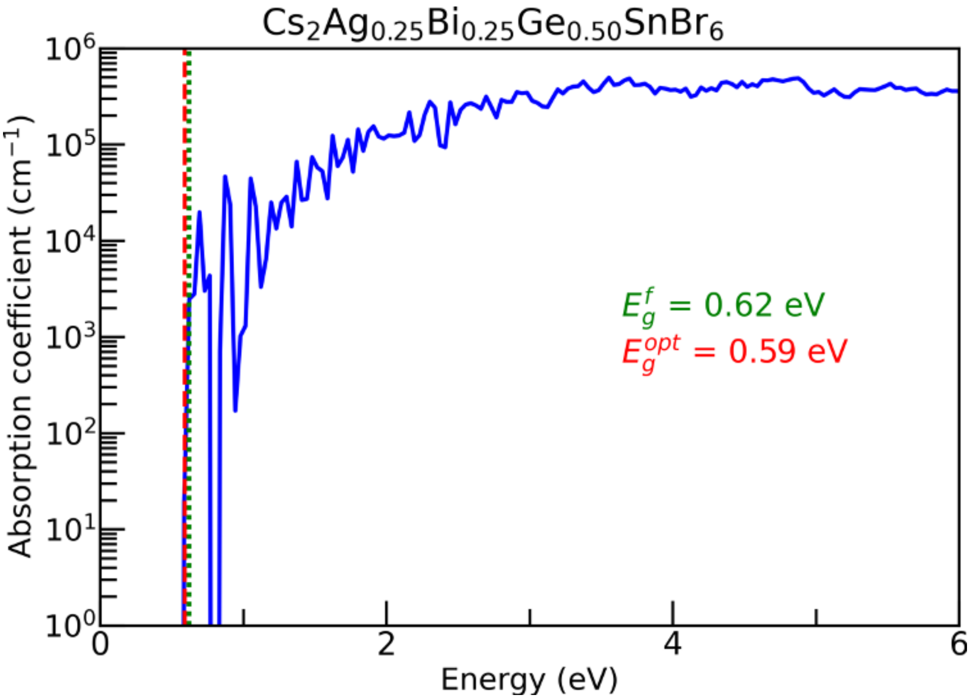

Figure S-175. Absorption coefficient at PBE+D3 + χ level of $Cs_2Ag_{0.25}Bi_{0.25}Ge_{0.50}SnBr_6$. In green-dotted and red-dashed lines are indicated the optical ($E_g^{optical+\chi}$) and fundamental band gaps (E_g^f at PBE+D3+SOC+χ^{HSE06} level), here placed as insets simply as E_g^{opt} and E_g^f, respectively.

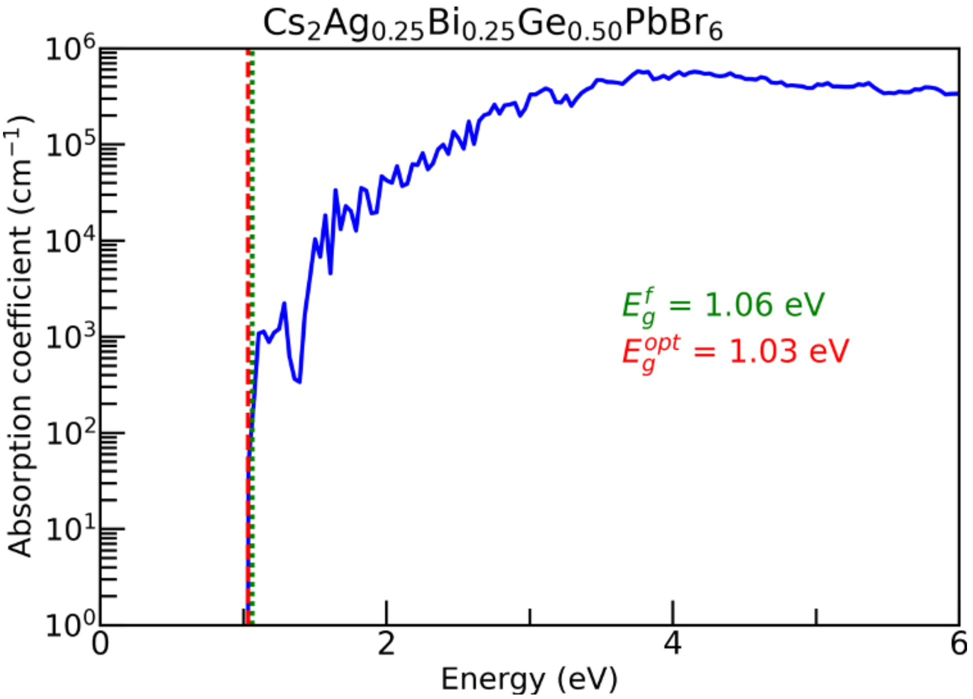

Figure S-176. Absorption coefficient at PBE+D3 + χ level of $Cs_2Ag_{0.25}Bi_{0.25}Ge_{0.50}PbBr_6$. In green-dotted and red-dashed lines are indicated the optical ($E_g^{optical+\chi}$) and fundamental band gaps (E_g^f at PBE+D3+SOC+χ^{HSE06} level), here placed as insets simply as E_g^{opt} and E_g^f, respectively.

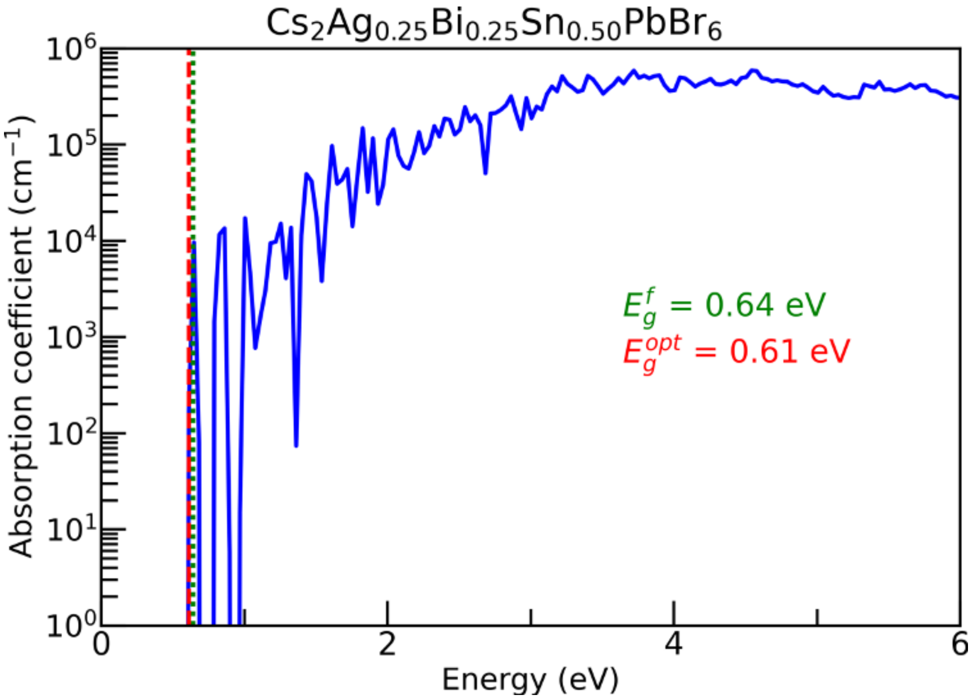

Figure S-177. Absorption coefficient at PBE+D3 + χ level of $Cs_2Ag_{0.25}Bi_{0.25}Sn_{0.50}PbBr_6$. In green-dotted and red-dashed lines are indicated the optical ($E_g^{optical+\chi}$) and fundamental band gaps (E_g^f at PBE+D3+SOC+χ^{HSE06} level), here placed as insets simply as E_g^{opt} and E_g^f, respectively.

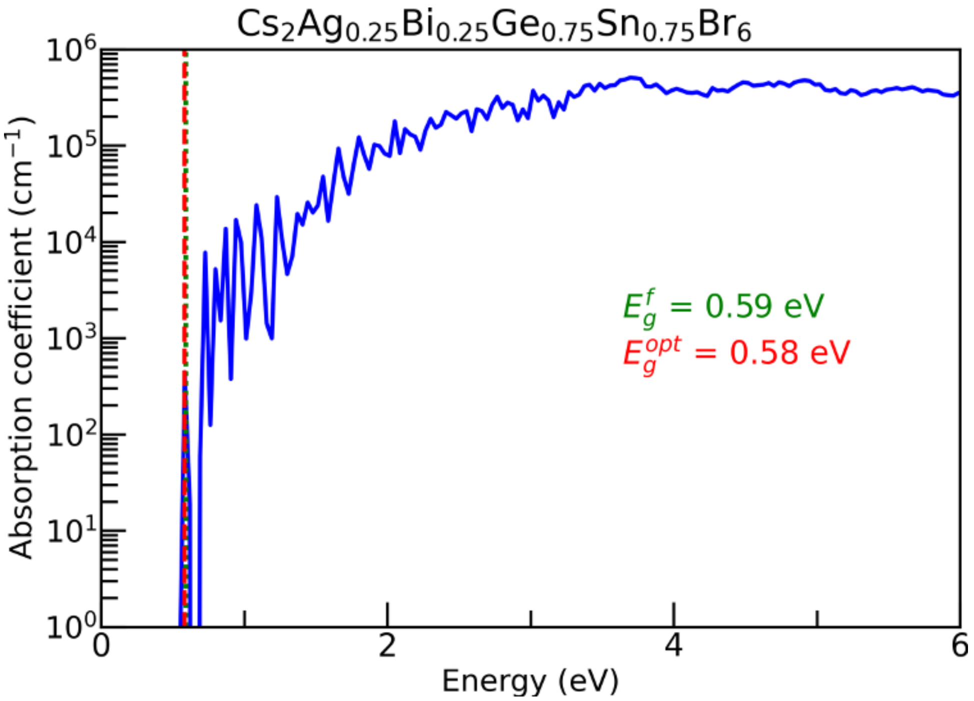

Figure S-178. Absorption coefficient at PBE+D3 + χ level of $Cs_2Ag_{0.25}Bi_{0.25}Ge_{0.75}Sn_{0.75}Br_6$. In green-dotted and red-dashed lines are indicated the optical ($E_g^{optical+\chi}$) and fundamental band gaps (E_g^f at PBE+D3+SOC+χ^{HSE06} level), here placed as insets simply as E_g^{opt} and E_g^f, respectively.

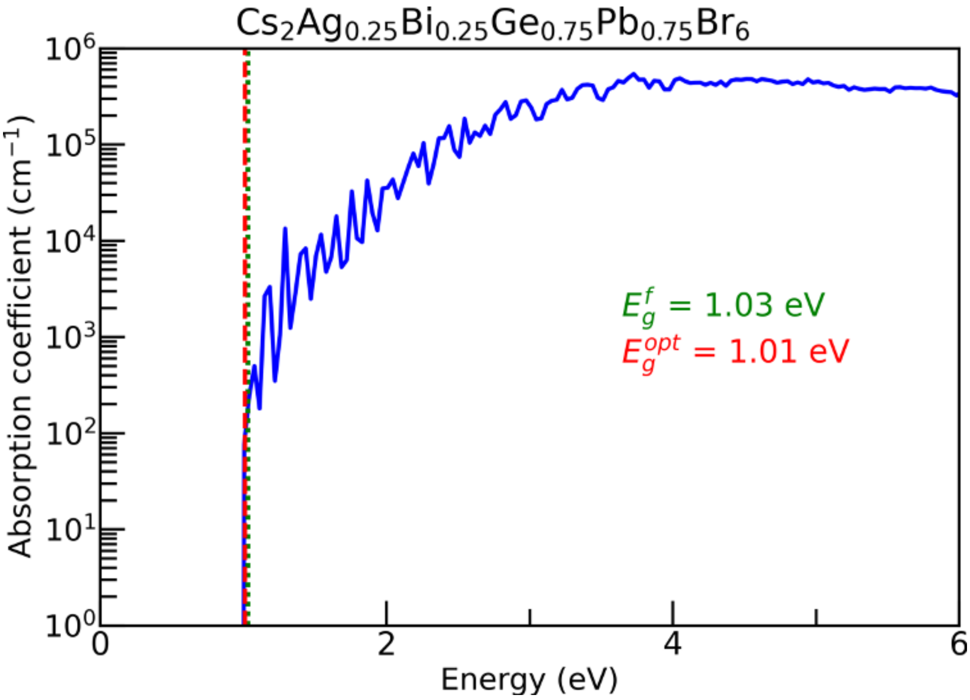

Figure S-179. Absorption coefficient at PBE+D3 + χ level of $Cs_2Ag_{0.25}Bi_{0.25}Ge_{0.75}Pb_{0.75}Br_6$. In green-dotted and red-dashed lines are indicated the optical ($E_g^{optical+\chi}$) and fundamental band gaps (E_g^f at PBE+D3+SOC+χ^{HSE06} level), here placed as insets simply as E_g^{opt} and E_g^f, respectively.

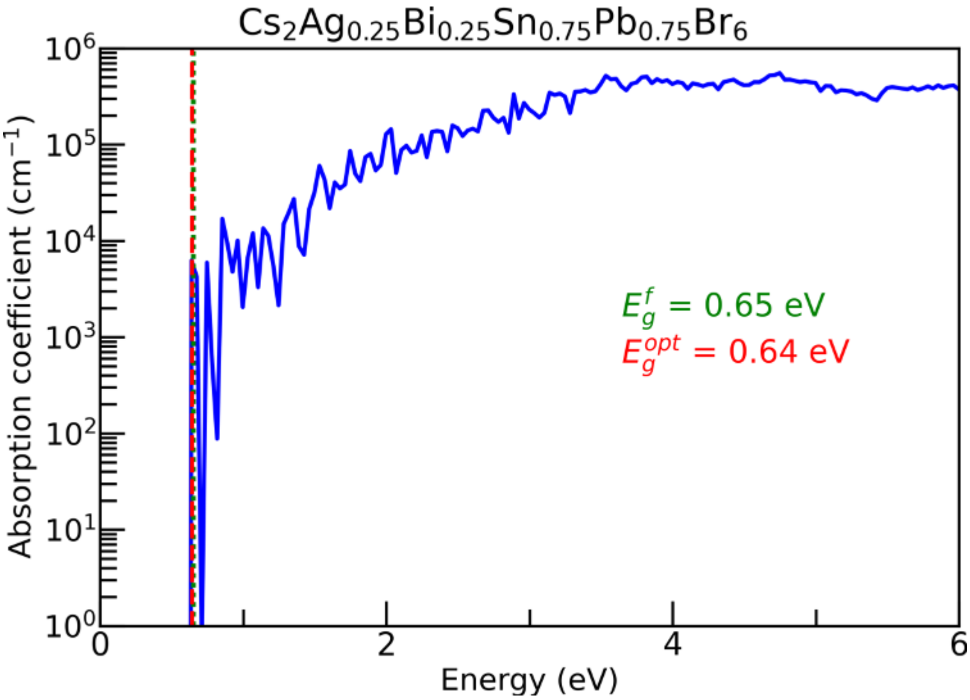

Figure S-180. Absorption coefficient at PBE+D3 + χ level of $Cs_2Ag_{0.25}Bi_{0.25}Sn_{0.75}Pb_{0.75}Br_6$. In green-dotted and red-dashed lines are indicated the optical ($E_g^{optical+\chi}$) and fundamental band gaps (E_g^f at PBE+D3+SOC+χ^{HSE06} level), here placed as insets simply as E_g^{opt} and E_g^f, respectively.

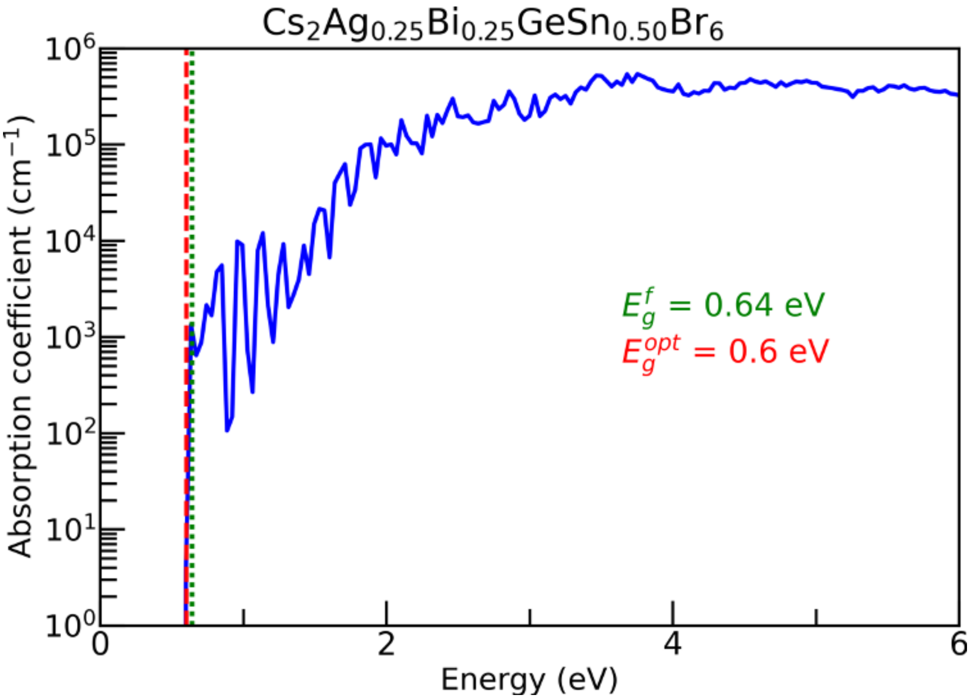

Figure S-181. Absorption coefficient at PBE+D3 + χ level of $Cs_2Ag_{0.25}Bi_{0.25}GeSn_{0.50}Br_6$. In green-dotted and red-dashed lines are indicated the optical ($E_g^{optical+\chi}$) and fundamental band gaps (E_g^f at PBE+D3+SOC+χ^{HSE06} level), here placed as insets simply as E_g^{opt} and E_g^f, respectively.

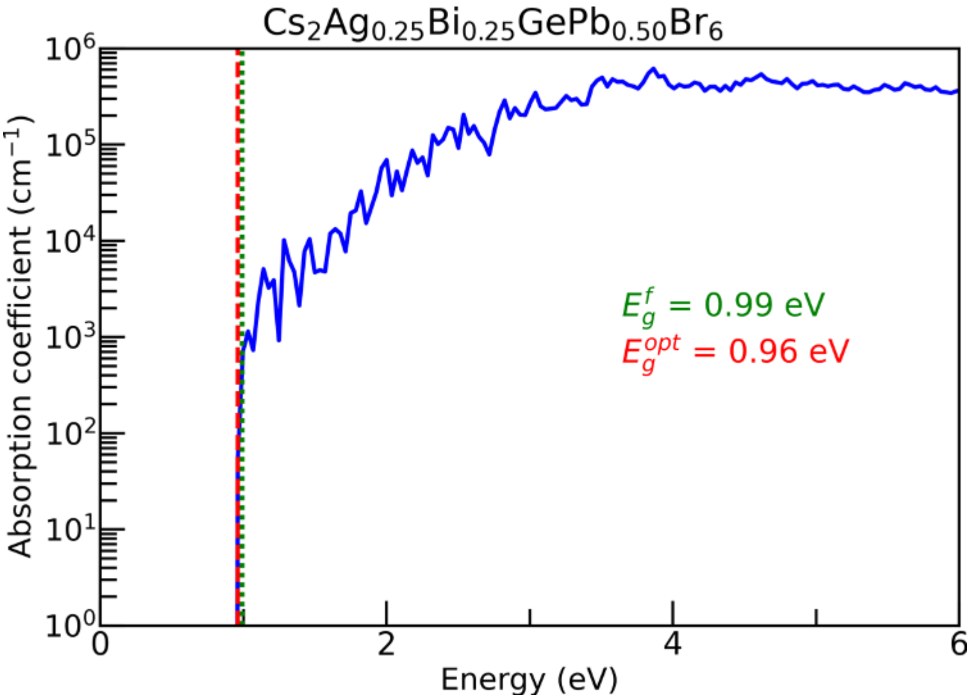

Figure S-182. Absorption coefficient at PBE+D3 + χ level of $Cs_2Ag_{0.25}Bi_{0.25}GePb_{0.50}Br_6$. In green-dotted and red-dashed lines are indicated the optical ($E_g^{optical+\chi}$) and fundamental band gaps (E_g^f at PBE+D3+SOC+χ^{HSE06} level), here placed as insets simply as E_g^{opt} and E_g^f, respectively.

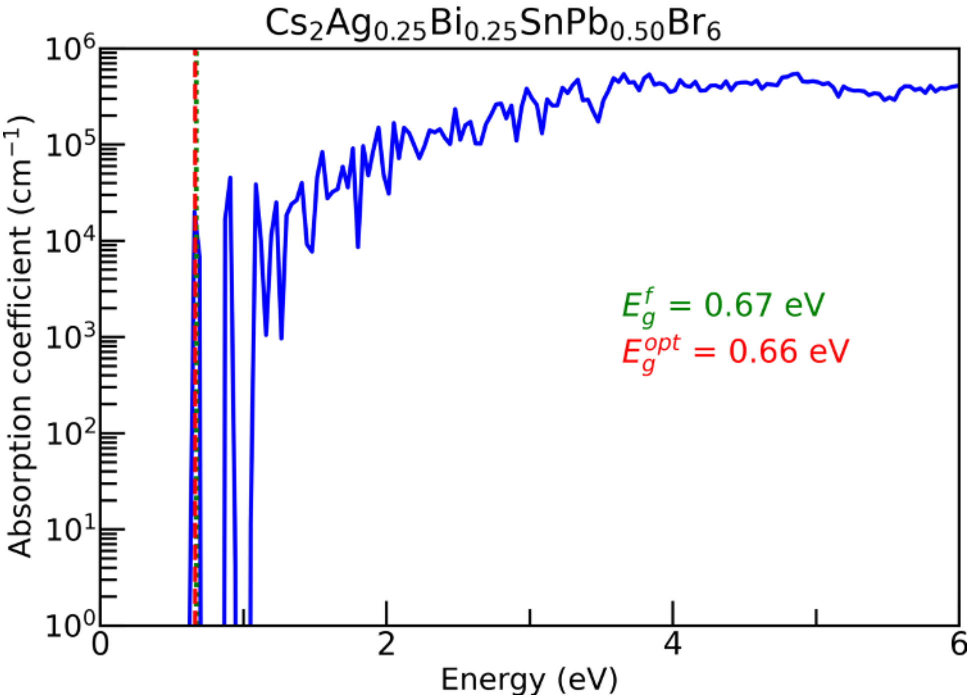

Figure S-183. Absorption coefficient at PBE+D3 + χ level of $Cs_2Ag_{0.25}Bi_{0.25}SnPb_{0.50}Br_6$. In green-dotted and red-dashed lines are indicated the optical ($E_g^{optical+\chi}$) and fundamental band gaps (E_g^f at PBE+D3+SOC+χ^{HSE06} level), here placed as insets simply as E_g^{opt} and E_g^f, respectively.

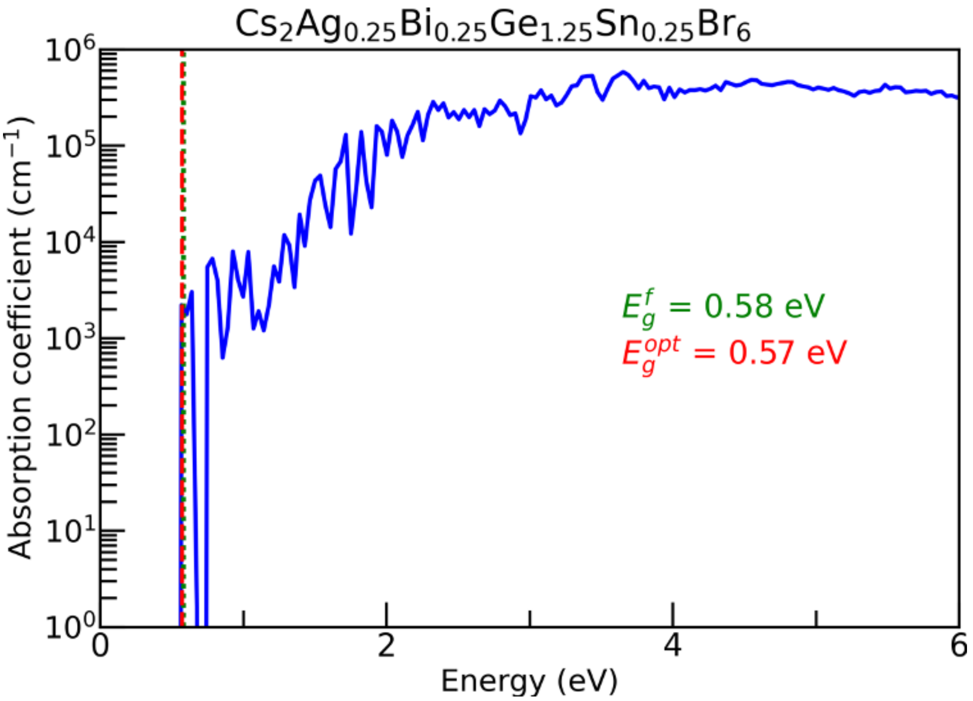

Figure S-184. Absorption coefficient at PBE+D3 + χ level of $Cs_2Ag_{0.25}Bi_{0.25}Ge_{1.25}Sn_{0.25}Br_6$. In green-dotted and red-dashed lines are indicated the optical ($E_g^{optical+\chi}$) and fundamental band gaps (E_g^f at PBE+D3+SOC+χ^{HSE06} level), here placed as insets simply as E_g^{opt} and E_g^f, respectively.

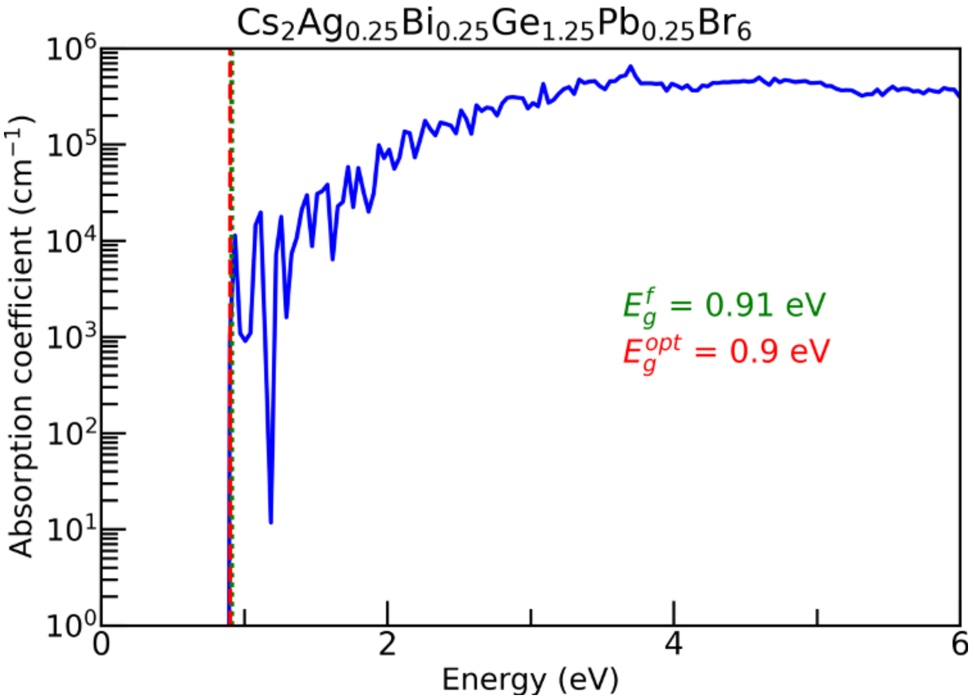

Figure S-185. Absorption coefficient at PBE+D3 + χ level of $Cs_2Ag_{0.25}Bi_{0.25}Ge_{1.25}Pb_{0.25}Br_6$. In green-dotted and red-dashed lines are indicated the optical ($E_g^{optical+\chi}$) and fundamental band gaps (E_g^f at PBE+D3+SOC+χ^{HSE06} level), here placed as insets simply as E_g^{opt} and E_g^f, respectively.

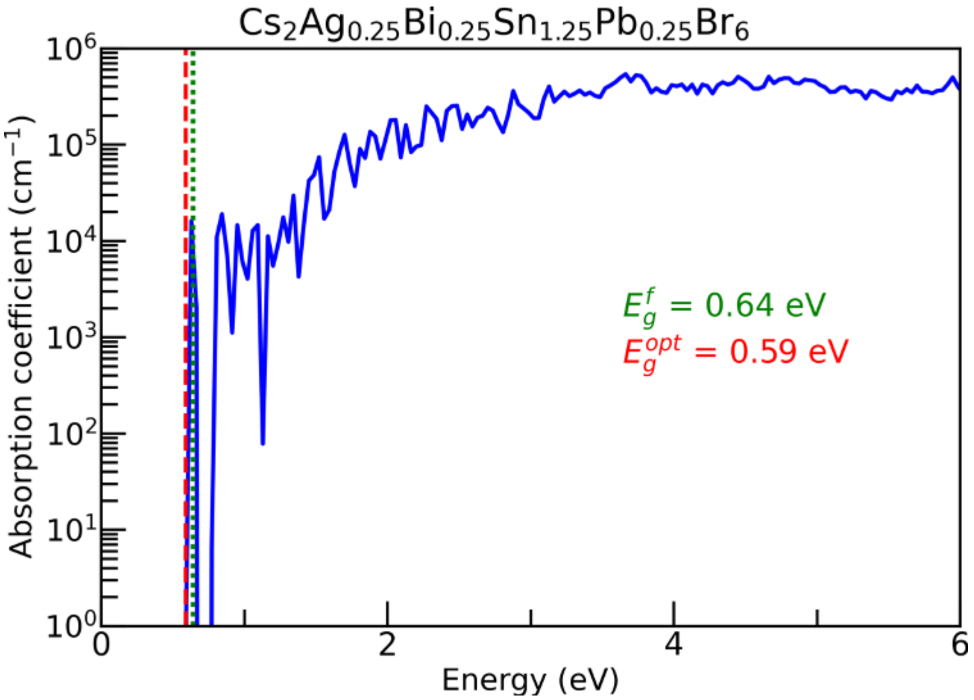

Figure S-186. Absorption coefficient at PBE+D3 + χ level of $Cs_2Ag_{0.25}Bi_{0.25}Sn_{1.25}Pb_{0.25}Br_6$. In green-dotted and red-dashed lines are indicated the optical ($E_g^{optical+\chi}$) and fundamental band gaps (E_g^f at PBE+D3+SOC+χ^{HSE06} level), here placed as insets simply as E_g^{opt} and E_g^f, respectively.

References

1 Hohenberg, P.; Kohn, W. Inhomogeneous Electron Gas. *Phys. Rev.* **1964**, *136*, B864–B871, DOI: 10.1103/physrev.136.b864.

2 Kohn, W.; Sham, L. J. Self-Consistent Equations Including Exchange and Correlation Effects. *Phys. Rev.* **1965**, *140*, A1133–A1138, DOI: 10.1103/physrev.140.a1133.

3 Slavney, A. H.; Hu, T.; Lindenberg, A. M.; Karunadasa, H. I. A Bismuth-Halide Double Perovskite with Long Carrier Recombination Lifetime for Photovoltaic Applications. *J. Am. Chem. Soc.* **2016**, *138*, 2138–2141, DOI: 10.1021/jacs.5b13294.

4 McClure, E. T.; Ball, M. R.; Windl, W.; Woodward, P. M. Cs_2AgBiX_6 (X = Br, Cl): New Visible Light Absorbing, Lead-Free Halide Perovskite Semiconductors. *Chem. Mater.* **2016**, *28*, 1348–1354, DOI: 10.1021/acs.chemmater.5b04231.

5 Lejaeghere, K.; Bihlmayer, G.; Bjorkman, T.; Blaha, P.; Blügel, S.; Blum, V.; Caliste, D.; Castelli, I. E.; Clark, S. J.; Dal Corso, A.; de Gironcoli, S.; Deutsch, T.; Dewhurst, J. K.; Di Marco, I.; Draxl, C.; Dułak, M.; Eriksson, O.; Flores-Livas, J. A.; Garrity, K. F.; Genovese, L.; Giannozzi, P.; Giantomassi, M.; Goedecker, S.; Gonze, X.; Grånäs, O.; Gross, E. K. U.; Gulans, A.; Gygi, F.; Hamann, D. R.; Hasnip, P. J.; Holzwarth, N. A. W.; Iuşan, D.; Jochym, D. B.; Jollet, F.; Jones, D.; Kresse, G.; Koepernik, K.; Küçükbenli, E.; Kvashnin, Y. O.; Locht, I. L. M.; Lubeck, S.; Marsman, M.; Marzari, N.; Nitzsche, U.; Nordström, L.; Ozaki, T.; Paulatto, L.; Pickard, C. J.; Poelmans, W.; Probert, M. I. J.; Refson, K.; Richter, M.; Rignanese, G.-M.; Saha, S.; Scheffler, M.; Schlipf, M.; Schwarz, K.; Sharma, S.; Tavazza, F.; Thunström, P.; Tkatchenko, A.; Torrent, M.; Vanderbilt, D.; van Setten, M. J.; Van Speybroeck, V.; Wills, J. M.; Yates, J. R.; Zhang, G.-X.; Cottenier, S. Reproducibility in Density Functional Theory Calculations of Solids. *Science* **2016**, *351*, aad3000, DOI: 10.1126/science.aad3000.

6 Dias, A. C.; Lima, M. P.; Da Silva, J. L. F. Role of Structural Phases and Octahedra Distortions in the Optoelectronic and Excitonic Properties of $CsGeX_3$ (X = Cl, Br,

I) Perovskites. *J. Phys. Chem. C* **2021**, *125*, 19142–19155, DOI: 10.1021/acs.jpcc.1c05245.

7 dos Santos, R. M.; Ornelas-Cruz, I.; Dias, A. C.; Lima, M. P.; Silva, J. L. F. D. Theoretical Investigation of the Role of Mixed A^+ Cations in the Structure, Stability, and Electronic Properties of Perovskite Alloys. *ACS Appl. Energy Mater.* **2023**, *6*, 5259–5273, DOI: 10.1021/acsaem.3c00186.

8 Corso, A. D. Reciprocal Space Integration and Special-Point Techniques. In *Quantum-Mechanical Ab-initio Calculation of the Properties of Crystalline Materials*; Pisani, C., Ed.; Springer Berlin Heidelberg: Berlin, Heidelberg, 1996; pp 77–89, DOI: 10.1007/978-3-642-61478-1_4.

9 Schwarz, U.; Hillebrecht, H.; Kaupp, M.; Syassen, K.; von Schnering, H.-G.; Thiele, G. Pressure-induced Phase Transition in $CsGeBr_3$ Studied by X-Ray Diffraction and Raman Spectroscopy. *J. Solid State Chem.* **1995**, *118*, 20–27, DOI: 10.1006/jssc.1995.1305.

10 Fabini, D. H.; Laurita, G.; Bechtel, J. S.; Stoumpos, C. C.; Evans, H. A.; Kontos, A. G.; Raptis, Y. S.; Falaras, P.; der Ven, A. V.; Kanatzidis, M. G.; Seshadri, R. Dynamic Stereochemical Activity of the Sn^{2+} Lone Pair in Perovskite $CsSnBr_3$. *J. Am. Chem. Soc.* **2016**, *138*, 11820–11832, DOI: 10.1021/jacs.6b06287.

11 Cottinghama, P.; Brutchey, R. L. On the crystal structure of colloidally prepared $CsPbBr_3$ quantum dots. *J. Am. Chem. Soc.* **2016**, *52*, 5246–5249, DOI: 10.1039/C6CC01088A.

12 Kresse, G.; Furthmüller, J. Efficiency of ab-initio total energy calculations for metals and semiconductors using a plane-wave basis set. *Comput. Mater. Sci.* **1996**, *6*, 15–50, DOI: 10.1016/0927-0256(96)00008-0.

13 Payne, M. C.; Teter, M. P.; Allan, D. C.; Arias, T. A.; Joannopoulos, J. D. Iterative Minimization Techniques for Ab initio Total-Energy Calculations Molecular Dynamics and Conjudate Gradients. *Rev. Mod. Phys.* **1992**, *64*, 1045.
